# Cellular Mechanisms Triggered by the Cotreatment of Resveratrol and Doxorubicin in Breast Cancer: A Translational In Vitro–In Silico Model

**DOI:** 10.1155/2020/5432651

**Published:** 2020-11-01

**Authors:** José Eduardo Vargas, Renato Puga, Guido Lenz, Cristiano Trindade, Eduardo Filippi-Chiela

**Affiliations:** ^1^Instituto de Ciências Biológicas, Universidade de Passo Fundo, Brazil; ^2^Hospital de Clínicas de Porto Alegre, Porto Alegre, Brazil; ^3^Hospital Israelita Albert Einstein, São Paulo, Brazil; ^4^Centro de Biotecnologia e Departamento de Biofísica, Universidade Federal do Rio Grande do Sul (UFRGS), Porto Alegre, Brazil; ^5^Facultad de Ciencias Básicas y Biomédicas, Universidad Simón Bolívar, Barranquilla, Colombia; ^6^Departamento de Ciências Morfológicas, Instituto de Ciências Básicas da Saúde, Universidade Federal do Rio Grande do Sul, Porto Alegre, RS, Brazil

## Abstract

Doxorubicin (Doxo) is the most effective chemotherapeutic agent for the treatment of breast cancer. However, resistance to Doxo is common. Adjuvant compounds capable of modulating mechanisms involved in Doxo resistance may potentiate the effectiveness of the drug. Resveratrol (Rsv) has been tested as an adjuvant in mammary malignancies. However, the cellular and molecular mechanisms underlying the effects of cotreatment with Doxo and Rsv in breast cancer are poorly understood. Here, we combined in vitro and in silico analysis to characterize these mechanisms. In vitro, we employed a clinically relevant experimental design consisting of acute (24 h) treatment followed by 15 days of analysis. Acute Rsv potentiated the long-lasting effect of Doxo through the induction of apoptosis and senescence. Cells that survived to the cotreatment triggered high levels of autophagy. Autophagy inhibition during its peak of activation but not concomitant with Doxo+Rsv increased the long-term toxicity of the cotreatment. To uncover key proteins potentially associated with in vitro effects, an in silico multistep strategy was implemented. Chemical-protein networks were predicted based on constitutive gene expression of MCF7 cells and interatomic data from breast cancer. Topological analysis, KM survival analysis, and a quantitative model based on the connectivity between apoptosis, senescence, and autophagy were performed. We found seven putative genes predicted to be modulated by Rsv in the context of Doxo treatment: CCND1, CDH1, ESR1, HSP90AA1, MAPK3, PTPN11, and RPS6KB1. Six out of these seven genes have been experimentally proven to be modulated by Rsv in cancer cells, with 4 of the 6 genes in MCF7 cells. In conclusion, acute Rsv potentiated the long-term toxicity of Doxo in breast cancer potentially through the modulation of genes and mechanisms involved in Doxo resistance. Rational autophagy inhibition potentiated the effects of Rsv+Doxo, a strategy that should be further tested in animal models.

## 1. Introduction

Despite substantial progress over the last years in terms of early diagnosis and treatment, breast cancer remains one of the leading causes of cancer deaths among women globally [1]. Around 20 to 30% of breast cancer patients develop stage IV, metastatic cancers, for which the 5-year survival rate is only 22%. Furthermore, 90% of the mortality among breast cancer patients is associated with metastatic diseases, which are resistant to adjuvant therapy [2]. The main subtypes of breast cancer are based on the expression status of estrogen receptor (ER), progesterone receptor (PR), and human epidermal growth factor 2-neu (HER2). Approximately 75% of cases are classified as hormone receptor-positive (HR+, ER+, and/or PR+) and HER2-negative [3]. HER2-positive cases account for 20% of breast cancers and show more aggressive clinical outcomes due to limited response to chemotherapy. Triple-negative breast cancers (TNBC), which account for 10%–15% of all cases, are usually marked by aggressive behaviour [4]. Underlying this aggressiveness, the cooccurrence of multiple alterations in typical cellular hallmarks of cancer can make the treatment of breast cancer particularly difficult [5].

Data from TCGA have revealed the significant molecular heterogeneity of mammary malignancies, with four main classes according to data from genomes, DNA methylation, exome sequencing, microRNA sequencing, and protein arrays [6, 7]. This large heterogeneity makes the employment of advanced therapies difficult, so that classical chemotherapeutics, along with surgery and radiotherapy, remain the primary choices for breast cancer therapy [8]. According to the National Comprehensive Cancer Network Guidelines, the naturally occurring anthracycline doxorubicin (Doxo) is the preferred single agent for the treatment of breast cancer [9]. In the clinics, the first-choice regimens for Doxo involve 24-hour treatment, followed by a recovery period for the patient. However, like healthy tissues, tumor cells can also recover during this period. This can lead to cancer resistance and relapse, which are common in Doxo-treated breast cancer patients [10].

Indeed, resistance to Doxo is one of the major obstacles to the effective treatment of breast cancer. Therefore, understanding the cellular mechanisms underlying the response and resistance triggered after the treatment is fundamental for planning rational strategies to increase Doxo efficacy [5]. Given this context, the natural polyphenol resveratrol (Rsv) has emerged in recent years as an alternative treatment, which can be combined with Doxo in order to increase the sensitivity of tumor cells to the anthracycline and overcome tumor resistance [11–13]. The toxicity and possible mechanisms of action of Rsv and its analogs in breast cancer cells have been demonstrated in several preclinical studies [14, 15]. Multiple cellular and molecular mechanisms are involved in the toxicity of Rsv, including the suppression of oncogenic pathways and the activation of tumor suppressors. As a consequence, Rsv is capable to suppress cell proliferation, trigger cell death activation, and attenuate cancer invasion [16]. As an adjuvant to other drugs in mammary malignancies, the effect of Rsv varies. The polyphenol chemosensitizes breast cancer cells to docetaxel [17] but has been shown to attenuate the efficacy of paclitaxel [18]. In normal epithelial cells, in turn, Rsv has a protective, antioxidant effect and attenuated the cardiotoxicity triggered by Doxo, which is the main side effect limiting the use of this drug [19, 20].

The first clinical report assessing the effect of Rsv in breast cancer suggested that this compound is able to modulate some of the key components of breast carcinogenesis, confirming its potential not only in preclinical studies but also in patients [21]. In addition, new pharmacological formulations of Rsv and Doxo can be used to increase the amounts of these compounds delivered to tumor cells, with consequent increases in effectiveness in vivo [13]. Thus, the anticarcinogenic effects of Rsv need to be elucidated in the context of Doxo treatment, especially considering the long-term effects of acute treatment, the crosstalk between cellular mechanisms involved in this response, and the putative molecular players involved in the response to the cotreatment. In the current study, we explored the effects of Rsv in potentiating Doxo toxicity in breast cancer using a clinically relevant experimental design addressing the crosstalk between autophagy, apoptosis, and senescence, in combination with a system biology approach to uncover the molecular mechanisms involved in the response to this cotreatment.

## 2. Material and Methods

### 2.1. In Vitro

#### 2.1.1. Cell Culture

Experiments were performed using the MCF7 human breast cancer cell line using exponentially growing cells never exceeding P25. Cells were kindly provided by prof. Andréa Buffon (Faculty of Pharmacy, Universidade Federal do Rio Grande do Sul). Cells were maintained in a humidified incubator with Dulbecco's Modified Eagle's Medium (DMEM) supplemented with 10% fetal bovine serum (FBS) (Gibco/Invitrogen, São Paulo, SP Brazil), along with 1% of penicillin/streptomycin, and 0.1% of amphotericin B (Sigma-Aldrich, St. Louis, MO, USA) at 37°C and 5% CO_2_.

#### 2.1.2. Drugs and Treatments

Cells were treated with resveratrol (Rsv, 10, 30, 60, and 120 *μ*M; Sigma-Aldrich, MO) and doxorubicin (Doxo, 100 and 200 nM; Sigma-Aldrich, MO) for 24 h. Control cells were treated with DMSO (vehicle) not exceeding 0.05%. After this, cells were washed twice with PBS 1x and then replated and grown in a complete, Drug-Free Medium (DFM) for 15 days. During cell growth in a DFM, analyses were performed as indicated in the subsections below. To suppress autophagy, cells were treated with 3-methyladenine (3-MA, Sigma-Aldrich, MO) 2 mM for 1 h before treatments or after cells replating in a DFM.

#### 2.1.3. Cell Viability Using Trypan Blue Exclusion Assay

Trypan blue is a vital dying that accumulates in cells losing viability [22]. The dye exclusion test is used to determine the number of viable cells present in a cell suspension. After 24 h of treatment, cells were harvested and suspended in PBS containing trypan blue (1 : 1). Then, the percentage of trypan blue-positive cells was determined using a hemocytometer. We also evaluated the Combination Index using the CompuSyn Software (http://www.combosyn.com/).

#### 2.1.4. Cumulative Population Doubling

After 24 h of treatment with Rsv, Doxo, or the cotreatment, cells were washed 3 times with PBS 1x, harvested, and seeded in a DFM in a 24-well plate. After 5, 10, and 15 days, the number of cells and the Cumulative Population Doubling (CPD) were determined, as previously described [23], according to the formula PD = [log *N*(*t*) − log*N*(to)]/log2, where *N*(*t*) is the number of cells per well at the time of the count (passage) and *N*(to) is the initial number of the cells. The sum of PDs was then plotted against the time of culture. We measured CPD also after autophagy inhibition using 3-MA.

#### 2.1.5. Cell Death Assays

*(1) Annexin V-FITC/Propidium Iodide*. Cell death (apoptosis and necrosis) was measured 5 days after cell replating in a DFM. To do this, we costained cells with Annexin V-FITC plus Propidium Iodide (PI) according to the manufacturer's protocol (BD Biosciences; CA, USA). Briefly, the supernatant and trypsinized cells were transferred to an eppendorf, centrifuged at 1400 rpm for 5 min, washed with 1x PBS, and centrifuged again at 1400 rpm for 5 min. The supernatant was discarded, and the annexin-binding buffer containing annexin (2.5 *μ*L/sample) and PI (3 *μ*M/sample) was added to the pellet. Cells were incubated at room temperature, in the dark, for 15 min. Stained cells were analyzed using the Attune flow cytometer (Attune-AB Applied Biosystems).

*(2) Active Caspase-3*. To measure active caspase-3 in MCF7 cells, we used the PE Active Caspase-3 Apoptosis Kit (BD Pharmingen), according to the manufacturer's instructions. Briefly, MCF7 cells were harvested and then centrifuged at 1200 rpm for 6 min. Then, cells were washed twice with PBS 1x and resuspended in BD Cytofix/Cytoperm™ solution of 3 × 10^4^ cells per 100 *μ*L and incubated for 20 min at 4°C. Afterwards, the cells were washed twice with BD Perm/Wash™ buffer (1x) at room temperature. Finally, the cells were incubated in BD Perm/Wash™ buffer (1x) plus the antibody against active caspase-3 for 30 min at room temperature in the dark. Stained cells were analyzed by flow cytometry (Attune Cytometry, BD Biosciences).

#### 2.1.6. Autophagy Assays

*(1) Acridine Orange*. Acridine orange (AO) is a marker of acidic vacuolar organelles that fluoresces green in the whole cell (cytoplasm and nucleus), but in acidic compartments (mainly autolysosomes), it suffers protonation and accumulates and emits red fluorescence. Thus, AO has been used as a marker of late autophagy [24]. To perform the AO experiment, cells were trypsinized and incubated with 2.7 *μ*M of AO for 15 min, in DMEM, at room temperature. After this, cells were analyzed by flow cytometry (Attune-AB applied biosystems). Data are presented as the percentage of AO-positive cells and red AO intensity.

*(2) SQSTM1 Levels (Flow Cytometry)*. After the treatment, cells were harvested, washed twice with ice-cold PBS (1x), and fixed with 4% paraformaldehyde in ice-cold PBS (1x) for 5 min. Cells were centrifuged at 1200 rpm for 5 min and washed again in ice-cold PBS (1x). Then, cells were incubated for 30 min at 4°C with staining solution (mix per sample: 200 *μ*L ice-cold PBS (1x)+4 *μ*L FBS+1 *μ*L Mouse Anti-SQSTM1 antibody (Abcam, ab56416)). Isotype-control IgG1 (BD Biosciences; CA, USA) was used as control (mix per sample: 200 *μ*L ice-cold PBS (1x)+4 *μ*L FBS+1 *μ*L isotype-control IgG1). Next, cells were washed twice with ice-cold PBS and incubated with the secondary goat anti-mouse marked with Alexa 488 for 1 h. After, cells were centrifuged for 5 min at 1200 rpm and resuspended in ice-cold PBS (1x). Samples were analyzed by flow cytometry (Attune-AB Applied Biosystems).

#### 2.1.7. Senescence Assays–Chromogenic SA-*β*-Gal and C12-FDG Staining

Senescence was assessed 15 days after treatment. To this, cells were incubated with 33 *μ*M of 5-dodecanoylaminofluorescein di-betaD-galactopiranoside (C12-FDG, Life Technologies) for 2 h in the CO_2_ incubator. C12-FDG is a substrate to the Senescence-Associated Acid *β*-Galactosidase (SA-*β*-gal) that emits green fluorescence when cleaved by the enzyme. Stained cells were trypsinized and analyzed using the flow cytometer (Attune-AB Applied Biosystems). Alternatively, the activity of SA-*β*-gal was also evaluated through the chromogenic assay using the substrate X-gal (Sigma-Aldrich), as described [25].

#### 2.1.8. Nuclear Morphometric Analysis (NMA)

Nuclear morphometric analysis was performed as described by our group to screen cell fate (i.e., apoptosis, senescence, or mitotic catastrophe) based on nuclear shape and size [26]. Briefly, treated cells were fixed with 4% paraformaldehyde and stained with DAPI 300 nM at room temperature in the dark. Images were acquired in a fluorescence microscope, followed by analysis in the Image-Pro Plus 6.0 software (IPP6, Media Cybernetics). The nuclear contours were delimited using the magic wand tool, followed by the acquisition of the following variables: area, Radiusratio (Rr), Roundness (Rou), Aspect (Asp), and Areabox (Arbx). After the acquisition, data were transferred to a spreadsheet available at http://www.ufrgs.br/labsinal/NMA, in which an analysis of the nuclear area versus shape is performed. The nuclear shape is defined by the Nuclear Irregularity Index (NII), which is calculated by the following formula: NII = Asp − Arbx + Rr + Rou. Through this analysis, nuclei are classified according to the size and shape in the following populations: normal (N), small and regular (SR), small and irregular (SI), large and regular (LR), and large and irregular (LIr). SR nuclei typically correspond to apoptotic cells, while LR and LIr correspond to nuclei from senescent cells.

#### 2.1.9. Statistical Analysis

All experiments were performed at least three times independently. Statistical analysis consisted of *t* tests or ANOVA tests followed by the Tukey test. Analyses were performed using the SPSS 18.0 software. “*p*” values < 0.05 were considered significant.

### 2.2. In Silico

#### 2.2.1. Data Preprocessing and Network Design

Constitutive gene expression of MCF7, BT483, and MDA-MD-231 breast cancer cell lines was obtained by the rank product method. This method is based on calculating rank products (RP) from replicate experiments. We used sample replicates from the GEO databases (GSE63427, GSE98265, GSE73526, and GSE24717). For each sample, the average of the signal between the same probes was calculated and applied to the normalized microarray data using the limma package in the R/Bioconductor software [27]. The parameters used to run the RP were based on a significance value of ≤0.01. After this, the data were used to obtain a protein-protein interaction (PPI) network using the metasearch engine stringApp of the Cytoscape 3.8.0 platform [28, 29].

To obtain the PPI network of breast cancer, an automatic extraction of gene-disease associations was performed based on a DISEASES resource (http://diseases.jensenlab.org/), where text mining with manually curated disease-gene associations, cancer mutation data, and genome-wide association studies were considered [30]. All associations predicted by DISEASES are based on levels of confidence. In this study, we applied a maximal confidence cut‐off = 1 to predict the 1000 most significant nodes related to “malignant breast cancer.”

To predict the chemical-protein- (CP-) PPI network for MCF7, BT483, and MDA-MD-231 cell lines and breast cancer, the metasearch engine STITCH 5 (http://stitch.embl.de/) was used. It is important to note that stringApp and STITCH are Bayesian models based on similar degrees of confidence [31]. Here, a degree of confidence of 0.400 (medium) was used for both platforms to predict networks. Finally, to obtain a common CP-PPI network between MCF7 and breast cancer, we used the Cytoscape plugin NetworkAnalyzer [32], function “intersection.”

Input data to predict a PPI network representative of apoptosis (hsa04210), senescence (hsa04218), and autophagy (hsa04140) was based on a curated network map from KEGG [33].

For all predicted networks, GeneCards (http://www.genecards.org/) and PubChem (https://pubchem.ncbi.nlm.nih.gov/) databases were used to search for synonymous names of genes and compounds recognizable by all metasearch software used in this work.

#### 2.2.2. Centrality Analysis

Degree and betweenness centrality parameters were considered for analysis of CP-PPI networks using the Cytoscape plugin, CentiScaPe 2.2 [34]. Centrality degree indicates the number of adjacent nodes that are connected to a unique node. In this study, the average of this parameter was calculated as the sum of different node degree scores divided by the total number of nodes in the network. Another centrality parameter, betweenness was analyzed, which is defined as the number of the shortest paths between two nodes that pass through a targeted node [34, 35]. Similar to the degree average parameter, the betweenness average is defined as the ratio of the sum of different betweenness scores and the total number of nodes in the study. The mathematics of each parameter is detailed in the previous work of our group [36].

Nodes with high degree and high betweenness scores, when compared to the average for each parameter, are called hubs (H) and bottlenecks (B), respectively. In addition, quarters were defined based on the median of all H-B in the analysis. Quarters were used as cut-offs to discriminate those nodes with the highest values of degree and betweenness.

To compare centrality patterns among CP-PPI network, Venn diagrams were performed using an online Venn tool (http://bioinformatics.psb.ugent.be/webtools/Venn/).

#### 2.2.3. Functional Enrichment Analysis

Cytoscape ClueGO 2.5.7 plugin was utilized to perform KEGG and REACTOME enrichment network analysis based on updated annotation data from *homo sapiens* [37]. In this work, the enrichment was calculated with the hypergeometric test, using a significant FDR adjusted *p* value threshold of 0.0001. For processing data, Cytoscape version 3.8.0 was used.

#### 2.2.4. Gene Expression and Kaplan-Meier Survival Analysis of Hub-Bottlenecks Predicted Genes from Tumor Samples

To analyze the gene expression of H-B nodes from patients with breast cancer, we used the UALCAN web resource (http://ualcan.path.uab.edu). In this work, we compare transcriptome data from TCGA across 1907 tumors and 144 normal samples [38].

Subsequently, the KM plotter (http://kmplot.com//) of each H-B gene was performed. To this, expression data from 3.951 breast cancer patients was obtained from GEO, EGA, and TCGA databases [39]. The median expression level of each gene was used to divide patients into two groups (high and low), and overall survival analysis was performed to determine the association between the expression levels of H-B genes and the overall survival time of patients with breast cancer. The hazard ratio was provided, and the *p* value was calculated using logrank tests.

**Figure 1:**
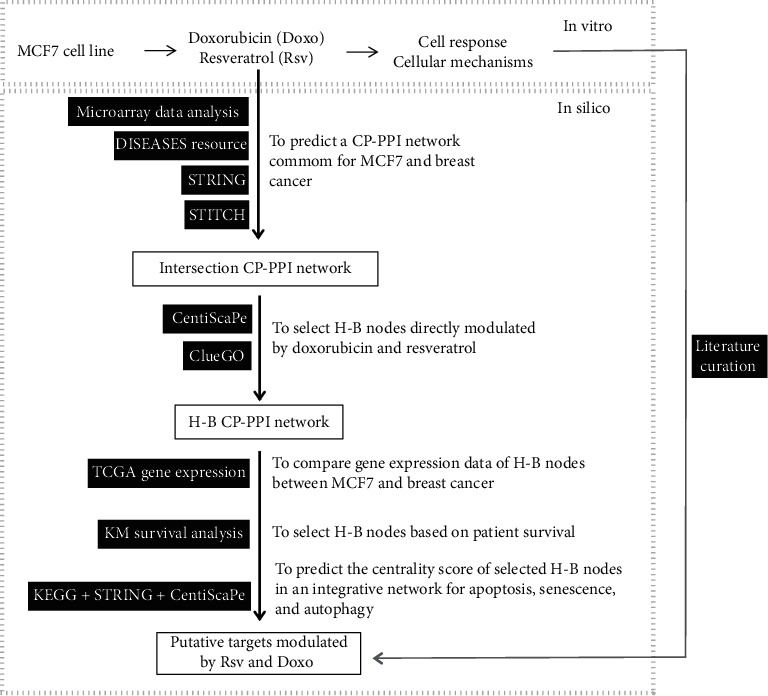
In silico strategy to identify putative genes/proteins modulated by Rsv and Doxo with therapeutic potential for breast cancer. This workflow shows the logical steps of the in silico analyses. On the top is shown an overview of the in vitro step, which involves a set of acute and long-term analyses after the treatment of MCF7 breast cancer cells with Rsv and Doxo (details are shown in Figure 2(a)). A translational strategy was performed to predict the putative genes and mechanisms modulated by Rsv and Doxo. To this, constitutive gene expression of MCF7 and metadata of breast cancer were used to predict interactomic networks. Topological analyses were prospected to predict hub-bottleneck (H-B) nodes and biological function associated. Gene expression, KM survival, and additional pathway analyses of these H-B nodes were also performed. Black boxes correspond to databases, algorithms, or metasearch software used.

An integrative workflow shows the logic strategy used in this study, which combines in vitro and in silico methods (Figure 1).

## 3. Results

### 3.1. Rsv Potentiates the Long-Term Toxicity of Acute Doxo Treatment

We first assessed the acute toxicity of Rsv and Doxo in MCF7 cells. For this analysis, we treated cells for 24 h with the following doses: Rsv 10, 30, 60, and 120 *μ*M; Doxo 100 and 200 nM; Doxo100+Rsv 10 *μ*M; and Doxo 100+Rsv 30 *μ*M. We then assessed cellular viability through the trypan blue exclusion assay (Supplementary Figure 1A). Analyzing the Combination Index (CI), we found a synergistic effect (CI 0,8) in the combination of Rsv 30 *μ*M and Doxo 100 nM. With these data in hand, we chose Rsv 30 *μ*M and Doxo 100 nM as doses for the following steps.

**Figure 2:**
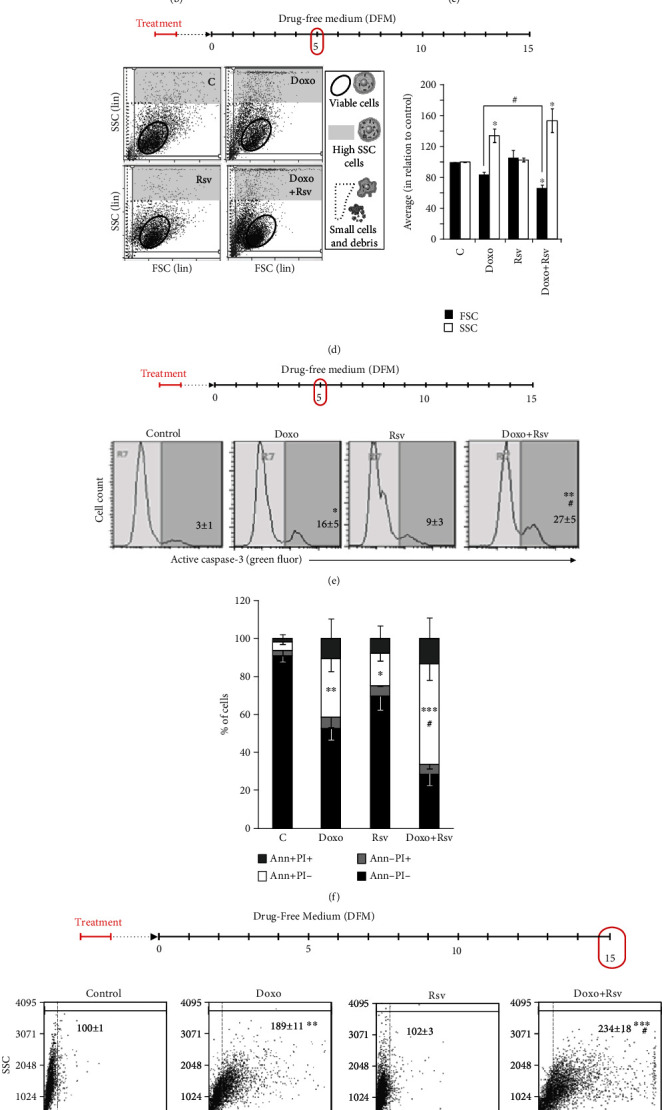
Rsv potentiated long-term apoptosis and senescence induced by acute Doxo treatment. (a) Experimental design. Cells were treated with resveratrol (Rsv) 30 *μ*M, doxorubicin (Doxo) 100 nM, or Rsv30+Doxo100 for 24 h. Dimethyl Sulfoxide (DMSO) not exceeding 0.05% was used as control. After this, cell viability was assessed. Then, cells were replated in a Drug-Free Medium (DFM) and grown for 15 days. During this period, several analyses were performed as indicated. CPD: Cumulative Population Doubling; FSC × SSC: forward scatter × side scatter; Annex/PI: Annexin V-FITC/Propidium Iodide staining; Casp-3: active caspase-3-positive cells; (b) cell viability after 24 h, measured through the trypan blue exclusion assay. (c) Cumulative Population Doubling along the 15 days. (d) Cell morphology measured at day 5. FSC × SSC plots (left) and averaged FSC and SSC (right, bar graph). (e) Active caspase-3-positive cells measured at day 5. Representative flow cytometry plots are shown. Numbers represent the percentage of positive cells (average ± standard deviation). (f) Annexin V-FITC/Propidium Iodide staining measured at day 5. (g) C12-FDG staining measured at day 15. Representative plots are shown. Numbers represent the average of green fluorescence (average ± standard deviation). (h) Nuclear morphometric analysis (NMA) measured at day 5. Representative images are shown on top (magnification: 200x); pie charts represent the percentage of nuclei in each population. Details are shown in Supplementary Figure 1C; ^∗^*p* < 0.05, ^∗∗^*p* < 0.01, and ^∗∗∗^*p* < 0.001 in relation to control; ^#^*p* < 0.05, ^##^*p* < 0.01, and ^###^*p* < 0.001 in relation to Doxo.

An overview of the experimental design is shown in Figure 2(a). We treated MCF7 breast cancer cells with either Rsv 30 *μ*M, Doxo 100 nM, or the cotreatment containing both. DMSO not exceeding 0.05% was used as a control. After 24 h, we determined cell viability and replated cells in a Drug-Free Medium (DFM) for 15 days. During these 15 days, cell numbers were quantified and CPD was calculated. At day 5, we assessed the levels of apoptosis and autophagy. To Doxo, we also assessed acridine orange staining at days 10 and 15, and after 15 days, we measured cell senescence. Autophagy was modulated in specific time points, as depicted below.

We did not observe any acute additive toxicity of Rsv to Doxo after 24 h of treatment (Figure 2(b)). Considering the long-term growth of MCF7-treated cells, Rsv alone did not exert any lasting toxicity effects, while Doxo-treated cells showed a stationary state until day 5 after the treatment. From day 5 onward, regrowth in the population of Doxo-treated cells had a rate similar to control. On the other hand, Rsv did in fact potentiate the long-term toxicity of Doxo (Figure 2(c)). In conclusion, these data suggest that acute treatment with clinically relevant doses of Rsv and Doxo is capable of affecting the long-term growth of breast cancer cells.

### 3.2. Rsv Potentiates Doxo-Induced Apoptosis and Senescence in Breast Cancer Cells

Next, we began to probe the mechanisms underlying the additive effect of Rsv on Doxo. We initially assessed cell size and intracellular granularity through flow cytometry. The forward scatter (FSC, i.e., cell size) versus side scatter (SSC, i.e., intracellular granularity) graph is a straightforward, objective source of information to infer the fate of subpopulations of cells. Particularly for examining responses to therapy, FSC/SSC data are quite valuable since cancer cell populations are molecularly heterogeneous and, thus, may respond through various cellular outcomes [23]. Through this analysis, we observed that treatment with Rsv increased the number of Doxo-induced shrunken cells (Figure 2(d)), a typical morphologic feature of apoptosis. Corroborating this, we also found that Rsv led to a reduction in average cell size (FSC), an effect which can be observed in the shift of the population of “viable” cells to the left in Figure 2(d). Doxo also increased the number of cells with high intracellular granularity (SSC) (Figure 2(d), gray area), and this effect was also potentiated by Rsv. These data suggest that Doxo and Doxo+Rsv triggered morphologic alterations that resemble to apoptosis in a given subpopulation of cells, while also promoting the increase in intracellular granularity (which is found in cellular mechanisms such as autophagy).

We then examined these mechanisms through specific assays. Regarding apoptosis, we observed an increase in both the percentage of cells with active caspase-3 (Figure 2(e)) and annexin-positive cells (Figure 2(f)) after Doxo treatment and found that this increase was potentiated by Rsv to both markers (Figures 2(e) and 2(f)). This result corroborates the FSC data and likely also underlies (at least partially) the reduction in CPD observed from the Doxo+Rsv cotreatment, compared to Doxo alone.

Clinically, patients are usually treated for 24 h with Doxo and then recover for 2 weeks or more. Thus, evaluating the long-term senescence after acute Doxo treatment is clinically relevant. Here, we measured the activity of senescence-associated beta-galactosidase (SA-*β*-gal) by measuring C12-FDG cleavage (Figure 2(g)) and the cleavage of chromogenic SA-*β*-gal substrate (Supplementary Figure 1B). We found that 24 h of Doxo treatment increased the activity of SA-*β*-gal in MCF7 cells after 15 days. While Rsv alone did not increase these markers, it did potentiate Doxo-induced senescence. We then assessed nuclear morphometry also 15 days after the treatment, since nuclear enlargement is a typical alteration of senescent cells. We found an increase in nuclear size after Doxo treatment, which was potentiated by Rsv in the cotreatment (Figure 2(h), top, DAPI images; Supplementary Figure 1C). Through the NMA technique, we observed an increase in the percentage of large and regular nuclei (Figure 2(h), bottom, pie charts; Supplementary Figure 1C), which suggests senescence entering, after Doxo treatment. Also, we found an increase in irregular, mainly elliptic nuclei. However, it is important to note that near to 35% of nuclei from Doxo-treated cells appeared with normal morphometry, which could represent resistant cells. Rsv potentiated Doxo-induced nuclear enlargement, since the cotreatment increased the percentage of large and regular nuclei while reducing the percentage of normal nuclei. Rsv+Doxo also led to a reduction in irregular nuclei (Figure 2(h), bottom, pie charts; Supplementary Figure 1C).

Altogether, these results show that Rsv potentiated the long-term effect of acute Doxo in MCF7 breast cancer cells. It is important to highlight that this addictive effect was not evident immediately after the end of treatment. In addition, the effect may be due to the long-term increase of apoptosis and senescence by Rsv in Doxo-treated breast cancer cells.

### 3.3. Rational Inhibition of Autophagy Induced by Doxo+Rsv Sensitizes Breast Cancer Cells

**Figure 3:**
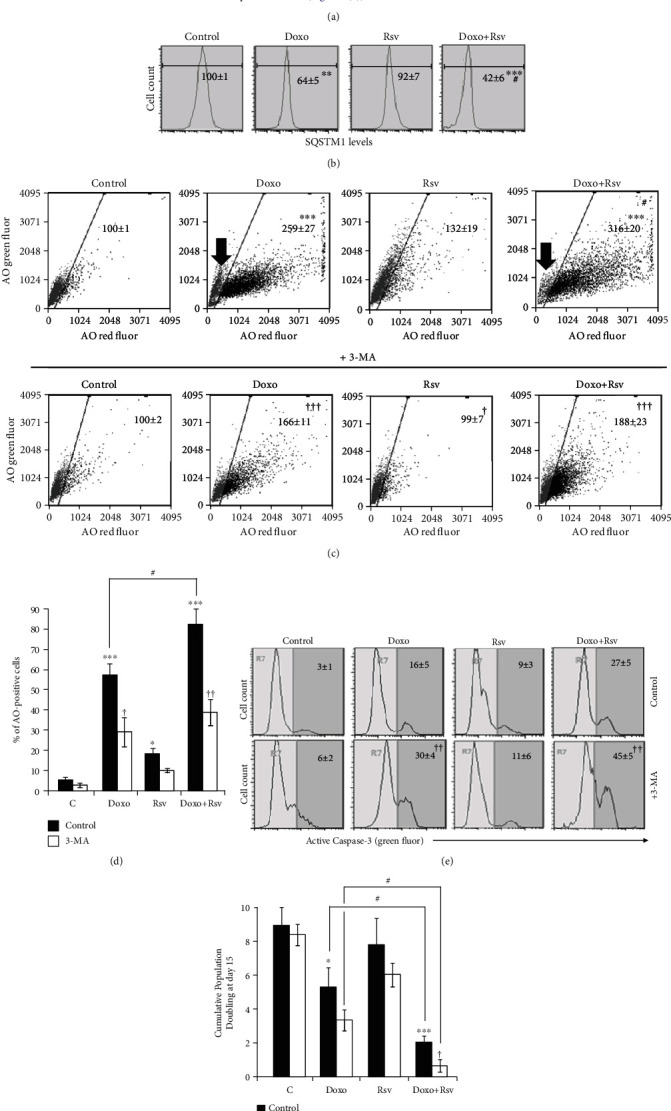
Rsv potentiated cytoprotective autophagy induced by Doxo in MCF7 cells. (a) Experimental design. Cells were treated with Rsv 30 *μ*M, Doxo 100 nM, or Rsv30+Doxo100 for 24 h. Dimethyl Sulfoxide (DMSO) not exceeding 0.05% was used as control. After this, cell viability was assessed. Then, cells were replated in a Drug-Free Medium and grown for 15 days. Cells were treated with 2 mM of 3-methyladenine (3-MA) for 1 h at days 3 and 4. (b) SQSTM1 levels measured by immunocytochemistry at day 5 (average ± standard deviation). (c) Acridine orange (AO) staining. Numbers represent the intensity of AO red fluorescence in relation to control, considered as 100 (average ± standard deviation). Black arrow points to the AO-negative population of cells in Doxo and Doxo+Rsv treatment. (d) Percentage of AO-positive cells. (e) Active caspase-3-positive cells measured by flow cytometry. Numbers correspond to the percentage of positive cells (average ± standard deviation). (f) Cumulative Population Doubling measured at day 15. Abbreviations: AO: acridine orange; SQSTM1: sequestosome 1; 3-MA: 3-methyladenine; Casp-3: active caspase-3-positive cells; CPD: Cumulative Population Doubling. ^∗^*p* < 0.05, ^∗∗^*p* < 0.01, and ^∗∗∗^*p* < 0.001 in relation to control; ^#^*p* < 0.05, ^##^*p* < 0.01, and ^###^*p* < 0.001 in relation to Doxo; ^†^*p* < 0.05, ^††^*p* < 0.01, and ^†††^*p* < 0.001, comparing 3-MA to control using PBS.

After treatment with chemotherapy, cancer cells usually activate a set of mechanisms involved in the stress response in order to adapt and survive. Among these mechanisms is autophagy, which is involved in the resistance of cancer cells to death [40, 41]. Here, we assessed autophagy induction by Rsv, Doxo, and cotreatment as shown in Figure 3(a) and Supplementary Figure 2B. We also tested the role of autophagy by treating cells with 3-methyladenine 2 mM for 1 h before treatments or after cells replating in DFM (Figure 3(a)). We found that Doxo-treated cells reduced the levels of autophagy adapter SQSTM1, suggesting increased autophagic flux (Figure 3(b)). Doxo also induced a long-lasting increase in both the intensity of acridine orange (AO) red staining (Figure 3(c), top) and the percentage of AO-positive cells (Figure 3(d)). Important to mention, after the 24 h of treatment, despite the percentage of AO-positive cells increased, the increase in the intensity of AO staining was still not significant, suggesting that autophagy is, in fact, increased only after drug withdrawal (Supplementary Figure 2A). Meanwhile, treatment with Rsv alone did not alter SQSTM1 levels (Figure 3(b)) and slightly increased the intensity and the percentage of AO-positive cells (Figures 3(a) and 3(b)). When combined with Doxo, Rsv increased the intensity of AO red fluorescence (Figure 3(c), top) and the percentage of AO-positive cells (Figure 3(d)), while reducing the levels of SQSTM1 (Figure 3(b)). Importantly, adding Rsv to Doxo led to a strong reduction in a subpopulation of nonautophagic cells, which could be intrinsically insensitive to Doxo (Figure 3(c), top, black arrows).

Data from autophagy analysis suggest that most cells that resisted to Doxo+Rsv have high levels of autophagy. Since autophagy acts as a cytoprotective mechanism in breast cancer cells [40], we then assessed the consequence of its inhibition in apoptosis and long-term cell growth. To this, we next inhibited autophagy with 3-methyladenine (3-MA) for 1 h just before the treatments (Supplementary Figure 2B, left scheme). However, this strategy did not alter the toxicity of Rsv, Doxo, or cotreatment in the long-term (Supplementary Figure 2B, right graph). We then decided to treat cells at days 3 and 4 (Figure 3(a)), since day 5 corresponded to the peak of Doxo-induced autophagy (Supplementary Figure 2A). The confirmation of autophagy inhibition is shown in Figures 3(c) and 3(d). As shown in Figure 3(e), adding 3-MA at days 3 and 4 for 1 h each led to an increase in the percentage of cells with active caspase-3 in response to Doxo and, to a greater extent, to Doxo+Rsv. Consequently, we found a reduction in final CPD at day 15 after 3-MA treatment to both Doxo and Doxo+Rsv (Figure 3(f)). Indeed, adding 3-MA to Doxo+Rsv was the more effective treatment. This suggests that the inhibition of autophagy during its activation is the most effective treatment to sensitize breast cancer cells to Doxo and Doxo+Rsv cotreatment.

### 3.4. A CP-PPI Network Composed of 24 Nodes and Edges Is a Robust Modeling to Predict Molecular Targets Modulated by Doxo and/or Rsv

**Figure 4:**
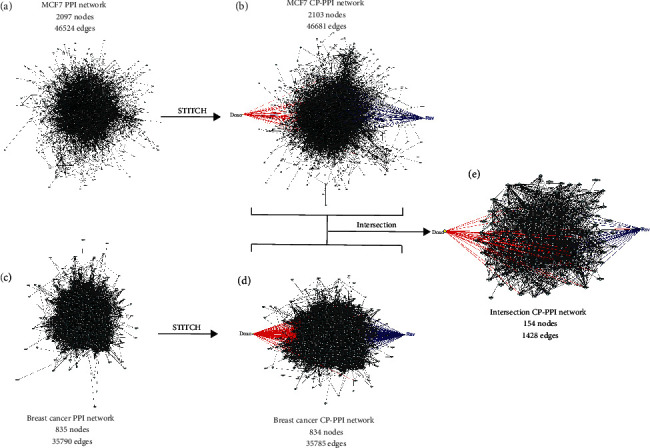
Strategy to predict a translational chemical-protein (CP) and protein-protein interacting (PPI) networks between MCF7 cell line and breast cancer. (a) MCF7 PPI network: based on the constitutive gene expression of MCF7, a PPI network was predicted using the STRING software. (b) MCF7 CP-PPI network: targets of Rsv and Doxo were predicted using the STITCH platform. (c) Breast cancer PPI network: the 1000 most significant genes associated with the term “malignant breast cancer” from DISEASES resource were used to perform a PPI network based on interacting data available from stringApp. (d) Breast cancer CP-PPI network: to predict targets of Rsv and Doxo, interactions were prospected using the STITCH platform. (e) Intersection CP-PPI network: an “intersection” operation, available in the NetworkAnalyzer, was performed to obtain common nodes and edges between the MCF7 CP-PPI network and the breast cancer CP-PPI network.

To expand upon our in vitro findings and provide molecular insights regarding the cellular mechanisms involved in the response of breast cancer cells to Rsv+Doxo, we developed a translational in silico strategy based on interactomic data available for MCF7 and breast cancer. A total of 3.502 genes constitutively expressed in the MCF7 cell line (Supplementary Table 1) were used as the input for STRING software (see details in Material and Methods). As a result, a PPI network called MCF7, composed of 2097 nodes and 46524 edges, was obtained (Figure 4(a)). Following this analysis, potential targets of Rsv and Doxo were predicted using the STITCH platform. In this context, a new network named *MCF7 CP-PPI* composed of 2103 nodes, including Rsv and Doxo, and 46681 edges was prospected (Figure 4(b)). Complementarily, interactomic data from DISEASES resource were used to extract gene-disease associations to predict a network for breast cancer (for details, see Material and Methods). This network, denoted as the *breast cancer PPI network*, was composed of 835 nodes and 35790 edges (Figure 4(c)). To predict the targets of Rsv and Doxo in this network, STITCH was also applied. A new network entitled *breast cancer CP-PPI network* was obtained (Figure 4(d)), which revealed 834 nodes including Rsv and Doxo and 35785 edges. To obtain a common network for the MCF7 and breast cancer CP-PPI networks, a merger based on intersection was performed. The network remaining after this operation possessed 154 nodes and 1428 edges and was designed as the *intersection CP-PPI network* (Figure 4(e)). Functional analysis of this network was also performed using the ClueGo plugin (Supplementary Figure 3 A, B, and C). In this analysis, four discrete pathways, as overexpressed terms, were identified (Supplementary Figure 3C). The predicted terms were ESR-mediated signaling (73.3%), viral carcinogenesis (13.3%), HIF-1 signaling pathway (6.7%), and PI3K-Akt signaling pathway (6.7%).

**Figure 5:**
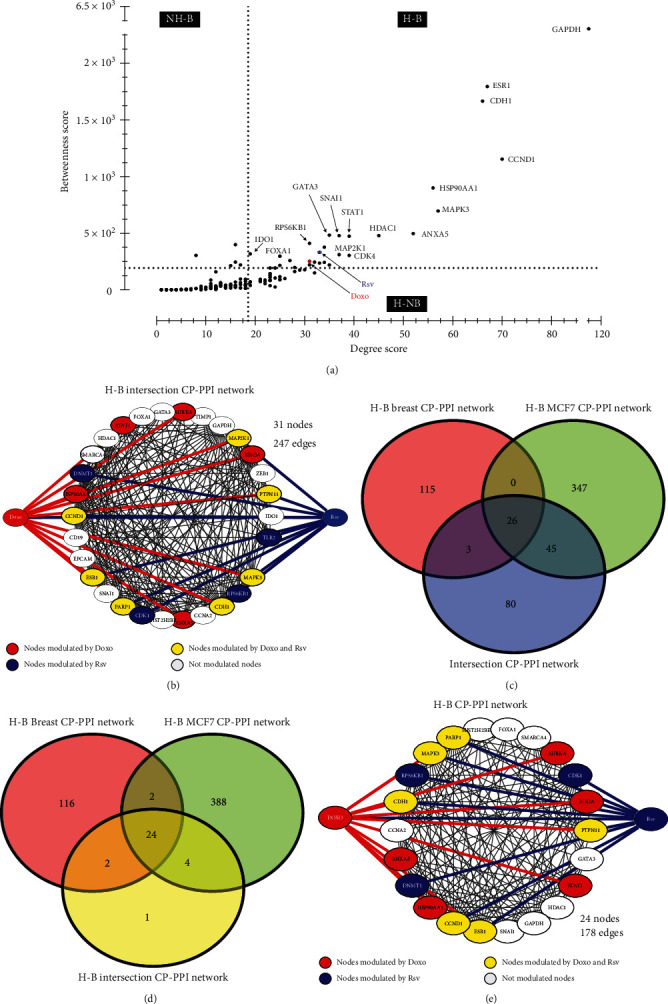
A network core composed of 24 hub-bottleneck (H-B) nodes is common between the MCF7 CP-PPI network, breast cancer CP-PPI network, and intersection CP-PPI network. (a) Centrality analysis of the intersection CP-PPI network. Dashed lines represent the thresholds calculated for each centrality, degree, and betweenness. Proteins/genes are represented by black dots, while compounds are marked in blue (Rsv) and red (Doxo). (b) H-B intersection network: all H-B nodes from the intersection CP-PPI network were selected to compose this graph. Nodes highlighted in red and blue are modulated by Doxo and Rsv, respectively; yellow nodes represent genes modulated by both compounds. Network connectivity of Rsv and Doxo nodes is also highlighted in blue and red, respectively. (c) Venn diagram between the H-B MCF7 CPPI network, H-B Breast CP-PPI network, and intersection CP-PPI network. (d) Venn diagram between the H-B MCF7 CP-PPI network, H-B Breast CP-PPI network, and H-B intersection CP-PPI network. (e) H-B CP-PPI network: network composed of 24 H-B nodes common between all H-B CP-PPI networks explored. H-B: hub-bottleneck; NH-B: non-hub-bottleneck; H-NB: hub-nonbottleneck. Color legends are similar to (b).

In addition, network topological features can also predict potential targets and mechanisms of action modulated by Rsv and Doxo. Furthermore, these properties can also provide measures of representativeness of the *intersection CP-PPI network* into the *MCF7 CP-PPI network* and *breast cancer CP-PPI network* based on structural attributes. To do this, the best ranking of compound-target (high impact on the network) was calculated based on network connectivity analysis. Accordingly, degree and betweenness parameters were calculated for the intersection network; 31 H-B nodes were identified, including Rsv and Doxo (Figure 5(a), Supplementary Table 2). Through this, we found that Rsv and Doxo, by itself, are nodes highly connected, which control high information flow in the network.

Subsequently, these H-B nodes were used to construct a new network, named the *H-B intersection CP-PPI network*, with 31 nodes and 247 edges (Figure 5(b)). Subsequently, the representativeness of all nodes comprising the *intersection CP-PPI network*, such as their H-B nodes, were evaluated into the *MCF7 CP-PPI network* and *breast cancer CP-PPI network*, respectively. To accomplish this, it was necessary to quantify whether common H-B nodes were included when the intersection operation was performed (Figure 4(e)). H-B nodes were also predicted for the *MCF7 CP-PPI network and breast cancer CP-PPI networks*, and new networks were prospected based on centrality analysis (see Supplementary Figure 4). These networks were named the *H-B breast cancer CP-PPI network* and the *H-B MCF7 CP-PPI network* composed of 144 nodes and 418 nodes, respectively. Comparing networks, only 26 nodes, including Rsv and Doxo, were common to the *Intersection CP-PPI network*, *H-B MCF7 CP-PPI network*, and *H-B Breast cancer CP-PPI network* (Figure 5(c)). Notwithstanding, 24 of these 26 nodes are shared by all H-B CP-PPI networks (Figure 5(d)). According to this, the H-B intersection CP-PPI network is majoritarian constituted by common H-B (24/31). It is important to note that only these 24 H-B connected by 178 edges were considered for posterior analysis (Figure 5(e)). Interestingly, 18 of these 24 H-B appeared in overexpressed terms, after functional analysis, as detailed below (see Supplementary Figure 3C).

### 3.5. Hub-Bottlenecks Directly Modulated by Doxo and/or Rsv Suggest That Seven Putative Genes Have a Central Role in the Response of Breast Cancer Cotreatment

Of these 24 interconnected H-Bs obtained from previous network strategy, 5 H-Bs (ANXA5, AURKA, HSP90AA1, STAT1, and RHOA) are directly modulated by Doxo, 1 H-B (CDK4) is directly modulated by Rsv, and 8 H-Bs (CCND1, CDH1, DNMT1, ESR1, MAPK3, PARP1, PTPN11, and RPS6KB1) are directly modulated by the combination of these compounds. It is important to note that the term “modulation” refers to drug-target interaction at the network level.

**Figure 6:**
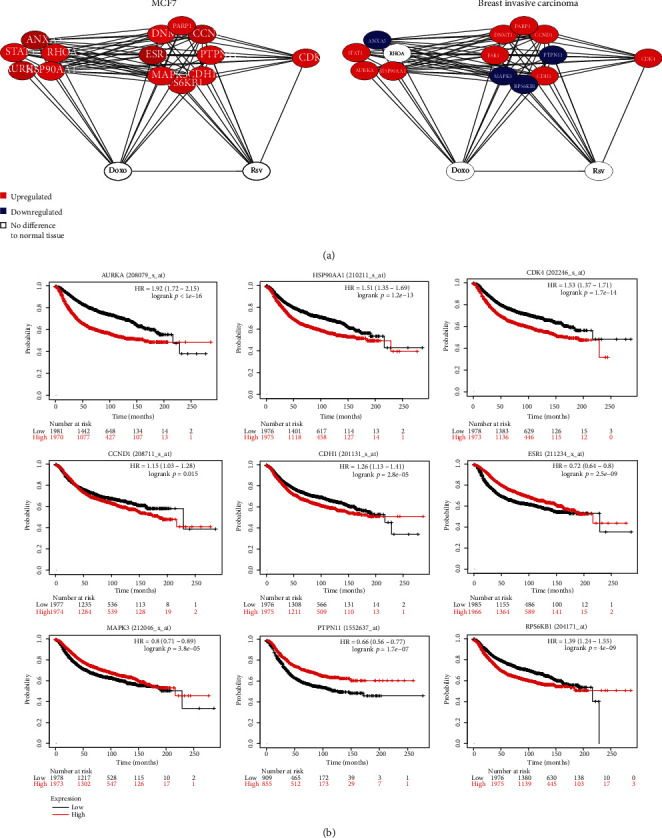
Fourteen H-B genes are directly modulated by Rsv, Doxo, or the combination, and nine out of these genes affect the survival of breast cancer patients. (a) Networks show only nodes directly modulated by Rsv or Doxo in MCF7 and breast cancer. Overlapping gene expression data with each network was performed. Red and blue nodes represent genes that are up- or downregulated, respectively, in cancer in comparison to normal mammary tissue. (b) Kaplan-Meier survival curves of nine H-B genes in breast cancer patients.

Subsequently, genes differentially expressed between MCF7 and breast cancer in relation to normal mammary tissue were highlighted, see details about gene expression data in Material and Methods. Nodes that were shown to be upregulated and downregulated in cancer are in red and blue, respectively; nodes without differential expression are in white (Figure 6(a)). All the nodes modulated by Doxo and/or Rsv appeared as upregulated in the MCF7 cell line based on microarray analysis (Supplementary Table 1). In breast cancer, according to the transcriptome data from TCGA, nine out of them appeared as being upregulated while four of them are downregulated. Subsequently, Kaplan-Meier survival analysis of each H-B gene was performed. To do this, expression data from 3.951 breast cancer patients were analyzed. Only nine H-Bs (AURKA, HSP90AA1, CDK4, CCND1, CDH1, ESR1, MAPK3, PTPN11, and RPS6KB1) showed a significant association between expression levels and the overall survival time of patients with breast cancer (Figure 6(b)).

**Figure 7:**
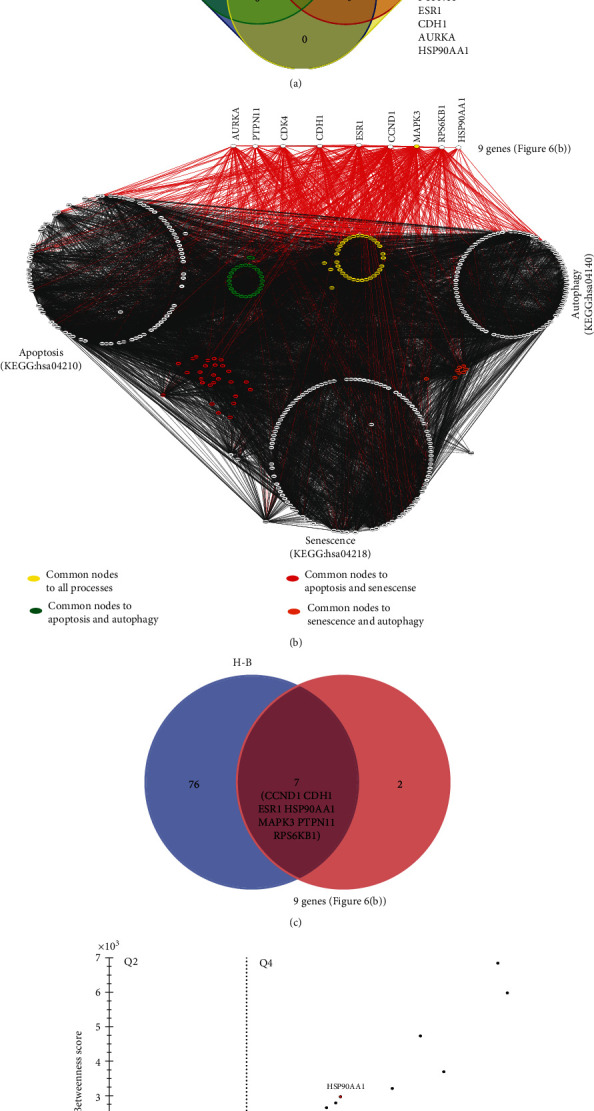
Seven H-B genes interplay between apoptosis, senescence, and autophagy. (a) Venn diagram between the nine predicted genes and KEGG pathways of apoptosis, senescence, and autophagy. (b) Apoptosis, senescence, and autophagy network. Network connectivity of these nine genes is highlighted in red; common nodes between molecular processes are also highlighted. (c) Venn diagram between nine predicted genes and H-B of apoptosis, senescence, and autophagy network. (d) Centrality analysis of apoptosis, senescence, and autophagy network; only H-Bs were considered. Dashed lines represent thresholds based on medians (degree and betweenness) used to define the quarters. Fourth-quarter (Q4) corresponds to H-Bs with higher degree and betweenness values from the network.

To predict the involvement of these nine genes with apoptosis, senescence, and autophagy, data of curated pathways were extracted from the KEGG database (see Material and Methods). Initially, a Venn diagram was constructed, including all genes/proteins associated with each process and the nine predicted genes (Figure 7(a)). MAPK3 was common for all cellular processes; CCDN1 and CDK4 were shared with senescence and RPS6KB1 with the autophagic process. Next, the involvement of these nine genes was analyzed at the network level. All proteins registered by KEGG for each cellular process together with the nine predicted genes were integrated into a unique PPI network, using STRING. A strongly connected network composed of 350 nodes and 8924 edges was prospected (Figure 7(b)). Subsequently, centrality analysis was performed to predict H-B, and 83 H-Bs were detected. Interestingly, seven (CCND1, CDH1, ESR1, HSP90AA1, MAPK3, PTPN11, and RPS6KB1) out of nine initial predicted genes/proteins are H-B (Figure 7(c)). To determine if these H-Bs have high degree and betweenness values in the network, an additional analysis was performed. Based on the median of degree ($\overset{\sim }{x}=772.3$) and betweenness ($\overset{\sim }{x}=96$), four quarters were defined (Figure 7(d)). In the fourth quarter, 5 H-Bs (CDH1, ESR1, HSP90AA1, MAPK3, and RPS6KB1) showed high connectivity. These top 5 H-Bs were analyzed in the other two breast cancer cell lines (BT483 and MDA-MD-231), which are ESR1-negative (Supplementary Figure 5). Interestingly, CDH1 and RPS6KB1 are common H-Bs in all CP-PPI networks. Since centrality is a key property of complex networks that influences the dynamics of processes, any pharmacological modulation on H-B can induce pathway-network perturbations.

## 4. Discussion

The frequent resistance of breast cancer cells to Doxo, along with its cardiotoxicity limits its utility as a treatment in breast cancer therapy. Adjuvant drugs, the redesign of clinical regimens, or the modulation of cellular mechanisms could sensitize resistant cells. Translationally, it is of fundamental importance to study the response of cancer cells over the long term. In the current study, using a clinically relevant protocol, here, we found that 24 h of treatment with Rsv was sufficient to potentiate the effects of Doxo for 15 days via the induction of apoptosis and senescence. Autophagy was detected in high levels in cells that survived at least 5 days to Doxo and Doxo+Rsv, and the rational inhibition of this mechanism triggered apoptosis and increased the long-term toxicity of cotreatment. This is in agreement with recent evidences suggesting autophagy inhibition as a strategy to overcome the resistance of breast cancer, including resistance to Doxo [42, 43]. Indeed, seven clinical trials combining autophagy inhibitors with endocrine or cytotoxic therapies for breast cancer treatment are currently being conducted. Our in silico analysis identified seven putative genes potentially involved in the additive effects of Rsv to Doxo. In support of this, a literature search revealed that Rsv is in fact capable of modulating these genes, which may underlie its additive effects with Doxo on breast cancer cells.

Despite being, to our knowledge, the first study that assessed the long-term effects of acute treatment with Rsv+Doxo, previous studies have tested the acute effects of Rsv as an adjuvant therapy in breast cancer treatment, which is of great therapeutic interest because Rsv attenuates Doxo-induced cardiotoxicity [20, 44] and it is not toxic to normal cells [45, 46]. In MCF7 cells, it has been shown that Rsv triggers G1/S cycle arrest and apoptosis only after 48 h to 72 h of treatment, or after 24 h of treatment with at least twice the dose used here. This effect occurs via the reduction of CDKs and/or cyclin D1 levels, accompanied by the suppression of prosurvival pathways, like Bcl-2 and NF-*κ*B [47–50]. Indeed, Venkatadri et al. found an IC50 of 162 *μ*M to 24 h of Rsv in MCF7 cells [49]. Rsv was also found to reduce the migration, invasion, and stemness of MCF7 cells [51]. Our study also found that Doxo-induced autophagy was potentiated by Rsv, which, in MCF7 cells, play its proautophagic effect by the direct inhibition of mTOR [52]. This, in turn, may also lead to the suppression of S6K1, a direct target of mTOR and one of the genes suggested to be modulated by Rsv in our in silico analysis. Rsv has also been described as modulating key pathways involved in breast cancer progression, such as MAPK, PI3K-AKT, cell cycle checkpoints, and ER signaling [53]. Here, we observed that Rsv was not effective as a treatment on its own in controlling the long-term growth of MCF7 cells, but that similar doses potentiated Doxo toxicity. Thus, understanding the molecular mechanisms underlying this additive effect is critical for further progress in overcoming Doxo resistance. Here, we present a list of seven putative genes that are modulated by Rsv in breast cancer in the context of cotreatment with Doxo: HSP90AA1, CCND1, CDH1, ESR1, MAPK3, PTPN11, and RPS6KB1. We found experimental evidence in support of Rsv modulating the expression of CCDN1/cyclin D1, CDH1/E-cadherin, ESR1/ER-*α*, and MAPK3/ERK1 in MCF7 cells. Rsv also modulates PTPN11/SHP2 and RPS6KB1/S6K1 in other cancer cells, while the modulation of HSP90AA1 by Rsv has not been assessed in cancer cells (Supp. Table 3).

Of these genes and proteins, three are classically upregulated in breast cancer, and these are associated with poor prognosis in breast cancer: CCDN1/cyclin D1 [54], PTPN11/SHP2 [55], and RPS6KB1/S6K1 [56]. Rsv acts as a negative regulator of these pathways in breast cancer and other cancer types. On the other hand, high levels of CDH1/E-cadherin [57] and MAPK3/ERK1 [58] have been associated with favorable prognoses in breast cancer, and Rsv increases their levels and/or activity in MCF7 cells. Importantly, modulation of these genes may attenuate several mechanisms associated with Doxo resistance in MCF7 including (a) increased drug efflux [59], (b) cell death resistance (through alterations in ER-*α* and/or NF-*κ*B pathways) [60, 61], (c) epithelial-to-mesenchymal transition [62], (d) the enrichment of cancer stem cell-like phenotype [60, 63], and (e) autophagy [43]. Indeed, here, we observed the regrowth of Doxo-treated MCF7 cells from day 5 after treatment onward, and we found that Rsv strongly attenuated this regrowth. This could be achieved through the modulation of those genes obtained in our in silico analysis, as detailed in the following paragraphs.

In MCF7 cells, achieving resistance to Doxo by increasing drug efflux involves the upregulation of MDR1 [64]. It has been demonstrated that Rsv can revert multidrug resistance in breast cancer cells, leading to the intracellular accumulation of Doxo and increased toxicity [12]. A similar result was found by Zhao et al. [46] using nanocapsules containing Rsv and Doxo. Molecularly, such an increase of MDR1 may be mediated by ER-*α*, the product of the ESR1 gene [64, 65]. ER-*α* is directly involved in breast carcinogenesis and is associated with unfavorable prognoses as well as Doxo resistance in breast cancer [66]. Rsv is considered a phytoestrogen due to its ability to compete with 17*β*-estradiol (E2) for binding to and modulating the activity of ER-*α* [67], and our in silico analysis suggested ER-*α* as a potential target of Rsv. Experimentally, the inhibition or downregulation of ER-*α* by Rsv has already been demonstrated in MCF7 cells [68]. Downstream of ER-*α*, the PI3K/Akt signaling pathway is activated, which drives cell survival and proliferation in breast cancer [69]. The upregulation of this pathway is also known to be in Doxo resistance [70]. Thus, PI3K/AKT inhibition, as triggered by Rsv in breast cancer [71], may sensitize cancer cells to Doxo [72]. A key effector of PI3K/Akt, S6K1, also activates ER*α* and promotes the proliferation and invasiveness of ER-positive breast cancer cells [73, 74]. The endocrine resistance of breast cancer cells can be achieved through direct phosphorylation of ER-*α* by S6K1, leading to ligand-independent activation of ER-*α*. This, in turn, upregulates S6K1 expression, leading to a positive regulatory loop that maintains cell proliferation [73, 75]. Our in silico analysis suggested S6K1 as another potential target modulated by Rsv. Experimentally, it is known that Rsv strongly suppressed the activity of S6K1 in MCF7 cells [76], which may interrupt the abovementioned loop and sensitize breast cancer cells. This result is even more relevant in this model since MCF7 cells have high levels of S6K1 expression and activity [73].

In addition to the ER-*α*/PI3k/Akt/S6K1 pathway, our in silico analysis suggested that Rsv may suppress other prosurvival pathways to sensitize MCF7 cells to Doxo-induced apoptosis or senescence [77]. One of the classic targets of Rsv in cancer cells is the cyclin D1/CDK4 complex. High levels of cyclin D1, for example, have been associated with increased mortality in breast cancer [78], and apoptosis resistance to tamoxifen [79] and Doxo [80]. Several studies have shown that Rsv reduces expression levels of CCDN1/cyclin D1 along with the activity of cyclin D1/CDK4, leading to G1/S-phase cell cycle arrest in MCF7 cells [51, 81, 82]. Cyclin D1 also modulates the response to chemotherapy in these cells, including Doxo [83, 84]. Another pathway potentially involved in our model is NF-*κ*B. NF-*κ*B overactivation may also be directly involved in Doxo resistance in MCF7 cells [85], as NF-*κ*B inhibition sensitized MCF7-resistant cells to Doxo [60]. Rsv is also capable of inhibiting NF-*κ*B in these cells [48]. Indeed, Rsv could potentially overcome Doxo resistance by inducing apoptosis through downregulating the expression of NF-*κ*B and BCL-2 [13]. This mechanism may involve the increase of E-cadherin levels by Rsv, since E-cadherin intracellular signaling attenuates NF-*κ*B signaling [86]. Our in silico analysis also indicates SHP2 as a target of Rsv. SHP2 is an oncoprotein that favors tumor growth, cell invasion, and resistance to apoptosis in MCF7 cells, as has been shown both in vitro and in vivo [87]. Inhibition of SHP2 was also found to reactivate senescence in breast cancer in mice [88]. Finally, our in silico analysis suggested ERK1 as a putative target of Rsv. In MCF7 cells, ERK1 inhibits cell proliferation via the downregulation of YAP1, a transcriptional coactivator involved in breast carcinogenesis [58]. Indeed, in breast cancer patients, high levels of ERK1 are associated with good prognoses, positive responses to therapy, and controlled cancer progression [58, 89]. Rsv has been previously described as increasing ERK1 in MCF7 cells [90, 91].

The epithelial-to-mesenchymal transition (EMT) also plays a role in the resistance of breast cancer to Doxo. This mechanism involves the reduction of epithelial markers, especially E-cadherin, in parallel with an increase in mesenchymal markers. In breast cancer, suppression of SHP2 was found to lead to an increase of E-cadherin, reversing the EMT. Here, we observed an increase in nuclei with elliptic, elongated shape 15 days after Doxo treatment, which is a typical alteration of EMT [92]. Adding Rsv to Doxo reduced this nuclear population. At a molecular level, our in silico analysis suggested that Rsv can modulate two key players of the EMT in breast cancer, Chd1/E-cadherin, and PTPN11/SHP2 [93]. Indeed, treatment with Rsv has been shown to reverse the EMT in breast cancer, sensitizing cells to Doxo [11, 90, 94]. The loss of E-cadherin can lead to disease progression, metastasis, apoptosis, and drug resistance in breast cancer [95–97]. Likewise, CDH1 promoter methylation correlates with decreased gene expression and poor prognosis in patients [98]. CDH1 drives proper cell cycle progression, and its depletion accelerates breast cancer cell proliferation and cooperates with PTEN loss to promote breast cancer progression in rodents [99]. On the other hand, the activation or increase of E-cadherin can sensitize breast cancer cells and also suppress cancer progression [96, 100]. A reduction in E-cadherin triggered by miR-106b~25 also promoted a bypass of Doxo-induced senescence and increased cell motility and invasion [101]. Rsv increases E-cadherin and reduces EMT in MCF7 cells [102]. Epigenetic mechanisms are also involved in EMT-mediated resistance to Doxo. miR-25 targets EP300, a transcriptional activator of E-cadherin, resulting in EMT with increased cell motility and Doxo resistance [101]. Rsv downregulates miR-25 in cancer cells, which restores E-cadherin levels and may sensitize cells to Doxo [103]. Complementarily, Rsv decreased other genes associated with the EMT, such MMP9 and MMP2, which are involved in the aggressiveness and invasiveness of Doxo-resistant breast cancer [94]. Another gene potentially modulated by Rsv in our model is PTPN11. Its upregulation in breast cancer is associated with EMT, cell motility, and high tumor grade [104]. Inhibition of the PTPN11-encoded protein SHP2 also led to EMT associated with the upregulation of E-cadherin and downregulation of mesenchymal markers [93]. Likewise, SHP2 depletion or knockdown has been shown to prevent invasion and metastasis in vivo [105]. With this in mind, SHP2 inhibitors may help in breast cancer management [106], as has already been demonstrated in animal models [88].

Finally, cancer stem cells (CSC) are also important for resistance to Doxo in breast cancer. Rsv has been shown to reduce breast CSCs in MCF7 cultures [45, 107]. Autophagy contributes to the survival of CSCs in breast cancer [108], and its inhibition can lead to the elimination of subpopulations of CRCs [109]. Thus, we can infer that the elimination of CSCs after autophagy inhibition may potentiate the efficacy of Doxo and Rsv+Doxo treatments. Molecularly, two genes suggested by our in silico analysis as potential targets of Rsv actually modulate the stemness of breast CSCs: HSP90AA1 and SHP2. HSP90 is the most important molecular chaperone involved in the response to stress, enabling cancer cells to survive under adverse conditions [110] and resist therapy [111]. HSP90AA1 is a NANOG transcriptional target that contributes to the maintenance of cancer cell stemness, and its overexpression of HSP90AA1 was associated with unfavorable prognosis in breast cancer [112], while the inhibition of HSP90 reduces tumor stemness and promotes antitumor immunity. In lung cancer, HSP90 inhibitors have been found to synergize with Doxo in vitro and in vivo [113]. The reduction of heat shock proteins by Rsv increases the sensitivity of breast cancer cells to Doxo [114], but this effect has not been tested in MCF7 cells. Notwithstanding, Rsv has been shown to reduce HSP90 in other cell types [115, 116]. The other gene involved in the maintenance of tumor-initiating cells that may be modulated by Rsv is SHP2 [55, 93, 105].

In conclusion, here, we found that Rsv potentiated the long-term response of MCF7 breast cancer cells to Doxo. Our model showed that acute treatment with these drugs led to long-term sensitivity, which was even higher when autophagy was rationally suppressed. Indeed, we believe experimental designs that resemble and reflect the clinics may improve the translationality of in vitro data. Our in silico analysis shed light at the molecular level on potential players modulated by Rsv in the context of Doxo treatment. Our results suggest that alternative regimens of treatment, including those that employ the rational modulation of cellular mechanisms, are promising for the development of new research and therapies on overcoming Doxo resistance in breast cancer.

## Data Availability

The sources of all data used in this manuscript are described along the text.

## Supplementary Materials

Supplementary 1 Supplementary Figure 1 Dose-response curve to Rsv and Doxo. (A) Cells were treated with the following doses: Rsv 10, 30, 60, or 120 *μ*M; Doxo 100 or 200 nM. After 24 h, cell viability was assessed using a trypan blue exclusion assay. (B) SA-*β*-gal chromogenic staining. (C) Nuclear morphometric analysis. Each dot represents a single nucleus. Nuclear populations are defined by objective thresholds set based on the control. The percentage of nuclei in each population is shown on the right. ^∗^*p* < 0.05, ^∗∗^*p* < 0.01, and ^∗∗∗^*p* < 0.001 in relation to control; ^#^*p* < 0.05, ^##^*p* < 0.01, and ^###^*p* < 0.001 in relation to Doxo.

Supplementary 2 Supplementary Figure 2 Kinetics of acridine orange staining after Doxo treatment and cell growth after early autophagy inhibition. (A) Cells were treated with Doxo 100 nM for 24 h, followed by cell replating in a Drug-Free Medium. The percentage of AO-positive cells and the intensity of red fluorescence in AO-positive cells were determined 5, 10, and 15 days after treatment. (B) Autophagy was inhibited with 2 mM of 3-MA for 1 h before treatments. Cumulative Population Doubling was performed after 15 days. ^∗^*p* < 0.05, ^∗∗^*p* < 0.01, and ^∗∗∗^*p* < 0.001 in relation to control; ^#^*p* < 0.05, ^##^*p* < 0.01, and ^###^*p* < 0.001 in relation to Doxo.

Supplementary 3 Supplementary Figure 3 Functional analysis of the intersection CP-PPI network showing well-described pathways for breast cancer. (A) Intersection KEGG/REACTOME pathway network analysis of related terms (sharing similar associated genes from ClueGO). (B) Most significant pathways based on REACTOME and KEGG were analyzed with ClueGO algorithms. (C) ClueGO pie chart showing overexpressed terms after prediction.

Supplementary 4 Supplementary Figure 4 H-B networks for MCF7 and breast cancer. (A) H-B breast cancer CP-PPI network. (B) H-B MCF7 CP-PPI network.

Supplementary 5 Supplementary Figure 5 H-B comparison between MCF7/breast cancer and BT483 and MDA-MD-231. (A) BT483 CP-PPI network and MDA-MD-231 CP-PPI network. (B) Venn diagram comparing the top five H-Bs obtained from Figure 7(d) and H-Bs of the BT483 CP-PPI network and H-B MDA-MD-231 CP-PPI network.

Supplementary 6 Supplementary Table 1 Gene expression of MCF7 cells. Constitutive gene expression analysis of three sample replicates obtained from GSE63427, microarray experiment.

Supplementary 7 Supplementary Table 2 CentiScape analysis of the intersection CP-PPI network. (A) Degree and betweenness centrality scores of each node. (B) Hub and bottleneck nodes.

Supplementary 8 Supplementary Table 3 Experimental evidences assessing the modulation of putative genes by Rsv in breast cancer cells. We search for studies assessing the effect of Rsv in the 7 putative genes found through the in silico strategy.
