# Supplementary figures and images for: Cellular Mechanisms Triggered by the Cotreatment of Resveratrol and Doxorubicin in Breast Cancer: A Translational In Vitro–In Silico Model
Source: Oxid Med Cell Longev. 2020 Nov 1;2020:5432651. doi: 10.1155/2020/5432651 (PMC7654215; doi:10.1155/2020/5432651)

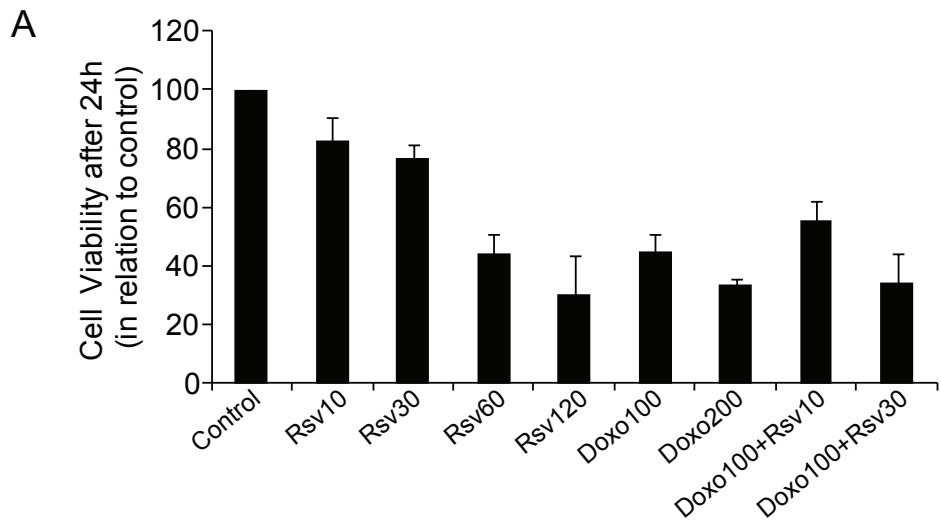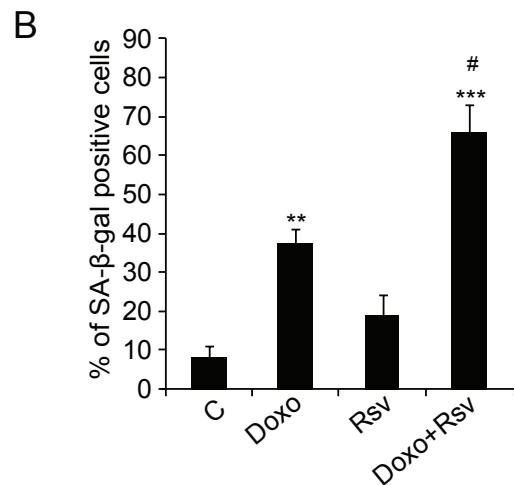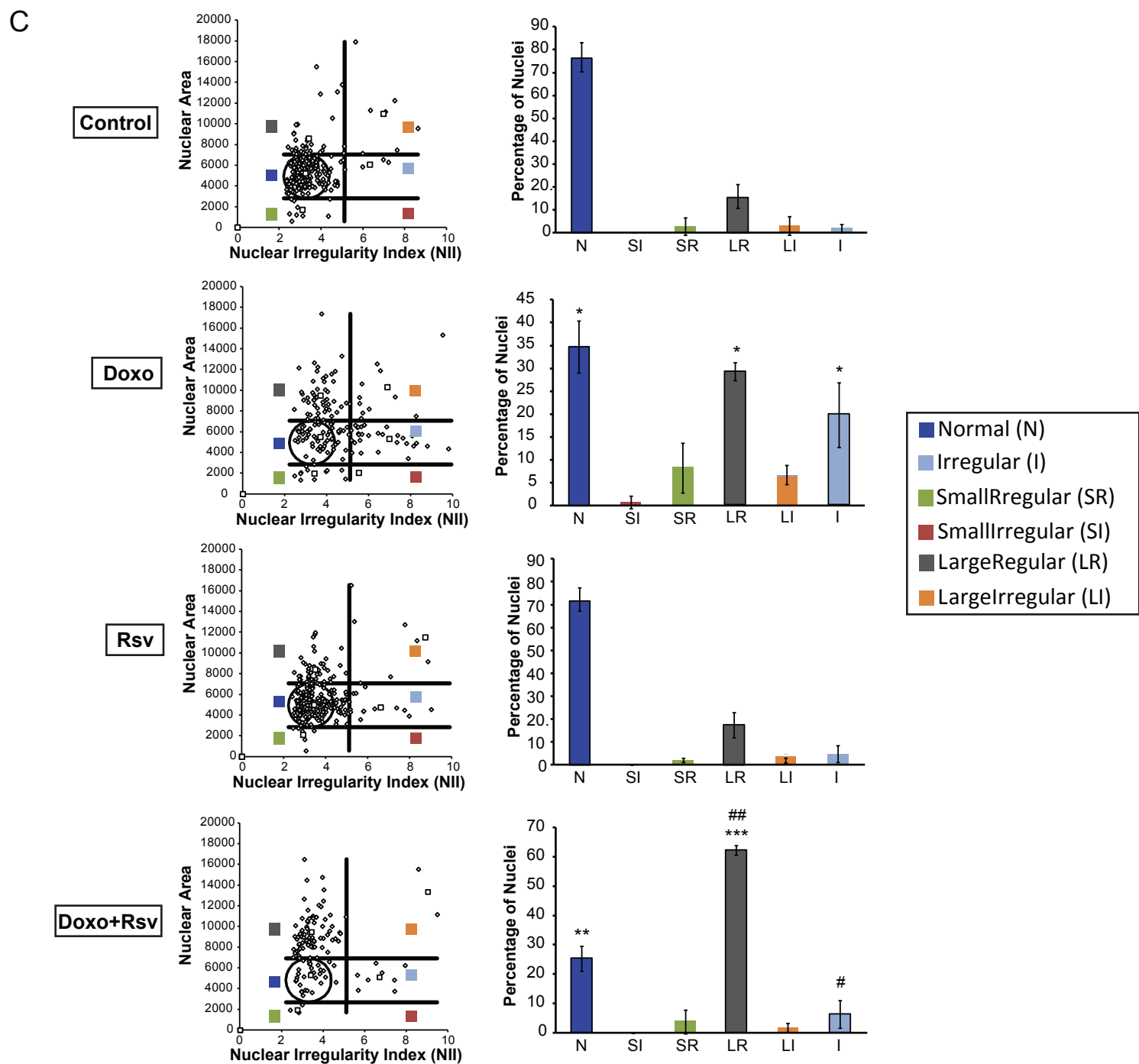

Supplement: Supplementary 1 — Supplementary Figure 1 Dose-response curve to Rsv and Doxo. (A) Cells were treated with the following doses: Rsv 10, 30, 60, or 120 μM; Doxo 100 or 200 nM. After 24 h, cell viability was assessed using a trypan blue exclusion assay. (B) SA-β-gal chromogenic staining. (C) Nuclear morphometric analysis. Each dot represents a single nucleus. Nuclear populations are defined by objective thresholds set based on the control. The percentage of nuclei in each population is shown on the right. ∗p < 0.05, ∗∗p < 0.01, and ∗∗∗p < 0.001 in relation to control; #p < 0.05, ##p < 0.01, and ###p < 0.001 in relation to Doxo. [file 5432651.f1.pdf]

A

Intersection  
KEGG/REACTOME  
pathway term network

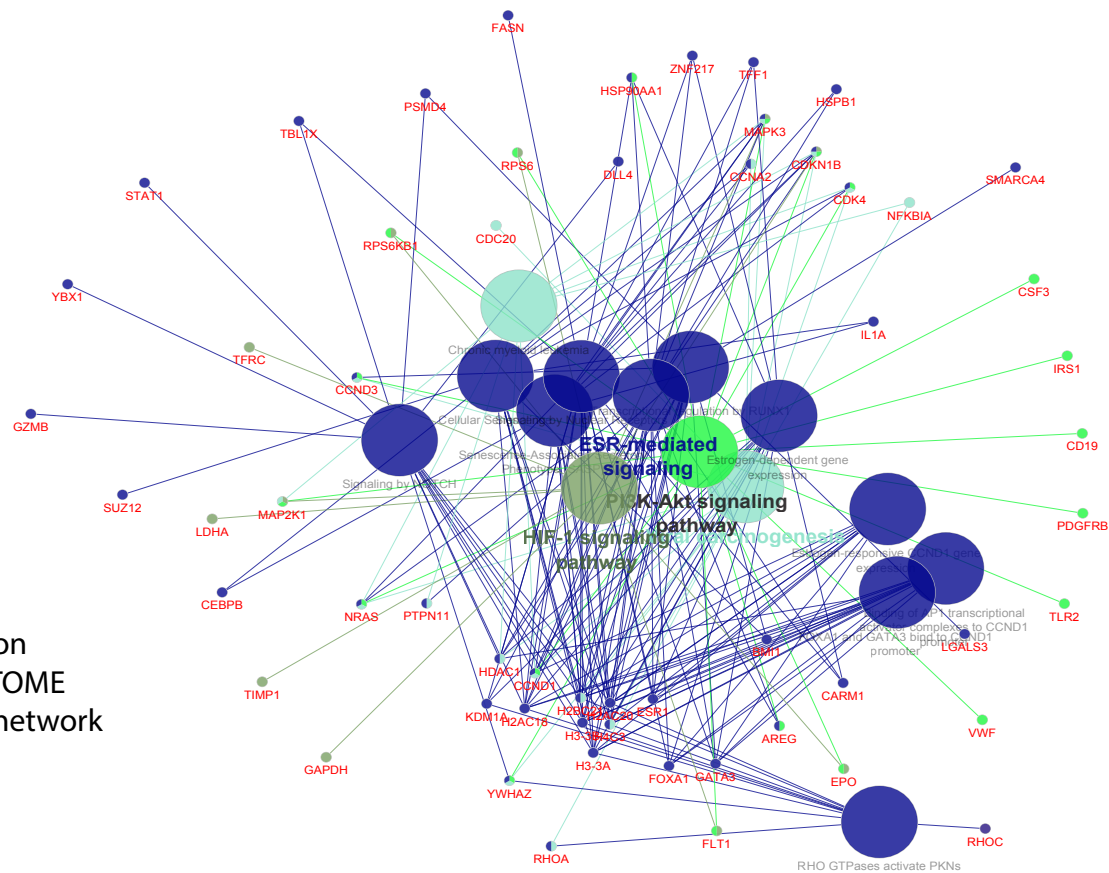

B

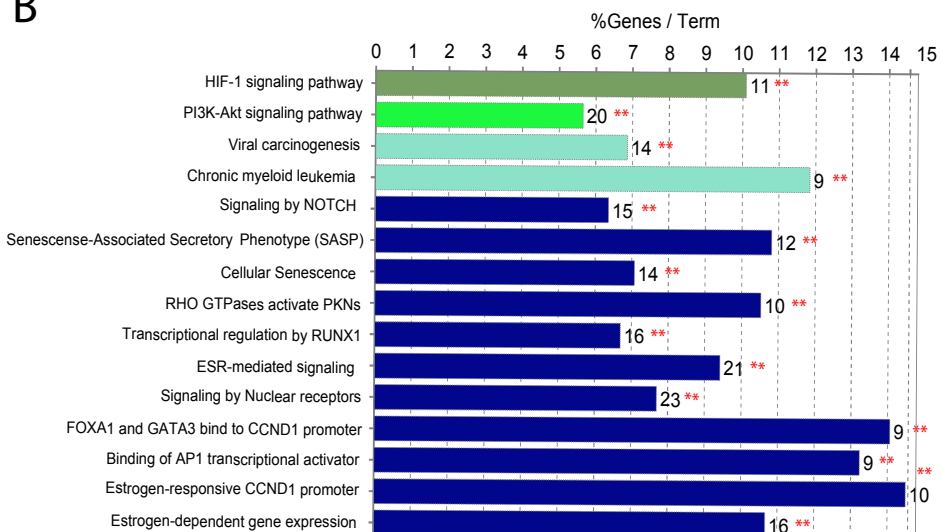

C

% Over-represented terms

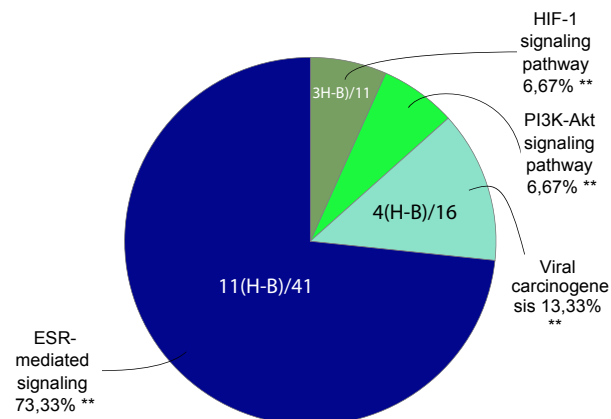

Supplement: Supplementary 3 — Supplementary Figure 3 Functional analysis of the intersection CP-PPI network showing well-described pathways for breast cancer. (A) Intersection KEGG/REACTOME pathway network analysis of related terms (sharing similar associated genes from ClueGO). (B) Most significant pathways based on REACTOME and KEGG were analyzed with ClueGO algorithms. (C) ClueGO pie chart showing overexpressed terms after prediction. [file 5432651.f3.pdf]

A

H-B Breast cancer CP-PPI network

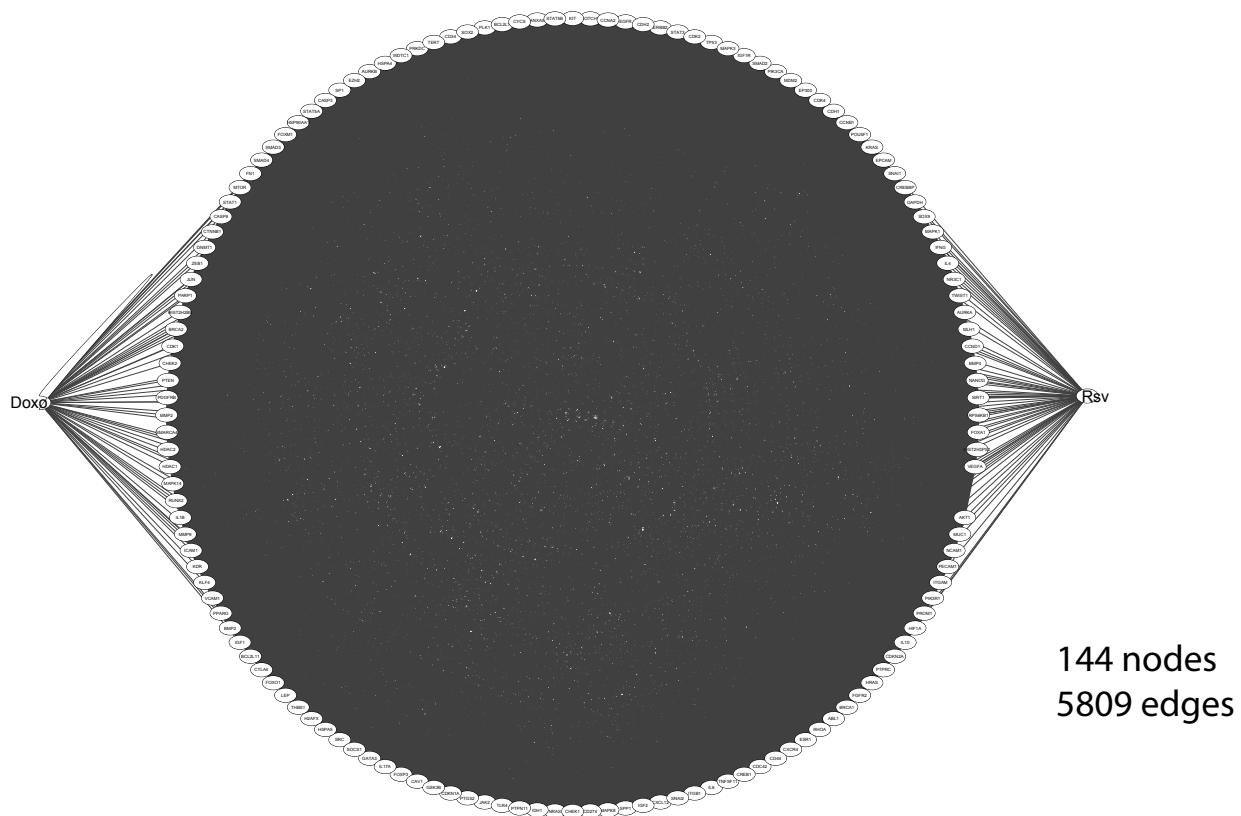

B

H-B MCF7 CP-PPI network

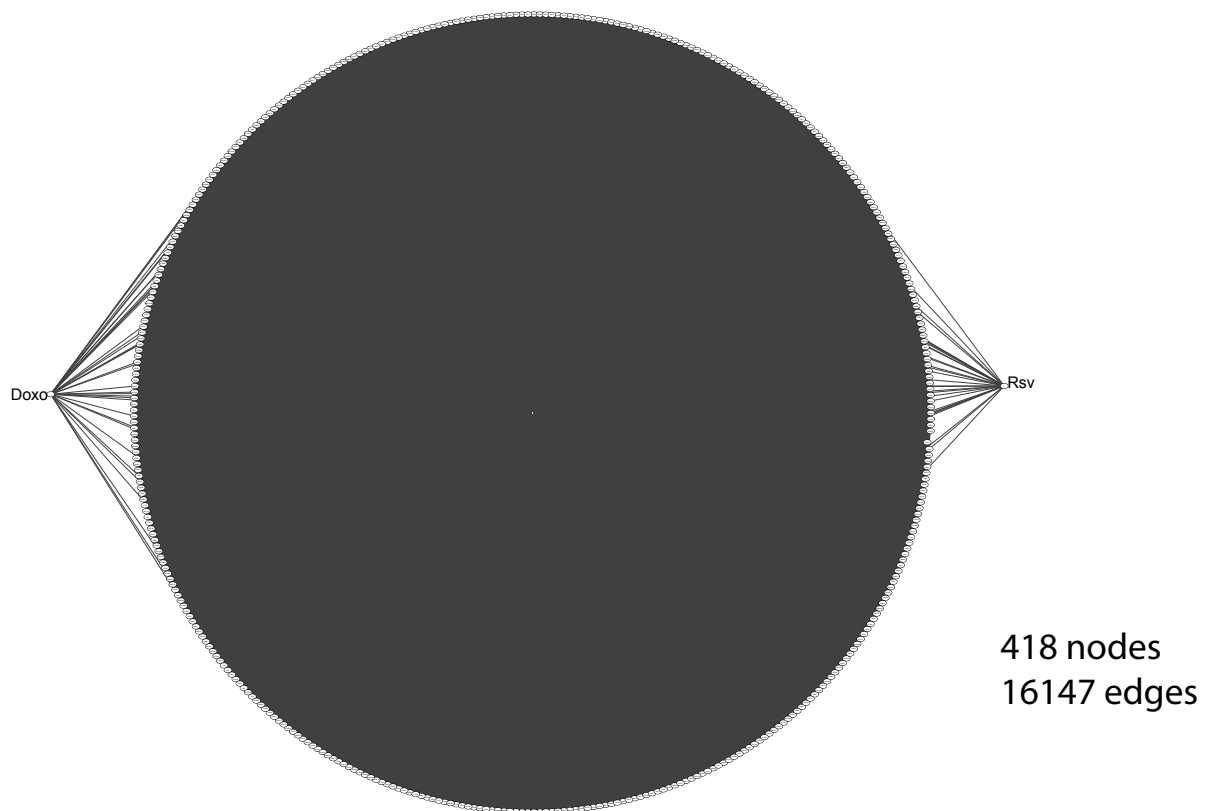

Supplement: Supplementary 4 — Supplementary Figure 4 H-B networks for MCF7 and breast cancer. (A) H-B breast cancer CP-PPI network. (B) H-B MCF7 CP-PPI network. [file 5432651.f4.pdf]

A

BT483  
1080 nodes  
17186 edges

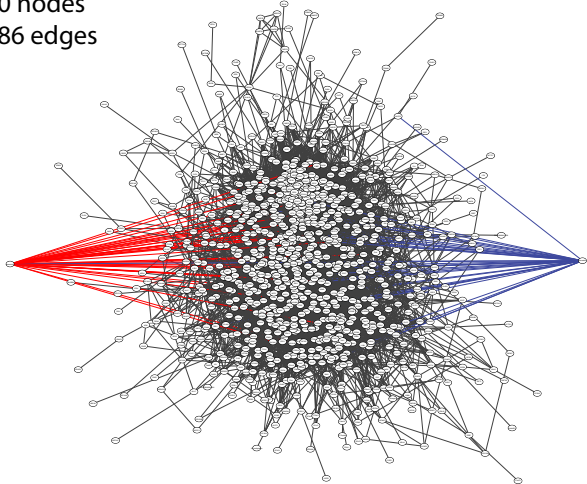

MDA-MD-231  
1656 nodes  
28519 edges

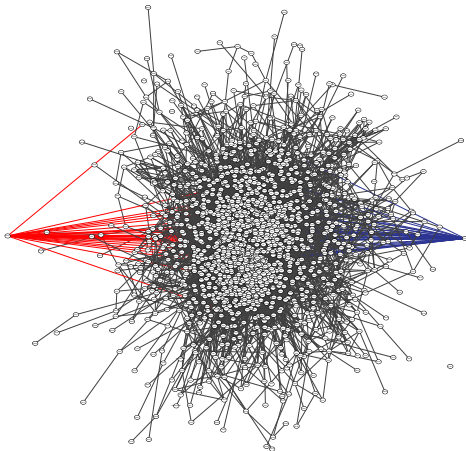

B

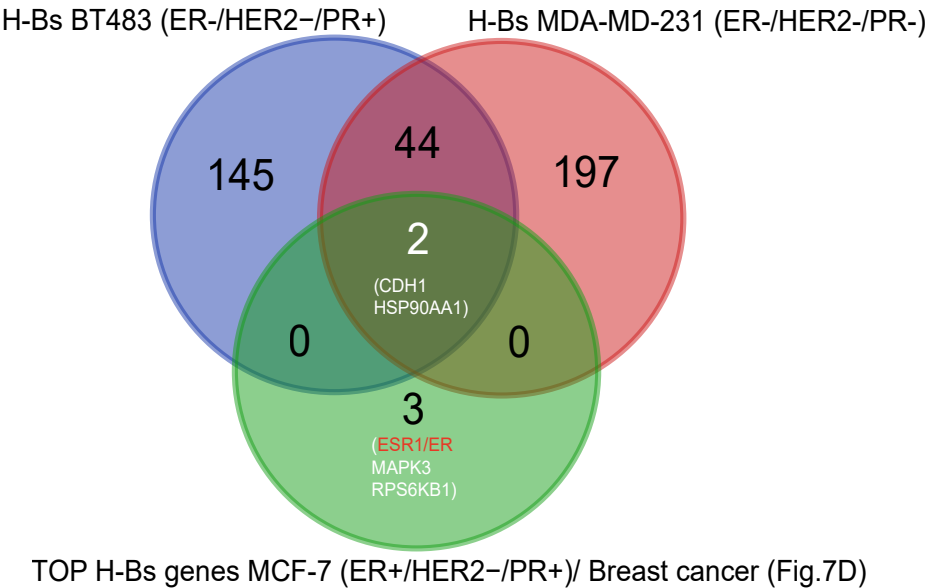

Supplement: Supplementary 5 — Supplementary Figure 5 H-B comparison between MCF7/breast cancer and BT483 and MDA-MD-231. (A) BT483 CP-PPI network and MDA-MD-231 CP-PPI network. (B) Venn diagram comparing the top five H-Bs obtained from Figure 7(d) and H-Bs of the BT483 CP-PPI network and H-B MDA-MD-231 CP-PPI network. [file 5432651.f5.pdf]
