# Supplementary material for: Cellular Mechanisms Triggered by the Cotreatment of Resveratrol and Doxorubicin in Breast Cancer: A Translational In Vitro–In Silico Model
Source: Oxid Med Cell Longev. 2020 Nov 1;2020:5432651. doi: 10.1155/2020/5432651 (PMC7654215; doi:10.1155/2020/5432651)
Supplement: Supplementary 6 — Supplementary Table 1 Gene expression of MCF7 cells. Constitutive gene expression analysis of three sample replicates obtained from GSE63427, microarray experiment. [file 5432651.f6.docx]

| **Supplementary table 1**. Gene expression analysis of three sample replicates from GSE63427. | | | | | | |  |  |
| --- | --- | --- | --- | --- | --- | --- | --- | --- |
| ILMN_Gene | p-Values (Up) | q-Values (Up) | RP-Values (Up) | Mean | std.dev | GSM1915062 | GSM1915063 | GSM1915064 |
| MARCH7 | 0.003972474 | 0.09624372 | 1437.967 | 10.44943 | 0.05894143 | 10.392238 | 10.446077 | 10.509977 |
| AARS | 0.000153946 | 0.012777512 | 419.82645 | 12.15994 | 0.10659863 | 12.271824 | 12.05956 | 12.148436 |
| ABCF1 | 0.007716003 | 0.1408201 | 1892.4164 | 10.054829 | 0.109208636 | 10.16384 | 9.945424 | 10.0552225 |
| ACBD3 | 0.009829942 | 0.16356835 | 2093.686 | 9.911141 | 0.19328071 | 9.7491865 | 9.859131 | 10.125105 |
| ACLY | 0.006294604 | 0.12616117 | 1738.018 | 10.180034 | 0.07325724 | 10.26387 | 10.147875 | 10.128357 |
| ACP1 | 0.002005736 | 0.06331847 | 1093.5153 | 10.837921 | 0.09990782 | 10.730799 | 10.854397 | 10.928566 |
| ACTB | 9.68467E-06 | 0.002024096 | 166.92511 | 13.297681 | 0.05063837 | 13.276914 | 13.355401 | 13.260727 |
| ACTG1 | 2.42117E-06 | 0.000815534 | 104.65921 | 13.664422 | 0.02420767 | 13.65182 | 13.649116 | 13.692331 |
| ACTL6A | 0.001651813 | 0.056516767 | 1013.1932 | 10.952516 | 0.10680611 | 10.840897 | 10.962902 | 11.05375 |
| ACTN1 | 0.00465141 | 0.10602891 | 1533.7145 | 10.359418 | 0.03956393 | 10.365455 | 10.395617 | 10.317183 |
| ACTR10 | 0.00992428 | 0.16442837 | 2102.1423 | 9.901055 | 0.1063395 | 9.955769 | 9.7785 | 9.968899 |
| ACTR2 | 0.001570012 | 0.055187438 | 993.8515 | 10.981814 | 0.26890507 | 11.22606 | 11.025725 | 10.693655 |
| ADCY3 | 0.00892797 | 0.1539498 | 2011.2802 | 9.966842 | 0.089752465 | 9.924737 | 9.905882 | 10.069905 |
| ADIPOR2 | 0.008660979 | 0.15168299 | 1985.1925 | 9.987647 | 0.12173599 | 10.063529 | 10.05218 | 9.847231 |
| ADRM1 | 0.002456073 | 0.07184739 | 1184.5172 | 10.728645 | 0.029412642 | 10.722238 | 10.760734 | 10.702965 |
| ADSL | 0.003685162 | 0.09184842 | 1394.3309 | 10.491071 | 0.14353591 | 10.469901 | 10.644016 | 10.359296 |
| AFG3L2 | 0.007399666 | 0.13772747 | 1859.75 | 10.083784 | 0.08251025 | 10.003449 | 10.079593 | 10.16831 |
| AGR2 | 0.000955987 | 0.04030012 | 819.3567 | 11.256611 | 0.04118973 | 11.230914 | 11.30412 | 11.234799 |
| AHCY | 0.001887329 | 0.06125257 | 1068.193 | 10.871572 | 0.08697314 | 10.803875 | 10.969664 | 10.841178 |
| AHCYL1 | 0.005542111 | 0.116602786 | 1648.897 | 10.251813 | 0.05938714 | 10.204506 | 10.3184595 | 10.232475 |
| AHSA1 | 0.002805413 | 0.07842949 | 1248.6389 | 10.652884 | 0.1020156 | 10.541898 | 10.742564 | 10.674189 |
| AIDA | 0.002893152 | 0.07959952 | 1264.5913 | 10.6312895 | 0.13300171 | 10.783053 | 10.575783 | 10.535031 |
| AIF1L | 0.002039661 | 0.06398192 | 1100.8896 | 10.833923 | 0.026916668 | 10.8619375 | 10.808258 | 10.831575 |
| AIMP2 | 0.001074336 | 0.0436452 | 856.9895 | 11.194969 | 0.05017057 | 11.244417 | 11.196382 | 11.144106 |
| AK3 | 0.009826829 | 0.163595 | 2093.399 | 9.913159 | 0.20730066 | 9.734403 | 10.140408 | 9.864668 |
| AKIRIN2 | 0.00929374 | 0.15751685 | 2045.1461 | 9.943164 | 0.024973528 | 9.96317 | 9.9151745 | 9.951145 |
| AKR1C3 | 0.006364357 | 0.12697239 | 1746.0199 | 10.172582 | 0.07564633 | 10.103813 | 10.253609 | 10.160324 |
| AKR7A2 | 0.000639794 | 0.031619657 | 705.31616 | 11.439961 | 0.095702715 | 11.3416605 | 11.445388 | 11.532835 |
| ALDH7A1 | 0.004067101 | 0.097447515 | 1451.6346 | 10.4320135 | 0.16916338 | 10.349559 | 10.626594 | 10.319887 |
| ALDH9A1 | 0.000697758 | 0.033252746 | 728.28534 | 11.39936 | 0.13782652 | 11.265256 | 11.392194 | 11.540629 |
| ALG5 | 0.00617516 | 0.124921866 | 1724.2269 | 10.189311 | 0.13768718 | 10.088333 | 10.1334505 | 10.34615 |
| ALKBH5 | 0.000245864 | 0.017197581 | 497.5076 | 11.936699 | 0.07292457 | 11.863475 | 11.937301 | 12.00932 |
| ALPP | 0.001289964 | 0.048698585 | 919.6375 | 11.096875 | 0.050363503 | 11.154574 | 11.07432 | 11.061732 |
| AMY1C | 7.62668E-05 | 0.00796988 | 329.36172 | 12.50957 | 0.058052257 | 12.53089 | 12.4438715 | 12.5539465 |
| ANAPC13 | 0.000297861 | 0.019646388 | 532.66583 | 11.825559 | 0.07612717 | 11.899285 | 11.830154 | 11.747239 |
| ANAPC5 | 0.002604773 | 0.074871585 | 1212.8402 | 10.688965 | 0.1694204 | 10.749276 | 10.819977 | 10.497641 |
| ANKIB1 | 0.008751859 | 0.15242821 | 1994.0198 | 9.983846 | 0.06723302 | 10.004447 | 9.908723 | 10.038368 |
| ANKRD30B | 0.001626708 | 0.0561004 | 1007.3642 | 10.962443 | 0.09288958 | 11.037524 | 10.858565 | 10.991239 |
| ANP32B | 2.06376E-05 | 0.003393365 | 211.76082 | 13.035207 | 0.0888293 | 12.939257 | 13.114579 | 13.051786 |
| ANXA2 | 0.009747766 | 0.1631399 | 2086.2498 | 9.91493 | 0.037502766 | 9.893313 | 9.893243 | 9.958235 |
| ANXA2P2 | 0 | 0 | 10.857671 | 14.231083 | 0.13341893 | 14.357943 | 14.243352 | 14.091952 |
| ANXA5 | 0.00463034 | 0.1056875 | 1530.9443 | 10.360389 | 0.15004271 | 10.397693 | 10.48826 | 10.195213 |
| AOF2 | 0.00539001 | 0.1144437 | 1629.8528 | 10.271526 | 0.105967395 | 10.324522 | 10.340542 | 10.149516 |
| AP1S1 | 0.008829568 | 0.1531665 | 2001.7247 | 9.976529 | 0.093292415 | 9.874948 | 10.058374 | 9.996264 |
| AP2M1 | 0.008391624 | 0.1485403 | 1959.2338 | 10.008004 | 0.04012243 | 10.039508 | 9.962833 | 10.021669 |
| AP2S1 | 0.000110019 | 0.010205883 | 372.27084 | 12.320263 | 0.04290567 | 12.363678 | 12.319226 | 12.277885 |
| AP3B1 | 0.004660201 | 0.10574297 | 1534.8762 | 10.358385 | 0.022411767 | 10.38409 | 10.348126 | 10.342939 |
| AP4B1 | 0.004742924 | 0.10636781 | 1546.1948 | 10.345971 | 0.0850977 | 10.410526 | 10.37785 | 10.2495365 |
| APEH | 0.009265723 | 0.1572725 | 2042.6696 | 9.944409 | 0.057585146 | 9.925674 | 10.009029 | 9.898525 |
| APEX1 | 0.001331931 | 0.04979526 | 931.8101 | 11.077428 | 0.034403756 | 11.111046 | 11.078949 | 11.042289 |
| APOA1BP | 0.003766962 | 0.09282031 | 1406.9531 | 10.478887 | 0.050478034 | 10.479334 | 10.52914 | 10.428187 |
| APPBP2 | 0.004565804 | 0.104835205 | 1521.832 | 10.369571 | 0.02983217 | 10.389532 | 10.383902 | 10.335277 |
| APRT | 0.001791895 | 0.059434034 | 1046.4708 | 10.910398 | 0.1251528 | 11.045129 | 10.888297 | 10.797768 |
| ARCN1 | 0.003099095 | 0.08315545 | 1299.6356 | 10.588626 | 0.11341554 | 10.46468 | 10.687221 | 10.613976 |
| ARD1A | 0.005904162 | 0.12142205 | 1692.0897 | 10.218755 | 0.08943592 | 10.207388 | 10.313332 | 10.135547 |
| ARF4 | 0.006450395 | 0.12795311 | 1755.9047 | 10.163891 | 0.08882428 | 10.2568035 | 10.079816 | 10.155051 |
| ARF5 | 0.008165534 | 0.14648138 | 1937.2211 | 10.024304 | 0.08888919 | 10.071016 | 10.0801 | 9.921799 |
| ARGLU1 | 0.007826714 | 0.14224201 | 1903.6185 | 10.049829 | 0.09088964 | 9.946589 | 10.085108 | 10.117791 |
| ARL2 | 0.002610134 | 0.07496358 | 1213.832 | 10.690175 | 0.06705432 | 10.761972 | 10.629173 | 10.679382 |
| ARL6IP1 | 0.00615458 | 0.12472372 | 1721.8328 | 10.188737 | 0.06525948 | 10.214027 | 10.114617 | 10.237566 |
| ARL6IP5 | 0.005317663 | 0.11353292 | 1620.601 | 10.278111 | 0.07763332 | 10.304312 | 10.339255 | 10.190768 |
| ARL8B | 0.005948839 | 0.12205145 | 1697.7878 | 10.212987 | 0.08946221 | 10.178444 | 10.31457 | 10.145946 |
| ARMET | 0.001575604 | 0.055216163 | 995.2612 | 10.979453 | 0.095423326 | 11.063567 | 10.875758 | 10.999034 |
| ARPC1A | 0.000574105 | 0.029772796 | 677.60504 | 11.495336 | 0.070632234 | 11.41378 | 11.535501 | 11.536728 |
| ARPC3 | 0.000225803 | 0.016253112 | 481.89288 | 11.978001 | 0.030493097 | 11.987653 | 11.94385 | 12.0025 |
| ARPC5 | 0.000244624 | 0.017145455 | 496.57025 | 11.921025 | 0.09856508 | 11.904491 | 12.026813 | 11.831774 |
| ARPC5L | 0.00827881 | 0.1474461 | 1948.2817 | 10.016425 | 0.06456846 | 10.074768 | 10.027454 | 9.947052 |
| ARPP19 | 0.008264945 | 0.14757797 | 1946.9905 | 10.017045 | 0.032662902 | 10.054746 | 9.999143 | 9.997249 |
| ASCC2 | 0.006170375 | 0.1248979 | 1723.6615 | 10.191162 | 0.15610576 | 10.332813 | 10.216877 | 10.023795 |
| ASNSD1 | 0.005756355 | 0.11923045 | 1674.5576 | 10.23088 | 0.10832603 | 10.119383 | 10.237529 | 10.335729 |
| ASPM | 0.005173431 | 0.11169073 | 1602.273 | 10.293075 | 0.19884896 | 10.503839 | 10.108795 | 10.266589 |
| ATAD2 | 0.005926097 | 0.121800944 | 1694.9377 | 10.212358 | 0.11333064 | 10.270212 | 10.081778 | 10.285086 |
| ATF5 | 0.007223814 | 0.13620815 | 1841.0271 | 10.100394 | 0.057851296 | 10.036157 | 10.148384 | 10.116644 |
| ATIC | 0.000253589 | 0.017525896 | 503.65652 | 11.90721 | 0.09380847 | 11.907952 | 11.813033 | 12.000646 |
| ATOX1 | 0.00162544 | 0.056168325 | 1007.0371 | 10.9547415 | 0.0418914 | 10.96695 | 10.989171 | 10.908101 |
| ATP1A1 | 0.004287917 | 0.10051689 | 1483.3629 | 10.406128 | 0.053902417 | 10.380925 | 10.468015 | 10.369443 |
| ATP1B1 | 0.004138843 | 0.09855388 | 1462.2539 | 10.423964 | 0.045096748 | 10.435822 | 10.4619465 | 10.374123 |
| ATP5A1 | 0.000110394 | 0.010213333 | 372.5818 | 12.318202 | 0.0423504 | 12.366991 | 12.29668 | 12.290934 |
| ATP5B | 1.46999E-06 | 0.000554348 | 93.19526 | 13.735313 | 0.10132722 | 13.846977 | 13.709744 | 13.649221 |
| ATP5C1 | 0.000385312 | 0.022773424 | 584.9101 | 11.697097 | 0.12145011 | 11.61293 | 11.642035 | 11.836325 |
| ATP5E | 5.53698E-05 | 0.006534013 | 294.93036 | 12.645512 | 0.03451617 | 12.620532 | 12.631106 | 12.684897 |
| ATP5EP2 | 2.88234E-08 | 2.43902E-05 | 41.287292 | 14.041737 | 0.042305723 | 14.090587 | 14.017242 | 14.01738 |
| ATP5F1 | 0.000787197 | 0.03560756 | 762.0579 | 11.3435335 | 0.06260214 | 11.309189 | 11.415791 | 11.30562 |
| ATP5G1 | 0.005988529 | 0.12257581 | 1702.2985 | 10.207466 | 0.013584862 | 10.220602 | 10.208323 | 10.193473 |
| ATP5H | 4.49645E-05 | 0.005652174 | 274.74493 | 12.740275 | 0.06273988 | 12.748992 | 12.7982 | 12.673632 |
| ATP5J | 0.001307027 | 0.049075756 | 924.5483 | 11.0885935 | 0.02671444 | 11.11365 | 11.060484 | 11.091648 |
| ATP5J2 | 0.000118522 | 0.010764398 | 382.87137 | 12.280597 | 0.03992822 | 12.317906 | 12.285399 | 12.238484 |
| ATP5L | 0.000559636 | 0.029196993 | 671.26764 | 11.502608 | 0.026711952 | 11.471978 | 11.514772 | 11.521071 |
| ATP5O | 0.000290511 | 0.019382693 | 527.4637 | 11.852104 | 0.13475457 | 11.87879 | 11.706004 | 11.97152 |
| ATP6AP1 | 0.000174872 | 0.01375737 | 438.63278 | 12.0943365 | 0.01586305 | 12.106387 | 12.0763645 | 12.100258 |
| ATP6AP2 | 0.00066389 | 0.032213986 | 715.0555 | 11.426063 | 0.04290386 | 11.41861 | 11.472204 | 11.387373 |
| ATP6V0E1 | 0.000181876 | 0.014116331 | 445.5546 | 12.08167 | 0.071065955 | 12.089541 | 12.006997 | 12.148474 |
| ATP6V0E2 | 0.003140485 | 0.083683565 | 1306.5582 | 10.586892 | 0.07068866 | 10.529953 | 10.564713 | 10.666011 |
| ATP6V1A | 0.000839684 | 0.03701652 | 781.001 | 11.314705 | 0.07356466 | 11.2796135 | 11.399244 | 11.265256 |
| ATP6V1D | 0.002879316 | 0.07953423 | 1262.2227 | 10.629562 | 0.06871148 | 10.703756 | 10.61681 | 10.56812 |
| ATP6V1E1 | 0.001909812 | 0.061751164 | 1072.8542 | 10.867973 | 0.052681617 | 10.807267 | 10.9017105 | 10.894941 |
| ATP6V1F | 0.001942036 | 0.062270794 | 1080.0817 | 10.854103 | 0.16337323 | 10.9286 | 10.96695 | 10.66676 |
| ATP6V1G1 | 0.000138324 | 0.011937811 | 404.30685 | 12.206288 | 0.13405184 | 12.055808 | 12.250117 | 12.31294 |
| AURKA | 0.001386868 | 0.05102439 | 946.89825 | 11.053155 | 0.024260214 | 11.027286 | 11.056783 | 11.0753975 |
| AURKAIP1 | 0.000199573 | 0.015052174 | 460.31863 | 12.037315 | 0.019081514 | 12.034553 | 12.019766 | 12.057628 |
| AVPI1 | 0.005201505 | 0.11215724 | 1605.8556 | 10.290737 | 0.05968547 | 10.235824 | 10.3542595 | 10.282127 |
| B2M | 0.000161642 | 0.013072261 | 426.57657 | 12.132974 | 0.062231686 | 12.154689 | 12.181439 | 12.062795 |
| BAG3 | 0.001576469 | 0.055190716 | 995.48065 | 10.977031 | 0.0930071 | 11.081892 | 10.944687 | 10.904514 |
| BAMBI | 0.000102237 | 0.00977135 | 363.02878 | 12.36071 | 0.09135654 | 12.348226 | 12.457667 | 12.276238 |
| BANF1 | 0.006062172 | 0.123791054 | 1710.6053 | 10.202823 | 0.0448121 | 10.193318 | 10.251624 | 10.163525 |
| BASP1 | 0.000176861 | 0.013851016 | 440.47296 | 12.0892515 | 0.050227545 | 12.128668 | 12.032698 | 12.106387 |
| BCAP31 | 0.00061858 | 0.031375732 | 696.7905 | 11.455246 | 0.030368019 | 11.490313 | 11.437714 | 11.437714 |
| BCKDK | 0.002809996 | 0.07843121 | 1249.404 | 10.644103 | 0.07396905 | 10.717388 | 10.645452 | 10.5694685 |
| BCYRN1 | 0.000286851 | 0.019286823 | 524.9627 | 11.842799 | 0.08839779 | 11.926257 | 11.750175 | 11.851962 |
| BLVRA | 0.001969447 | 0.06268624 | 1085.9008 | 10.852126 | 0.08405805 | 10.948599 | 10.813137 | 10.794642 |
| BLVRB | 0.008408774 | 0.14869215 | 1961.0427 | 10.006648 | 0.03372808 | 9.980924 | 10.044834 | 9.994188 |
| BMI1 | 0.001926673 | 0.062064994 | 1076.7529 | 10.864586 | 0.066312745 | 10.934816 | 10.855894 | 10.803048 |
| BMP7 | 0.000192368 | 0.014700441 | 454.7166 | 12.047341 | 0.10410214 | 12.085546 | 12.1269455 | 11.929536 |
| BMS1P5 | 0.001374013 | 0.050712764 | 943.3036 | 11.05749 | 0.07470809 | 11.05077 | 10.98637 | 11.135332 |
| BNIP3 | 0.002034992 | 0.063893214 | 1099.8945 | 10.835148 | 0.025361354 | 10.858565 | 10.80821 | 10.838668 |
| BOLA2 | 0.006495043 | 0.12825213 | 1760.8499 | 10.159163 | 0.058486957 | 10.103932 | 10.220437 | 10.15312 |
| BOLA3 | 0.006956736 | 0.13349392 | 1812.3408 | 10.11758 | 0.04356948 | 10.147454 | 10.067587 | 10.1377 |
| BOP1 | 0.003810746 | 0.09343463 | 1413.9043 | 10.472953 | 0.07425582 | 10.434647 | 10.558539 | 10.425673 |
| BRD2 | 0.000636421 | 0.031724136 | 703.8403 | 11.444764 | 0.14163385 | 11.540629 | 11.282082 | 11.511581 |
| BRIX1 | 0.00325575 | 0.08531345 | 1325.6755 | 10.560647 | 0.04093763 | 10.594144 | 10.572785 | 10.515014 |
| BSCL2 | 0.001079783 | 0.04371295 | 858.7114 | 11.1929245 | 0.06038701 | 11.249754 | 11.199503 | 11.1295185 |
| BTBD2 | 0.001345737 | 0.04998822 | 935.6195 | 11.07222 | 0.10950421 | 11.056665 | 11.188668 | 10.971323 |
| BTBD6 | 0.005248458 | 0.11267946 | 1611.6671 | 10.285386 | 0.055852506 | 10.335729 | 10.225305 | 10.295125 |
| BTF3 | 0.000573298 | 0.02977545 | 677.2396 | 11.500106 | 0.09305868 | 11.572577 | 11.5325775 | 11.395162 |
| BTG1 | 0.000330461 | 0.020769928 | 553.81915 | 11.770943 | 0.10816931 | 11.753178 | 11.672757 | 11.886896 |
| BUD31 | 0.000519427 | 0.027896285 | 652.502 | 11.546777 | 0.0404788 | 11.593496 | 11.522185 | 11.52465 |
| BZW2 | 0.001356373 | 0.050221983 | 938.3377 | 11.063277 | 0.16235368 | 11.191915 | 11.11706 | 10.8808565 |
| C10ORF58 | 0.001270075 | 0.048 | 914.364 | 11.102631 | 0.0439044 | 11.096061 | 11.149448 | 11.06238 |
| C11ORF10 | 2.98322E-05 | 0.004367088 | 239.23013 | 12.905562 | 0.08650631 | 12.822429 | 12.995087 | 12.899169 |
| C11ORF2 | 0.005490863 | 0.115875915 | 1642.5803 | 10.264095 | 0.20457344 | 10.411224 | 10.350579 | 10.030484 |
| C11ORF48 | 0.00569378 | 0.1185003 | 1666.913 | 10.236252 | 0.020183181 | 10.257156 | 10.234723 | 10.216877 |
| C11ORF59 | 0.001864386 | 0.060964186 | 1063.0889 | 10.880104 | 0.14074962 | 10.9436865 | 10.718782 | 10.977846 |
| C12ORF10 | 0.002602352 | 0.074864015 | 1212.2872 | 10.689228 | 0.20789573 | 10.55385 | 10.9286 | 10.585233 |
| C12ORF41 | 0.007311178 | 0.13703619 | 1850.2573 | 10.09003 | 0.080295675 | 10.182372 | 10.036646 | 10.05107 |
| C12ORF47 | 0.005364155 | 0.11410423 | 1626.659 | 10.274259 | 0.035826042 | 10.237529 | 10.276138 | 10.309107 |
| C12ORF57 | 0.004031475 | 0.09672753 | 1446.6472 | 10.440846 | 0.08156155 | 10.367125 | 10.528462 | 10.42695 |
| C12ORF62 | 0.008910013 | 0.15371656 | 2009.6047 | 9.969779 | 0.17684467 | 9.969956 | 10.146535 | 9.792846 |
| C14ORF109 | 0.00324252 | 0.08522424 | 1323.4683 | 10.563838 | 0.14058173 | 10.408879 | 10.683199 | 10.599436 |
| C14ORF112 | 0.000399752 | 0.023309244 | 592.5327 | 11.672681 | 0.112592116 | 11.747239 | 11.727642 | 11.543165 |
| C14ORF156 | 1.2567E-05 | 0.002435754 | 181.33272 | 13.1998415 | 0.021985458 | 13.183961 | 13.190632 | 13.224936 |
| C14ORF166 | 0.000708509 | 0.0334891 | 732.5592 | 11.394147 | 0.070984654 | 11.331319 | 11.379973 | 11.471149 |
| C14ORF32 | 0.008322938 | 0.14792828 | 1952.5083 | 10.007653 | 0.14831339 | 10.17675 | 9.946589 | 9.89962 |
| C14ORF4 | 0.002647893 | 0.07560988 | 1220.6326 | 10.68102 | 0.075712055 | 10.655821 | 10.62112 | 10.766118 |
| C14ORF85 | 0.000476134 | 0.026055206 | 631.8382 | 11.593682 | 0.0899121 | 11.685071 | 11.505323 | 11.5906515 |
| C15ORF24 | 0.001620972 | 0.05612575 | 1006.06696 | 10.959 | 0.042426094 | 10.920919 | 10.951347 | 11.00473 |
| C16ORF33 | 0.000895313 | 0.038730673 | 799.7612 | 11.286359 | 0.083081946 | 11.242248 | 11.382194 | 11.234637 |
| C16ORF58 | 0.001592177 | 0.0553497 | 999.1067 | 10.974673 | 0.13936271 | 10.996015 | 11.102133 | 10.82587 |
| C16ORF61 | 6.66686E-05 | 0.007366242 | 314.34283 | 12.558144 | 0.16434759 | 12.43705 | 12.492151 | 12.745231 |
| C16ORF63 | 0.004531619 | 0.10439575 | 1517.3702 | 10.37168 | 0.047225144 | 10.326499 | 10.367829 | 10.420713 |
| C16ORF75 | 0.002905661 | 0.07975396 | 1266.7705 | 10.6290655 | 0.13577968 | 10.495523 | 10.766977 | 10.624696 |
| C17ORF45 | 3.07258E-05 | 0.004460251 | 241.54585 | 12.876969 | 0.038391978 | 12.846515 | 12.920095 | 12.864299 |
| C17ORF61 | 0.001702485 | 0.057851125 | 1025.4227 | 10.931172 | 0.094285704 | 10.8225765 | 10.992175 | 10.978766 |
| C17ORF71 | 0.006763533 | 0.1311649 | 1791.1042 | 10.137601 | 0.054814663 | 10.154793 | 10.181759 | 10.076251 |
| C19ORF2 | 0.003702254 | 0.09201003 | 1397.0651 | 10.488088 | 0.12022923 | 10.610435 | 10.370094 | 10.483735 |
| C19ORF31 | 0.00383758 | 0.09395977 | 1417.9418 | 10.468598 | 0.02233925 | 10.454455 | 10.494352 | 10.456988 |
| C19ORF43 | 0.004484147 | 0.103577234 | 1510.9631 | 10.37839 | 0.0896795 | 10.297341 | 10.474733 | 10.363096 |
| C19ORF53 | 0.003222344 | 0.08495137 | 1320.1774 | 10.564767 | 0.059736587 | 10.50029 | 10.61823 | 10.575783 |
| C19ORF56 | 0.000502162 | 0.027179407 | 644.1943 | 11.564448 | 0.070589915 | 11.551189 | 11.640728 | 11.501429 |
| C1ORF59 | 0.001732 | 0.0583965 | 1032.6649 | 10.923175 | 0.1515213 | 10.766977 | 11.069541 | 10.933008 |
| C1QBP | 0.000400012 | 0.023285234 | 592.7578 | 11.676921 | 0.09694332 | 11.68671 | 11.575455 | 11.768599 |
| C20ORF11 | 0.000531216 | 0.028310291 | 658.2798 | 11.531926 | 0.091836065 | 11.433855 | 11.546026 | 11.615896 |
| C20ORF111 | 0.008083473 | 0.14538518 | 1929.297 | 10.026244 | 0.09373377 | 10.130194 | 10.00038 | 9.948157 |
| C20ORF20 | 0.0009412 | 0.040017158 | 815.01416 | 11.264415 | 0.06385021 | 11.228041 | 11.33814 | 11.227061 |
| C20ORF24 | 0.000354298 | 0.021717314 | 567.65234 | 11.736523 | 0.04522056 | 11.733268 | 11.693017 | 11.783282 |
| C20ORF27 | 0.002841673 | 0.0791244 | 1255.1449 | 10.639328 | 0.11224714 | 10.751469 | 10.639541 | 10.526975 |
| C20ORF30 | 0.003877962 | 0.09481466 | 1424.1073 | 10.45725 | 0.1197697 | 10.338224 | 10.455776 | 10.57775 |
| C20ORF43 | 0.000228973 | 0.01634568 | 484.5583 | 11.956677 | 0.0712152 | 11.95083 | 12.030637 | 11.888567 |
| C20ORF45 | 0.004721998 | 0.10610428 | 1543.384 | 10.350415 | 0.036874413 | 10.382914 | 10.357993 | 10.310342 |
| C20ORF52 | 0.00020848 | 0.015488222 | 467.8971 | 11.996264 | 0.24174787 | 12.05956 | 12.2000675 | 11.729165 |
| C21ORF55 | 8.56056E-05 | 0.008633721 | 341.81723 | 12.45982 | 0.054508828 | 12.509168 | 12.401311 | 12.468981 |
| C22ORF13 | 0.006780999 | 0.13143016 | 1793.0146 | 10.133389 | 0.034449 | 10.131374 | 10.168802 | 10.099993 |
| C2ORF25 | 0.000823485 | 0.036675226 | 775.31683 | 11.327164 | 0.111992925 | 11.2181425 | 11.32144 | 11.441909 |
| C2ORF28 | 0.000703724 | 0.03339945 | 730.6574 | 11.398319 | 0.009033565 | 11.408617 | 11.394614 | 11.391728 |
| C2ORF29 | 0.001912752 | 0.06178864 | 1073.5421 | 10.869161 | 0.097813435 | 10.964922 | 10.769417 | 10.873145 |
| C2ORF47 | 0.005099787 | 0.11106843 | 1592.8405 | 10.304073 | 0.036844213 | 10.332089 | 10.262338 | 10.317793 |
| C2ORF69 | 0.00162789 | 0.056085404 | 1007.5914 | 10.956223 | 0.051410925 | 10.94195 | 10.913456 | 11.013262 |
| C3ORF10 | 0.00390739 | 0.09533263 | 1428.6456 | 10.454793 | 0.01627641 | 10.467958 | 10.459827 | 10.436595 |
| C3ORF14 | 0.001161786 | 0.045699548 | 883.85077 | 11.154655 | 0.031531297 | 11.11921 | 11.179586 | 11.165169 |
| C3ORF21 | 0.008337926 | 0.14804299 | 1953.9254 | 10.010718 | 0.0837468 | 10.068814 | 9.914721 | 10.048617 |
| C3ORF57 | 0.000123825 | 0.011015384 | 389.21213 | 12.263864 | 0.085676655 | 12.346756 | 12.269183 | 12.175651 |
| C4ORF34 | 0.000193002 | 0.014716484 | 455.44974 | 12.048225 | 0.056890707 | 12.015282 | 12.015478 | 12.113917 |
| C5ORF15 | 0.00253479 | 0.073653266 | 1199.211 | 10.708649 | 0.06742778 | 10.786502 | 10.66893 | 10.670513 |
| C6ORF115 | 0.00270479 | 0.07679214 | 1230.9445 | 10.676188 | 0.15646906 | 10.696088 | 10.510721 | 10.821755 |
| C6ORF125 | 0.002838531 | 0.07916398 | 1254.666 | 10.639889 | 0.003083958 | 10.636591 | 10.640375 | 10.642701 |
| C6ORF153 | 0.001385196 | 0.051016986 | 946.53156 | 11.052957 | 0.0335054 | 11.079111 | 11.01519 | 11.064569 |
| C6ORF160 | 0.000501067 | 0.0271625 | 643.5649 | 11.562214 | 0.21542993 | 11.534194 | 11.362165 | 11.790283 |
| C6ORF173 | 0.00251666 | 0.07324916 | 1195.7523 | 10.716049 | 0.05750792 | 10.679382 | 10.782329 | 10.686437 |
| C7ORF27 | 0.001650574 | 0.056530107 | 1012.90656 | 10.953789 | 0.06873865 | 11.021885 | 10.884425 | 10.955057 |
| C7ORF30 | 0.002173488 | 0.066262744 | 1128.3907 | 10.798448 | 0.0878565 | 10.735396 | 10.898801 | 10.7611475 |
| C7ORF44 | 0.001992045 | 0.063231476 | 1090.6713 | 10.845038 | 0.11533211 | 10.72181 | 10.950386 | 10.862919 |
| C7ORF50 | 9.46273E-05 | 0.009170391 | 353.8044 | 12.394485 | 0.13371615 | 12.361217 | 12.541696 | 12.280545 |
| C7ORF55 | 0.007014297 | 0.1337845 | 1818.5055 | 10.115919 | 0.107949175 | 10.235141 | 10.087816 | 10.024801 |
| C7ORF59 | 0.004294748 | 0.10060905 | 1484.2672 | 10.400758 | 0.05475131 | 10.339923 | 10.416271 | 10.446077 |
| C7ORF68 | 0.005528247 | 0.11645234 | 1647.1404 | 10.256353 | 0.1879293 | 10.049627 | 10.4168625 | 10.30257 |
| C8ORF55 | 0.000991007 | 0.041474067 | 830.6607 | 11.237179 | 0.12006159 | 11.138032 | 11.37067 | 11.202835 |
| C8ORF59 | 0.000379432 | 0.022696551 | 581.84625 | 11.709264 | 0.14584471 | 11.875546 | 11.649217 | 11.603029 |
| C8ORF76 | 0.001936646 | 0.062212963 | 1078.9832 | 10.861339 | 0.11255726 | 10.893782 | 10.736122 | 10.95411 |
| C9ORF114 | 0.006712976 | 0.13040313 | 1785.4744 | 10.142125 | 0.15209107 | 10.097912 | 10.311423 | 10.017038 |
| C9ORF142 | 0.002784343 | 0.07802908 | 1244.9458 | 10.648778 | 0.0974135 | 10.75956 | 10.610266 | 10.576508 |
| C9ORF78 | 0.000951577 | 0.04021194 | 818.1815 | 11.256717 | 0.05298761 | 11.308763 | 11.202835 | 11.2585535 |
| C9ORF80 | 0.00185888 | 0.06084151 | 1061.9934 | 10.8824415 | 0.04140281 | 10.909657 | 10.902873 | 10.834794 |
| CA12 | 0.007382516 | 0.13770376 | 1857.9186 | 10.082969 | 0.048109185 | 10.0798 | 10.036522 | 10.132584 |
| CA2 | 0.000595809 | 0.030533236 | 687.13074 | 11.476443 | 0.039569374 | 11.430803 | 11.497379 | 11.501144 |
| CAB39 | 0.0035833 | 0.09008623 | 1378.979 | 10.507243 | 0.08986684 | 10.419506 | 10.599098 | 10.503126 |
| CALM1 | 0.000629446 | 0.031603474 | 701.32086 | 11.44921 | 0.009758521 | 11.447931 | 11.440153 | 11.459544 |
| CALM2 | 2.5797E-05 | 0.003942731 | 227.42375 | 12.964523 | 0.063897125 | 12.935724 | 13.037752 | 12.920095 |
| CALM3 | 4.51375E-05 | 0.00565343 | 275.21112 | 12.729951 | 0.044101354 | 12.759544 | 12.751044 | 12.679264 |
| CALR | 0.007386003 | 0.13769479 | 1858.2561 | 10.082485 | 0.056249 | 10.081239 | 10.026869 | 10.139346 |
| CAMK2N1 | 0.006808555 | 0.13181697 | 1796.1274 | 10.130959 | 0.048672367 | 10.147164 | 10.076251 | 10.169461 |
| CAMLG | 0.000576613 | 0.02985821 | 678.6917 | 11.490113 | 0.06342702 | 11.427207 | 11.489085 | 11.554049 |
| CAP1 | 0.000673517 | 0.032409154 | 719.01483 | 11.423606 | 0.12937798 | 11.571266 | 11.369418 | 11.330132 |
| CAPN1 | 0.002102122 | 0.06500089 | 1114.2402 | 10.815249 | 0.08841233 | 10.809792 | 10.906263 | 10.7296915 |
| CAPNS1 | 0.008176688 | 0.14652996 | 1938.2734 | 10.023444 | 0.036871403 | 9.997508 | 10.065652 | 10.007171 |
| CAPZA2 | 0.003983484 | 0.096375875 | 1439.6089 | 10.444091 | 0.11725619 | 10.331431 | 10.565458 | 10.435384 |
| CARHSP1 | 0.000759497 | 0.03499336 | 751.5776 | 11.364178 | 0.02255813 | 11.376602 | 11.377793 | 11.33814 |
| CARM1 | 0.001195798 | 0.046199333 | 893.235 | 11.135983 | 0.025828911 | 11.151443 | 11.106166 | 11.150343 |
| CBS | 0.007008013 | 0.13381177 | 1817.9144 | 10.117774 | 0.07108102 | 10.046984 | 10.189142 | 10.117195 |
| CBWD5 | 0.008774312 | 0.152666 | 1996.1472 | 9.979 | 0.07282392 | 9.911754 | 9.968899 | 10.056347 |
| CBX1 | 0.009599701 | 0.1612062 | 2072.782 | 9.925475 | 0.15496507 | 9.833188 | 10.104383 | 9.838854 |
| CBX5 | 0.00399683 | 0.096496865 | 1441.5491 | 10.446262 | 0.06333097 | 10.461929 | 10.50029 | 10.376569 |
| CCDC109B | 0.002006572 | 0.06328727 | 1093.7191 | 10.8415985 | 0.03931362 | 10.796342 | 10.867296 | 10.861158 |
| CCDC45 | 0.008415317 | 0.14873204 | 1961.6498 | 10.005914 | 0.062298417 | 10.030484 | 9.935077 | 10.05218 |
| CCDC59 | 0.001531908 | 0.0541222 | 984.64966 | 10.993931 | 0.07054654 | 10.915989 | 11.053411 | 11.012393 |
| CCDC6 | 0.000967372 | 0.040681213 | 823.02264 | 11.247348 | 0.12742083 | 11.381104 | 11.233555 | 11.127385 |
| CCDC72 | 5.38998E-05 | 0.00640411 | 291.91354 | 12.657368 | 0.1514708 | 12.828079 | 12.604972 | 12.53905 |
| CCDC90B | 0.009818125 | 0.16368572 | 2092.5837 | 9.9132395 | 0.10726549 | 9.7902565 | 9.98747 | 9.961992 |
| CCNA2 | 0.006294056 | 0.12622312 | 1737.9642 | 10.174048 | 0.052400507 | 10.114023 | 10.210658 | 10.197465 |
| CCNB2 | 0.001926789 | 0.06201113 | 1076.7948 | 10.862801 | 0.10198978 | 10.882709 | 10.953369 | 10.752325 |
| CCND1 | 6.32098E-05 | 0.00712013 | 308.04816 | 12.588302 | 0.060052782 | 12.652684 | 12.533806 | 12.578414 |
| CCND3 | 0.002337263 | 0.0698441 | 1161.3776 | 10.755826 | 0.0392704 | 10.798802 | 10.72181 | 10.746865 |
| CCNF | 0.008272208 | 0.14747997 | 1947.5983 | 10.015466 | 0.0510939 | 9.9618025 | 10.021065 | 10.063529 |
| CCNI | 8.19738E-05 | 0.008439169 | 337.17673 | 12.478917 | 0.042992003 | 12.455257 | 12.452954 | 12.528543 |
| CCNK | 0.005122702 | 0.1112184 | 1595.6564 | 10.303275 | 0.1345895 | 10.430761 | 10.1625595 | 10.316505 |
| CCT2 | 0.000626333 | 0.03153846 | 699.9795 | 11.452309 | 0.005335108 | 11.457162 | 11.453169 | 11.446596 |
| CCT3 | 0.00164037 | 0.05629179 | 1010.578 | 10.954373 | 0.069365345 | 10.963459 | 11.01875 | 10.880915 |
| CCT7 | 0.0017288 | 0.058402143 | 1031.8726 | 10.921437 | 0.06472619 | 10.907432 | 10.99202 | 10.864861 |
| CCT8 | 0.000562259 | 0.02928979 | 672.3767 | 11.507401 | 0.09511782 | 11.400425 | 11.582439 | 11.539341 |
| CD24 | 0.001104571 | 0.044457078 | 866.26605 | 11.184547 | 0.023735434 | 11.161371 | 11.183465 | 11.208805 |
| CD2BP2 | 0.000622759 | 0.03144978 | 698.45074 | 11.461305 | 0.15479335 | 11.594945 | 11.497277 | 11.291693 |
| CD63 | 0.000680031 | 0.03263209 | 721.325 | 11.416745 | 0.06324798 | 11.440153 | 11.34513 | 11.464952 |
| CD81 | 3.62022E-05 | 0.004944882 | 255.31189 | 12.820366 | 0.17540465 | 13.012367 | 12.7802105 | 12.668522 |
| CD9 | 0.005302992 | 0.1134291 | 1618.6945 | 10.277307 | 0.039053787 | 10.310342 | 10.287374 | 10.234205 |
| CDC20 | 0.000400069 | 0.023249581 | 592.7788 | 11.677341 | 0.056649618 | 11.625896 | 11.6680765 | 11.738053 |
| CDC23 | 0.005094224 | 0.11101696 | 1592.1549 | 10.303678 | 0.045854587 | 10.251483 | 10.322065 | 10.337484 |
| CDC26 | 0.0011193 | 0.04479008 | 870.7726 | 11.163455 | 0.2540398 | 11.438975 | 10.938502 | 11.112885 |
| CDC2L6 | 0.009183835 | 0.15657198 | 2035.2329 | 9.949575 | 0.019232424 | 9.964214 | 9.9277935 | 9.956718 |
| CDC37 | 8.62397E-05 | 0.008647399 | 342.8001 | 12.443664 | 0.15857159 | 12.262834 | 12.509168 | 12.558988 |
| CDC42EP4 | 0.003431717 | 0.08780236 | 1354.6897 | 10.532499 | 0.06327686 | 10.582338 | 10.4613085 | 10.55385 |
| CDC45L | 0.003293769 | 0.08579129 | 1331.9435 | 10.55342 | 0.05742905 | 10.4942 | 10.608872 | 10.557186 |
| CDCA5 | 0.002185046 | 0.06649824 | 1130.8733 | 10.794524 | 0.10026955 | 10.776455 | 10.902599 | 10.704517 |
| CDH1 | 0.000226235 | 0.016250517 | 482.20328 | 11.969228 | 0.10120792 | 12.070981 | 11.968128 | 11.868574 |
| CDK2AP1 | 0.000106589 | 0.009994594 | 368.29303 | 12.331456 | 0.038744986 | 12.368255 | 12.335095 | 12.291021 |
| CDK4 | 0.000743529 | 0.034579087 | 745.61847 | 11.375953 | 0.16924365 | 11.363249 | 11.551189 | 11.213418 |
| CDK7 | 0.004173603 | 0.09904172 | 1467.2356 | 10.419029 | 0.025515292 | 10.435384 | 10.432075 | 10.389628 |
| CDKN1B | 0.009428922 | 0.15918589 | 2057.5713 | 9.933736 | 0.08137711 | 9.989823 | 9.840402 | 9.9709835 |
| CDKN3 | 0.002763302 | 0.07787977 | 1241.1451 | 10.658952 | 0.032142658 | 10.677129 | 10.62184 | 10.677888 |
| CDT1 | 0.003338214 | 0.0863654 | 1339.4764 | 10.549443 | 0.19232738 | 10.567442 | 10.73214 | 10.34875 |
| CEBPB | 0.000467718 | 0.025716323 | 627.9799 | 11.604889 | 0.11333489 | 11.701332 | 11.633274 | 11.480059 |
| CEBPZ | 0.00176408 | 0.059076253 | 1040.0847 | 10.912206 | 0.032562807 | 10.874883 | 10.926917 | 10.934816 |
| CENPB | 0.000239984 | 0.016888438 | 493.17203 | 11.945746 | 0.09217199 | 12.036217 | 11.851962 | 11.949059 |
| CENPN | 0.000759382 | 0.035034575 | 751.49786 | 11.362709 | 0.031674724 | 11.330132 | 11.393396 | 11.364598 |
| CENTA1 | 0.002561509 | 0.073995836 | 1204.3351 | 10.701634 | 0.07670238 | 10.688857 | 10.632122 | 10.783922 |
| CEP55 | 0.007846919 | 0.14245997 | 1905.6747 | 10.047797 | 0.050808974 | 9.9900465 | 10.085627 | 10.067719 |
| CETN2 | 0.000310054 | 0.01992037 | 541.2652 | 11.803803 | 0.107921265 | 11.798433 | 11.91431 | 11.6986685 |
| CFDP1 | 0.003656626 | 0.091399856 | 1390.2135 | 10.493651 | 0.06649667 | 10.475142 | 10.567442 | 10.438371 |
| CFL1 | 2.71517E-05 | 0.004077922 | 231.79037 | 12.933255 | 0.05184107 | 12.874104 | 12.954872 | 12.970789 |
| CGN | 0.005161469 | 0.1116409 | 1600.7913 | 10.294663 | 0.09201237 | 10.291062 | 10.204506 | 10.388425 |
| CHCHD2 | 3.57987E-05 | 0.004909091 | 254.47844 | 12.821805 | 0.12309703 | 12.954872 | 12.798548 | 12.711996 |
| CHCHD4 | 0.008779962 | 0.15261123 | 1996.7456 | 9.976627 | 0.031668726 | 9.985488 | 9.941472 | 10.002922 |
| CHCHD9 | 0.000454459 | 0.0251869 | 621.74664 | 11.610822 | 0.065703265 | 11.597744 | 11.552642 | 11.682081 |
| CHMP2B | 0.007460483 | 0.13811846 | 1865.9816 | 10.07924 | 0.006294282 | 10.0778885 | 10.07373 | 10.0861 |
| CHMP4A | 0.004309247 | 0.10060902 | 1486.2852 | 10.405626 | 0.096718945 | 10.404045 | 10.503126 | 10.309708 |
| CHMP5 | 0.004214331 | 0.099735335 | 1472.8918 | 10.414645 | 0.061199844 | 10.354709 | 10.477035 | 10.412191 |
| CHP | 0.009470052 | 0.15964723 | 2061.1296 | 9.9305525 | 0.069889165 | 9.931715 | 9.999854 | 9.86009 |
| CHSY1 | 0.002974722 | 0.080818325 | 1279.0237 | 10.61975 | 0.15708682 | 10.587265 | 10.481444 | 10.790539 |
| CHURC1 | 0.000746498 | 0.034670684 | 746.74225 | 11.370355 | 0.039281446 | 11.327887 | 11.405386 | 11.377793 |
| CIB1 | 0.000147288 | 0.012463415 | 413.35165 | 12.183795 | 0.106630266 | 12.233832 | 12.256204 | 12.061348 |
| CIP29 | 0.001187295 | 0.046179373 | 890.89746 | 11.140923 | 0.057334315 | 11.166166 | 11.181304 | 11.075298 |
| CIRBP | 0.000407736 | 0.02349834 | 597.00116 | 11.657792 | 0.17070118 | 11.467331 | 11.796979 | 11.709069 |
| CIRH1A | 0.003325676 | 0.086363025 | 1337.5348 | 10.551025 | 0.015971558 | 10.559911 | 10.532587 | 10.560578 |
| CISD1 | 0.001033666 | 0.04259145 | 843.76105 | 11.216148 | 0.04438809 | 11.168869 | 11.222649 | 11.256928 |
| CITED2 | 0.008630282 | 0.15137462 | 1982.2903 | 9.990317 | 0.06965384 | 9.918325 | 10.05737 | 9.995256 |
| CKS1B | 0.000627918 | 0.031572465 | 700.6951 | 11.449172 | 0.060534034 | 11.511855 | 11.391045 | 11.444614 |
| CKS2 | 0.000204387 | 0.015315334 | 464.63132 | 12.021819 | 0.027109496 | 11.995529 | 12.0202465 | 12.04968 |
| CLCN7 | 0.004996339 | 0.10984981 | 1579.5707 | 10.31781 | 0.098185375 | 10.425121 | 10.232475 | 10.295833 |
| CLDN3 | 0.000602957 | 0.030763235 | 690.21814 | 11.474744 | 0.1222465 | 11.615896 | 11.402953 | 11.405386 |
| CLDN7 | 0.007003776 | 0.13380452 | 1817.4014 | 10.117203 | 0.10017186 | 10.0107 | 10.209535 | 10.131374 |
| CLIC1 | 0.000221018 | 0.016109243 | 478.50708 | 11.985092 | 0.048831638 | 11.931382 | 11.99708 | 12.026813 |
| CLINT1 | 0.007196893 | 0.1359962 | 1838.2102 | 10.098794 | 0.07084203 | 10.115588 | 10.159731 | 10.021065 |
| CLNS1A | 0.001236035 | 0.047072448 | 905.0041 | 11.118514 | 0.04444742 | 11.067405 | 11.148132 | 11.140002 |
| CNDP2 | 0.001133568 | 0.04499771 | 875.2687 | 11.162811 | 0.13233645 | 11.082899 | 11.315565 | 11.089968 |
| CNIH | 0.002252176 | 0.068241924 | 1144.9287 | 10.774441 | 0.046293423 | 10.736912 | 10.826171 | 10.760238 |
| CNIH4 | 0.0003611 | 0.022017574 | 571.66815 | 11.724724 | 0.113659635 | 11.788545 | 11.593496 | 11.792128 |
| CNOT2 | 0.007398484 | 0.13777938 | 1859.6147 | 10.081357 | 0.09742655 | 10.17344 | 9.979347 | 10.091287 |
| CNPY2 | 0.000620078 | 0.03140584 | 697.3355 | 11.456387 | 0.09290488 | 11.395765 | 11.410047 | 11.563346 |
| COBLL1 | 0.009200957 | 0.15655616 | 2036.736 | 9.950522 | 0.11018291 | 10.013336 | 10.014934 | 9.8232975 |
| COIL | 0.005776705 | 0.11943802 | 1677.0531 | 10.229619 | 0.034697585 | 10.261185 | 10.235208 | 10.192468 |
| COMMD1 | 0.00402914 | 0.096738406 | 1446.3104 | 10.4396 | 0.13273539 | 10.5922985 | 10.351803 | 10.374699 |
| COMMD2 | 0.008987808 | 0.15459742 | 2016.8236 | 9.964324 | 0.08460867 | 10.062018 | 9.916235 | 9.914721 |
| COMMD3 | 0.000845161 | 0.037116457 | 782.772 | 11.311627 | 0.14237857 | 11.197426 | 11.471149 | 11.266306 |
| COMMD7 | 0.005454229 | 0.115242995 | 1637.7876 | 10.261907 | 0.103423215 | 10.211224 | 10.380896 | 10.193602 |
| COPA | 0.001992189 | 0.06317824 | 1090.7006 | 10.846194 | 0.05430774 | 10.839526 | 10.795527 | 10.903527 |
| COPB1 | 0.006096299 | 0.12412265 | 1714.7358 | 10.195859 | 0.08421727 | 10.116136 | 10.283946 | 10.187495 |
| COPB2 | 0.006928461 | 0.13309856 | 1809.2039 | 10.121745 | 0.04749982 | 10.170623 | 10.1188545 | 10.075755 |
| COPE | 0.004225025 | 0.09978421 | 1474.3629 | 10.41378 | 0.035382718 | 10.4544525 | 10.396796 | 10.390091 |
| COPS3 | 0.001115178 | 0.044728324 | 869.46735 | 11.174102 | 0.10688634 | 11.112885 | 11.297522 | 11.1118965 |
| COPS5 | 0.001553641 | 0.05472284 | 990.12646 | 10.9841 | 0.036019012 | 11.009562 | 10.942888 | 10.999849 |
| COPS7A | 0.003874849 | 0.09480536 | 1423.6105 | 10.459769 | 0.16452074 | 10.625508 | 10.2964945 | 10.457306 |
| COQ10B | 0.003333689 | 0.08631269 | 1338.8306 | 10.54848 | 0.09322163 | 10.640375 | 10.551077 | 10.453986 |
| COQ2 | 0.004520234 | 0.10420266 | 1515.9325 | 10.370412 | 0.059769806 | 10.319062 | 10.35615 | 10.436022 |
| COQ5 | 0.004071857 | 0.09749413 | 1452.3523 | 10.433642 | 0.14797343 | 10.3549 | 10.341689 | 10.604337 |
| COX17 | 0.000186747 | 0.014334071 | 450.3737 | 12.058888 | 0.11835202 | 12.007732 | 12.194214 | 11.974719 |
| COX4I1 | 4.15057E-06 | 0.001125 | 126.02223 | 13.534785 | 0.11930756 | 13.424415 | 13.518571 | 13.661371 |
| COX5A | 2.6604E-05 | 0.004030568 | 229.38399 | 12.952945 | 0.12594374 | 12.910931 | 13.094524 | 12.853377 |
| COX5B | 5.35539E-05 | 0.006406897 | 291.28436 | 12.658843 | 0.071681045 | 12.576142 | 12.697266 | 12.703122 |
| COX6A1 | 0.000325128 | 0.020583943 | 550.7014 | 11.775945 | 0.050220095 | 11.792128 | 11.719628 | 11.816078 |
| COX6B1 | 7.09056E-05 | 0.007592592 | 321.38647 | 12.541828 | 0.059613544 | 12.498905 | 12.516686 | 12.609895 |
| COX6C | 3.17058E-07 | 0.000164179 | 63.130157 | 13.915133 | 0.05083942 | 13.870764 | 13.904028 | 13.970607 |
| COX7A2 | 1.3547E-05 | 0.002554348 | 185.29587 | 13.180697 | 0.13044734 | 13.068611 | 13.323884 | 13.149596 |
| COX7A2L | 0.000774601 | 0.035175394 | 757.2295 | 11.354854 | 0.025997104 | 11.332471 | 11.348721 | 11.3833685 |
| COX7B | 0.000415317 | 0.023738056 | 601.34424 | 11.662318 | 0.14100388 | 11.677277 | 11.795247 | 11.514432 |
| COX7C | 5.30351E-06 | 0.001286713 | 138.96022 | 13.435422 | 0.17023158 | 13.327295 | 13.631648 | 13.347322 |
| COX8A | 2.28858E-05 | 0.003609091 | 219.38217 | 12.992967 | 0.08711052 | 13.082068 | 12.988837 | 12.907994 |
| CPNE3 | 0.001146913 | 0.045217045 | 879.26086 | 11.1584015 | 0.050988387 | 11.1682005 | 11.203779 | 11.103225 |
| CPSF3 | 0.002562691 | 0.07396839 | 1204.5641 | 10.702201 | 0.06528782 | 10.771015 | 10.64113 | 10.694455 |
| CRABP2 | 0.000287283 | 0.01927853 | 525.292 | 11.85011 | 0.037364904 | 11.880371 | 11.861615 | 11.808347 |
| CRIP2 | 0.005201332 | 0.11222326 | 1605.8354 | 10.292469 | 0.06651981 | 10.21744 | 10.315745 | 10.344224 |
| CRKL | 0.004540901 | 0.10454015 | 1518.5542 | 10.372233 | 0.03699506 | 10.395617 | 10.329581 | 10.391502 |
| CRLS1 | 0.005426385 | 0.11486455 | 1634.3842 | 10.263759 | 0.057872273 | 10.25888 | 10.323916 | 10.208481 |
| CSDA | 0.000241396 | 0.01695344 | 494.25967 | 11.930797 | 0.077967584 | 11.917852 | 12.014426 | 11.860111 |
| CSE1L | 0.00413132 | 0.09850997 | 1461.1006 | 10.425159 | 0.04174076 | 10.434134 | 10.379662 | 10.461683 |
| CSK | 0.005026979 | 0.11031373 | 1583.6425 | 10.310191 | 0.17726181 | 10.501787 | 10.152024 | 10.276759 |
| CSNK1G2 | 0.002096443 | 0.0649991 | 1112.9777 | 10.812419 | 0.1230017 | 10.90716 | 10.673409 | 10.856684 |
| CSNK2B | 0.002462472 | 0.0719133 | 1185.7721 | 10.727496 | 0.05654028 | 10.720241 | 10.787314 | 10.674934 |
| CST3 | 0.000307287 | 0.01996442 | 539.44525 | 11.815418 | 0.14526036 | 11.892054 | 11.906313 | 11.647888 |
| CSTB | 0.000305125 | 0.019861164 | 537.83527 | 11.818255 | 0.09474472 | 11.866908 | 11.709069 | 11.87879 |
| CSTF2 | 0.006225774 | 0.125361 | 1729.9635 | 10.182739 | 0.062075064 | 10.165947 | 10.130788 | 10.251483 |
| CTDSP2 | 0.006874272 | 0.1324242 | 1803.2927 | 10.128169 | 0.05753777 | 10.1799965 | 10.138254 | 10.066255 |
| CTNNA1 | 0.000674987 | 0.032434903 | 719.5277 | 11.416583 | 0.05060491 | 11.457764 | 11.431894 | 11.36009 |
| CTSL1 | 0.008706779 | 0.15233132 | 1989.6549 | 9.98547 | 0.07981599 | 10.065367 | 9.985307 | 9.905735 |
| CTXN1 | 0.0023268 | 0.069651425 | 1159.2799 | 10.758228 | 0.020893896 | 10.73928 | 10.754769 | 10.780636 |
| CUEDC1 | 0.003257595 | 0.08523303 | 1325.9803 | 10.560214 | 0.089779325 | 10.543333 | 10.657235 | 10.480073 |
| CWC15 | 0.009932726 | 0.16441126 | 2102.8315 | 9.903962 | 0.024189766 | 9.892823 | 9.887349 | 9.931715 |
| CWF19L1 | 0.008277166 | 0.14749256 | 1948.1045 | 10.019057 | 0.08703278 | 10.033584 | 10.097912 | 9.925674 |
| CXCR7 | 0.004737419 | 0.106313065 | 1545.502 | 10.345277 | 0.037764415 | 10.328183 | 10.319082 | 10.388566 |
| CXORF26 | 0.003329221 | 0.08639043 | 1338.0997 | 10.551848 | 0.04915313 | 10.608053 | 10.530584 | 10.516907 |
| CXXC5 | 0.000248977 | 0.017310621 | 500.2904 | 11.927383 | 0.022408307 | 11.915073 | 11.91383 | 11.953249 |
| CYB5A | 0.008564247 | 0.15059707 | 1975.9465 | 9.994561 | 0.015711866 | 9.980485 | 9.991687 | 10.011512 |
| CYB5B | 0.000329192 | 0.020727769 | 553.3676 | 11.771312 | 0.09630595 | 11.881914 | 11.726016 | 11.706004 |
| CYBA | 0.000162939 | 0.013146511 | 427.86224 | 12.122329 | 0.08143824 | 12.108446 | 12.048724 | 12.209816 |
| CYC1 | 0.000313801 | 0.020086715 | 543.6992 | 11.795136 | 0.09632322 | 11.893795 | 11.790283 | 11.701332 |
| CYP1B1 | 0.001224679 | 0.046845645 | 901.763 | 11.117419 | 0.13694146 | 11.245464 | 10.973045 | 11.133746 |
| DAD1 | 0.000219231 | 0.016012631 | 476.84866 | 11.988026 | 0.0365885 | 11.998665 | 11.947297 | 12.018116 |
| DAP3 | 0.003737966 | 0.09269836 | 1402.4833 | 10.484222 | 0.12823267 | 10.613188 | 10.482745 | 10.356735 |
| DARS | 0.000296218 | 0.019575238 | 531.3976 | 11.835727 | 0.083709344 | 11.745675 | 11.850334 | 11.911171 |
| DARS2 | 0.009466882 | 0.15967137 | 2060.821 | 9.932239 | 0.082993455 | 9.852464 | 9.92614 | 10.018114 |
| DAZAP2 | 4.28316E-05 | 0.005586466 | 270.3738 | 12.750796 | 0.06693473 | 12.768198 | 12.807311 | 12.676879 |
| DBI | 0.000171961 | 0.013652174 | 436.12286 | 12.104149 | 0.03806666 | 12.145518 | 12.096329 | 12.0706 |
| DBNDD1 | 0.004875022 | 0.10821113 | 1563.6813 | 10.327685 | 0.12178949 | 10.449419 | 10.327797 | 10.20584 |
| DCAF7 | 0.001856661 | 0.06082625 | 1061.5732 | 10.8849745 | 0.074829556 | 10.889007 | 10.957706 | 10.80821 |
| DCAKD | 0.009992074 | 0.16507857 | 2108.3303 | 9.899399 | 0.14368217 | 9.773082 | 10.055711 | 9.869404 |
| DCP2 | 0.00804433 | 0.14505717 | 1925.3868 | 10.029244 | 0.102520585 | 10.146535 | 9.956718 | 9.98448 |
| DCTN2 | 0.001838128 | 0.060504746 | 1057.3802 | 10.892848 | 0.15448307 | 10.902599 | 10.73372 | 11.042224 |
| DCTN5 | 0.008440451 | 0.14902443 | 1964.1307 | 10.004071 | 0.08846587 | 9.942545 | 10.1054535 | 9.964214 |
| DCTPP1 | 0.000174065 | 0.013725 | 437.97876 | 12.097211 | 0.021593247 | 12.099109 | 12.074732 | 12.117793 |
| DCXR | 0.003650343 | 0.09137446 | 1389.1637 | 10.496502 | 0.076289885 | 10.505115 | 10.56812 | 10.416271 |
| DDA1 | 0.006625065 | 0.12934722 | 1775.5728 | 10.1497755 | 0.121960565 | 10.262844 | 10.165947 | 10.020536 |
| DDOST | 0.000298034 | 0.019583333 | 532.7698 | 11.83748 | 0.21196753 | 11.726016 | 11.7045 | 12.0819235 |
| DDT | 0.000640226 | 0.031596016 | 705.5233 | 11.440618 | 0.03150475 | 11.446596 | 11.468705 | 11.406552 |
| DDX1 | 0.001333718 | 0.049701396 | 932.3023 | 11.070457 | 0.11066532 | 11.185623 | 10.964922 | 11.060827 |
| DDX18 | 0.005047991 | 0.1104256 | 1586.3035 | 10.310444 | 0.08200021 | 10.402714 | 10.245905 | 10.282712 |
| DDX21 | 0.004749294 | 0.10644186 | 1547.0657 | 10.346605 | 0.029404873 | 10.376569 | 10.317793 | 10.345453 |
| DDX47 | 0.006609904 | 0.12919661 | 1773.8654 | 10.150788 | 0.12934227 | 10.011894 | 10.267782 | 10.17269 |
| DDX5 | 6.2518E-05 | 0.007111475 | 306.80347 | 12.588969 | 0.17774768 | 12.666124 | 12.385681 | 12.715102 |
| DDX55 | 0.00927757 | 0.15739657 | 2043.7086 | 9.944892 | 0.043012068 | 9.952659 | 9.898525 | 9.983491 |
| DECR1 | 0.000186689 | 0.014361419 | 450.3134 | 12.065201 | 0.0812092 | 12.115636 | 11.97152 | 12.108446 |
| DEK | 0.000787917 | 0.035547465 | 762.382 | 11.343358 | 0.22581087 | 11.126315 | 11.32674 | 11.577019 |
| DERA | 0.003654321 | 0.091408074 | 1389.8069 | 10.496441 | 0.07156125 | 10.430086 | 10.572266 | 10.486971 |
| DERL1 | 0.00307791 | 0.08265093 | 1296.1448 | 10.590965 | 0.10774384 | 10.480779 | 10.696088 | 10.596028 |
| DEXI | 0.007149536 | 0.13539629 | 1832.9939 | 10.103828 | 0.124549225 | 10.16831 | 10.182916 | 9.9602585 |
| DHCR24 | 0.00877437 | 0.15259048 | 1996.1637 | 9.978661 | 0.1604828 | 10.010176 | 9.804758 | 10.121048 |
| DHRS7 | 0.002133597 | 0.065623224 | 1120.4598 | 10.807448 | 0.03187888 | 10.781396 | 10.797952 | 10.842996 |
| DHX15 | 0.001162218 | 0.04566478 | 883.9538 | 11.149677 | 0.05050945 | 11.20354 | 11.103373 | 11.14212 |
| DIMT1L | 0.003167551 | 0.08421073 | 1310.9814 | 10.575676 | 0.054877363 | 10.528462 | 10.635884 | 10.56268 |
| DKC1 | 0.001029775 | 0.042481568 | 842.6184 | 11.219893 | 0.051906385 | 11.235731 | 11.161912 | 11.262034 |
| DLD | 0.004676572 | 0.105837576 | 1537.094 | 10.357257 | 0.07632828 | 10.429458 | 10.277381 | 10.364931 |
| DNAJA1 | 5.50527E-05 | 0.006518771 | 294.2735 | 12.647235 | 0.030333605 | 12.625765 | 12.681937 | 12.634005 |
| DNAJA2 | 0.004432524 | 0.10265821 | 1503.5101 | 10.385849 | 0.018756172 | 10.398948 | 10.364363 | 10.394237 |
| DNAJA3 | 0.001610423 | 0.055872 | 1003.4574 | 10.96543 | 0.070162475 | 10.990185 | 10.886245 | 11.019858 |
| DNAJB11 | 0.00097158 | 0.040808715 | 824.4335 | 11.245087 | 0.11603886 | 11.314295 | 11.309844 | 11.111121 |
| DNAJC8 | 0.001005938 | 0.041947115 | 835.28906 | 11.227843 | 0.07781384 | 11.215687 | 11.156823 | 11.31102 |
| DNAJC9 | 0.000874878 | 0.038084064 | 793.0495 | 11.297772 | 0.039158966 | 11.305532 | 11.332471 | 11.255315 |
| DNCL1 | 6.93203E-05 | 0.007586751 | 318.68832 | 12.549526 | 0.06612717 | 12.580942 | 12.473547 | 12.594089 |
| DNLZ | 0.004394045 | 0.10217627 | 1498.0648 | 10.392428 | 0.06960103 | 10.472534 | 10.346757 | 10.357993 |
| DNMT1 | 0.000355912 | 0.021777777 | 568.6202 | 11.735424 | 0.14015862 | 11.770127 | 11.854971 | 11.581174 |
| DPM1 | 0.000293077 | 0.019478926 | 529.1001 | 11.840584 | 0.1676847 | 11.74199 | 11.745562 | 12.034198 |
| DPM3 | 0.007710267 | 0.14086361 | 1891.7448 | 10.061395 | 0.030961115 | 10.032546 | 10.094106 | 10.057531 |
| DPY30 | 0.009207903 | 0.15659755 | 2037.3275 | 9.947246 | 0.030424817 | 9.975776 | 9.950735 | 9.915227 |
| DPYSL2 | 0.006414712 | 0.12753697 | 1751.8444 | 10.16423 | 0.09839557 | 10.199209 | 10.053124 | 10.240357 |
| DRAP1 | 0.006317 | 0.12631816 | 1740.6436 | 10.177358 | 0.029022017 | 10.198633 | 10.144297 | 10.189142 |
| DSTN | 0.002537471 | 0.07360786 | 1199.7024 | 10.706909 | 0.08994968 | 10.656978 | 10.653 | 10.810748 |
| DUSP19 | 0.002418055 | 0.07133673 | 1177.1421 | 10.734107 | 0.034565322 | 10.757877 | 10.694455 | 10.749989 |
| DUSP23 | 0.002443016 | 0.07164666 | 1181.9138 | 10.730508 | 0.021832023 | 10.715058 | 10.720982 | 10.755485 |
| DUSP3 | 0.006545454 | 0.12851612 | 1766.422 | 10.1561 | 0.024854772 | 10.184657 | 10.139346 | 10.144297 |
| DYNC1I2 | 0.00983072 | 0.16350287 | 2093.738 | 9.906798 | 0.06181927 | 9.95621 | 9.926707 | 9.837478 |
| DYNC1LI2 | 0.002367845 | 0.07039417 | 1167.5382 | 10.746109 | 0.053110085 | 10.801261 | 10.695309 | 10.7417555 |
| DYNLL1 | 0.000379374 | 0.022732297 | 581.8088 | 11.705634 | 0.029011898 | 11.672757 | 11.716503 | 11.727642 |
| DYNLL2 | 0.009430103 | 0.1591284 | 2057.6746 | 9.934556 | 0.029751461 | 9.967301 | 9.909185 | 9.927182 |
| DYNLRB1 | 0.008372139 | 0.14834678 | 1957.2103 | 10.008595 | 0.096261665 | 9.974281 | 10.117312 | 9.934193 |
| DYNLT1 | 0.002101689 | 0.0650455 | 1114.0931 | 10.8197975 | 0.03446364 | 10.848038 | 10.781396 | 10.829961 |
| E4F1 | 0.007768404 | 0.14140451 | 1897.5822 | 10.047968 | 0.13190818 | 9.956718 | 9.987975 | 10.199209 |
| EAPP | 0.007358275 | 0.13732545 | 1855.292 | 10.088013 | 0.014913307 | 10.076807 | 10.104939 | 10.082292 |
| EBNA1BP2 | 0.000579985 | 0.029988078 | 680.1881 | 11.4921255 | 0.08889893 | 11.39465 | 11.568737 | 11.51299 |
| ECH1 | 0.004689053 | 0.10591276 | 1538.7983 | 10.349514 | 0.08315258 | 10.328942 | 10.441022 | 10.278579 |
| ECHS1 | 0.000550038 | 0.028869893 | 667.23236 | 11.516019 | 0.024601184 | 11.539341 | 11.518407 | 11.490313 |
| ECT2 | 0.004719635 | 0.10611989 | 1543.1023 | 10.349227 | 0.100909926 | 10.233046 | 10.399613 | 10.41502 |
| EDF1 | 0.000270911 | 0.01846562 | 514.2954 | 11.883293 | 0.03345606 | 11.885177 | 11.915768 | 11.848935 |
| EEF1A1 | 4.93169E-05 | 0.006003509 | 283.81497 | 12.695236 | 0.056406744 | 12.754637 | 12.642397 | 12.688677 |
| EEF1A2 | 2.53646E-06 | 0.000838095 | 105.2982 | 13.654568 | 0.14593734 | 13.813509 | 13.52661 | 13.623584 |
| EEF1AL7 | 1.14141E-05 | 0.00231579 | 175.57715 | 13.214768 | 0.102359116 | 13.217205 | 13.111213 | 13.315887 |
| EEF1B2 | 0.003966969 | 0.09624475 | 1437.194 | 10.450059 | 0.036423497 | 10.414245 | 10.448868 | 10.487062 |
| EEF1G | 5.95204E-05 | 0.006883333 | 301.92197 | 12.615677 | 0.08453091 | 12.594089 | 12.708908 | 12.544033 |
| EEF2 | 1.1587E-05 | 0.002323699 | 176.15036 | 13.210629 | 0.093277454 | 13.209437 | 13.117955 | 13.304499 |
| EFHA1 | 0.003183375 | 0.0843728 | 1313.8187 | 10.572625 | 0.048605338 | 10.616146 | 10.581556 | 10.520174 |
| EFR3A | 0.008165965 | 0.14641343 | 1937.2599 | 10.023496 | 0.06267666 | 9.958721 | 10.0279255 | 10.083839 |
| EHD1 | 0.005745287 | 0.119143456 | 1673.256 | 10.230901 | 0.048669536 | 10.282127 | 10.185272 | 10.225305 |
| EIF1 | 0.000755404 | 0.034944 | 750.0043 | 11.364463 | 0.10553643 | 11.478706 | 11.270609 | 11.344073 |
| EIF1B | 0.002313599 | 0.069556326 | 1156.6151 | 10.762216 | 0.045362778 | 10.762733 | 10.807318 | 10.716597 |
| EIF2A | 0.004340088 | 0.10112491 | 1490.4679 | 10.396654 | 0.11889634 | 10.5229 | 10.380253 | 10.28681 |
| EIF2AK2 | 0.00155511 | 0.054719068 | 990.4651 | 10.989471 | 0.11762488 | 10.8540745 | 11.047889 | 11.066454 |
| EIF2B2 | 0.006129302 | 0.124356724 | 1718.7001 | 10.193272 | 0.10717204 | 10.069905 | 10.246505 | 10.263405 |
| EIF2S3 | 0.004544619 | 0.10448708 | 1519.0742 | 10.366164 | 0.18421161 | 10.278579 | 10.577827 | 10.242086 |
| EIF3A | 0.00610633 | 0.124108374 | 1715.9387 | 10.195618 | 0.08499297 | 10.218025 | 10.1016655 | 10.267161 |
| EIF3B | 0.003696259 | 0.09192688 | 1396.0852 | 10.489512 | 0.057025418 | 10.502158 | 10.427226 | 10.539154 |
| EIF3D | 0.006502854 | 0.1281875 | 1761.7213 | 10.161645 | 0.088956505 | 10.204004 | 10.221506 | 10.059425 |
| EIF3E | 3.54528E-06 | 0.001042373 | 118.24101 | 13.592673 | 0.038131304 | 13.598944 | 13.551795 | 13.62728 |
| EIF3F | 0.00315729 | 0.08406677 | 1309.1317 | 10.582693 | 0.20949139 | 10.35615 | 10.622513 | 10.769417 |
| EIF3G | 0.003122586 | 0.08339877 | 1303.6384 | 10.582219 | 0.015768977 | 10.595308 | 10.586637 | 10.564713 |
| EIF3H | 0.000666282 | 0.032239888 | 715.938 | 11.423015 | 0.17948785 | 11.3512535 | 11.290509 | 11.62728 |
| EIF3I | 0.001264686 | 0.047900654 | 912.91705 | 11.094345 | 0.29230398 | 10.823328 | 11.404078 | 11.055631 |
| EIF3K | 0.000352856 | 0.021744227 | 566.6778 | 11.740864 | 0.08724163 | 11.836325 | 11.721001 | 11.665267 |
| EIF3L | 0.000422667 | 0.024 | 605.379 | 11.650037 | 0.102493644 | 11.707879 | 11.710534 | 11.531697 |
| EIF3M | 0.002278838 | 0.0688094 | 1150.0061 | 10.76931 | 0.057846915 | 10.7131815 | 10.828734 | 10.766014 |
| EIF4A1 | 5.77045E-05 | 0.006740741 | 298.90573 | 12.629977 | 0.035360195 | 12.59784 | 12.667857 | 12.624231 |
| EIF4A2 | 0.000231135 | 0.016432377 | 486.17035 | 11.964898 | 0.0399772 | 11.980066 | 11.919557 | 11.995071 |
| EIF4A3 | 0.000302617 | 0.019809434 | 535.99194 | 11.816585 | 0.018859062 | 11.808347 | 11.838161 | 11.8032465 |
| EIF4B | 0.000809074 | 0.036266148 | 770.0162 | 11.334198 | 0.11804194 | 11.323647 | 11.457162 | 11.2217865 |
| EIF4E2 | 0.00305341 | 0.08243969 | 1292.2992 | 10.602059 | 0.179117 | 10.428791 | 10.786502 | 10.590886 |
| EIF4G2 | 0.001945524 | 0.06221014 | 1080.8274 | 10.860335 | 0.048852444 | 10.842069 | 10.9156885 | 10.823248 |
| EIF4H | 0.002086643 | 0.06486917 | 1110.7622 | 10.817261 | 0.091726705 | 10.715924 | 10.841245 | 10.894612 |
| ELF1 | 0.00276973 | 0.07793431 | 1242.1593 | 10.663376 | 0.13819936 | 10.770195 | 10.507296 | 10.712638 |
| ELP2 | 0.003285669 | 0.08570902 | 1330.6416 | 10.558536 | 0.002978261 | 10.560578 | 10.555119 | 10.559911 |
| EMD | 0.00092503 | 0.039572135 | 809.91943 | 11.268474 | 0.062354486 | 11.340463 | 11.2314005 | 11.233555 |
| ENO1 | 8.67585E-06 | 0.001869565 | 160.28848 | 13.318497 | 0.018097017 | 13.297683 | 13.327295 | 13.330512 |
| ENOPH1 | 0.003365078 | 0.086801484 | 1343.7449 | 10.547345 | 0.080891 | 10.463978 | 10.552552 | 10.625508 |
| ENY2 | 0.000149132 | 0.012558253 | 415.219 | 12.163951 | 0.07104262 | 12.0819235 | 12.2058115 | 12.204117 |
| EPCAM | 0.000166974 | 0.013347927 | 431.7407 | 12.121697 | 0.10483261 | 12.074543 | 12.241827 | 12.048724 |
| EPN1 | 0.001100969 | 0.044363532 | 865.1917 | 11.184825 | 0.056357168 | 11.120104 | 11.2230625 | 11.211308 |
| EPRS | 0.002437597 | 0.07154822 | 1180.8312 | 10.729663 | 0.14366068 | 10.85497 | 10.7611475 | 10.572871 |
| ERGIC3 | 0.004408428 | 0.10230502 | 1500.0597 | 10.390837 | 0.053568542 | 10.430185 | 10.412495 | 10.32983 |
| ERH | 0.000309477 | 0.019920222 | 540.81165 | 11.803108 | 0.06204642 | 11.822094 | 11.853444 | 11.733788 |
| ERP29 | 0.002535078 | 0.0736 | 1199.2621 | 10.709611 | 0.021260088 | 10.68753 | 10.729941 | 10.711363 |
| ESD | 0.004782066 | 0.106900126 | 1551.4873 | 10.337628 | 0.10633389 | 10.36209 | 10.4296 | 10.221195 |
| ESR1 | 0.00289834 | 0.079615995 | 1265.5834 | 10.626856 | 0.06125511 | 10.695309 | 10.577207 | 10.608053 |
| ESYT1 | 0.001953623 | 0.062354185 | 1082.4958 | 10.852356 | 0.107965924 | 10.753987 | 10.967868 | 10.835213 |
| ETFA | 0.000266069 | 0.018279208 | 511.3437 | 11.888139 | 0.056830335 | 11.952528 | 11.84498 | 11.866908 |
| EVL | 0.000206001 | 0.015403017 | 465.82278 | 12.018796 | 0.14344564 | 12.180334 | 11.969742 | 11.906313 |
| EWSR1 | 0.007244625 | 0.13637818 | 1843.3944 | 10.096858 | 0.0523996 | 10.039206 | 10.109781 | 10.141586 |
| EXOSC3 | 0.003986914 | 0.09639164 | 1440.1381 | 10.445777 | 0.038657732 | 10.475231 | 10.402002 | 10.460097 |
| EXOSC6 | 0.003403989 | 0.08754485 | 1349.992 | 10.535201 | 0.09235056 | 10.610266 | 10.563263 | 10.432075 |
| EXOSC8 | 0.00895371 | 0.15424033 | 2013.7131 | 9.964486 | 0.2216347 | 10.207909 | 9.911196 | 9.774356 |
| EZR | 0.000333458 | 0.020882672 | 555.519 | 11.767148 | 0.06148816 | 11.711818 | 11.756283 | 11.833345 |
| F2R | 0.002915461 | 0.07983346 | 1268.4894 | 10.621985 | 0.061457213 | 10.563404 | 10.685963 | 10.61659 |
| FADD | 0.005578054 | 0.11721684 | 1653.1854 | 10.2474785 | 0.07302909 | 10.212371 | 10.331431 | 10.198633 |
| FAF2 | 0.001131925 | 0.044983964 | 874.76996 | 11.162511 | 0.07390667 | 11.163865 | 11.235731 | 11.087936 |
| FAM102B | 0.005620886 | 0.11747651 | 1658.5046 | 10.245769 | 0.092020616 | 10.221506 | 10.16831 | 10.347489 |
| FAM108C1 | 0.003117744 | 0.08333359 | 1302.7974 | 10.585063 | 0.097588055 | 10.685748 | 10.578542 | 10.490899 |
| FAM115A | 0.008194933 | 0.1467811 | 1940.118 | 10.02424 | 0.04065372 | 10.025422 | 9.983008 | 10.06429 |
| FAM120A | 0.000193002 | 0.014716484 | 455.43372 | 12.048558 | 0.049651235 | 11.991236 | 12.078078 | 12.0763645 |
| FAM120AOS | 0.001420995 | 0.05167715 | 955.3178 | 11.038811 | 0.083866544 | 11.108135 | 10.945589 | 11.062707 |
| FAM120B | 0.008579812 | 0.15071797 | 1977.4539 | 9.9935 | 0.04676509 | 10.023301 | 9.9396 | 10.017596 |
| FAM127A | 0.003338848 | 0.086317435 | 1339.585 | 10.548875 | 0.074383244 | 10.632122 | 10.488938 | 10.525567 |
| FAM136A | 0.00304214 | 0.08219938 | 1290.2648 | 10.600063 | 0.016523195 | 10.585931 | 10.596028 | 10.61823 |
| FAM174B | 0.008855278 | 0.15330589 | 2004.235 | 9.974582 | 0.08933028 | 9.872127 | 10.036157 | 10.015463 |
| FAM175A | 0.009751369 | 0.16304289 | 2086.6575 | 9.915423 | 0.100336194 | 10.0279255 | 9.8351965 | 9.883149 |
| FAM177A1 | 0.007095492 | 0.13474056 | 1827.2052 | 10.108437 | 0.023188252 | 10.082972 | 10.114002 | 10.128335 |
| FAM20B | 0.00654018 | 0.12855807 | 1765.9296 | 10.150407 | 0.05557633 | 10.11832 | 10.11832 | 10.2145815 |
| FAM32A | 0.005138064 | 0.1114125 | 1597.786 | 10.2982025 | 0.0933423 | 10.225305 | 10.265897 | 10.403406 |
| FAM38A | 0.003899983 | 0.09521886 | 1427.49 | 10.456611 | 0.026449917 | 10.486241 | 10.435384 | 10.448206 |
| FAM39E | 0.002889232 | 0.07961795 | 1263.9354 | 10.632142 | 0.07185688 | 10.549529 | 10.66676 | 10.680137 |
| FAM3A | 0.002776388 | 0.07805835 | 1243.3534 | 10.652261 | 0.09195249 | 10.73372 | 10.670513 | 10.552552 |
| FAM44B | 0.001199343 | 0.04628476 | 894.2309 | 11.132989 | 0.14744765 | 11.046918 | 11.303244 | 11.048806 |
| FAM45A | 0.007596876 | 0.13960063 | 1880.0607 | 10.062786 | 0.11502438 | 9.979823 | 10.014444 | 10.194093 |
| FAM46A | 0.001464547 | 0.052599378 | 967.09015 | 11.019834 | 0.043093357 | 10.9799185 | 11.065523 | 11.014061 |
| FAM50A | 0.000552343 | 0.02894713 | 668.15857 | 11.512263 | 0.07733103 | 11.423639 | 11.566037 | 11.547111 |
| FAM53C | 0.009218366 | 0.15662193 | 2038.3354 | 9.950493 | 0.08049512 | 10.021622 | 9.8631115 | 9.966744 |
| FAM82A2 | 0.008729694 | 0.1522715 | 1991.8378 | 9.984093 | 0.08792985 | 9.920283 | 10.084393 | 9.947601 |
| FAM84B | 0.003289503 | 0.08574455 | 1331.2969 | 10.553792 | 0.039617036 | 10.510721 | 10.588676 | 10.561977 |
| FAM96A | 0.00229521 | 0.06912326 | 1152.9994 | 10.769218 | 0.06113513 | 10.708446 | 10.768498 | 10.830709 |
| FAM96B | 0.005973338 | 0.122409336 | 1700.5089 | 10.211537 | 0.14102194 | 10.372815 | 10.111424 | 10.150372 |
| FAM98A | 0.004650228 | 0.106071666 | 1533.5369 | 10.358875 | 0.09684053 | 10.4666395 | 10.279143 | 10.330842 |
| FANCI | 0.009167781 | 0.15660611 | 2033.7909 | 9.950753 | 0.02600089 | 9.954192 | 9.923204 | 9.974864 |
| FARSLB | 2.99764E-06 | 0.000920354 | 113.002686 | 13.614718 | 0.09711105 | 13.631648 | 13.510255 | 13.7022505 |
| FASN | 0.00046057 | 0.025403816 | 624.68365 | 11.602038 | 0.17842421 | 11.604357 | 11.422466 | 11.779292 |
| FAU | 6.22586E-06 | 0.001430464 | 147.04205 | 13.406593 | 0.06849626 | 13.485685 | 13.367046 | 13.367046 |
| FBL | 0.000523261 | 0.028015433 | 654.552 | 11.540489 | 0.035590254 | 11.535501 | 11.578312 | 11.507658 |
| FBP1 | 0.000915951 | 0.03932921 | 806.89014 | 11.271855 | 0.1301263 | 11.134576 | 11.287598 | 11.393396 |
| FBXO21 | 0.009041045 | 0.15512858 | 2021.8141 | 9.960134 | 0.05305586 | 9.901274 | 10.00428 | 9.974846 |
| FDFT1 | 0.002853462 | 0.07926181 | 1257.2336 | 10.639926 | 0.05940353 | 10.691901 | 10.575173 | 10.652702 |
| FEN1 | 0.005974261 | 0.12235596 | 1700.6265 | 10.211207 | 0.043245826 | 10.212087 | 10.254006 | 10.167528 |
| FERMT2 | 0.007173632 | 0.13570447 | 1835.5784 | 10.101115 | 0.045257222 | 10.136563 | 10.116644 | 10.0501375 |
| FEZ2 | 0.008733355 | 0.1522588 | 1992.1982 | 9.983075 | 0.096820645 | 9.877969 | 10.0686245 | 10.002631 |
| FH | 0.002871765 | 0.07938884 | 1260.7527 | 10.64122 | 0.09839192 | 10.693655 | 10.527717 | 10.70229 |
| FIS1 | 0.004260161 | 0.100136854 | 1479.2152 | 10.407311 | 0.121926166 | 10.276759 | 10.42695 | 10.518228 |
| FKBP3 | 0.009533522 | 0.16056116 | 2066.9102 | 9.928251 | 0.0164253 | 9.945571 | 9.912897 | 9.926287 |
| FKBP4 | 0.002105378 | 0.06498577 | 1114.8666 | 10.8112755 | 0.16532545 | 10.912613 | 10.900716 | 10.620498 |
| FLJ35390 | 0.008725688 | 0.1523548 | 1991.4907 | 9.980201 | 0.27021816 | 10.07373 | 9.675643 | 10.191228 |
| FLJ40504 | 0.002217819 | 0.06731846 | 1137.7516 | 10.788288 | 0.05644721 | 10.838702 | 10.79886 | 10.727303 |
| FLJ43681 | 7.41339E-05 | 0.007865444 | 325.97354 | 12.526971 | 0.07009769 | 12.5488205 | 12.44855 | 12.583541 |
| FLJ44124 | 0.001523722 | 0.053942855 | 982.4779 | 10.99841 | 0.06004027 | 11.001833 | 10.936731 | 11.056665 |
| FLOT2 | 0.002004093 | 0.06332423 | 1093.1388 | 10.845459 | 0.097923845 | 10.958532 | 10.788923 | 10.788923 |
| FN3KRP | 0.002276071 | 0.06878571 | 1149.5334 | 10.771965 | 0.030824961 | 10.750703 | 10.757877 | 10.807318 |
| FOXA1 | 0.000163717 | 0.013148149 | 428.82428 | 12.127643 | 0.07889443 | 12.038035 | 12.186669 | 12.158221 |
| FOXC1 | 0.006692886 | 0.13030471 | 1783.2327 | 10.144055 | 0.048868917 | 10.200381 | 10.112932 | 10.1188545 |
| FSCN1 | 0.003465787 | 0.08834828 | 1360.1012 | 10.526553 | 0.10011727 | 10.61892 | 10.540575 | 10.420164 |
| FTH1 | 0.007861503 | 0.1426501 | 1907.1461 | 10.046006 | 0.037212186 | 10.088925 | 10.026343 | 10.022751 |
| FTHL11 | 0.000658241 | 0.03211955 | 712.7058 | 11.428731 | 0.09084465 | 11.446092 | 11.33046 | 11.509644 |
| FTHL12 | 3.41846E-05 | 0.004801619 | 250.30014 | 12.842813 | 0.1315821 | 12.941045 | 12.894081 | 12.693315 |
| FTHL16 | 0.001546435 | 0.054524392 | 988.39264 | 10.986506 | 0.047174186 | 10.940011 | 11.034331 | 10.985178 |
| FTHL2 | 0.006913616 | 0.13296065 | 1807.5529 | 10.124024 | 0.06789352 | 10.048292 | 10.1443405 | 10.1794405 |
| FTHL3 | 0.003742866 | 0.09268737 | 1403.1694 | 10.486911 | 0.1572107 | 10.404745 | 10.387809 | 10.668179 |
| FTHL7 | 0.00602499 | 0.12324941 | 1706.3612 | 10.206463 | 0.13999546 | 10.051401 | 10.244426 | 10.323562 |
| FTL | 6.53139E-05 | 0.00726282 | 311.74518 | 12.577397 | 0.066808976 | 12.612787 | 12.619067 | 12.500339 |
| GABARAPL2 | 0.007358102 | 0.13739613 | 1855.2734 | 10.085988 | 0.078793645 | 10.030675 | 10.051086 | 10.176206 |
| GALK1 | 0.000971753 | 0.040766627 | 824.5017 | 11.243423 | 0.1420411 | 11.406552 | 11.147119 | 11.176598 |
| GANAB | 0.008065054 | 0.14520447 | 1927.37 | 10.028805 | 0.15527707 | 10.155911 | 9.855735 | 10.074768 |
| GAPDH | 3.65769E-05 | 0.004937743 | 256.0555 | 12.803893 | 0.06262599 | 12.830575 | 12.848759 | 12.732345 |
| GAPVD1 | 0.006281864 | 0.12605147 | 1736.5317 | 10.182701 | 0.11188783 | 10.152024 | 10.306728 | 10.089353 |
| GARS | 0.001780971 | 0.059184864 | 1044.0178 | 10.907086 | 0.07508013 | 10.993144 | 10.873145 | 10.85497 |
| GATA3 | 0.001967055 | 0.06272519 | 1085.3671 | 10.854112 | 0.11910279 | 10.991239 | 10.794642 | 10.776455 |
| GATAD2A | 0.003666455 | 0.09157956 | 1391.7277 | 10.495542 | 0.03714153 | 10.4613085 | 10.535031 | 10.490284 |
| GCA | 0.005953277 | 0.12207033 | 1698.327 | 10.209321 | 0.012611447 | 10.208939 | 10.222119 | 10.196905 |
| GCN1L1 | 0.003500433 | 0.08871001 | 1365.5758 | 10.523442 | 0.013026599 | 10.533214 | 10.508654 | 10.528462 |
| GDI2 | 0.000655502 | 0.03212147 | 711.5739 | 11.438167 | 0.13398618 | 11.5906515 | 11.339261 | 11.384588 |
| GEMIN4 | 0.006526287 | 0.12843052 | 1764.4163 | 10.159276 | 0.116802625 | 10.112403 | 10.292234 | 10.073191 |
| GGCT | 0.002129792 | 0.06556433 | 1119.6808 | 10.805649 | 0.11957705 | 10.681522 | 10.815339 | 10.920087 |
| GHITM | 0.000141523 | 0.012123457 | 408.2394 | 12.192749 | 0.120414734 | 12.315257 | 12.074543 | 12.188444 |
| GINS2 | 0.000544878 | 0.028729483 | 665.09033 | 11.522723 | 0.047688417 | 11.568737 | 11.525912 | 11.47352 |
| GLA | 0.000974145 | 0.040817633 | 825.3926 | 11.243256 | 0.066257015 | 11.286516 | 11.276272 | 11.166977 |
| GLG1 | 0.001001557 | 0.04181468 | 833.9627 | 11.232547 | 0.011229784 | 11.227061 | 11.245464 | 11.225113 |
| GLO1 | 0.000511126 | 0.027535714 | 648.36554 | 11.560803 | 0.21361282 | 11.419982 | 11.806593 | 11.455838 |
| GLRX3 | 0.003237159 | 0.08514784 | 1322.5867 | 10.565902 | 0.038089618 | 10.525567 | 10.570882 | 10.601256 |
| GLRX5 | 7.03868E-05 | 0.007560371 | 320.38852 | 12.541016 | 0.06439976 | 12.615377 | 12.503834 | 12.503834 |
| GLTP | 0.001223583 | 0.04685541 | 901.4621 | 11.12265 | 0.03849946 | 11.160871 | 11.123201 | 11.083878 |
| GLTSCR2 | 7.42492E-05 | 0.007853659 | 326.18588 | 12.519867 | 0.10396675 | 12.639798 | 12.455257 | 12.464547 |
| GLUD1 | 0.005536606 | 0.11655764 | 1648.1677 | 10.252744 | 0.06362861 | 10.242707 | 10.320794 | 10.19473 |
| GMDS | 0.00754825 | 0.13914931 | 1874.9916 | 10.0710535 | 0.07106242 | 10.0420885 | 10.019049 | 10.152024 |
| GMFB | 0.001771373 | 0.059035543 | 1041.731 | 10.908498 | 0.038619027 | 10.905344 | 10.871554 | 10.948599 |
| GMPS | 0.006623047 | 0.12938063 | 1775.3026 | 10.150561 | 0.15689076 | 10.216877 | 10.263405 | 9.971402 |
| GNA13 | 0.004812763 | 0.107309766 | 1555.4841 | 10.339223 | 0.03869987 | 10.361873 | 10.2945385 | 10.36126 |
| GNB1 | 0.000439846 | 0.024772728 | 614.06195 | 11.627034 | 0.046001934 | 11.574018 | 11.656393 | 11.650691 |
| GNB2L1 | 2.88234E-08 | 2.43902E-05 | 39.616333 | 14.026203 | 0.11500538 | 14.104391 | 14.080067 | 13.894151 |
| GNL2 | 0.002291751 | 0.069079064 | 1152.3771 | 10.768996 | 0.059654355 | 10.835213 | 10.752325 | 10.719451 |
| GNS | 0.000548567 | 0.028880121 | 666.5105 | 11.519188 | 0.07393231 | 11.453169 | 11.599071 | 11.505323 |
| GOLPH3 | 0.007545224 | 0.13916746 | 1874.6797 | 10.070517 | 0.0896366 | 10.021065 | 10.016499 | 10.173986 |
| GORASP2 | 0.007629648 | 0.1400545 | 1883.5055 | 10.065483 | 0.1164443 | 9.994188 | 10.199858 | 10.002403 |
| GOT1 | 0.009346198 | 0.15825135 | 2050.0916 | 9.93842 | 0.039674703 | 9.970943 | 9.950101 | 9.894217 |
| GOT2 | 0.004303366 | 0.10060714 | 1485.5642 | 10.403278 | 0.04626277 | 10.4372 | 10.422057 | 10.350579 |
| GPD1L | 0.001626218 | 0.056139305 | 1007.22217 | 10.959259 | 0.079680964 | 11.007728 | 11.002753 | 10.867296 |
| GPI | 0.005352712 | 0.11400061 | 1625.222 | 10.271792 | 0.023593847 | 10.294053 | 10.274262 | 10.24706 |
| GPN1 | 0.006975154 | 0.13362563 | 1814.3123 | 10.118209 | 0.013101767 | 10.107711 | 10.132892 | 10.114023 |
| GPR137 | 0.004698132 | 0.10591098 | 1540.0332 | 10.351922 | 0.07005952 | 10.375313 | 10.407294 | 10.27316 |
| GPR172A | 0.006244365 | 0.1255168 | 1732.18 | 10.183918 | 0.062381435 | 10.155404 | 10.255461 | 10.140889 |
| GPX4 | 0.000216435 | 0.015908899 | 474.3155 | 11.999087 | 0.011376637 | 11.992233 | 11.992808 | 12.012219 |
| GSPT1 | 7.62091E-05 | 0.007987916 | 329.24786 | 12.506642 | 0.03750196 | 12.536487 | 12.464547 | 12.518894 |
| GSTO1 | 0.002104975 | 0.06503116 | 1114.8125 | 10.815193 | 0.08123324 | 10.766408 | 10.908967 | 10.770203 |
| GSTO2 | 0.00487727 | 0.10819182 | 1563.9508 | 10.330555 | 0.11672964 | 10.434011 | 10.204004 | 10.353648 |
| GTF2A2 | 0.001226235 | 0.046853524 | 902.2317 | 11.118941 | 0.08658002 | 11.190923 | 11.022866 | 11.143034 |
| GTF2E2 | 0.004609759 | 0.10535639 | 1528.0095 | 10.359084 | 0.051244773 | 10.300199 | 10.383488 | 10.393566 |
| GTF2F2 | 0.003784314 | 0.093115605 | 1409.5425 | 10.47531 | 0.111448266 | 10.507967 | 10.351181 | 10.566781 |
| GTF2H5 | 0.004800196 | 0.10709839 | 1553.8788 | 10.337728 | 0.08341859 | 10.24706 | 10.3549 | 10.411224 |
| GTF3A | 0.000409812 | 0.023578772 | 598.2861 | 11.661748 | 0.10179732 | 11.765486 | 11.657752 | 11.562009 |
| GTPBP4 | 0.000300167 | 0.0196862 | 534.2126 | 11.822711 | 0.05464733 | 11.816078 | 11.880371 | 11.771682 |
| GTPBP6 | 0.001210988 | 0.046475664 | 897.8944 | 11.124081 | 0.12753467 | 11.2684355 | 11.026677 | 11.077129 |
| GUK1 | 0.0031078 | 0.083195984 | 1301.1584 | 10.590535 | 0.20914951 | 10.830738 | 10.448742 | 10.492126 |
| GUSB | 0.009751687 | 0.16296965 | 2086.6758 | 9.914205 | 0.07893984 | 9.943999 | 9.824703 | 9.97391 |
| GYG1 | 0.002082349 | 0.06479372 | 1109.96 | 10.824139 | 0.014057741 | 10.840363 | 10.8164835 | 10.81557 |
| H1F0 | 0.00112567 | 0.044941314 | 872.82623 | 11.170522 | 0.038541596 | 11.163007 | 11.136291 | 11.212267 |
| H2AFJ | 0.004817432 | 0.10727599 | 1556.1708 | 10.336286 | 0.038492467 | 10.314085 | 10.314038 | 10.380733 |
| H2AFY2 | 0.007517323 | 0.13872659 | 1871.8643 | 10.074089 | 0.13650906 | 10.07735 | 10.208939 | 9.935979 |
| H2AFZ | 5.36116E-06 | 0.001291667 | 139.82475 | 13.44177 | 0.06607433 | 13.444518 | 13.506428 | 13.374365 |
| H3F3A | 5.10175E-06 | 0.001282609 | 136.55655 | 13.476205 | 0.08586409 | 13.484191 | 13.386627 | 13.557797 |
| H3F3B | 0.007873408 | 0.14279142 | 1908.4142 | 10.048421 | 0.1156021 | 10.009573 | 10.178444 | 9.957248 |
| HACL1 | 0.007337926 | 0.13724098 | 1853.0779 | 10.09045 | 0.066399 | 10.055183 | 10.167042 | 10.049127 |
| HADH | 0.003757307 | 0.092648186 | 1405.4419 | 10.479664 | 0.11289636 | 10.467291 | 10.598237 | 10.373464 |
| HARS | 0.005596818 | 0.11725604 | 1655.5435 | 10.253277 | 0.17614526 | 10.389081 | 10.316505 | 10.054245 |
| HARS2 | 0.003557388 | 0.08962963 | 1374.7902 | 10.50837 | 0.064983875 | 10.488938 | 10.455319 | 10.580853 |
| HCFC1 | 0.001725832 | 0.058358673 | 1031.1356 | 10.923232 | 0.033453587 | 10.885319 | 10.948599 | 10.935778 |
| HDAC1 | 0.00027878 | 0.0188538 | 519.51587 | 11.861016 | 0.11148591 | 11.924504 | 11.926257 | 11.732287 |
| HDDC2 | 0.000156021 | 0.012827015 | 421.61487 | 12.149094 | 0.023790756 | 12.132607 | 12.176367 | 12.138306 |
| HDGFRP3 | 0.004583444 | 0.10517064 | 1524.1788 | 10.367848 | 0.049167786 | 10.383488 | 10.312763 | 10.407294 |
| HEATR6 | 0.000646855 | 0.031787537 | 708.1544 | 11.437699 | 0.08874588 | 11.4622555 | 11.511581 | 11.339261 |
| HEBP1 | 0.002897446 | 0.079654515 | 1265.422 | 10.632115 | 0.12030204 | 10.732998 | 10.498972 | 10.664375 |
| HEBP2 | 0.000381449 | 0.022738831 | 582.94464 | 11.699 | 0.10556924 | 11.800019 | 11.707578 | 11.589404 |
| HECTD1 | 0.008134836 | 0.14608178 | 1934.2677 | 10.0258 | 0.07756734 | 9.987975 | 10.115024 | 9.974401 |
| HES4 | 0.00025771 | 0.017775347 | 506.20377 | 11.896956 | 0.12949786 | 11.995071 | 11.945621 | 11.750175 |
| HES6 | 0.003517121 | 0.089002915 | 1368.1788 | 10.519312 | 0.13532345 | 10.674189 | 10.42392 | 10.459827 |
| HEXB | 0.00053845 | 0.028477134 | 661.999 | 11.533592 | 0.19616902 | 11.589404 | 11.695807 | 11.315565 |
| HGS | 0.00262763 | 0.07527911 | 1217.0372 | 10.683595 | 0.073981166 | 10.765289 | 10.664375 | 10.62112 |
| HIATL2 | 0.002917968 | 0.07971339 | 1268.9314 | 10.6313715 | 0.14389524 | 10.539055 | 10.557887 | 10.797171 |
| HIBADH | 0.004888742 | 0.108169645 | 1565.481 | 10.328411 | 0.09665256 | 10.226519 | 10.418793 | 10.339923 |
| HIGD1A | 0.003168819 | 0.08417994 | 1311.2192 | 10.577411 | 0.022832725 | 10.603312 | 10.568722 | 10.560197 |
| HIGD2A | 0.000638439 | 0.03159772 | 704.7703 | 11.439598 | 0.03694163 | 11.468705 | 11.39804 | 11.45205 |
| HINT1 | 4.35234E-05 | 0.005592593 | 271.89694 | 12.747105 | 0.09223022 | 12.642296 | 12.783144 | 12.815873 |
| HINT2 | 0.007946648 | 0.14381899 | 1915.6552 | 10.041943 | 0.006832446 | 10.045888 | 10.034054 | 10.045888 |
| HIRIP3 | 0.007054217 | 0.13432437 | 1822.7194 | 10.107497 | 0.05129675 | 10.127964 | 10.049127 | 10.145401 |
| HIST1H1C | 0.00323223 | 0.0850827 | 1321.8429 | 10.56845 | 0.09813843 | 10.68177 | 10.511426 | 10.512156 |
| HIST1H2BK | 0.001165216 | 0.04573077 | 884.79767 | 11.146545 | 0.089016974 | 11.248498 | 11.106893 | 11.084245 |
| HIST1H4C | 3.51646E-06 | 0.00106087 | 117.49541 | 13.579679 | 0.07074712 | 13.661371 | 13.538834 | 13.538834 |
| HIST2H2AA3 | 0.000510319 | 0.027534992 | 647.94446 | 11.550103 | 0.068814896 | 11.485904 | 11.622753 | 11.541654 |
| HIST2H2AA4 | 0.00069009 | 0.0329326 | 725.3071 | 11.405677 | 0.04362469 | 11.455838 | 11.384588 | 11.376602 |
| HIST2H2AC | 0.000845766 | 0.03709608 | 782.96893 | 11.311306 | 0.093686394 | 11.203779 | 11.375345 | 11.3547945 |
| HIST2H2BE | 0.004659855 | 0.10580432 | 1534.8164 | 10.355256 | 0.16714224 | 10.548198 | 10.262844 | 10.2547245 |
| HLA-A | 0.004264311 | 0.10009878 | 1479.8768 | 10.408255 | 0.10687932 | 10.510943 | 10.297627 | 10.416193 |
| HLA-E | 0.006448464 | 0.12798798 | 1755.679 | 10.166308 | 0.19039442 | 10.282712 | 10.269623 | 9.946589 |
| HMG20B | 0.007652794 | 0.14003481 | 1885.8484 | 10.062266 | 0.10971041 | 10.179014 | 10.046479 | 9.961304 |
| HMGB1L1 | 0.008049403 | 0.14507325 | 1925.8588 | 10.032683 | 0.07726067 | 10.010036 | 9.969276 | 10.118736 |
| HMGB2 | 0.0008628 | 0.03765283 | 789.03424 | 11.303983 | 0.09166988 | 11.406475 | 11.229825 | 11.275651 |
| HMGCR | 0.007630542 | 0.13999683 | 1883.6001 | 10.064126 | 0.04899534 | 10.078423 | 10.009573 | 10.104383 |
| HMGCS1 | 0.005770566 | 0.11938223 | 1676.3334 | 10.2278595 | 0.06282087 | 10.289274 | 10.163721 | 10.230583 |
| HMGN1 | 1.49017E-05 | 0.002706806 | 190.81343 | 13.15533 | 0.056919657 | 13.172901 | 13.091696 | 13.201391 |
| HMGN4 | 0.005884735 | 0.12116617 | 1689.6721 | 10.218395 | 0.08853914 | 10.183513 | 10.15261 | 10.319062 |
| HNRNPA0 | 0.003622356 | 0.09073935 | 1384.976 | 10.503045 | 0.117421836 | 10.57933 | 10.561977 | 10.367829 |
| HNRNPA2B1 | 0.003885081 | 0.09492183 | 1425.1989 | 10.461545 | 0.03699381 | 10.458806 | 10.425997 | 10.499832 |
| HNRNPAB | 0.000591918 | 0.030423705 | 685.4596 | 11.480428 | 0.04402686 | 11.463334 | 11.530438 | 11.447512 |
| HNRNPD | 7.17992E-05 | 0.007664615 | 322.6039 | 12.530541 | 0.03009627 | 12.50941 | 12.565001 | 12.517215 |
| HNRNPH1 | 0.000615063 | 0.031243045 | 695.3732 | 11.467628 | 0.12524588 | 11.443928 | 11.603029 | 11.355924 |
| HNRNPM | 0.00134689 | 0.049924146 | 935.9218 | 11.072089 | 0.09952504 | 11.077006 | 11.1690645 | 10.970197 |
| HNRNPUL2 | 0.004310919 | 0.100580364 | 1486.5138 | 10.401706 | 0.10110771 | 10.496265 | 10.295125 | 10.413729 |
| HNRPA1L-2 | 0.0017663 | 0.05909354 | 1040.67 | 10.916846 | 0.13482751 | 10.879347 | 11.066454 | 10.804738 |
| HNRPA1P4 | 0.000309016 | 0.019964619 | 540.4703 | 11.8114195 | 0.19369233 | 11.978403 | 11.856783 | 11.599071 |
| HNRPA2B1 | 2.14446E-05 | 0.003476636 | 214.17514 | 13.018559 | 0.1497314 | 13.16644 | 13.022198 | 12.8670435 |
| HNRPC | 0.004151496 | 0.09871967 | 1463.9962 | 10.423171 | 0.13012005 | 10.568843 | 10.382212 | 10.3184595 |
| HNRPK | 0.009600565 | 0.16114272 | 2072.868 | 9.9237795 | 0.047200702 | 9.978197 | 9.899201 | 9.893939 |
| HNRPR | 0.001394132 | 0.051183067 | 948.8456 | 11.048202 | 0.13406275 | 11.145997 | 11.103225 | 10.895381 |
| HNRPUL1 | 0.003418228 | 0.0877809 | 1352.2444 | 10.5326605 | 0.059774242 | 10.478277 | 10.59666 | 10.523044 |
| HPRT1 | 0.000757249 | 0.03498269 | 750.72345 | 11.364879 | 0.044237 | 11.3940115 | 11.386649 | 11.313975 |
| HPS6 | 0.006306393 | 0.12632447 | 1739.3868 | 10.179698 | 0.081113316 | 10.261704 | 10.177883 | 10.099508 |
| HS.127310 | 0.001843345 | 0.060561553 | 1058.5079 | 10.888717 | 0.047863834 | 10.836913 | 10.897937 | 10.931299 |
| HS.137971 | 0.004208105 | 0.09965597 | 1472.038 | 10.41709 | 0.054648668 | 10.418131 | 10.36193 | 10.471212 |
| HS.213061 | 3.15328E-05 | 0.004539419 | 244.30278 | 12.874499 | 0.13302606 | 13.002498 | 12.736959 | 12.884043 |
| HS.370359 | 0.006276561 | 0.12601794 | 1735.9368 | 10.180344 | 0.081848696 | 10.2684355 | 10.106648 | 10.165947 |
| HS.388347 | 0.001187698 | 0.046143338 | 890.9933 | 11.139735 | 0.10598182 | 11.05167 | 11.110175 | 11.257358 |
| HS.413494 | 0.001779876 | 0.05920518 | 1043.77 | 10.907928 | 0.034838043 | 10.922629 | 10.933008 | 10.868151 |
| HS.497591 | 0.007002652 | 0.13385674 | 1817.2882 | 10.11476 | 0.09931972 | 10.09401 | 10.2228155 | 10.027454 |
| HS.508682 | 2.92558E-05 | 0.004337607 | 237.79562 | 12.901158 | 0.05725567 | 12.957907 | 12.9021635 | 12.843409 |
| HS.567759 | 0.006350522 | 0.12684226 | 1744.4047 | 10.172211 | 0.13706818 | 10.31963 | 10.048617 | 10.148384 |
| HS.57079 | 0.000237102 | 0.016787754 | 490.92447 | 11.9509 | 0.037723225 | 11.959502 | 11.983578 | 11.909617 |
| HS.579631 | 0.001586874 | 0.055387326 | 997.6812 | 10.976302 | 0.12434747 | 11.091949 | 10.844779 | 10.992175 |
| HS3ST3A1 | 0.008718136 | 0.1522996 | 1990.7673 | 9.984069 | 0.063662924 | 9.966199 | 10.054757 | 9.931251 |
| HSBP1 | 0.004013287 | 0.09655825 | 1443.9182 | 10.4417925 | 0.16071668 | 10.611725 | 10.421419 | 10.292234 |
| HSD17B4 | 0.005236669 | 0.11263546 | 1610.153 | 10.286193 | 0.07297228 | 10.36317 | 10.218025 | 10.277381 |
| HSD17B7 | 0.003440595 | 0.087899856 | 1356.041 | 10.538636 | 0.15492207 | 10.387213 | 10.53186 | 10.696835 |
| HSP90AA1 | 0.000766732 | 0.035001315 | 754.287 | 11.356769 | 0.10331498 | 11.344053 | 11.2604 | 11.465853 |
| HSP90AB1 | 0.000637488 | 0.031686246 | 704.26013 | 11.445451 | 0.09715662 | 11.474665 | 11.52465 | 11.33704 |
| HSP90B1 | 0.000210757 | 0.015590618 | 469.46948 | 12.011883 | 0.07282345 | 12.087395 | 12.00617 | 11.942084 |
| HSPA1A | 0.000489278 | 0.026648352 | 638.32324 | 11.581235 | 0.08328981 | 11.665267 | 11.57973 | 11.498708 |
| HSPA1B | 0.001079351 | 0.043746494 | 858.5791 | 11.189739 | 0.1481195 | 11.164867 | 11.055631 | 11.348721 |
| HSPA8 | 0.000113507 | 0.010417989 | 376.2162 | 12.31133 | 0.042655878 | 12.265883 | 12.31761 | 12.350499 |
| HSPA9 | 0.000731106 | 0.034277026 | 740.9264 | 11.384938 | 0.020414585 | 11.407716 | 11.368292 | 11.378807 |
| HSPB1 | 4.03528E-07 | 0.000186667 | 68.45315 | 13.89371 | 0.06437875 | 13.916738 | 13.943408 | 13.820984 |
| HSPBL2 | 0.001778579 | 0.05921881 | 1043.4635 | 10.906372 | 0.072976075 | 10.9873 | 10.845572 | 10.886245 |
| HSPE1 | 0.000386695 | 0.022816326 | 585.64374 | 11.695838 | 0.27222583 | 11.988741 | 11.45058 | 11.648193 |
| HSPH1 | 0.000460915 | 0.02538254 | 624.86426 | 11.602798 | 0.10700731 | 11.562009 | 11.724202 | 11.522185 |
| HTATIP2 | 0.004613075 | 0.105362736 | 1528.5289 | 10.360878 | 0.120807104 | 10.351181 | 10.245212 | 10.486241 |
| IAH1 | 0.005729175 | 0.11895153 | 1671.3035 | 10.233981 | 0.02147223 | 10.231257 | 10.256685 | 10.214001 |
| IARS2 | 0.003779414 | 0.09306104 | 1408.7554 | 10.480952 | 0.04620957 | 10.427596 | 10.507967 | 10.507296 |
| ID2 | 0.001885917 | 0.061321463 | 1067.8108 | 10.873402 | 0.052968547 | 10.933577 | 10.833834 | 10.852794 |
| ID3 | 0.003947887 | 0.09605049 | 1434.4788 | 10.454495 | 0.094675936 | 10.465255 | 10.543333 | 10.3549 |
| IDH2 | 0.00720724 | 0.13611758 | 1839.3356 | 10.098788 | 0.03175984 | 10.072138 | 10.090297 | 10.133931 |
| IER3 | 0.000497031 | 0.026985915 | 641.7549 | 11.577365 | 0.11606724 | 11.623149 | 11.663557 | 11.445388 |
| IFI27 | 0.00013155 | 0.011583757 | 397.7677 | 12.224511 | 0.057605688 | 12.291021 | 12.192118 | 12.190395 |
| IFI6 | 8.69026E-05 | 0.008688761 | 343.83652 | 12.442833 | 0.063101046 | 12.370597 | 12.487211 | 12.47069 |
| IFITM1 | 0.001802271 | 0.059550475 | 1049.2197 | 10.899362 | 0.049431004 | 10.897937 | 10.850659 | 10.949491 |
| IFITM2 | 7.00698E-05 | 0.007596875 | 319.9969 | 12.545396 | 0.014070902 | 12.558988 | 12.546311 | 12.53089 |
| IFITM3 | 0.000164438 | 0.013175519 | 429.54395 | 12.129933 | 0.12505075 | 12.19635 | 11.985687 | 12.207762 |
| IGFBP5 | 0.003621404 | 0.09078107 | 1384.8535 | 10.499214 | 0.05447043 | 10.490104 | 10.449874 | 10.557666 |
| IKBKG | 0.008163285 | 0.14651681 | 1937.0037 | 10.021317 | 0.09848109 | 10.12446 | 10.011213 | 9.928277 |
| IL13RA1 | 0.004755635 | 0.10651517 | 1547.9106 | 10.340793 | 0.11971437 | 10.325813 | 10.467291 | 10.229272 |
| IL18 | 0.000102381 | 0.009731507 | 363.13638 | 12.366726 | 0.060639653 | 12.417167 | 12.299446 | 12.383563 |
| ILF2 | 0.000307575 | 0.019908583 | 539.59576 | 11.802101 | 0.026410198 | 11.777718 | 11.798433 | 11.830154 |
| IMMT | 0.004659423 | 0.10593316 | 1534.7538 | 10.353736 | 0.09697234 | 10.264036 | 10.45663 | 10.340542 |
| IMP3 | 0.000439038 | 0.024767479 | 613.5327 | 11.634369 | 0.08136547 | 11.675769 | 11.540629 | 11.68671 |
| IMP4 | 0.00044708 | 0.024937298 | 617.99945 | 11.623016 | 0.011183338 | 11.628736 | 11.630182 | 11.610129 |
| IMPDH2 | 0.001436646 | 0.052028183 | 959.6555 | 11.026893 | 0.086288355 | 10.946541 | 11.118092 | 11.016044 |
| INTS2 | 0.007222834 | 0.13626373 | 1840.9559 | 10.097565 | 0.007815924 | 10.097418 | 10.089824 | 10.1054535 |
| IRAK1 | 0.001856575 | 0.060880907 | 1061.5568 | 10.884835 | 0.09126008 | 10.797952 | 10.876636 | 10.9799185 |
| IRF9 | 0.001361071 | 0.050288606 | 939.5604 | 11.06183 | 0.10154805 | 11.085972 | 11.149131 | 10.950386 |
| IRS1 | 0.006664553 | 0.12989888 | 1780.0977 | 10.144204 | 0.050549135 | 10.096893 | 10.197465 | 10.138254 |
| IRX3 | 0.001751917 | 0.058896318 | 1037.2308 | 10.9163885 | 0.025811566 | 10.919885 | 10.940273 | 10.889007 |
| ISG15 | 2.89964E-05 | 0.004317597 | 237.13228 | 12.9014435 | 0.12555699 | 12.810084 | 12.84963 | 13.044615 |
| ISOC1 | 0.000309275 | 0.019944238 | 540.6458 | 11.805106 | 0.097136326 | 11.735313 | 11.916043 | 11.763962 |
| ITGAE | 0.007386753 | 0.1376348 | 1858.3092 | 10.086835 | 0.04746953 | 10.034622 | 10.098496 | 10.127388 |
| ITGB4BP | 0.003792587 | 0.09318697 | 1410.9976 | 10.476303 | 0.085673556 | 10.384762 | 10.554556 | 10.489593 |
| ITPR3 | 0.002840549 | 0.07915663 | 1254.9536 | 10.645755 | 0.14916503 | 10.802188 | 10.505115 | 10.629962 |
| JMJD8 | 0.000807488 | 0.036241915 | 769.37366 | 11.333477 | 0.11361808 | 11.244617 | 11.461495 | 11.294318 |
| JTB | 5.57733E-05 | 0.006559322 | 295.52817 | 12.637666 | 0.09822159 | 12.578414 | 12.583541 | 12.751044 |
| JUND | 8.11956E-05 | 0.008383929 | 335.98413 | 12.482772 | 0.01615274 | 12.471282 | 12.501241 | 12.475793 |
| KBTBD2 | 0.00351934 | 0.08899417 | 1368.6028 | 10.517607 | 0.045073032 | 10.56268 | 10.517606 | 10.472534 |
| KCTD3 | 0.00544806 | 0.11518282 | 1637.0295 | 10.263477 | 0.0259549 | 10.28399 | 10.234298 | 10.272143 |
| KDELR1 | 0.001395659 | 0.05118499 | 949.1464 | 11.04791 | 0.05991498 | 11.0328455 | 11.113919 | 10.996964 |
| KDELR2 | 0.001191676 | 0.046142858 | 892.1782 | 11.138008 | 0.10562125 | 11.131685 | 11.246648 | 11.035689 |
| KDM5B | 0.006068773 | 0.12378013 | 1711.4293 | 10.201076 | 0.04385361 | 10.248803 | 10.191863 | 10.1625595 |
| KHDRBS1 | 0.000154465 | 0.012759524 | 420.28223 | 12.15446 | 0.022433674 | 12.140443 | 12.180334 | 12.142603 |
| KIAA0101 | 0.001024702 | 0.042474315 | 840.8267 | 11.21944 | 0.036292 | 11.178541 | 11.231983 | 11.247798 |
| KIAA0174 | 0.001481668 | 0.053104337 | 971.6035 | 11.01327 | 0.028967926 | 11.016881 | 10.982665 | 11.040262 |
| KIAA0182 | 0.004959445 | 0.10938525 | 1574.6078 | 10.31532 | 0.16035935 | 10.131863 | 10.428791 | 10.385305 |
| KIAA0391 | 0.007418228 | 0.1377773 | 1861.6725 | 10.081437 | 0.103710294 | 10.200937 | 10.02844 | 10.014934 |
| KIAA1191 | 0.006386321 | 0.12719116 | 1748.4177 | 10.170968 | 0.1408309 | 10.053876 | 10.13179 | 10.327241 |
| KIAA1310 | 0.004224304 | 0.09983515 | 1474.2466 | 10.412991 | 0.06390111 | 10.357279 | 10.398948 | 10.482745 |
| KIAA1598 | 0.007188246 | 0.13590682 | 1837.216 | 10.097343 | 0.10447218 | 10.01592 | 10.060971 | 10.21514 |
| KIF20A | 0.009860926 | 0.16376975 | 2096.511 | 9.908042 | 0.098942004 | 9.801998 | 9.924245 | 9.997882 |
| KIF22 | 0.002352684 | 0.07012371 | 1164.3896 | 10.754936 | 0.06265356 | 10.688102 | 10.764366 | 10.81234 |
| KLHDC2 | 0.00345273 | 0.088080145 | 1357.9846 | 10.525601 | 0.0675378 | 10.473839 | 10.601998 | 10.500967 |
| KLHDC3 | 0.001093359 | 0.044159487 | 862.867 | 11.184394 | 0.11974756 | 11.233555 | 11.271735 | 11.047889 |
| KPNA2 | 0.004666628 | 0.105750486 | 1535.735 | 10.355218 | 0.07259774 | 10.272115 | 10.4063015 | 10.387237 |
| KPNA4 | 0.003949991 | 0.09603434 | 1434.846 | 10.452294 | 0.08857466 | 10.554556 | 10.402714 | 10.399613 |
| KPNB1 | 0.000135528 | 0.01181407 | 401.5071 | 12.212601 | 0.08209016 | 12.299446 | 12.136281 | 12.202076 |
| KRT10 | 0.005517871 | 0.116304375 | 1645.8467 | 10.254725 | 0.1908699 | 10.05737 | 10.438371 | 10.2684355 |
| KRT18P13 | 0.002469822 | 0.07206728 | 1187.1678 | 10.722976 | 0.12810822 | 10.75558 | 10.581715 | 10.83163 |
| KRT18P28 | 0.006248833 | 0.12553388 | 1732.6512 | 10.179726 | 0.18844843 | 10.250195 | 9.966199 | 10.322785 |
| KRT19 | 1.36335E-05 | 0.002556757 | 185.54372 | 13.175972 | 0.051694058 | 13.173854 | 13.228693 | 13.12537 |
| KRT8 | 4.68957E-05 | 0.005831541 | 278.4611 | 12.716853 | 0.1155621 | 12.739635 | 12.819327 | 12.591597 |
| KRT80 | 0.002708595 | 0.07677451 | 1231.6432 | 10.665218 | 0.008745201 | 10.65859 | 10.67513 | 10.661934 |
| KRT8P9 | 0.007794489 | 0.14180493 | 1900.2681 | 10.050625 | 0.08120922 | 10.044834 | 9.972466 | 10.134575 |
| KRTCAP2 | 0.000707817 | 0.033502046 | 732.3589 | 11.393851 | 0.0569847 | 11.458311 | 11.373065 | 11.350178 |
| KYNU | 0.000761803 | 0.03496032 | 752.563 | 11.356917 | 0.052696474 | 11.339269 | 11.416173 | 11.3153105 |
| LAGE3 | 0.000581628 | 0.030028274 | 680.86804 | 11.485111 | 0.023527501 | 11.510262 | 11.46364 | 11.481431 |
| LAIR1 | 0.002719692 | 0.07702612 | 1233.5382 | 10.667366 | 0.09784571 | 10.581169 | 10.647208 | 10.773721 |
| LAMA5 | 0.000822534 | 0.036774486 | 774.8832 | 11.325038 | 0.051981956 | 11.315565 | 11.278443 | 11.381104 |
| LAMP1 | 0.003379143 | 0.08709955 | 1346.0789 | 10.539646 | 0.05595252 | 10.556502 | 10.585233 | 10.477203 |
| LAMP2 | 0.002885023 | 0.07956518 | 1263.1951 | 10.630059 | 0.029107666 | 10.613505 | 10.613004 | 10.663669 |
| LAP3 | 0.004008791 | 0.09658403 | 1443.3425 | 10.444818 | 0.034932178 | 10.406621 | 10.475142 | 10.452691 |
| LAPTM4B | 0.000322188 | 0.0204351 | 548.95557 | 11.774301 | 0.015584934 | 11.775693 | 11.758066 | 11.789143 |
| LASP1 | 0.000645587 | 0.031770214 | 707.71277 | 11.436737 | 0.07411525 | 11.483013 | 11.4759445 | 11.3512535 |
| LASS2 | 0.002017265 | 0.0634515 | 1096.084 | 10.837746 | 0.09565995 | 10.727302 | 10.894519 | 10.891417 |
| LCMT1 | 0.008989508 | 0.15455005 | 2016.9829 | 9.963258 | 0.036266465 | 9.922655 | 9.992437 | 9.974681 |
| LDHA | 0.002143483 | 0.06581062 | 1122.3529 | 10.8049135 | 0.0626867 | 10.746633 | 10.796879 | 10.871231 |
| LDLR | 0.000727705 | 0.034210026 | 739.87274 | 11.38045 | 0.10910551 | 11.494652 | 11.277279 | 11.369418 |
| LDOC1 | 0.003029631 | 0.08198908 | 1288.2394 | 10.606017 | 0.06221576 | 10.558539 | 10.583064 | 10.676449 |
| LGALS1 | 0.002314118 | 0.069451556 | 1156.7172 | 10.76101 | 0.09949682 | 10.873145 | 10.683289 | 10.726596 |
| LGALS3 | 0.002909293 | 0.07979051 | 1267.4987 | 10.627487 | 0.12464369 | 10.489593 | 10.660729 | 10.73214 |
| LILRB3 | 0.008226783 | 0.14719959 | 1943.2015 | 10.020526 | 0.021995861 | 10.041059 | 9.997313 | 10.023207 |
| LIPA | 0.009868796 | 0.16382201 | 2097.1965 | 9.9066925 | 0.047231533 | 9.854293 | 9.945989 | 9.919797 |
| LITAF | 0.001686805 | 0.057543755 | 1021.55896 | 10.938214 | 0.22706285 | 10.691295 | 10.985317 | 11.138032 |
| LLPH | 0.003431343 | 0.08785757 | 1354.6282 | 10.531922 | 0.09416355 | 10.639541 | 10.491546 | 10.46468 |
| LMBR1 | 0.004697988 | 0.1059766 | 1540.0144 | 10.3516655 | 0.06060487 | 10.295125 | 10.344224 | 10.4156475 |
| LMNB2 | 0.007422292 | 0.13770534 | 1862.0895 | 10.0709715 | 0.19957525 | 10.169461 | 9.841295 | 10.202155 |
| LMTK3 | 0.004819566 | 0.10725465 | 1556.4529 | 10.336951 | 0.08833388 | 10.418793 | 10.34875 | 10.243311 |
| LOC100127993 | 5.67822E-06 | 0.001340136 | 143.18288 | 13.437149 | 0.056229226 | 13.385772 | 13.428458 | 13.497218 |
| LOC100128062 | 0.002805442 | 0.07836715 | 1248.6471 | 10.652366 | 0.10363007 | 10.680137 | 10.537679 | 10.73928 |
| LOC100128084 | 0.000375627 | 0.022585789 | 579.7567 | 11.710167 | 0.09770632 | 11.729165 | 11.604357 | 11.796979 |
| LOC100128266 | 0.000223756 | 0.016240586 | 480.2793 | 11.973961 | 0.064718045 | 11.902962 | 12.029653 | 11.989271 |
| LOC100128288 | 0.002400012 | 0.07098551 | 1173.837 | 10.73711 | 0.09198213 | 10.661531 | 10.710273 | 10.839526 |
| LOC100128353 | 0.001462616 | 0.052584454 | 966.5644 | 11.019711 | 0.054709624 | 10.963057 | 11.023831 | 11.072243 |
| LOC100128410 | 0.000954401 | 0.04028224 | 818.9059 | 11.257339 | 0.05020426 | 11.285326 | 11.199379 | 11.287311 |
| LOC100128505 | 3.80181E-05 | 0.005073077 | 259.08835 | 12.806743 | 0.10872832 | 12.825278 | 12.689938 | 12.905011 |
| LOC100128731 | 0.000173776 | 0.013733485 | 437.73575 | 12.098386 | 0.081032075 | 12.130765 | 12.158221 | 12.00617 |
| LOC100128936 | 0.000156338 | 0.012822695 | 421.88943 | 12.145972 | 0.073386714 | 12.061348 | 12.18445 | 12.192118 |
| LOC100129028 | 3.12734E-05 | 0.004520834 | 243.21936 | 12.87455 | 0.13464634 | 12.731456 | 12.893439 | 12.998754 |
| LOC100129086 | 0.005562172 | 0.11695394 | 1651.1487 | 10.2514305 | 0.0541038 | 10.2547245 | 10.303811 | 10.195754 |
| LOC100129141 | 2.88234E-07 | 0.000172414 | 56.763355 | 13.950967 | 0.079993695 | 14.021168 | 13.967855 | 13.863877 |
| LOC100129158 | 2.88234E-08 | 2.43902E-05 | 39.28911 | 14.034839 | 0.09119343 | 14.1177025 | 14.049678 | 13.937136 |
| LOC100129362 | 0.000297948 | 0.0196148 | 532.71625 | 11.832004 | 0.1312727 | 11.901083 | 11.680614 | 11.91431 |
| LOC100129379 | 7.34997E-06 | 0.001655844 | 153.54134 | 13.369456 | 0.11574853 | 13.497218 | 13.339565 | 13.2715845 |
| LOC100129553 | 2.88234E-07 | 0.000172414 | 57.072445 | 13.9556265 | 0.047782905 | 13.97997 | 13.986335 | 13.900574 |
| LOC100129599 | 0.005211362 | 0.1123 | 1607.0734 | 10.286773 | 0.15928756 | 10.291645 | 10.125105 | 10.443568 |
| LOC100129650 | 0.004294748 | 0.10060905 | 1484.2644 | 10.40593 | 0.03550571 | 10.426429 | 10.364931 | 10.426429 |
| LOC100129685 | 0.003479564 | 0.08843956 | 1362.1688 | 10.525802 | 0.0486681 | 10.470273 | 10.561051 | 10.54608 |
| LOC100129742 | 0.00010411 | 0.009788618 | 365.88293 | 12.350517 | 0.056843683 | 12.306153 | 12.330805 | 12.414593 |
| LOC100129758 | 0 | 0 | 29.071564 | 14.09921 | 0.06275582 | 14.1714735 | 14.067751 | 14.058407 |
| LOC100129902 | 3.45881E-07 | 0.000169014 | 65.55625 | 13.893586 | 0.11677313 | 13.999042 | 13.768089 | 13.913627 |
| LOC100130003 | 3.35505E-05 | 0.004770492 | 248.77524 | 12.852565 | 0.06283602 | 12.7802105 | 12.884043 | 12.893439 |
| LOC100130070 | 0.001868508 | 0.060984008 | 1064.058 | 10.881246 | 0.12737834 | 10.903527 | 10.744198 | 10.996015 |
| LOC100130168 | 0.000130455 | 0.011516539 | 396.26974 | 12.241752 | 0.20681143 | 12.30409 | 12.0109415 | 12.410223 |
| LOC100130308 | 0.000631204 | 0.03160029 | 701.8658 | 11.455241 | 0.14226183 | 11.360854 | 11.618868 | 11.386001 |
| LOC100130446 | 0 | 0 | 24.301746 | 14.118775 | 0.06697307 | 14.178795 | 14.046533 | 14.131 |
| LOC100130516 | 6.94068E-05 | 0.007572327 | 318.7906 | 12.535969 | 0.23603031 | 12.783144 | 12.31294 | 12.511823 |
| LOC100130553 | 0 | 0 | 14.460854 | 14.20618 | 0.053080395 | 14.264281 | 14.194031 | 14.160227 |
| LOC100130561 | 0.007214562 | 0.13618171 | 1840.1221 | 10.091501 | 0.18155731 | 10.105036 | 9.903556 | 10.265913 |
| LOC100130562 | 0.00205903 | 0.06429883 | 1104.8978 | 10.824584 | 0.065785006 | 10.75816 | 10.825883 | 10.88971 |
| LOC100130835 | 0.003912636 | 0.09532654 | 1429.5044 | 10.455993 | 0.18127754 | 10.512888 | 10.253093 | 10.601998 |
| LOC100130886 | 0.004133539 | 0.09849519 | 1461.4501 | 10.426673 | 0.060817134 | 10.487612 | 10.426429 | 10.365978 |
| LOC100130919 | 0.000352424 | 0.021756228 | 566.5036 | 11.734955 | 0.03007375 | 11.759286 | 11.701332 | 11.744248 |
| LOC100130980 | 0 | 0 | 31.89156 | 14.062809 | 0.15670061 | 14.243352 | 13.982943 | 13.962132 |
| LOC100131196 | 2.36352E-06 | 0.000803922 | 103.83604 | 13.67307 | 0.060636517 | 13.60308 | 13.706386 | 13.709744 |
| LOC100131205 | 0.000132732 | 0.011628788 | 398.7499 | 12.22404 | 0.05655786 | 12.267901 | 12.160205 | 12.244011 |
| LOC100131387 | 4.89998E-07 | 0.0002125 | 73.80555 | 13.817459 | 0.16276766 | 13.717319 | 13.72979 | 14.005269 |
| LOC100131531 | 0.000388165 | 0.022825424 | 586.33887 | 11.692757 | 0.06743762 | 11.697328 | 11.623149 | 11.7577915 |
| LOC100131609 | 0.000502364 | 0.027147975 | 644.3032 | 11.558625 | 0.066515796 | 11.489348 | 11.6219845 | 11.564544 |
| LOC100131735 | 0.005081599 | 0.110881135 | 1590.5785 | 10.30505 | 0.119122624 | 10.181192 | 10.315164 | 10.418793 |
| LOC100131785 | 0.004286793 | 0.10055848 | 1483.1925 | 10.403991 | 0.024858395 | 10.432687 | 10.389081 | 10.3902025 |
| LOC100131801 | 0.000827982 | 0.036734015 | 776.9573 | 11.316811 | 0.17566797 | 11.364598 | 11.122193 | 11.46364 |
| LOC100131866 | 0.008830633 | 0.15303198 | 2001.8414 | 9.977253 | 0.062268175 | 10.031386 | 9.991168 | 9.9092045 |
| LOC100131905 | 2.06664E-05 | 0.003382076 | 211.89932 | 13.033295 | 0.055520725 | 13.028425 | 13.09109 | 12.98037 |
| LOC100132037 | 0.00172514 | 0.058392197 | 1030.959 | 10.929428 | 0.14166656 | 10.951347 | 10.778079 | 11.058857 |
| LOC100132291 | 2.34334E-05 | 0.003662162 | 221.05331 | 12.982944 | 0.07515764 | 12.988837 | 12.905011 | 13.054979 |
| LOC100132391 | 0.001763504 | 0.05911401 | 1039.9016 | 10.914071 | 0.123639606 | 10.959369 | 10.774172 | 11.008675 |
| LOC100132457 | 0.000187122 | 0.014331126 | 450.67657 | 12.062317 | 0.14537665 | 12.164173 | 11.895832 | 12.1269455 |
| LOC100132488 | 0 | 0 | 19.02674 | 14.1579485 | 0.12324481 | 14.160227 | 14.28004 | 14.033582 |
| LOC100132499 | 0.001115496 | 0.044689376 | 869.5881 | 11.173448 | 0.022347728 | 11.198528 | 11.155649 | 11.166166 |
| LOC100132593 | 0 | 0 | 12.557073 | 14.221985 | 0.05060395 | 14.163623 | 14.248689 | 14.253644 |
| LOC100132673 | 8.33574E-05 | 0.008505883 | 339.53854 | 12.469087 | 0.04983086 | 12.425958 | 12.523637 | 12.457667 |
| LOC100132727 | 0.002069897 | 0.06458004 | 1107.2649 | 10.827583 | 0.019227384 | 10.84976 | 10.81557 | 10.817422 |
| LOC100132742 | 3.37522E-05 | 0.004760162 | 249.5902 | 12.840461 | 0.10533864 | 12.7603855 | 12.801207 | 12.95979 |
| LOC100132795 | 2.26552E-05 | 0.00362212 | 218.64494 | 12.991554 | 0.02739471 | 13.006478 | 13.008246 | 12.959938 |
| LOC100133233 | 6.18839E-05 | 0.007085809 | 305.53912 | 12.596626 | 0.14907247 | 12.7623825 | 12.5539465 | 12.473547 |
| LOC100133273 | 3.54528E-06 | 0.001042373 | 118.5868 | 13.57496 | 0.13336003 | 13.7022505 | 13.586366 | 13.436263 |
| LOC100133328 | 0.0072614 | 0.13647184 | 1845.1273 | 10.0903635 | 0.110644735 | 10.02216 | 10.030906 | 10.218025 |
| LOC100133372 | 0.00012348 | 0.011041237 | 388.9732 | 12.270779 | 0.16650626 | 12.2058115 | 12.459974 | 12.146549 |
| LOC100133465 | 3.17058E-07 | 0.000164179 | 63.189903 | 13.90554 | 0.1291602 | 13.92414 | 14.024393 | 13.768089 |
| LOC100133477 | 0.001135931 | 0.044988584 | 876.0761 | 11.160203 | 0.1202678 | 11.298586 | 11.080908 | 11.101117 |
| LOC100133607 | 5.07292E-06 | 0.001284672 | 136.00241 | 13.48473 | 0.14412275 | 13.543603 | 13.32049 | 13.5900955 |
| LOC100133649 | 8.12821E-06 | 0.00178481 | 157.64493 | 13.346324 | 0.10350224 | 13.363307 | 13.235379 | 13.440283 |
| LOC100133772 | 0.000761169 | 0.03502387 | 752.28143 | 11.360768 | 0.096269235 | 11.411302 | 11.249754 | 11.4212475 |
| LOC100133812 | 8.93526E-07 | 0.000348315 | 83.53011 | 13.790359 | 0.038836725 | 13.772279 | 13.763858 | 13.834939 |
| LOC100133876 | 2.30587E-07 | 0.000148148 | 56.18983 | 13.956028 | 0.079510525 | 14.046533 | 13.92414 | 13.897411 |
| LOC100133931 | 3.63175E-06 | 0.00102439 | 121.28086 | 13.553207 | 0.14247039 | 13.461236 | 13.717319 | 13.481066 |
| LOC100134134 | 0.002182856 | 0.066489905 | 1130.4598 | 10.795063 | 0.051496115 | 10.803048 | 10.740041 | 10.8421 |
| LOC100134159 | 0.000664178 | 0.03218296 | 715.1365 | 11.431348 | 0.17793418 | 11.633274 | 11.363249 | 11.297522 |
| LOC100134273 | 0.004683577 | 0.10585798 | 1538.0488 | 10.355331 | 0.110397406 | 10.462642 | 10.242086 | 10.361268 |
| LOC100134504 | 0.001059578 | 0.043299176 | 851.99677 | 11.200084 | 0.1134868 | 11.247608 | 11.07056 | 11.282082 |
| LOC100134537 | 0.003552833 | 0.08957994 | 1374.1272 | 10.51171 | 0.108199075 | 10.3963995 | 10.611014 | 10.527717 |
| LOC100190938 | 0.000139563 | 0.011985148 | 405.79037 | 12.194283 | 0.046780106 | 12.172641 | 12.162242 | 12.247965 |
| LOC124512 | 0.004004237 | 0.096541345 | 1442.6912 | 10.445624 | 0.112324856 | 10.423283 | 10.34615 | 10.567442 |
| LOC127295 | 0.00015164 | 0.012646635 | 417.63467 | 12.166401 | 0.10839965 | 12.2000675 | 12.253972 | 12.045162 |
| LOC128192 | 0.000417507 | 0.023784893 | 602.4983 | 11.654696 | 0.06859505 | 11.611436 | 11.733788 | 11.618868 |
| LOC134997 | 8.70468E-06 | 0.001864198 | 160.32864 | 13.324891 | 0.08367509 | 13.420293 | 13.263943 | 13.290441 |
| LOC148430 | 5.07292E-06 | 0.001284672 | 135.89636 | 13.46622 | 0.17570876 | 13.28091 | 13.4873295 | 13.63042 |
| LOC148915 | 4.29181E-05 | 0.00555597 | 270.87943 | 12.746544 | 0.04773883 | 12.8014765 | 12.715102 | 12.723053 |
| LOC151162 | 0.006958754 | 0.13345882 | 1812.564 | 10.119816 | 0.07855991 | 10.122679 | 10.196905 | 10.039864 |
| LOC151579 | 0.001948521 | 0.062248617 | 1081.4806 | 10.861703 | 0.11854905 | 10.869875 | 10.73928 | 10.975955 |
| LOC158345 | 9.41373E-05 | 0.009174157 | 353.2308 | 12.401589 | 0.27169532 | 12.096649 | 12.61791 | 12.490209 |
| LOC203547 | 0.004298265 | 0.10055563 | 1484.8142 | 10.400745 | 0.064365774 | 10.35557 | 10.372223 | 10.474444 |
| LOC220433 | 0.000212342 | 0.015641188 | 471.0822 | 12.007476 | 0.1011648 | 12.004269 | 11.907952 | 12.110206 |
| LOC255783 | 0.006400271 | 0.12732282 | 1750.1038 | 10.165691 | 0.07362716 | 10.206852 | 10.080688 | 10.209535 |
| LOC283412 | 4.87692E-05 | 0.005957746 | 282.79004 | 12.684563 | 0.15149851 | 12.660862 | 12.846515 | 12.546311 |
| LOC284230 | 2.88234E-08 | 2.43902E-05 | 35.243473 | 14.072422 | 0.019376861 | 14.094796 | 14.061234 | 14.061234 |
| LOC284393 | 0 | 0 | 22.53115 | 14.080363 | 0.2606139 | 14.16775 | 14.286052 | 13.787285 |
| LOC284821 | 8.21468E-06 | 0.001792453 | 157.95541 | 13.33332 | 0.04117323 | 13.323884 | 13.378391 | 13.297683 |
| LOC285053 | 9.16585E-06 | 0.001927273 | 163.8271 | 13.31039 | 0.05931258 | 13.370911 | 13.307897 | 13.252364 |
| LOC285176 | 0.001508417 | 0.0537852 | 978.4824 | 11.003177 | 0.089185335 | 10.9020815 | 11.036729 | 11.07072 |
| LOC285900 | 2.18193E-05 | 0.00352093 | 215.79686 | 13.008914 | 0.1121216 | 13.136475 | 12.964301 | 12.925965 |
| LOC286016 | 0.003742924 | 0.09262268 | 1403.202 | 10.482757 | 0.08661058 | 10.402093 | 10.574288 | 10.4718895 |
| LOC286157 | 0.002987058 | 0.081026584 | 1281.0432 | 10.608997 | 0.06506964 | 10.542607 | 10.672661 | 10.611725 |
| LOC286444 | 1.27111E-05 | 0.00245 | 182.2257 | 13.186393 | 0.02852176 | 13.217978 | 13.16252 | 13.178679 |
| LOC286512 | 0.004992679 | 0.10983893 | 1579.0382 | 10.31266 | 0.22403255 | 10.1144905 | 10.26774 | 10.555749 |
| LOC338870 | 8.33574E-05 | 0.008505883 | 339.53748 | 12.462723 | 0.047442332 | 12.408168 | 12.494312 | 12.485688 |
| LOC340598 | 0.008506744 | 0.14981371 | 1970.3969 | 9.997945 | 0.07669655 | 10.072609 | 10.001862 | 9.919366 |
| LOC341315 | 0.000956967 | 0.040292475 | 819.6548 | 11.252379 | 0.044148743 | 11.257358 | 11.205952 | 11.293827 |
| LOC341457 | 0 | 0 | 19.264986 | 14.164086 | 0.060423 | 14.142141 | 14.232415 | 14.1177025 |
| LOC343184 | 2.88234E-07 | 0.000172414 | 58.35533 | 13.947631 | 0.05979751 | 14.014919 | 13.900574 | 13.927398 |
| LOC345041 | 0.006696172 | 0.13029557 | 1783.5723 | 10.141267 | 0.03224164 | 10.164914 | 10.104541 | 10.1543455 |
| LOC347544 | 6.57751E-05 | 0.007290735 | 312.33286 | 12.579133 | 0.07246886 | 12.501241 | 12.591597 | 12.644562 |
| LOC374395 | 0.000384159 | 0.022821918 | 584.5002 | 11.700539 | 0.11043357 | 11.682081 | 11.819038 | 11.600497 |
| LOC387841 | 0.00189514 | 0.061391223 | 1069.7797 | 10.874107 | 0.03689309 | 10.878348 | 10.908696 | 10.835277 |
| LOC387867 | 1.93693E-05 | 0.0032 | 208.34853 | 13.058983 | 0.07086476 | 12.98037 | 13.078626 | 13.117955 |
| LOC387930 | 0 | 0 | 16.286507 | 14.184189 | 0.10741674 | 14.085939 | 14.298878 | 14.16775 |
| LOC388076 | 0.001649594 | 0.056552373 | 1012.6477 | 10.959508 | 0.15571634 | 11.055631 | 10.77985 | 11.043046 |
| LOC388275 | 0.002157203 | 0.065998234 | 1125.2418 | 10.800105 | 0.15513581 | 10.873959 | 10.904514 | 10.62184 |
| LOC388339 | 4.36387E-05 | 0.005545788 | 272.26984 | 12.738912 | 0.07923982 | 12.813119 | 12.655452 | 12.748165 |
| LOC388474 | 0 | 0 | 28.953552 | 14.096147 | 0.07810306 | 14.049678 | 14.052444 | 14.186318 |
| LOC388524 | 0.003320891 | 0.08630337 | 1336.6416 | 10.552686 | 0.03281407 | 10.578778 | 10.515846 | 10.563436 |
| LOC388532 | 0.000146942 | 0.012464548 | 413.0995 | 12.177075 | 0.1285994 | 12.243964 | 12.028818 | 12.258447 |
| LOC388556 | 0.001039661 | 0.042787664 | 845.646 | 11.213249 | 0.06420637 | 11.160477 | 11.284733 | 11.194539 |
| LOC388564 | 0.003756211 | 0.09275302 | 1405.2845 | 10.478688 | 0.13732755 | 10.4996395 | 10.604337 | 10.332089 |
| LOC388654 | 4.5541E-06 | 0.001206107 | 130.23186 | 13.489571 | 0.1377052 | 13.640566 | 13.457233 | 13.370911 |
| LOC388720 | 0 | 0 | 14.325698 | 14.205357 | 0.12525749 | 14.347527 | 14.157298 | 14.111245 |
| LOC388789 | 0.001233239 | 0.047069307 | 904.2784 | 11.118203 | 0.04911847 | 11.106166 | 11.076223 | 11.172222 |
| LOC389101 | 0.003912175 | 0.09538229 | 1429.4204 | 10.455758 | 0.061640512 | 10.525649 | 10.409153 | 10.43247 |
| LOC389141 | 0.007293452 | 0.13685182 | 1848.3276 | 10.090069 | 0.044270698 | 10.085613 | 10.048195 | 10.136399 |
| LOC389156 | 0.000532369 | 0.028328221 | 658.8603 | 11.5284815 | 0.09207165 | 11.526105 | 11.437621 | 11.621718 |
| LOC389168 | 2.2367E-05 | 0.003592593 | 217.5544 | 13.008504 | 0.03945122 | 12.9947605 | 12.977763 | 13.052989 |
| LOC389223 | 1.44117E-07 | 9.61538E-05 | 51.808716 | 13.970155 | 0.101379074 | 13.853106 | 14.030205 | 14.027154 |
| LOC389342 | 1.421E-05 | 0.00262234 | 188.08257 | 13.1656065 | 0.11579152 | 13.031947 | 13.235446 | 13.229426 |
| LOC389404 | 0.004307344 | 0.100632325 | 1486.0548 | 10.403772 | 0.07420078 | 10.4246235 | 10.321377 | 10.465317 |
| LOC389435 | 1.8447E-06 | 0.000666667 | 98.49122 | 13.704414 | 0.121324144 | 13.802878 | 13.568879 | 13.741484 |
| LOC389787 | 6.13074E-05 | 0.007066445 | 304.5605 | 12.603753 | 0.16236067 | 12.466886 | 12.561231 | 12.783144 |
| LOC390345 | 1.10682E-05 | 0.002285714 | 173.82788 | 13.234108 | 0.10997932 | 13.286724 | 13.107705 | 13.307897 |
| LOC390354 | 1.49882E-05 | 0.002708333 | 191.3613 | 13.139313 | 0.14873667 | 13.097436 | 13.304499 | 13.016004 |
| LOC390466 | 0.007725457 | 0.14084445 | 1893.4076 | 10.051665 | 0.103086956 | 10.126836 | 9.934149 | 10.09401 |
| LOC391019 | 0.001120857 | 0.04480069 | 871.25183 | 11.173045 | 0.11538045 | 11.278443 | 11.049769 | 11.190923 |
| LOC391075 | 0.000153081 | 0.012736211 | 418.85162 | 12.157966 | 0.09179325 | 12.204117 | 12.217525 | 12.052255 |
| LOC391126 | 8.82573E-05 | 0.008748571 | 345.54797 | 12.431594 | 0.10794443 | 12.370295 | 12.368255 | 12.5562315 |
| LOC391370 | 1.55358E-05 | 0.002764103 | 193.39607 | 13.135091 | 0.056280933 | 13.180369 | 13.072079 | 13.1528225 |
| LOC391656 | 8.99291E-06 | 0.001902439 | 162.21971 | 13.321446 | 0.13425292 | 13.39707 | 13.40083 | 13.16644 |
| LOC391777 | 2.88234E-07 | 0.000172414 | 57.619385 | 13.948929 | 0.047799665 | 13.934089 | 14.002388 | 13.910309 |
| LOC391811 | 0.00018006 | 0.014006726 | 443.85382 | 12.086098 | 0.09114062 | 12.098449 | 11.989412 | 12.170434 |
| LOC391833 | 3.87387E-05 | 0.005129771 | 260.86966 | 12.793223 | 0.11757 | 12.786307 | 12.679264 | 12.914099 |
| LOC392285 | 0.000776071 | 0.035196077 | 757.8169 | 11.351715 | 0.13580893 | 11.3078575 | 11.243254 | 11.504033 |
| LOC392437 | 0.001638871 | 0.056351833 | 1010.2234 | 10.955635 | 0.08896687 | 10.9183855 | 11.057172 | 10.891347 |
| LOC399748 | 0.004232202 | 0.09981781 | 1475.2346 | 10.415114 | 0.081889674 | 10.450724 | 10.47317 | 10.321448 |
| LOC399900 | 0.000246411 | 0.017201208 | 498.16058 | 11.925525 | 0.13827226 | 11.9229765 | 11.788545 | 12.065054 |
| LOC399942 | 0.001749842 | 0.058883607 | 1036.8077 | 10.9152565 | 0.05456569 | 10.936731 | 10.955818 | 10.853221 |
| LOC399965 | 0.000741944 | 0.034551676 | 744.9956 | 11.3691635 | 0.107511915 | 11.493226 | 11.31102 | 11.303244 |
| LOC400027 | 0.006534761 | 0.12852438 | 1765.3245 | 10.153665 | 0.030711628 | 10.1542225 | 10.122679 | 10.184094 |
| LOC400721 | 3.47322E-05 | 0.004839358 | 251.67255 | 12.829776 | 0.22999904 | 12.896516 | 12.573787 | 13.019023 |
| LOC400948 | 0.000295238 | 0.019585086 | 530.5166 | 11.829464 | 0.052431874 | 11.888567 | 11.81128 | 11.788545 |
| LOC400963 | 0 | 0 | 14.81248 | 14.214676 | 0.063273475 | 14.2585335 | 14.142141 | 14.243352 |
| LOC401019 | 2.30587E-07 | 0.000148148 | 54.02651 | 13.964058 | 0.05430711 | 13.923879 | 14.025843 | 13.942452 |
| LOC401115 | 5.20263E-05 | 0.006267361 | 288.8265 | 12.680031 | 0.054364413 | 12.742344 | 12.642296 | 12.655452 |
| LOC401206 | 0 | 0 | 1.587401 | 14.412133 | 0.030425765 | 14.438385 | 14.378787 | 14.419228 |
| LOC401397 | 0.003693405 | 0.091987796 | 1395.6853 | 10.494756 | 0.0605191 | 10.425673 | 10.520174 | 10.53842 |
| LOC402057 | 0.000828702 | 0.036672194 | 777.2256 | 11.320758 | 0.09542176 | 11.324392 | 11.22357 | 11.4143095 |
| LOC402112 | 0.006195884 | 0.1251951 | 1726.5535 | 10.187416 | 0.03448273 | 10.218556 | 10.150357 | 10.193336 |
| LOC402175 | 0.000159134 | 0.012990588 | 424.4273 | 12.144311 | 0.08356185 | 12.065054 | 12.231598 | 12.136281 |
| LOC402251 | 3.62022E-05 | 0.004944882 | 255.30222 | 12.8219385 | 0.19573629 | 12.7058935 | 12.711996 | 13.047929 |
| LOC402644 | 0.000643944 | 0.031734373 | 707.18256 | 11.436348 | 0.122526996 | 11.339261 | 11.395765 | 11.574018 |
| LOC402694 | 4.49646E-06 | 0.0012 | 129.74689 | 13.497185 | 0.16812135 | 13.538834 | 13.640566 | 13.312153 |
| LOC439953 | 6.13074E-05 | 0.007066445 | 304.59805 | 12.606583 | 0.10310745 | 12.489186 | 12.682449 | 12.648111 |
| LOC440027 | 0 | 0 | 23.838615 | 14.142078 | 0.0409062 | 14.097986 | 14.149455 | 14.178795 |
| LOC440043 | 0.000154321 | 0.012778043 | 420.16333 | 12.152245 | 0.07900342 | 12.067249 | 12.166051 | 12.223436 |
| LOC440055 | 0.000100219 | 0.009631579 | 360.6056 | 12.384269 | 0.093620874 | 12.276238 | 12.434818 | 12.441749 |
| LOC440063 | 8.20027E-05 | 0.008417159 | 337.3127 | 12.472402 | 0.12651694 | 12.452954 | 12.356733 | 12.607515 |
| LOC440093 | 0.006843662 | 0.13227521 | 1799.9696 | 10.124458 | 0.09543502 | 10.162003 | 10.015961 | 10.195412 |
| LOC440353 | 0.006906238 | 0.1328924 | 1806.7782 | 10.1235 | 0.08006886 | 10.126254 | 10.202155 | 10.0420885 |
| LOC440575 | 2.53646E-06 | 0.000838095 | 105.4993 | 13.657424 | 0.058051016 | 13.720659 | 13.645062 | 13.606548 |
| LOC440589 | 0 | 0 | 13.782348 | 14.216266 | 0.12770015 | 14.252494 | 14.074366 | 14.321938 |
| LOC440595 | 0.000267453 | 0.018337945 | 512.22345 | 11.890178 | 0.100163646 | 11.787698 | 11.894983 | 11.987852 |
| LOC440733 | 0 | 0 | 14.016986 | 14.210972 | 0.05602131 | 14.174872 | 14.182534 | 14.275508 |
| LOC440737 | 5.70704E-06 | 0.001337838 | 144.21008 | 13.432933 | 0.051941298 | 13.40083 | 13.40511 | 13.492859 |
| LOC440926 | 0.000849081 | 0.037194446 | 784.13965 | 11.309082 | 0.14148735 | 11.189982 | 11.27178 | 11.465483 |
| LOC440927 | 3.33487E-05 | 0.004761317 | 248.17891 | 12.849144 | 0.099854246 | 12.914099 | 12.899169 | 12.734165 |
| LOC440991 | 0.003024212 | 0.08197031 | 1287.3702 | 10.600606 | 0.124333255 | 10.62112 | 10.713406 | 10.467291 |
| LOC441013 | 0.00139318 | 0.05120233 | 948.5843 | 11.048604 | 0.09603776 | 11.05317 | 10.950365 | 11.142278 |
| LOC441034 | 2.88234E-08 | 2.43902E-05 | 32.60126 | 14.061673 | 0.17409766 | 14.091952 | 13.874422 | 14.218645 |
| LOC441073 | 0.000583415 | 0.03007578 | 681.5629 | 11.484622 | 0.1065225 | 11.506269 | 11.368938 | 11.578658 |
| LOC441087 | 0.000131694 | 0.011567089 | 397.86246 | 12.230403 | 0.051043246 | 12.188444 | 12.28723 | 12.215536 |
| LOC441089 | 0.008363463 | 0.14826877 | 1956.4113 | 10.010148 | 0.10316913 | 10.04437 | 9.894217 | 10.091858 |
| LOC441246 | 2.88234E-08 | 2.43902E-05 | 36.894268 | 14.0576935 | 0.069783196 | 14.101088 | 13.977197 | 14.094796 |
| LOC441377 | 0.000684297 | 0.032746207 | 723.08026 | 11.413901 | 0.06323655 | 11.3833685 | 11.371723 | 11.486609 |
| LOC441506 | 0.001083934 | 0.043829836 | 859.9568 | 11.193984 | 0.012543305 | 11.201919 | 11.1795225 | 11.200509 |
| LOC441775 | 3.45881E-07 | 0.000169014 | 65.72891 | 13.877521 | 0.1510166 | 13.706386 | 13.9920845 | 13.934089 |
| LOC441876 | 0 | 0 | 7.3061438 | 14.294881 | 0.058562808 | 14.22742 | 14.332649 | 14.324575 |
| LOC442454 | 4.35522E-05 | 0.005575646 | 271.95264 | 12.735061 | 0.12524128 | 12.631106 | 12.874104 | 12.69997 |
| LOC442727 | 0.006494726 | 0.1283189 | 1760.8132 | 10.157001 | 0.13529293 | 10.140889 | 10.030484 | 10.299627 |
| LOC550643 | 0.000332104 | 0.020835442 | 554.7372 | 11.768399 | 0.0681488 | 11.6986685 | 11.771682 | 11.834847 |
| LOC641768 | 0.000737909 | 0.034502696 | 743.60583 | 11.374973 | 0.17962544 | 11.334855 | 11.2188 | 11.571266 |
| LOC641814 | 4.15057E-06 | 0.001125 | 126.165565 | 13.535812 | 0.08744945 | 13.623584 | 13.4486885 | 13.535164 |
| LOC641848 | 0.008655099 | 0.1517332 | 1984.6747 | 9.985637 | 0.08874556 | 9.941972 | 9.927182 | 10.087754 |
| LOC642197 | 0.00242921 | 0.07148346 | 1179.3103 | 10.734492 | 0.06158481 | 10.676419 | 10.799072 | 10.727986 |
| LOC642210 | 0 | 0 | 1.8171206 | 14.409177 | 0.05728955 | 14.419228 | 14.347527 | 14.460775 |
| LOC642250 | 0.004727273 | 0.10615405 | 1544.113 | 10.350765 | 0.055832993 | 10.393936 | 10.287713 | 10.370648 |
| LOC642333 | 0.002742722 | 0.077362604 | 1237.5951 | 10.667054 | 0.105259955 | 10.701596 | 10.548863 | 10.750703 |
| LOC642357 | 2.88234E-07 | 0.000172414 | 57.83983 | 13.911347 | 0.1585433 | 13.795114 | 14.091952 | 13.846977 |
| LOC642590 | 0.003424137 | 0.08773781 | 1353.287 | 10.532414 | 0.14048676 | 10.582508 | 10.640988 | 10.373747 |
| LOC642741 | 5.85692E-05 | 0.006795987 | 300.68414 | 12.619924 | 0.12545033 | 12.583541 | 12.759544 | 12.516686 |
| LOC642755 | 0.006541275 | 0.1285068 | 1766.0499 | 10.153787 | 0.027105078 | 10.159366 | 10.124326 | 10.177668 |
| LOC642817 | 0.000471494 | 0.025882911 | 629.8246 | 11.602103 | 0.19397298 | 11.738053 | 11.688283 | 11.379973 |
| LOC642828 | 0.000251801 | 0.017437126 | 502.38315 | 11.919426 | 0.06427926 | 11.853444 | 11.981855 | 11.9229765 |
| LOC642892 | 1.44117E-07 | 9.61538E-05 | 51.980762 | 13.958301 | 0.15349677 | 14.134437 | 13.88736 | 13.853106 |
| LOC642975 | 0.002781749 | 0.078082524 | 1244.426 | 10.649689 | 0.060837105 | 10.70229 | 10.663711 | 10.583064 |
| LOC642989 | 0.000160402 | 0.013063381 | 425.41095 | 12.135352 | 0.047131646 | 12.119161 | 12.188444 | 12.098449 |
| LOC643031 | 0.002090967 | 0.06494539 | 1111.7028 | 10.8164625 | 0.10087815 | 10.819114 | 10.915989 | 10.714285 |
| LOC643284 | 0 | 0 | 25.114145 | 14.131432 | 0.047360558 | 14.157298 | 14.160227 | 14.076771 |
| LOC643287 | 0.001446475 | 0.052275 | 962.0209 | 11.027996 | 0.069376685 | 11.02471 | 10.96032 | 11.098957 |
| LOC643357 | 0.000269297 | 0.018391732 | 513.20184 | 11.886378 | 0.050108545 | 11.872068 | 11.942084 | 11.84498 |
| LOC643358 | 3.45881E-07 | 0.000169014 | 64.90005 | 13.90467 | 0.10536367 | 13.913627 | 14.005269 | 13.795114 |
| LOC643431 | 0.001842509 | 0.06059147 | 1058.2944 | 10.890762 | 0.17917632 | 10.863797 | 10.726596 | 11.081892 |
| LOC643433 | 0.000226379 | 0.016227273 | 482.40833 | 11.970123 | 0.054449435 | 12.00932 | 11.993098 | 11.907952 |
| LOC643509 | 0.008655474 | 0.15166312 | 1984.7228 | 9.988537 | 0.058699597 | 10.041922 | 9.925675 | 9.998013 |
| LOC643531 | 1.70635E-05 | 0.002945274 | 199.2173 | 13.092785 | 0.08093257 | 13.016004 | 13.177312 | 13.08504 |
| LOC643668 | 0.009952874 | 0.1645093 | 2104.6885 | 9.900161 | 0.029749319 | 9.873809 | 9.932421 | 9.894252 |
| LOC643863 | 1.22788E-05 | 0.002420455 | 179.73715 | 13.21418 | 0.11759833 | 13.335486 | 13.100678 | 13.206377 |
| LOC643997 | 0.004660806 | 0.10568758 | 1534.946 | 10.353771 | 0.051903907 | 10.2964945 | 10.367125 | 10.397693 |
| LOC644029 | 0.000102381 | 0.009731507 | 363.1261 | 12.355491 | 0.11190236 | 12.296984 | 12.484517 | 12.284968 |
| LOC644039 | 0 | 0 | 24.454866 | 14.09969 | 0.23003872 | 14.024393 | 13.916738 | 14.357943 |
| LOC644214 | 0.002707241 | 0.076798856 | 1231.4053 | 10.673691 | 0.14038472 | 10.650723 | 10.546206 | 10.824143 |
| LOC644237 | 0.003836283 | 0.09399435 | 1417.7236 | 10.467693 | 0.058988176 | 10.436595 | 10.535724 | 10.430761 |
| LOC644315 | 0.009820747 | 0.16365081 | 2092.773 | 9.907352 | 0.06923616 | 9.9045105 | 9.839581 | 9.977965 |
| LOC644464 | 2.24823E-06 | 0.000795918 | 101.89455 | 13.685609 | 0.11884996 | 13.61971 | 13.614308 | 13.82281 |
| LOC644511 | 0.000223929 | 0.016219206 | 480.56668 | 11.978404 | 0.036140345 | 11.94066 | 12.012693 | 11.981855 |
| LOC644604 | 2.88234E-08 | 2.43902E-05 | 41.91593 | 14.039657 | 0.06715813 | 14.076771 | 13.962132 | 14.080067 |
| LOC644743 | 0.005849542 | 0.12065636 | 1685.4835 | 10.223359 | 0.048061296 | 10.240788 | 10.260274 | 10.169015 |
| LOC644745 | 0.000208883 | 0.015485043 | 468.11642 | 12.018978 | 0.07483948 | 12.048105 | 11.933954 | 12.074875 |
| LOC644774 | 0.004878163 | 0.108142495 | 1564.0896 | 10.329362 | 0.08186061 | 10.27316 | 10.291645 | 10.423283 |
| LOC644790 | 0.001058915 | 0.043323115 | 851.86633 | 11.204183 | 0.056004763 | 11.14097 | 11.2239685 | 11.247608 |
| LOC644863 | 0.000170548 | 0.013571101 | 434.73102 | 12.10432 | 0.08893291 | 12.01638 | 12.102364 | 12.194214 |
| LOC644907 | 1.274E-05 | 0.002441989 | 182.4321 | 13.180028 | 0.134878 | 13.224936 | 13.028425 | 13.286724 |
| LOC644914 | 0.002258979 | 0.068328686 | 1146.2343 | 10.770436 | 0.099619396 | 10.7338505 | 10.694284 | 10.883177 |
| LOC644928 | 0.001429296 | 0.051870294 | 957.6571 | 11.031903 | 0.053998057 | 11.093898 | 10.9951315 | 11.006682 |
| LOC644934 | 0.000329077 | 0.020758182 | 553.30133 | 11.774283 | 0.053196702 | 11.718109 | 11.780846 | 11.823894 |
| LOC645058 | 0.00093117 | 0.039785713 | 811.99133 | 11.264089 | 0.08165903 | 11.296241 | 11.324778 | 11.1712475 |
| LOC645138 | 1.39794E-05 | 0.002593583 | 186.84836 | 13.164291 | 0.09844993 | 13.1528225 | 13.267974 | 13.072079 |
| LOC645157 | 0.000474924 | 0.026030015 | 631.3926 | 11.580985 | 0.13396642 | 11.51299 | 11.494652 | 11.735313 |
| LOC645173 | 2.69787E-05 | 0.004069565 | 230.73448 | 12.930978 | 0.055174228 | 12.884043 | 12.917137 | 12.9917555 |
| LOC645174 | 3.17058E-07 | 0.000164179 | 62.065575 | 13.929256 | 0.020053882 | 13.9072275 | 13.934089 | 13.946452 |
| LOC645296 | 9.88644E-06 | 0.002053892 | 168.12566 | 13.26356 | 0.09910849 | 13.282868 | 13.156219 | 13.351594 |
| LOC645317 | 0.000184528 | 0.014290178 | 448.19998 | 12.068084 | 0.046540443 | 12.038645 | 12.043867 | 12.121739 |
| LOC645385 | 4.28893E-05 | 0.005573034 | 270.58173 | 12.752284 | 0.06963028 | 12.729018 | 12.830568 | 12.697266 |
| LOC645387 | 1.18753E-05 | 0.002354286 | 178.314 | 13.216374 | 0.08763714 | 13.284687 | 13.117563 | 13.246875 |
| LOC645436 | 0.000103793 | 0.009785326 | 365.15613 | 12.355949 | 0.074192375 | 12.297597 | 12.439446 | 12.330805 |
| LOC645452 | 0.001269701 | 0.04803817 | 914.24927 | 11.103188 | 0.13260631 | 11.138453 | 10.956514 | 11.214598 |
| LOC645683 | 3.45881E-07 | 0.000169014 | 65.86689 | 13.891561 | 0.11058126 | 14.018104 | 13.813509 | 13.843071 |
| LOC645688 | 8.71909E-05 | 0.008692529 | 344.0662 | 12.451916 | 0.06488442 | 12.523637 | 12.397292 | 12.434818 |
| LOC645715 | 0.005250562 | 0.11265492 | 1611.9915 | 10.284898 | 0.07608321 | 10.214222 | 10.365428 | 10.275043 |
| LOC645895 | 5.59175E-06 | 0.001337931 | 141.80519 | 13.445709 | 0.06226166 | 13.4486885 | 13.382011 | 13.506428 |
| LOC645899 | 5.18822E-06 | 0.001276596 | 137.54729 | 13.471196 | 0.115436934 | 13.440283 | 13.374365 | 13.598944 |
| LOC645979 | 0.00113475 | 0.044993144 | 875.71265 | 11.164062 | 0.06542659 | 11.208035 | 11.088873 | 11.195274 |
| LOC646195 | 2.24823E-06 | 0.000795918 | 101.47804 | 13.682884 | 0.16063282 | 13.547646 | 13.860438 | 13.640566 |
| LOC646200 | 1.42388E-05 | 0.0026 | 188.48622 | 13.166348 | 0.087011665 | 13.195058 | 13.068611 | 13.235379 |
| LOC646294 | 6.05292E-06 | 0.0014 | 145.88269 | 13.399849 | 0.11510716 | 13.293872 | 13.522309 | 13.383368 |
| LOC646347 | 0.003801263 | 0.09333404 | 1412.4075 | 10.474002 | 0.16973358 | 10.315164 | 10.453986 | 10.652856 |
| LOC646483 | 6.38151E-05 | 0.007141936 | 309.15134 | 12.588298 | 0.16903402 | 12.571318 | 12.765182 | 12.428395 |
| LOC646531 | 0.00012936 | 0.011448979 | 395.4896 | 12.241046 | 0.09657218 | 12.190395 | 12.352406 | 12.180334 |
| LOC646567 | 0.00765827 | 0.14006115 | 1886.3962 | 10.063365 | 0.0831035 | 10.05218 | 10.151493 | 9.986419 |
| LOC646630 | 3.90557E-05 | 0.005152091 | 261.50912 | 12.783206 | 0.15366757 | 12.6500845 | 12.748165 | 12.951367 |
| LOC646688 | 6.71586E-05 | 0.007396826 | 315.13474 | 12.569633 | 0.05250112 | 12.556696 | 12.524808 | 12.627392 |
| LOC646723 | 0 | 0 | 2.0800838 | 14.441513 | 0.08813314 | 14.378787 | 14.542277 | 14.403475 |
| LOC646766 | 2.88234E-08 | 2.43902E-05 | 46.63793 | 14.004418 | 0.09754472 | 13.9920845 | 13.913627 | 14.107543 |
| LOC646785 | 0.00273739 | 0.07727502 | 1236.5991 | 10.662364 | 0.07430924 | 10.583075 | 10.673603 | 10.7304125 |
| LOC646819 | 5.13057E-06 | 0.001280576 | 136.89455 | 13.476369 | 0.050233383 | 13.465687 | 13.432337 | 13.531085 |
| LOC646849 | 0.00088289 | 0.038384713 | 795.815 | 11.29214 | 0.032908678 | 11.28543 | 11.2631035 | 11.327887 |
| LOC646942 | 0.007908024 | 0.14326945 | 1911.9197 | 10.043816 | 0.048960995 | 10.064254 | 9.9879465 | 10.079246 |
| LOC646993 | 0.004376607 | 0.10190738 | 1495.7588 | 10.391759 | 0.13428982 | 10.30257 | 10.326499 | 10.546206 |
| LOC646996 | 0.007348562 | 0.13729186 | 1854.2324 | 10.084142 | 0.1380037 | 10.018561 | 9.991157 | 10.242707 |
| LOC647000 | 0.000433245 | 0.024480456 | 610.3561 | 11.639272 | 0.052467026 | 11.583152 | 11.687098 | 11.647568 |
| LOC647030 | 0.000425491 | 0.024120916 | 606.4985 | 11.644024 | 0.077687316 | 11.65356 | 11.562009 | 11.716503 |
| LOC647099 | 5.18822E-07 | 0.000222222 | 74.07966 | 13.852849 | 0.0647873 | 13.927398 | 13.820984 | 13.810165 |
| LOC647276 | 4.32351E-07 | 0.000189873 | 71.4056 | 13.878124 | 0.06415012 | 13.938013 | 13.885933 | 13.810428 |
| LOC647285 | 0.000765233 | 0.03497892 | 753.7786 | 11.36055 | 0.0881948 | 11.295112 | 11.325693 | 11.460847 |
| LOC647302 | 0.000296218 | 0.019575238 | 531.39874 | 11.835183 | 0.082912795 | 11.741093 | 11.866908 | 11.89755 |
| LOC647340 | 3.36658E-05 | 0.004767347 | 249.33665 | 12.860272 | 0.0758716 | 12.925965 | 12.777228 | 12.877625 |
| LOC647361 | 2.88234E-08 | 2.43902E-05 | 46.383686 | 14.013702 | 0.025453553 | 13.986335 | 14.036668 | 14.018104 |
| LOC647673 | 0.007717012 | 0.14076446 | 1892.5013 | 10.054645 | 0.08008577 | 10.12567 | 10.070417 | 9.967846 |
| LOC647856 | 0.008090679 | 0.14543937 | 1929.9843 | 10.029285 | 0.05689013 | 10.079152 | 10.041385 | 9.967319 |
| LOC647954 | 0.000549259 | 0.028872726 | 666.8633 | 11.514424 | 0.04042953 | 11.485395 | 11.560602 | 11.497277 |
| LOC648000 | 3.83352E-06 | 0.001072581 | 123.10375 | 13.557304 | 0.16729824 | 13.606548 | 13.370911 | 13.694453 |
| LOC648024 | 0.000658241 | 0.03211955 | 712.70764 | 11.429139 | 0.07410613 | 11.489085 | 11.45205 | 11.346284 |
| LOC648210 | 0.000146654 | 0.012470588 | 412.66156 | 12.177041 | 0.011438031 | 12.172066 | 12.168934 | 12.1901245 |
| LOC648294 | 0.004991757 | 0.10988832 | 1578.8967 | 10.313914 | 0.10230466 | 10.248164 | 10.261797 | 10.431784 |
| LOC648390 | 0.000158587 | 0.012976415 | 423.96683 | 12.145679 | 0.059290525 | 12.209816 | 12.134354 | 12.092869 |
| LOC648622 | 4.05834E-05 | 0.005313207 | 265.55692 | 12.780083 | 0.153675 | 12.9021635 | 12.607515 | 12.830568 |
| LOC648638 | 0.006973598 | 0.13366961 | 1814.1342 | 10.119283 | 0.09326391 | 10.150372 | 10.19303 | 10.014444 |
| LOC648729 | 0 | 0 | 24.384995 | 14.125756 | 0.12798586 | 14.011901 | 14.101088 | 14.264281 |
| LOC648771 | 4.92881E-06 | 0.001266667 | 133.2487 | 13.499309 | 0.047652025 | 13.531085 | 13.444518 | 13.522324 |
| LOC649049 | 1.421E-05 | 0.00262234 | 188.41098 | 13.153432 | 0.120457806 | 13.044615 | 13.282868 | 13.132812 |
| LOC649076 | 2.88234E-08 | 2.43902E-05 | 35.765972 | 14.056745 | 0.1024571 | 13.97437 | 14.1714735 | 14.024393 |
| LOC649150 | 0.003206434 | 0.08472506 | 1317.504 | 10.570483 | 0.04685946 | 10.554574 | 10.623225 | 10.5336485 |
| LOC649447 | 0.000217473 | 0.015951375 | 475.40442 | 11.987386 | 0.17971222 | 11.993098 | 11.804885 | 12.164173 |
| LOC649548 | 2.88234E-08 | 2.43902E-05 | 46.64988 | 14.010619 | 0.060678147 | 14.070646 | 14.011901 | 13.94931 |
| LOC649821 | 0.000150055 | 0.012605327 | 416.10696 | 12.173192 | 0.07975112 | 12.247965 | 12.182356 | 12.089254 |
| LOC650152 | 4.70687E-05 | 0.005832143 | 278.7402 | 12.707977 | 0.117408596 | 12.840031 | 12.668522 | 12.615377 |
| LOC650215 | 0.007150112 | 0.13533333 | 1833.0671 | 10.102864 | 0.19211468 | 9.960802 | 10.321448 | 10.026343 |
| LOC650276 | 2.30587E-07 | 0.000148148 | 54.866673 | 13.96498 | 0.09800903 | 13.883904 | 13.937136 | 14.073897 |
| LOC650369 | 0.003218712 | 0.08492015 | 1319.5161 | 10.5714 | 0.0674936 | 10.584459 | 10.498331 | 10.63141 |
| LOC650518 | 8.50579E-05 | 0.008603498 | 341.22565 | 12.463273 | 0.039955687 | 12.503834 | 12.462036 | 12.423951 |
| LOC650646 | 1.11258E-05 | 0.002284024 | 174.0785 | 13.232495 | 0.1932727 | 13.051786 | 13.436263 | 13.209437 |
| LOC651064 | 0.000792615 | 0.035666667 | 764.19977 | 11.343407 | 0.030555772 | 11.31102 | 11.347479 | 11.371723 |
| LOC651149 | 0.009812273 | 0.16366683 | 2092.122 | 9.913482 | 0.183236 | 10.04815 | 9.987475 | 9.704819 |
| LOC651202 | 4.34369E-05 | 0.00560223 | 271.68228 | 12.7523575 | 0.10553448 | 12.751044 | 12.647486 | 12.858542 |
| LOC651436 | 4.15057E-06 | 0.001125 | 126.31372 | 13.53413 | 0.11767212 | 13.577978 | 13.623584 | 13.40083 |
| LOC651816 | 0.003409494 | 0.08762148 | 1350.8369 | 10.534415 | 0.06852055 | 10.590196 | 10.45793 | 10.555119 |
| LOC651894 | 4.72128E-05 | 0.005829182 | 279.0281 | 12.711698 | 0.06484965 | 12.757112 | 12.637429 | 12.740555 |
| LOC652071 | 3.74705E-07 | 0.000178082 | 67.20646 | 13.906035 | 0.013361601 | 13.910309 | 13.89106 | 13.916738 |
| LOC652595 | 0.002004035 | 0.06338013 | 1093.124 | 10.840449 | 0.15618277 | 10.662946 | 10.901591 | 10.956812 |
| LOC652624 | 9.03326E-05 | 0.008928775 | 348.11273 | 12.434636 | 0.061715484 | 12.473547 | 12.363478 | 12.466886 |
| LOC653079 | 0.009750043 | 0.16309932 | 2086.4917 | 9.913544 | 0.038860146 | 9.957619 | 9.898801 | 9.884213 |
| LOC653147 | 0.003724217 | 0.09242346 | 1400.4608 | 10.486031 | 0.056411903 | 10.451878 | 10.455071 | 10.551144 |
| LOC653156 | 0.000351444 | 0.021734403 | 565.7617 | 11.738625 | 0.034413557 | 11.7045 | 11.738053 | 11.77332 |
| LOC653162 | 0.003565372 | 0.089700505 | 1376.126 | 10.507828 | 0.08322615 | 10.457555 | 10.603894 | 10.462034 |
| LOC653226 | 0.000412204 | 0.02359901 | 599.4551 | 11.664418 | 0.03461272 | 11.627535 | 11.669531 | 11.696192 |
| LOC653232 | 3.57411E-06 | 0.001016393 | 120.553 | 13.569255 | 0.13594232 | 13.421391 | 13.688821 | 13.597552 |
| LOC653314 | 3.97763E-05 | 0.005227273 | 263.75244 | 12.774152 | 0.05420261 | 12.753066 | 12.835729 | 12.733661 |
| LOC653377 | 0.004123652 | 0.098394774 | 1459.9646 | 10.425575 | 0.17581433 | 10.452074 | 10.238016 | 10.586637 |
| LOC653505 | 0.000197527 | 0.014995623 | 458.77216 | 12.038803 | 0.07641937 | 11.964598 | 12.117259 | 12.034553 |
| LOC653506 | 0.00041082 | 0.023558678 | 598.8486 | 11.666046 | 0.0750173 | 11.683781 | 11.730606 | 11.58375 |
| LOC653566 | 0.004776763 | 0.106850415 | 1550.7777 | 10.341057 | 0.05180369 | 10.353819 | 10.284065 | 10.385287 |
| LOC653658 | 1.82164E-05 | 0.003082927 | 203.81758 | 13.070656 | 0.06135383 | 13.121472 | 13.087997 | 13.002498 |
| LOC653737 | 0.000367499 | 0.022329247 | 575.2967 | 11.714444 | 0.05355919 | 11.654335 | 11.757097 | 11.731902 |
| LOC653773 | 4.36098E-05 | 0.0055625 | 272.1999 | 12.738252 | 0.103174366 | 12.843409 | 12.734165 | 12.637181 |
| LOC653874 | 0.005672019 | 0.11826022 | 1664.2367 | 10.243865 | 0.17010641 | 10.279143 | 10.393566 | 10.058886 |
| LOC653881 | 1.84182E-05 | 0.003086957 | 204.57292 | 13.065094 | 0.103212975 | 12.951367 | 13.1528225 | 13.09109 |
| LOC654121 | 0.002796161 | 0.07829701 | 1247.0557 | 10.653121 | 0.15686439 | 10.508654 | 10.6307335 | 10.819977 |
| LOC654194 | 9.79997E-07 | 0.000377778 | 85.63465 | 13.779132 | 0.022316366 | 13.754895 | 13.783671 | 13.79883 |
| LOC727803 | 0.004253127 | 0.100039326 | 1478.2131 | 10.410949 | 0.1203609 | 10.361268 | 10.548198 | 10.323381 |
| LOC727808 | 4.75587E-06 | 0.00125 | 131.62128 | 13.500539 | 0.10819206 | 13.472908 | 13.408842 | 13.619867 |
| LOC727865 | 0.000595492 | 0.03056213 | 686.9435 | 11.476878 | 0.022558162 | 11.451888 | 11.483011 | 11.495736 |
| LOC728006 | 0.00557912 | 0.1170974 | 1653.3529 | 10.248814 | 0.10423467 | 10.322785 | 10.129602 | 10.294053 |
| LOC728031 | 0.000150141 | 0.012582125 | 416.2435 | 12.15949 | 0.097332574 | 12.100258 | 12.106387 | 12.271824 |
| LOC728126 | 1.31435E-05 | 0.002491803 | 184.01367 | 13.188583 | 0.14903098 | 13.117955 | 13.359798 | 13.087997 |
| LOC728128 | 0.003588084 | 0.0901412 | 1379.7333 | 10.507256 | 0.041529126 | 10.474732 | 10.554035 | 10.492999 |
| LOC728244 | 3.48475E-05 | 0.004836 | 251.91833 | 12.842755 | 0.039097942 | 12.887068 | 12.813119 | 12.828079 |
| LOC728324 | 0.00982092 | 0.16357513 | 2092.789 | 9.908531 | 0.08210103 | 9.921519 | 9.820711 | 9.983365 |
| LOC728368 | 0.001887387 | 0.061197195 | 1068.2017 | 10.872993 | 0.04785752 | 10.858473 | 10.834077 | 10.926429 |
| LOC728428 | 4.74434E-05 | 0.005836879 | 279.53665 | 12.701233 | 0.12758532 | 12.663575 | 12.843409 | 12.596715 |
| LOC728453 | 0.000122413 | 0.011002591 | 387.61005 | 12.259274 | 0.030508924 | 12.2850485 | 12.225589 | 12.267184 |
| LOC728481 | 2.30587E-06 | 0.000792079 | 102.97753 | 13.671351 | 0.009814519 | 13.682684 | 13.665685 | 13.665685 |
| LOC728484 | 0.008428864 | 0.14889562 | 1962.8995 | 10.0024805 | 0.11100268 | 9.982502 | 9.902825 | 10.122117 |
| LOC728492 | 0.001208826 | 0.046495564 | 897.1311 | 11.125529 | 0.119491234 | 11.182411 | 10.988223 | 11.205952 |
| LOC728517 | 0 | 0 | 30.48687 | 14.086906 | 0.11425319 | 14.027154 | 14.218645 | 14.014919 |
| LOC728553 | 7.3788E-06 | 0.001651613 | 153.88602 | 13.355536 | 0.035700336 | 13.315433 | 13.367326 | 13.383851 |
| LOC728576 | 0 | 0 | 22.250578 | 14.150726 | 0.03967725 | 14.14838 | 14.191525 | 14.112275 |
| LOC728590 | 2.93999E-05 | 0.004340426 | 238.25928 | 12.89402 | 0.040108163 | 12.894236 | 12.853804 | 12.934019 |
| LOC728620 | 0.001246757 | 0.04737678 | 907.87225 | 11.1154585 | 0.07016302 | 11.120786 | 11.042783 | 11.182805 |
| LOC728643 | 0.00088315 | 0.038347933 | 795.86755 | 11.291306 | 0.089500986 | 11.240179 | 11.39465 | 11.239087 |
| LOC728658 | 0 | 0 | 14.187942 | 14.206181 | 0.018001411 | 14.211236 | 14.221113 | 14.186192 |
| LOC728661 | 0.007551623 | 0.13913755 | 1875.3694 | 10.067748 | 0.05130881 | 10.125568 | 10.050031 | 10.027647 |
| LOC728666 | 0.00734297 | 0.13726132 | 1853.5616 | 10.086983 | 0.03729537 | 10.069766 | 10.061405 | 10.129776 |
| LOC728672 | 1.17023E-05 | 0.002333333 | 177.07814 | 13.209396 | 0.06880897 | 13.198854 | 13.146467 | 13.282868 |
| LOC728693 | 0.006093417 | 0.12413682 | 1714.3568 | 10.195881 | 0.03887273 | 10.183092 | 10.165014 | 10.239537 |
| LOC728782 | 2.98322E-05 | 0.004367088 | 239.23663 | 12.894986 | 0.05331103 | 12.926205 | 12.83343 | 12.925324 |
| LOC728809 | 0.000740993 | 0.034553763 | 744.64374 | 11.372003 | 0.08611983 | 11.46364 | 11.292739 | 11.359631 |
| LOC728820 | 0.000950193 | 0.04020244 | 817.6765 | 11.254357 | 0.065372504 | 11.179937 | 11.280623 | 11.302512 |
| LOC728823 | 0.002917738 | 0.079769894 | 1268.8837 | 10.630364 | 0.15841876 | 10.613976 | 10.480779 | 10.796342 |
| LOC728873 | 0.000115524 | 0.010547369 | 378.8748 | 12.300378 | 0.08790682 | 12.39259 | 12.291021 | 12.217525 |
| LOC728877 | 0.001583242 | 0.055316214 | 996.8474 | 10.976776 | 0.06226418 | 11.041205 | 10.91693 | 10.972195 |
| LOC728888 | 0.003549144 | 0.089552 | 1373.4917 | 10.51179 | 0.12415336 | 10.62184 | 10.536331 | 10.377198 |
| LOC728937 | 2.53646E-06 | 0.000838095 | 105.653076 | 13.659888 | 0.07113703 | 13.741484 | 13.62728 | 13.6109 |
| LOC728973 | 5.36116E-05 | 0.006391753 | 291.40552 | 12.670303 | 0.06884598 | 12.736959 | 12.599459 | 12.674491 |
| LOC729009 | 0.000372312 | 0.022464348 | 578.0051 | 11.716474 | 0.12824233 | 11.692616 | 11.601837 | 11.854971 |
| LOC729082 | 0.007676168 | 0.14031453 | 1888.1935 | 10.059926 | 0.050351035 | 10.102724 | 10.004447 | 10.072609 |
| LOC729102 | 7.03868E-05 | 0.007560371 | 320.32175 | 12.542157 | 0.1003608 | 12.485688 | 12.658032 | 12.482754 |
| LOC729148 | 0.004798841 | 0.10713707 | 1553.6776 | 10.3359 | 0.092036076 | 10.281589 | 10.442165 | 10.283946 |
| LOC729200 | 0.006817173 | 0.1319102 | 1797.0338 | 10.124864 | 0.07410974 | 10.1226845 | 10.0518675 | 10.200039 |
| LOC729208 | 0.001836917 | 0.060522318 | 1057.0916 | 10.887326 | 0.033943057 | 10.880108 | 10.8575735 | 10.924298 |
| LOC729236 | 0.000715225 | 0.033714674 | 735.0944 | 11.3938875 | 0.038196664 | 11.413808 | 11.349848 | 11.418005 |
| LOC729279 | 0.00179276 | 0.059405923 | 1046.6382 | 10.9117 | 0.121420376 | 10.8575735 | 11.05077 | 10.826755 |
| LOC729301 | 1.76976E-05 | 0.003009804 | 202.06357 | 13.088939 | 0.08541828 | 13.034794 | 13.044615 | 13.187408 |
| LOC729317 | 0.001689629 | 0.057583496 | 1022.26227 | 10.94291 | 0.13583705 | 10.975018 | 10.793897 | 11.059818 |
| LOC729324 | 0.005459446 | 0.11528302 | 1638.5623 | 10.263562 | 0.072677046 | 10.189373 | 10.266689 | 10.334626 |
| LOC729340 | 0.001794633 | 0.05941126 | 1047.0957 | 10.902274 | 0.07401873 | 10.8899145 | 10.835213 | 10.981694 |
| LOC729402 | 0.00114135 | 0.045100227 | 877.4215 | 11.16071 | 0.079712495 | 11.238227 | 11.07897 | 11.164936 |
| LOC729439 | 1.15294E-07 | 7.84314E-05 | 51.044685 | 13.960826 | 0.14305413 | 13.860438 | 13.897411 | 14.124624 |
| LOC729466 | 1.7467E-05 | 0.002985222 | 200.5889 | 13.089458 | 0.123444445 | 13.2027645 | 12.957907 | 13.107705 |
| LOC729500 | 0.002375915 | 0.07045299 | 1168.9915 | 10.74162 | 0.10479639 | 10.744717 | 10.844834 | 10.63531 |
| LOC729603 | 5.26604E-05 | 0.0063218 | 289.98257 | 12.663994 | 0.06690043 | 12.684897 | 12.589137 | 12.717946 |
| LOC729608 | 0.002426846 | 0.07147453 | 1178.8232 | 10.731972 | 0.12218692 | 10.872333 | 10.674189 | 10.649393 |
| LOC729617 | 3.51646E-06 | 0.00106087 | 117.640366 | 13.598549 | 0.014641115 | 13.5900955 | 13.5900955 | 13.615455 |
| LOC729646 | 0.003198334 | 0.08470458 | 1316.118 | 10.569373 | 0.10010234 | 10.523657 | 10.684171 | 10.50029 |
| LOC729679 | 2.42117E-05 | 0.003716814 | 223.6072 | 12.958382 | 0.10321181 | 13.072079 | 12.87059 | 12.932478 |
| LOC729708 | 0.007578428 | 0.13940933 | 1878.1335 | 10.069715 | 0.05169864 | 10.103245 | 10.095721 | 10.010176 |
| LOC729742 | 3.52511E-05 | 0.00487251 | 252.98814 | 12.829898 | 0.012539768 | 12.833787 | 12.815873 | 12.840031 |
| LOC729768 | 0.001179714 | 0.04603937 | 888.9078 | 11.147057 | 0.07428134 | 11.148132 | 11.072243 | 11.220794 |
| LOC729769 | 0.000612642 | 0.03116569 | 694.4667 | 11.459065 | 0.1744485 | 11.459544 | 11.284378 | 11.633274 |
| LOC729789 | 4.0641E-06 | 0.001119048 | 125.12265 | 13.538879 | 0.09724314 | 13.649221 | 13.501732 | 13.465687 |
| LOC729798 | 0.004962414 | 0.10938119 | 1575.0089 | 10.322261 | 0.04811259 | 10.375638 | 10.282231 | 10.308913 |
| LOC729816 | 0.000787744 | 0.035585936 | 762.27216 | 11.348109 | 0.052242447 | 11.39804 | 11.293827 | 11.35246 |
| LOC729841 | 0.001115178 | 0.044728324 | 869.4752 | 11.174502 | 0.053612106 | 11.170146 | 11.23016 | 11.123201 |
| LOC729843 | 0.005154522 | 0.111629836 | 1599.8756 | 10.296198 | 0.077492505 | 10.301463 | 10.2162075 | 10.370924 |
| LOC729903 | 2.88234E-08 | 2.43902E-05 | 39.612934 | 14.031104 | 0.14548436 | 14.080067 | 14.14579 | 13.867455 |
| LOC729978 | 0.001934917 | 0.062215015 | 1078.6029 | 10.862227 | 0.0732351 | 10.930351 | 10.784775 | 10.871554 |
| LOC730004 | 0.006425146 | 0.12759817 | 1753.0668 | 10.162991 | 0.08468465 | 10.11398 | 10.114215 | 10.260776 |
| LOC730029 | 0.000648671 | 0.03183168 | 708.7995 | 11.435661 | 0.15844768 | 11.454746 | 11.268535 | 11.583701 |
| LOC730052 | 0.005028362 | 0.11027434 | 1583.7987 | 10.310933 | 0.100191094 | 10.2413025 | 10.265736 | 10.42576 |
| LOC730187 | 3.54528E-06 | 0.001042373 | 118.53069 | 13.589587 | 0.083243296 | 13.522324 | 13.563751 | 13.682684 |
| LOC730246 | 1.29994E-05 | 0.002478022 | 183.58508 | 13.181686 | 0.055582386 | 13.149596 | 13.149596 | 13.245868 |
| LOC730255 | 0.001006543 | 0.04192197 | 835.56866 | 11.230947 | 0.050529975 | 11.2217865 | 11.185623 | 11.28543 |
| LOC730278 | 0.00022537 | 0.016289582 | 481.65677 | 11.979346 | 0.016030995 | 11.961114 | 11.991236 | 11.985687 |
| LOC730313 | 0.000453796 | 0.0251904 | 621.4847 | 11.61106 | 0.09869635 | 11.650691 | 11.498708 | 11.683781 |
| LOC730316 | 0.002003257 | 0.06341332 | 1092.9521 | 10.842187 | 0.01765306 | 10.824143 | 10.842996 | 10.859422 |
| LOC730432 | 0.001878423 | 0.061192486 | 1066.1873 | 10.876553 | 0.087487064 | 10.932174 | 10.921775 | 10.77571 |
| LOC730534 | 5.67822E-06 | 0.001340136 | 143.45628 | 13.42172 | 0.057488937 | 13.374365 | 13.485685 | 13.40511 |
| LOC730740 | 4.38404E-05 | 0.005551095 | 272.56744 | 12.749499 | 0.028816719 | 12.723053 | 12.745231 | 12.7802105 |
| LOC730744 | 0.005225457 | 0.11246402 | 1608.7549 | 10.289416 | 0.09092172 | 10.393566 | 10.248803 | 10.22588 |
| LOC730754 | 0 | 0 | 19.389912 | 14.167245 | 0.040834185 | 14.120974 | 14.19823 | 14.182534 |
| LOC731049 | 0.001171182 | 0.04580947 | 886.46265 | 11.148895 | 0.037256077 | 11.127385 | 11.127385 | 11.191915 |
| LOC731096 | 5.21704E-06 | 0.001274648 | 137.63017 | 13.476215 | 0.10031808 | 13.510255 | 13.363307 | 13.555085 |
| LOC731314 | 0.002322967 | 0.069596715 | 1158.4698 | 10.758686 | 0.07005188 | 10.683289 | 10.821755 | 10.771015 |
| LOC731365 | 8.79115E-06 | 0.001871166 | 160.51614 | 13.333859 | 0.059050955 | 13.382011 | 13.351594 | 13.267974 |
| LOC731640 | 7.93797E-05 | 0.00827027 | 333.06787 | 12.489507 | 0.10518407 | 12.544033 | 12.5562315 | 12.368255 |
| LOC731985 | 5.13633E-05 | 0.006209059 | 287.5996 | 12.687104 | 0.040755168 | 12.734165 | 12.663575 | 12.663575 |
| LOC91561 | 0.000136681 | 0.011825437 | 402.73068 | 12.207179 | 0.059016522 | 12.2153845 | 12.144488 | 12.2616625 |
| LPP | 0.007459849 | 0.13818046 | 1865.906 | 10.0774 | 0.013375353 | 10.0908 | 10.06405 | 10.07735 |
| LRAP | 5.57733E-05 | 0.006559322 | 295.53796 | 12.638119 | 0.21962994 | 12.815873 | 12.39259 | 12.7058935 |
| LRP10 | 0.002934946 | 0.07992543 | 1271.9221 | 10.617756 | 0.0721332 | 10.646275 | 10.67127 | 10.535724 |
| LRRC37B2 | 0.005330432 | 0.11366564 | 1622.2687 | 10.276916 | 0.09561541 | 10.333985 | 10.16653 | 10.330232 |
| LSM1 | 0.000822592 | 0.03672973 | 774.9415 | 11.324199 | 0.18531473 | 11.219799 | 11.214635 | 11.53816 |
| LSM2 | 0.000392085 | 0.022978041 | 588.7905 | 11.683251 | 0.075329825 | 11.643569 | 11.770127 | 11.63606 |
| LSM3 | 0.000163544 | 0.013164734 | 428.61038 | 12.125577 | 0.03305778 | 12.115215 | 12.098942 | 12.162575 |
| LSM4 | 0.002881882 | 0.079541765 | 1262.6719 | 10.632807 | 0.07951864 | 10.546856 | 10.703756 | 10.647808 |
| LSM5 | 0.001639822 | 0.056328714 | 1010.4134 | 10.958306 | 0.13486306 | 11.068263 | 10.807829 | 10.99883 |
| LSM7 | 0.002409667 | 0.07114979 | 1175.5999 | 10.738616 | 0.021020854 | 10.724187 | 10.728926 | 10.762733 |
| LSMD1 | 0.00919084 | 0.15661444 | 2035.7823 | 9.951931 | 0.04458604 | 10.002922 | 9.920283 | 9.932588 |
| LTA4H | 0.001396466 | 0.051160507 | 949.3582 | 11.044463 | 0.1273253 | 11.100012 | 11.134576 | 10.898801 |
| LTV1 | 0.007574854 | 0.1394175 | 1877.7722 | 10.072342 | 0.073211834 | 10.011725 | 10.153682 | 10.05162 |
| LXN | 0.000239926 | 0.016918698 | 493.0682 | 11.944894 | 0.084717974 | 11.848547 | 12.007732 | 11.978403 |
| LY6E | 0.001599729 | 0.055556558 | 1000.96564 | 10.969098 | 0.20941715 | 10.817422 | 11.208035 | 10.881838 |
| LYPLAL1 | 0.008788811 | 0.15268853 | 1997.6644 | 9.978801 | 0.108004145 | 9.936239 | 10.101601 | 9.898561 |
| LYRM1 | 0.003694616 | 0.09195194 | 1395.8499 | 10.491613 | 0.09201995 | 10.580853 | 10.397045 | 10.4969425 |
| LYRM2 | 0.008796277 | 0.15274175 | 1998.4443 | 9.976944 | 0.046620604 | 9.9277935 | 10.020536 | 9.982502 |
| M6PRBP1 | 0.001094743 | 0.044163954 | 863.25525 | 11.183749 | 0.09557691 | 11.07432 | 11.22606 | 11.250868 |
| MAD2L2 | 0.009944025 | 0.16444138 | 2103.8735 | 9.90175 | 0.0570446 | 9.836565 | 9.942545 | 9.92614 |
| MAGMAS | 0.007635125 | 0.14000687 | 1884.0731 | 10.064237 | 0.06817623 | 10.007532 | 10.045299 | 10.13988 |
| MAGT1 | 0.005700265 | 0.11856415 | 1667.7673 | 10.238014 | 0.087267175 | 10.191863 | 10.183513 | 10.338666 |
| MAL2 | 0.001296795 | 0.04890326 | 921.65356 | 11.090317 | 0.009560732 | 11.098957 | 11.080046 | 11.091949 |
| MAN1B1 | 0.006838387 | 0.13224694 | 1799.3937 | 10.1304865 | 0.07207718 | 10.0476 | 10.165417 | 10.178444 |
| MAN2B2 | 0.005303107 | 0.11336168 | 1618.7147 | 10.278705 | 0.15485303 | 10.23704 | 10.148947 | 10.450128 |
| MAP2K1 | 0.00941033 | 0.15902679 | 2055.854 | 9.936104 | 0.06749954 | 9.989032 | 9.86009 | 9.959189 |
| MAP3K1 | 0.002384764 | 0.070654996 | 1170.789 | 10.741832 | 0.05823131 | 10.794642 | 10.679382 | 10.751469 |
| MAPBPIP | 0.006865625 | 0.13240467 | 1802.3629 | 10.126689 | 0.09093792 | 10.143719 | 10.207909 | 10.02844 |
| MAPK3 | 0.004655618 | 0.10598557 | 1534.241 | 10.357844 | 0.07370122 | 10.421944 | 10.374273 | 10.277314 |
| MAPK6 | 0.001730443 | 0.05840078 | 1032.2677 | 10.920316 | 0.018015604 | 10.9410925 | 10.910826 | 10.909029 |
| MAPKAPK3 | 0.009894161 | 0.16408604 | 2099.4167 | 9.905724 | 0.102643594 | 10.004967 | 9.912216 | 9.799988 |
| MAPRE1 | 0.001209633 | 0.046475083 | 897.4347 | 11.12939 | 0.07947314 | 11.097811 | 11.219799 | 11.07056 |
| MARCKS | 0.00176996 | 0.05910202 | 1041.4202 | 10.911315 | 0.09404876 | 10.982665 | 10.804738 | 10.946541 |
| MARCKSL1 | 0.002161526 | 0.06595603 | 1125.9921 | 10.794632 | 0.13683271 | 10.645452 | 10.824143 | 10.914301 |
| MAT2A | 0.00129985 | 0.04891215 | 922.4548 | 11.094438 | 0.038546264 | 11.09103 | 11.05771 | 11.134576 |
| MCEE | 0.009763792 | 0.16301492 | 2087.6833 | 9.911422 | 0.06278215 | 9.940115 | 9.954731 | 9.83942 |
| MCM3 | 0.005602093 | 0.11722497 | 1656.179 | 10.246945 | 0.14166732 | 10.143528 | 10.408419 | 10.1888895 |
| MCM4 | 0.009245864 | 0.15701224 | 2040.832 | 9.946818 | 0.078813344 | 9.947797 | 10.025137 | 9.867519 |
| MCM6 | 0.000383092 | 0.0227976 | 583.76056 | 11.697398 | 0.15210475 | 11.830154 | 11.531432 | 11.730606 |
| MCTS1 | 0.006997809 | 0.13383792 | 1816.7806 | 10.11756 | 0.031179542 | 10.082292 | 10.141461 | 10.128926 |
| MDH1 | 0.000212285 | 0.015670212 | 470.77914 | 12.005338 | 0.13461164 | 11.850334 | 12.092869 | 12.072812 |
| MDH2 | 6.8888E-06 | 0.001562092 | 151.649 | 13.3592825 | 0.0816854 | 13.301268 | 13.452697 | 13.323884 |
| MED10 | 0.004821496 | 0.107228845 | 1556.6328 | 10.335689 | 0.006109441 | 10.334545 | 10.330232 | 10.342289 |
| MED20 | 0.004158557 | 0.09875222 | 1465.0637 | 10.420869 | 0.13838811 | 10.505899 | 10.495523 | 10.261185 |
| MED29 | 0.007401741 | 0.13769223 | 1859.9725 | 10.077634 | 0.13002412 | 10.171765 | 9.929273 | 10.131863 |
| MED6 | 0.001456592 | 0.052476637 | 965.00726 | 11.021179 | 0.09721004 | 11.027594 | 11.115022 | 10.920919 |
| MEPCE | 0.008280019 | 0.14739199 | 1948.3735 | 10.011553 | 0.13661346 | 10.137129 | 10.0314455 | 9.866083 |
| METAP1 | 0.003673027 | 0.091677696 | 1392.6298 | 10.494354 | 0.07125186 | 10.551077 | 10.414382 | 10.517606 |
| METAP2 | 0.001192195 | 0.046111483 | 892.2971 | 11.1296 | 0.22078875 | 11.266306 | 11.247608 | 10.874883 |
| METTL5 | 0.003562057 | 0.08968215 | 1375.529 | 10.511807 | 0.03144968 | 10.48826 | 10.5475235 | 10.4996395 |
| MFSD1 | 0.007270681 | 0.13657229 | 1846.0159 | 10.093607 | 0.11044392 | 9.978887 | 10.199209 | 10.102724 |
| MFSD10 | 0.001072347 | 0.043615475 | 856.2904 | 11.196971 | 0.019514084 | 11.196382 | 11.216773 | 11.177758 |
| MGC16703 | 0.005066784 | 0.110627435 | 1588.6364 | 10.306125 | 0.06589984 | 10.298207 | 10.24454 | 10.375625 |
| MGC71993 | 0.008845765 | 0.15321767 | 2003.3586 | 9.974141 | 0.03303595 | 9.941472 | 9.973419 | 10.007532 |
| MGC87895 | 0.007327348 | 0.13711704 | 1851.9231 | 10.084338 | 0.15426303 | 9.963988 | 10.030785 | 10.258241 |
| MGEA5 | 0.003477287 | 0.08844648 | 1361.8348 | 10.524705 | 0.07717026 | 10.585233 | 10.437806 | 10.551077 |
| MGMT | 0.004090506 | 0.09787311 | 1455.0569 | 10.430678 | 0.09868715 | 10.317183 | 10.496265 | 10.478588 |
| MGP | 0.004816078 | 0.107314706 | 1556.0009 | 10.336067 | 0.06351345 | 10.263044 | 10.366684 | 10.378472 |
| MIF | 1.72652E-05 | 0.002965346 | 199.79813 | 13.095245 | 0.04768209 | 13.143285 | 13.047929 | 13.094524 |
| MIR1974 | 0.001521618 | 0.05397853 | 981.8416 | 10.997851 | 0.4505287 | 11.371723 | 10.497641 | 11.124189 |
| MIR1978 | 0.003756903 | 0.092704125 | 1405.3895 | 10.481431 | 0.056441803 | 10.49282 | 10.420164 | 10.531311 |
| MIS12 | 0.004186776 | 0.09921858 | 1469.0282 | 10.417981 | 0.11005444 | 10.475912 | 10.486971 | 10.291062 |
| MKI67IP | 0.006846573 | 0.13225779 | 1800.2935 | 10.128564 | 0.03644852 | 10.087185 | 10.142595 | 10.155911 |
| MKRN1 | 0.00152205 | 0.053938713 | 981.9597 | 10.992789 | 0.109531716 | 10.935778 | 11.119067 | 10.923522 |
| MLPH | 0.000307488 | 0.019940186 | 539.54034 | 11.807563 | 0.040478136 | 11.760909 | 11.833345 | 11.828435 |
| MMADHC | 0.00114429 | 0.04516496 | 878.4214 | 11.15923 | 0.0791008 | 11.239087 | 11.157696 | 11.080908 |
| MOBKL1B | 0.006098663 | 0.12409795 | 1715.0107 | 10.191972 | 0.23941255 | 10.122117 | 9.995256 | 10.458543 |
| MORF4L2 | 0.000908399 | 0.03910174 | 804.5472 | 11.27848 | 0.07996845 | 11.358465 | 11.198528 | 11.278443 |
| MRFAP1 | 0.000316135 | 0.020198895 | 544.9772 | 11.802449 | 0.13030341 | 11.736714 | 11.718109 | 11.952528 |
| MRLC2 | 1.90523E-05 | 0.00316268 | 207.2289 | 13.039044 | 0.08179046 | 13.037752 | 13.121472 | 12.957907 |
| MRPL13 | 0.001234738 | 0.047074724 | 904.6873 | 11.117859 | 0.109123416 | 11.158734 | 11.2006445 | 10.9942 |
| MRPL14 | 0.002429527 | 0.07137172 | 1179.3689 | 10.732162 | 0.04358709 | 10.716597 | 10.698496 | 10.781396 |
| MRPL15 | 0.004563988 | 0.10486291 | 1521.5833 | 10.366754 | 0.12501739 | 10.2495365 | 10.352393 | 10.498331 |
| MRPL16 | 0.008727561 | 0.15231086 | 1991.6366 | 9.984214 | 0.012991464 | 9.969406 | 9.989535 | 9.993699 |
| MRPL17 | 0.001758921 | 0.05907454 | 1038.7489 | 10.912185 | 0.03506044 | 10.952268 | 10.887218 | 10.897068 |
| MRPL18 | 0.003438001 | 0.08789831 | 1355.6101 | 10.533802 | 0.006478526 | 10.534135 | 10.5271635 | 10.540108 |
| MRPL19 | 0.007140399 | 0.13537104 | 1832.0109 | 10.10233 | 0.09644223 | 10.009029 | 10.096328 | 10.201633 |
| MRPL20 | 0.007738946 | 0.14101627 | 1894.7844 | 10.054092 | 0.03843127 | 10.097455 | 10.040578 | 10.024243 |
| MRPL22 | 0.002105436 | 0.064929776 | 1114.8811 | 10.816406 | 0.08403205 | 10.727353 | 10.894302 | 10.827564 |
| MRPL23 | 0.006707644 | 0.13037255 | 1784.819 | 10.140252 | 0.028778933 | 10.10728 | 10.160324 | 10.1531515 |
| MRPL24 | 0.003741166 | 0.09271143 | 1402.8948 | 10.480502 | 0.10483219 | 10.439985 | 10.599546 | 10.401976 |
| MRPL32 | 0.001307258 | 0.04903135 | 924.6285 | 11.079051 | 0.16880687 | 10.984442 | 10.978766 | 11.273945 |
| MRPL33 | 0.000405171 | 0.02338935 | 595.6192 | 11.669155 | 0.054127462 | 11.606695 | 11.702347 | 11.698423 |
| MRPL36 | 0.000844469 | 0.03713308 | 782.56244 | 11.311859 | 0.07102409 | 11.369418 | 11.232487 | 11.333673 |
| MRPL37 | 0.000747651 | 0.03463151 | 747.1562 | 11.366958 | 0.12830886 | 11.514432 | 11.305532 | 11.280908 |
| MRPL41 | 0.000480256 | 0.02623937 | 633.85565 | 11.586751 | 0.045727845 | 11.617427 | 11.608633 | 11.534194 |
| MRPL46 | 0.007560789 | 0.13923249 | 1876.3661 | 10.072616 | 0.032889552 | 10.066255 | 10.108221 | 10.043371 |
| MRPL50 | 0.001801378 | 0.059577692 | 1048.8992 | 10.90536 | 0.12488764 | 10.795527 | 10.879347 | 11.041205 |
| MRPL51 | 0.000341471 | 0.021193203 | 560.313 | 11.746114 | 0.022097683 | 11.754756 | 11.762584 | 11.721001 |
| MRPL54 | 0.00888217 | 0.15346514 | 2006.9879 | 9.9711275 | 0.031931326 | 9.971402 | 10.002922 | 9.939061 |
| MRPS10 | 0.003275408 | 0.085634515 | 1329.0492 | 10.554437 | 0.08803954 | 10.596028 | 10.613976 | 10.453307 |
| MRPS11 | 0.004461405 | 0.103189334 | 1507.7003 | 10.381412 | 0.040991303 | 10.345033 | 10.425825 | 10.373375 |
| MRPS15 | 0.007645069 | 0.14004119 | 1885.109 | 10.065274 | 0.052010387 | 10.019049 | 10.055183 | 10.121591 |
| MRPS17 | 0.002892604 | 0.079647616 | 1264.4921 | 10.6286955 | 0.089667946 | 10.720982 | 10.623208 | 10.541898 |
| MRPS21 | 0.000766761 | 0.034956638 | 754.3006 | 11.360519 | 0.06571132 | 11.289958 | 11.371638 | 11.419962 |
| MRPS22 | 0.004595694 | 0.10524291 | 1526.0693 | 10.36143 | 0.14162163 | 10.320794 | 10.518929 | 10.24457 |
| MRPS23 | 0.002554332 | 0.07391159 | 1202.8953 | 10.698665 | 0.13914035 | 10.644803 | 10.856684 | 10.594506 |
| MRPS24 | 0.001183202 | 0.04607183 | 889.8177 | 11.142947 | 0.07749489 | 11.069541 | 11.135332 | 11.2239685 |
| MRPS26 | 0.009634635 | 0.16155824 | 2075.92 | 9.920124 | 0.09868614 | 9.976364 | 9.977834 | 9.806174 |
| MRPS28 | 0.007711593 | 0.14081368 | 1891.8922 | 10.060047 | 0.04127757 | 10.060465 | 10.101113 | 10.018561 |
| MRPS30 | 0.006501384 | 0.12823138 | 1761.5951 | 10.159031 | 0.066791825 | 10.096328 | 10.229272 | 10.151493 |
| MRPS6 | 0.000885715 | 0.03841125 | 796.66846 | 11.290708 | 0.07616464 | 11.2314005 | 11.376602 | 11.26412 |
| MRPS7 | 0.006475039 | 0.12807582 | 1758.5449 | 10.159772 | 0.041480843 | 10.112932 | 10.1745205 | 10.191863 |
| MSH3 | 0.000935407 | 0.03991759 | 813.2426 | 11.265014 | 0.025797348 | 11.291672 | 11.240173 | 11.263194 |
| MSH6 | 0.000940191 | 0.040023312 | 814.70526 | 11.264229 | 0.09765779 | 11.243254 | 11.178763 | 11.37067 |
| MSRB2 | 0.001201361 | 0.04631111 | 894.90045 | 11.13107 | 0.12742703 | 11.155649 | 11.244417 | 10.993144 |
| MST4 | 0.008548366 | 0.15039402 | 1974.5627 | 9.995549 | 0.10537265 | 10.051633 | 9.873997 | 10.06102 |
| MT1A | 0.005411973 | 0.11476956 | 1632.5564 | 10.264714 | 0.11727593 | 10.148384 | 10.382914 | 10.262844 |
| MT2A | 0.003355133 | 0.08660938 | 1342.1862 | 10.545489 | 0.1758634 | 10.57022 | 10.707678 | 10.358569 |
| MTCH1 | 0.000198449 | 0.015 | 459.59296 | 12.033343 | 0.030622002 | 12.000646 | 12.061348 | 12.038035 |
| MTCP1 | 0.00835548 | 0.14820297 | 1955.653 | 10.01187 | 0.20732486 | 9.774784 | 10.15916 | 10.1016655 |
| MTDH | 0.002927163 | 0.079838835 | 1270.5197 | 10.626622 | 0.19414058 | 10.446842 | 10.600539 | 10.832486 |
| MTERFD1 | 0.008234738 | 0.14726599 | 1943.9814 | 10.014823 | 0.12586214 | 10.122993 | 10.044795 | 9.87668 |
| MTHFS | 0.008241598 | 0.14731273 | 1944.6923 | 10.017593 | 0.10561849 | 9.897567 | 10.058886 | 10.096328 |
| MTMR4 | 0.009699227 | 0.1624843 | 2081.7458 | 9.916905 | 0.04302571 | 9.882684 | 9.965207 | 9.902825 |
| MTPN | 0.001516775 | 0.053972308 | 980.68195 | 10.999881 | 0.05256295 | 11.0598755 | 10.961927 | 10.977838 |
| MX1 | 0.007056436 | 0.1342193 | 1822.9795 | 10.110659 | 0.00262381 | 10.113436 | 10.11032 | 10.108221 |
| MXD4 | 0.006798639 | 0.13169849 | 1794.9744 | 10.1335745 | 0.054896366 | 10.094572 | 10.19635 | 10.109799 |
| MYB | 0.003104168 | 0.08316293 | 1300.5237 | 10.590766 | 0.05995484 | 10.527717 | 10.597529 | 10.647053 |
| MYH10 | 0.005641581 | 0.11776715 | 1660.7587 | 10.240521 | 0.07199662 | 10.313968 | 10.170068 | 10.237529 |
| MYH9 | 0.004702946 | 0.10588189 | 1540.6631 | 10.352387 | 0.103607014 | 10.429382 | 10.2345915 | 10.393188 |
| MYL12A | 3.53087E-05 | 0.004861111 | 253.24123 | 12.833969 | 0.20183827 | 12.604972 | 12.986004 | 12.910931 |
| MYL6 | 7.20586E-07 | 0.000297619 | 77.44333 | 13.822686 | 0.11657126 | 13.757041 | 13.957278 | 13.753741 |
| MYL6B | 0.006236756 | 0.12550928 | 1731.1857 | 10.181382 | 0.07766964 | 10.100561 | 10.188126 | 10.255461 |
| MYLIP | 0.006220355 | 0.12547036 | 1729.348 | 10.182384 | 0.078284405 | 10.092962 | 10.238551 | 10.215639 |
| MYO1B | 0.008546463 | 0.15043683 | 1974.3816 | 9.997303 | 0.102935396 | 10.020339 | 9.884801 | 10.086768 |
| MYO5C | 0.003281807 | 0.0857372 | 1329.9984 | 10.559066 | 0.04870339 | 10.615302 | 10.531311 | 10.530584 |
| NACA | 1.11258E-05 | 0.002284024 | 174.17133 | 13.254463 | 0.044270046 | 13.284548 | 13.275212 | 13.2036295 |
| NAG18 | 0.003254943 | 0.085356764 | 1325.5021 | 10.559211 | 0.115576334 | 10.638113 | 10.612972 | 10.426546 |
| NAGK | 0.008583732 | 0.15071052 | 1977.8494 | 9.996122 | 0.11067517 | 10.027454 | 10.087754 | 9.873159 |
| NAP1L4 | 0.009539171 | 0.16057836 | 2067.3625 | 9.925901 | 0.110036954 | 9.9137125 | 10.041525 | 9.822466 |
| NARS | 0.002247709 | 0.068166085 | 1143.9559 | 10.773303 | 0.15866512 | 10.942888 | 10.628467 | 10.748554 |
| NCBP1 | 0.001995936 | 0.06323927 | 1091.5571 | 10.848079 | 0.2947874 | 10.923522 | 10.5229 | 11.097811 |
| NCL | 0.004495475 | 0.103769794 | 1512.6254 | 10.379053 | 0.063510254 | 10.452221 | 10.346755 | 10.338182 |
| NCOA4 | 0.000384735 | 0.022817094 | 584.70667 | 11.695583 | 0.076222025 | 11.630182 | 11.779292 | 11.677277 |
| NCRNA00219 | 0.001771373 | 0.059035543 | 1041.7356 | 10.904644 | 0.08219039 | 10.813137 | 10.972195 | 10.9286 |
| NDUFA1 | 2.65752E-05 | 0.004043859 | 229.32994 | 12.946426 | 0.013110821 | 12.942502 | 12.96105 | 12.935724 |
| NDUFA11 | 0.003116735 | 0.08337086 | 1302.6388 | 10.588777 | 0.05541853 | 10.531311 | 10.593129 | 10.6418915 |
| NDUFA12 | 0.000532599 | 0.02829709 | 659.0177 | 11.535249 | 0.15125966 | 11.522185 | 11.692616 | 11.3909445 |
| NDUFA13 | 0.001617571 | 0.056063935 | 1005.1479 | 10.961258 | 0.108621 | 10.926917 | 11.082899 | 10.873959 |
| NDUFA2 | 0.000371073 | 0.022467714 | 577.25696 | 11.714351 | 0.035315618 | 11.727642 | 11.741093 | 11.674318 |
| NDUFA3 | 0.000320257 | 0.020349817 | 547.6547 | 11.792468 | 0.114383504 | 11.820387 | 11.890306 | 11.666709 |
| NDUFA4 | 2.4154E-05 | 0.003724444 | 223.09503 | 12.982892 | 0.064819805 | 12.973782 | 13.051786 | 12.923111 |
| NDUFA8 | 0.000288926 | 0.019351352 | 526.2533 | 11.841739 | 0.069643304 | 11.919557 | 11.785269 | 11.820387 |
| NDUFAB1 | 6.36421E-05 | 0.007145631 | 309.02365 | 12.588937 | 0.0682674 | 12.564199 | 12.666124 | 12.536487 |
| NDUFAF2 | 0.002952672 | 0.0803451 | 1274.9592 | 10.620221 | 0.10816188 | 10.53186 | 10.58796 | 10.740843 |
| NDUFB10 | 0.000201764 | 0.015151516 | 462.25146 | 12.027009 | 0.06050897 | 12.0763645 | 12.045162 | 11.959502 |
| NDUFB11 | 0.000777685 | 0.03522324 | 758.54956 | 11.350243 | 0.0713652 | 11.333673 | 11.428434 | 11.288619 |
| NDUFB2 | 6.96662E-05 | 0.007576802 | 319.2425 | 12.547437 | 0.039829414 | 12.591597 | 12.536487 | 12.514229 |
| NDUFB3 | 0.000155502 | 0.012814727 | 421.0474 | 12.141149 | 0.089915894 | 12.100503 | 12.0787325 | 12.244211 |
| NDUFB5 | 0.00020822 | 0.015502146 | 467.58896 | 12.008176 | 0.026141768 | 11.99708 | 12.038035 | 11.989412 |
| NDUFB6 | 0.005119041 | 0.1112782 | 1595.2777 | 10.299863 | 0.04169088 | 10.336901 | 10.307979 | 10.254712 |
| NDUFB7 | 0.000997954 | 0.04171446 | 832.83185 | 11.235732 | 0.03317291 | 11.225113 | 11.209168 | 11.272914 |
| NDUFB8 | 0.000440393 | 0.024763372 | 614.4839 | 11.621426 | 0.10840142 | 11.497277 | 11.669673 | 11.697328 |
| NDUFB9 | 0.000318153 | 0.020253211 | 546.23145 | 11.794827 | 0.12505798 | 11.730606 | 11.938948 | 11.714928 |
| NDUFS3 | 0.000138785 | 0.011947891 | 404.8409 | 12.199017 | 0.124703884 | 12.072812 | 12.202076 | 12.322164 |
| NDUFS4 | 0.001046463 | 0.042914893 | 847.89795 | 11.206116 | 0.096152365 | 11.095744 | 11.250868 | 11.271735 |
| NDUFS5 | 9.40797E-05 | 0.009194367 | 353.19797 | 12.412663 | 0.09483872 | 12.487966 | 12.306153 | 12.4438715 |
| NDUFS8 | 8.30403E-05 | 0.008498525 | 338.9481 | 12.454464 | 0.29694435 | 12.676879 | 12.569252 | 12.117259 |
| NDUFV1 | 0.005243039 | 0.1127026 | 1611.0172 | 10.287625 | 0.12494724 | 10.262338 | 10.423283 | 10.177257 |
| NDUFV2 | 0.001063757 | 0.043418825 | 853.4717 | 11.20259 | 0.038360294 | 11.176598 | 11.1845255 | 11.246648 |
| NEDD8 | 0.000419756 | 0.02387377 | 603.82 | 11.655296 | 0.06782341 | 11.72946 | 11.596423 | 11.640007 |
| NENF | 0.002766559 | 0.07790828 | 1241.6445 | 10.653548 | 0.012044459 | 10.64113 | 10.665181 | 10.65433 |
| NFE2L2 | 0.009410532 | 0.15895277 | 2055.874 | 9.936839 | 0.0526648 | 9.944939 | 9.984984 | 9.880593 |
| NFKB1 | 0.003243241 | 0.08517865 | 1323.5856 | 10.564426 | 0.019967262 | 10.555749 | 10.587265 | 10.550267 |
| NFKBIA | 0.000114458 | 0.010477573 | 377.21088 | 12.311702 | 0.12447173 | 12.2460165 | 12.233832 | 12.455257 |
| NGFRAP1 | 0.005324379 | 0.11360639 | 1621.4424 | 10.276566 | 0.023505561 | 10.30313 | 10.258462 | 10.268104 |
| NGRN | 0.00262887 | 0.07525247 | 1217.3535 | 10.690389 | 0.1011427 | 10.578506 | 10.77534 | 10.71732 |
| NHP2 | 0.000483023 | 0.026349057 | 635.11395 | 11.583388 | 0.0545995 | 11.598496 | 11.628842 | 11.522825 |
| NIF3L1 | 0.002079236 | 0.064813115 | 1109.2255 | 10.823136 | 0.049587075 | 10.789695 | 10.799606 | 10.880108 |
| NINJ1 | 0.001345103 | 0.05001822 | 935.3703 | 11.064636 | 0.22431223 | 11.32009 | 10.89985 | 10.9739685 |
| NIP7 | 0.004933678 | 0.10902484 | 1571.3077 | 10.322196 | 0.07219126 | 10.242086 | 10.342289 | 10.382212 |
| NIPSNAP1 | 0.007099988 | 0.13475218 | 1827.6903 | 10.110547 | 0.17514582 | 9.983491 | 10.310342 | 10.037808 |
| NIT2 | 0.009610076 | 0.16122437 | 2073.665 | 9.925126 | 0.108858675 | 10.039864 | 9.8232975 | 9.912216 |
| NMD3 | 0.000251686 | 0.017464 | 502.25143 | 11.917202 | 0.022905499 | 11.932888 | 11.890917 | 11.927802 |
| NME1 | 0.004870323 | 0.10817606 | 1563.1206 | 10.331193 | 0.040104274 | 10.36563 | 10.287162 | 10.340787 |
| NME1-NME2 | 0.001458552 | 0.052492738 | 965.5837 | 11.02103 | 0.029270144 | 10.993945 | 11.052081 | 11.017065 |
| NME3 | 0.008333573 | 0.14804147 | 1953.4949 | 10.012269 | 0.16009957 | 9.82748 | 10.099993 | 10.109333 |
| NME4 | 0.003210296 | 0.08476256 | 1318.0844 | 10.567483 | 0.10955636 | 10.537679 | 10.688857 | 10.475912 |
| NMRAL1 | 0.002983917 | 0.081004694 | 1280.5057 | 10.606074 | 0.08395145 | 10.623208 | 10.680137 | 10.514877 |
| NOL11 | 0.000459849 | 0.025404459 | 624.2521 | 11.607838 | 0.07225746 | 11.688283 | 11.586785 | 11.548444 |
| NOL7 | 0.000225659 | 0.016276507 | 481.7921 | 11.97435 | 0.0772221 | 11.885229 | 12.01638 | 12.02144 |
| NONO | 0.007626535 | 0.14007147 | 1883.1519 | 10.065469 | 0.06157022 | 10.0828085 | 10.11651 | 9.997088 |
| NOP10 | 0.000185853 | 0.014328889 | 449.64453 | 12.059601 | 0.08065828 | 12.102242 | 12.109989 | 11.966573 |
| NOP56 | 0.003065054 | 0.082497284 | 1294.1641 | 10.598849 | 0.04766612 | 10.571262 | 10.571395 | 10.653889 |
| NOP58 | 0.001128466 | 0.04500115 | 873.70197 | 11.169957 | 0.045173343 | 11.149131 | 11.2217865 | 11.138953 |
| NOSIP | 0.001431689 | 0.051902823 | 958.2174 | 11.033604 | 0.070921354 | 11.025725 | 11.108135 | 10.96695 |
| NPC2 | 0.000401943 | 0.023280468 | 593.9453 | 11.672195 | 0.11749205 | 11.566037 | 11.652116 | 11.798433 |
| NPEPL1 | 0.002346803 | 0.0700086 | 1163.2415 | 10.750653 | 0.1152761 | 10.883563 | 10.677888 | 10.690509 |
| NQO1 | 0.004242434 | 0.09992328 | 1476.7666 | 10.41013 | 0.06849921 | 10.485349 | 10.351337 | 10.3937025 |
| NR2C2AP | 0.005680434 | 0.118364565 | 1665.2305 | 10.237748 | 0.043889493 | 10.197465 | 10.284521 | 10.231257 |
| NR2F2 | 0.008881018 | 0.15352167 | 2006.8795 | 9.972221 | 0.08475481 | 9.9168625 | 10.069793 | 9.930008 |
| NRAS | 0.005601343 | 0.11728002 | 1656.0948 | 10.245959 | 0.03808245 | 10.22588 | 10.28988 | 10.222119 |
| NRD1 | 0.009170491 | 0.15657529 | 2034.0349 | 9.95083 | 0.04451077 | 9.935979 | 10.000868 | 9.915644 |
| NRIP1 | 0.005090765 | 0.11101131 | 1591.6846 | 10.304698 | 0.05393848 | 10.246505 | 10.353018 | 10.31457 |
| NSA2 | 0.000629561 | 0.031563584 | 701.37006 | 11.46157 | 0.2288578 | 11.586785 | 11.600497 | 11.197426 |
| NSDHL | 0.009658298 | 0.16187681 | 2078.1584 | 9.917453 | 0.02097663 | 9.917275 | 9.938518 | 9.896565 |
| NSMCE4A | 0.003488817 | 0.08854499 | 1363.6504 | 10.522412 | 0.04202732 | 10.486971 | 10.568843 | 10.511426 |
| NSUN2 | 0.002334035 | 0.06980776 | 1160.69 | 10.755803 | 0.040628042 | 10.74599 | 10.80044 | 10.720982 |
| NT5C | 0.002409062 | 0.0711925 | 1175.4872 | 10.741226 | 0.051324748 | 10.790539 | 10.745037 | 10.688102 |
| NT5C2 | 0.001691705 | 0.057597645 | 1022.85626 | 10.935127 | 0.030634418 | 10.967868 | 10.930351 | 10.90716 |
| NT5C3 | 0.00646619 | 0.12797377 | 1757.5876 | 10.160759 | 0.06538746 | 10.089634 | 10.218263 | 10.174382 |
| NUCB1 | 0.000683288 | 0.032743093 | 722.70154 | 11.407883 | 0.07271848 | 11.472204 | 11.328978 | 11.422466 |
| NUCKS1 | 0.000179311 | 0.013979775 | 442.83337 | 12.085079 | 0.11924693 | 11.9488125 | 12.136085 | 12.170341 |
| NUDC | 0.000410071 | 0.023554636 | 598.4096 | 11.665512 | 0.15105075 | 11.819038 | 11.660432 | 11.517065 |
| NUDT14 | 0.007442987 | 0.13794178 | 1864.2782 | 10.080009 | 0.03582274 | 10.066821 | 10.120557 | 10.05265 |
| NUDT5 | 0.00194097 | 0.06229417 | 1079.8994 | 10.863121 | 0.046738476 | 10.862919 | 10.90996 | 10.8164835 |
| NUDT7 | 0.006222056 | 0.12543173 | 1729.539 | 10.182647 | 0.030953491 | 10.172339 | 10.158163 | 10.21744 |
| NUP107 | 0.003615611 | 0.09070137 | 1383.9159 | 10.501666 | 0.13741612 | 10.635107 | 10.360593 | 10.509297 |
| NUP205 | 0.006222517 | 0.12536818 | 1729.6069 | 10.1842985 | 0.17396918 | 10.224651 | 9.993699 | 10.334545 |
| NUP37 | 0.002151669 | 0.06594523 | 1124.0544 | 10.803886 | 0.08474276 | 10.851541 | 10.8540745 | 10.706045 |
| NUP62 | 0.006202196 | 0.12524971 | 1727.2485 | 10.189987 | 0.084366694 | 10.259411 | 10.214462 | 10.096089 |
| NUP85 | 0.008766848 | 0.15261264 | 1995.5016 | 9.982506 | 0.1897611 | 10.020536 | 10.150372 | 9.776609 |
| NUP88 | 0.002459618 | 0.07189048 | 1185.221 | 10.726379 | 0.06722077 | 10.793897 | 10.659459 | 10.725782 |
| NUSAP1 | 0.007025912 | 0.13393241 | 1819.7915 | 10.114205 | 0.03132786 | 10.088394 | 10.14906 | 10.105162 |
| NXT1 | 0.002356517 | 0.07017768 | 1165.2743 | 10.7506 | 0.045859504 | 10.711921 | 10.801261 | 10.738618 |
| OAZ1 | 2.53646E-06 | 0.000838095 | 105.653076 | 13.656471 | 0.075834304 | 13.733847 | 13.582278 | 13.653289 |
| OCIAD1 | 0.00159163 | 0.05538616 | 998.9185 | 10.974294 | 0.08467407 | 10.996298 | 11.045794 | 10.88079 |
| OCIAD2 | 0.002847092 | 0.07914824 | 1256.0242 | 10.6392765 | 0.059514083 | 10.620986 | 10.591055 | 10.70579 |
| ODC1 | 0.009029918 | 0.15501435 | 2020.7928 | 9.962769 | 0.025586182 | 9.950101 | 9.992218 | 9.945989 |
| ORC6L | 0.000738456 | 0.03448183 | 743.7526 | 11.3772545 | 0.10037853 | 11.261369 | 11.43714 | 11.433253 |
| OSTC | 0.00132608 | 0.049683586 | 930.21924 | 11.07915 | 0.025142064 | 11.068665 | 11.107839 | 11.060949 |
| OSTF1 | 0.004583847 | 0.10511038 | 1524.2473 | 10.36349 | 0.022637432 | 10.339255 | 10.38409 | 10.367125 |
| OXR1 | 0.009881882 | 0.16396078 | 2098.4087 | 9.904696 | 0.1521619 | 9.896106 | 9.757011 | 10.060971 |
| P15RS | 0.005766242 | 0.11936396 | 1675.8555 | 10.232152 | 0.14537479 | 10.06458 | 10.324522 | 10.307352 |
| P4HB | 0.000285035 | 0.019201942 | 523.78345 | 11.846412 | 0.07942466 | 11.911171 | 11.7577915 | 11.870271 |
| PA2G4 | 0.006495503 | 0.12818828 | 1760.9067 | 10.161891 | 0.22182883 | 9.972466 | 10.405924 | 10.10728 |
| PABPC1 | 3.41846E-05 | 0.004801619 | 250.29816 | 12.839473 | 0.114478596 | 12.738503 | 12.8160715 | 12.963844 |
| PABPC4 | 0.004988413 | 0.10988444 | 1578.4694 | 10.31594 | 0.15073133 | 10.16653 | 10.467958 | 10.313332 |
| PABPN1 | 0.009056955 | 0.15524803 | 2023.3475 | 9.95899 | 0.08289744 | 9.92365 | 9.89962 | 10.053701 |
| PAFAH1B3 | 0.002664005 | 0.07588259 | 1223.4344 | 10.680261 | 0.070462406 | 10.599098 | 10.725782 | 10.715902 |
| PAM | 0.004933533 | 0.10909114 | 1571.2899 | 10.320261 | 0.059368875 | 10.337484 | 10.369114 | 10.254185 |
| PAPOLA | 0.002803078 | 0.07842742 | 1248.1837 | 10.649363 | 0.0526598 | 10.593772 | 10.655821 | 10.698496 |
| PAPSS2 | 0.00646129 | 0.12802285 | 1757.1477 | 10.162526 | 0.040400427 | 10.19635 | 10.117791 | 10.17344 |
| PAQR4 | 0.006072462 | 0.12378261 | 1711.8374 | 10.201279 | 0.07891902 | 10.287374 | 10.184094 | 10.132367 |
| PARK7 | 0.000121779 | 0.010974026 | 386.97324 | 12.265129 | 0.008078028 | 12.258447 | 12.262834 | 12.274106 |
| PARL | 0.004009137 | 0.09652533 | 1443.3994 | 10.443746 | 0.02553128 | 10.420727 | 10.439305 | 10.471207 |
| PARP1 | 0.00072269 | 0.034020353 | 737.6556 | 11.385186 | 0.17345974 | 11.432551 | 11.192965 | 11.530045 |
| PARP4 | 0.005658241 | 0.1180439 | 1662.7637 | 10.2402 | 0.050086543 | 10.243311 | 10.18863 | 10.288658 |
| PAWR | 0.005731884 | 0.118936606 | 1671.6566 | 10.231472 | 0.16219167 | 10.245905 | 10.062546 | 10.385964 |
| PBX3 | 0.007511356 | 0.13883804 | 1871.3337 | 10.073285 | 0.124865346 | 9.933024 | 10.172339 | 10.1144905 |
| PCBP1 | 0.000148729 | 0.012554744 | 414.84708 | 12.175385 | 0.05061263 | 12.229154 | 12.128668 | 12.168334 |
| PCCB | 0.000671096 | 0.0323375 | 717.7483 | 11.4221115 | 0.061080277 | 11.422466 | 11.483013 | 11.360854 |
| PCID2 | 0.004794575 | 0.10711075 | 1553.099 | 10.339773 | 0.0732914 | 10.28988 | 10.30552 | 10.42392 |
| PCMT1 | 0.000865654 | 0.0377299 | 789.884 | 11.299806 | 0.027057005 | 11.269406 | 11.321249 | 11.308763 |
| PCNP | 0.002133885 | 0.06557396 | 1120.5383 | 10.806343 | 0.06818487 | 10.7296915 | 10.860244 | 10.829094 |
| PCP4 | 0.004417825 | 0.1023861 | 1501.3824 | 10.384854 | 0.16906373 | 10.512156 | 10.449376 | 10.19303 |
| PDCD5 | 0.002567043 | 0.07403242 | 1205.4489 | 10.697923 | 0.036886882 | 10.660035 | 10.700016 | 10.73372 |
| PDCD6 | 0.000494725 | 0.02690282 | 640.81696 | 11.5710945 | 0.058304254 | 11.57973 | 11.508955 | 11.6246 |
| PDCD7 | 0.000335793 | 0.02099099 | 557.0816 | 11.760223 | 0.04019598 | 11.71741 | 11.797153 | 11.766108 |
| PDCL3 | 0.007749179 | 0.14112861 | 1895.6841 | 10.052728 | 0.06837341 | 10.124635 | 10.045007 | 9.9885435 |
| PDHA1 | 0.001769441 | 0.059141617 | 1041.3053 | 10.910671 | 0.043390628 | 10.904514 | 10.956812 | 10.870688 |
| PDHB | 0.00174327 | 0.05871942 | 1035.2579 | 10.924962 | 0.17941518 | 11.130612 | 10.843834 | 10.80044 |
| PDXDC1 | 0.002149478 | 0.06593634 | 1123.6016 | 10.805088 | 0.014757119 | 10.806457 | 10.819114 | 10.789695 |
| PDXK | 0.002916009 | 0.07978549 | 1268.5594 | 10.6285925 | 0.07532908 | 10.602901 | 10.5694685 | 10.713406 |
| PEBP1 | 0.000363694 | 0.022136843 | 573.1157 | 11.727851 | 0.101824805 | 11.689598 | 11.650691 | 11.843262 |
| PELO | 0.008712141 | 0.15234828 | 1990.1943 | 9.9828415 | 0.111292504 | 10.104383 | 9.885922 | 9.958219 |
| PERP | 0.003025365 | 0.08193755 | 1287.5493 | 10.611501 | 0.13917936 | 10.575013 | 10.4942 | 10.765289 |
| PFDN5 | 7.4595E-05 | 0.007866262 | 326.68985 | 12.515231 | 0.09386809 | 12.622409 | 12.475639 | 12.447645 |
| PFKP | 0.002012365 | 0.06341235 | 1095.0782 | 10.839522 | 0.054037955 | 10.898801 | 10.826755 | 10.793012 |
| PFN1 | 1.38352E-05 | 0.002580645 | 186.08214 | 13.158984 | 0.0798579 | 13.10136 | 13.125451 | 13.250141 |
| PGD | 0.003950453 | 0.09597829 | 1434.9276 | 10.453759 | 0.08991059 | 10.394237 | 10.557186 | 10.409853 |
| PGK1 | 0.001976826 | 0.062863424 | 1087.4498 | 10.851069 | 0.052204408 | 10.887307 | 10.791231 | 10.874666 |
| PGRMC1 | 0.000277973 | 0.018835938 | 518.92535 | 11.86362 | 0.112878606 | 11.966232 | 11.74271 | 11.881914 |
| PGRMC2 | 0.004652706 | 0.10598884 | 1533.8574 | 10.359092 | 0.059841845 | 10.428187 | 10.325171 | 10.323916 |
| PHB | 0.003421456 | 0.08779882 | 1352.8112 | 10.531886 | 0.0804218 | 10.580104 | 10.576508 | 10.439046 |
| PHF13 | 0.00918202 | 0.156618 | 2035.0813 | 9.953822 | 0.12291729 | 9.829944 | 10.075755 | 9.955769 |
| PHGDH | 0.003468871 | 0.088297136 | 1360.5546 | 10.525899 | 0.07547026 | 10.588676 | 10.546856 | 10.442165 |
| PHPT1 | 0.001051738 | 0.043080285 | 849.55524 | 11.206929 | 0.054288186 | 11.180137 | 11.1712475 | 11.269406 |
| PIGY | 0.001916931 | 0.061866045 | 1074.4979 | 10.866076 | 0.049006645 | 10.836074 | 10.839526 | 10.922629 |
| PIK3R2 | 0.000764484 | 0.03503699 | 753.58636 | 11.359235 | 0.0494214 | 11.32674 | 11.416109 | 11.334855 |
| PIN1 | 0.00271975 | 0.07696493 | 1233.5511 | 10.66768 | 0.07597488 | 10.680988 | 10.585931 | 10.736122 |
| PIP4K2A | 0.002638468 | 0.0754028 | 1219.0437 | 10.679466 | 0.1147868 | 10.804738 | 10.65433 | 10.57933 |
| PIP5K2B | 0.006400185 | 0.12739415 | 1750.091 | 10.167385 | 0.04242573 | 10.199858 | 10.119383 | 10.182916 |
| PIPSL | 0.006063931 | 0.12375412 | 1710.8485 | 10.201629 | 0.051597007 | 10.260605 | 10.164819 | 10.179462 |
| PJA2 | 0.002081253 | 0.06481777 | 1109.6593 | 10.822116 | 0.008237573 | 10.8164835 | 10.818293 | 10.83157 |
| PLOD1 | 0.006897417 | 0.13279633 | 1805.7704 | 10.124107 | 0.09572838 | 10.21514 | 10.024289 | 10.132892 |
| PLOD3 | 0.004028939 | 0.09680055 | 1446.2777 | 10.43892 | 0.04967719 | 10.46924 | 10.38159 | 10.465931 |
| PLSCR3 | 0.002737361 | 0.07733713 | 1236.576 | 10.658293 | 0.050678458 | 10.663711 | 10.706045 | 10.6051235 |
| PMPCB | 0.003332997 | 0.08642377 | 1338.7189 | 10.547129 | 0.086864665 | 10.586637 | 10.447533 | 10.607218 |
| PMS2L4 | 0.009914106 | 0.16433828 | 2101.241 | 9.903516 | 0.120452374 | 9.859131 | 10.039864 | 9.811555 |
| PNN | 0.009549404 | 0.16051696 | 2068.2573 | 9.928932 | 0.13384145 | 9.845806 | 10.083327 | 9.857663 |
| PNPO | 0.000259555 | 0.017867064 | 507.48706 | 11.902229 | 0.041033342 | 11.942084 | 11.860111 | 11.904491 |
| PNPT1 | 0.008270911 | 0.14753264 | 1947.4845 | 10.013978 | 0.12027397 | 10.088933 | 9.875248 | 10.077754 |
| POGK | 0.007881392 | 0.14286155 | 1909.1228 | 10.042907 | 0.13204667 | 10.1531515 | 9.896565 | 10.079003 |
| POLA2 | 0.007298813 | 0.13687837 | 1848.8972 | 10.091854 | 0.10602622 | 9.98852 | 10.200381 | 10.086661 |
| POLE3 | 0.000135816 | 0.011809524 | 401.77295 | 12.203176 | 0.059265863 | 12.146549 | 12.198212 | 12.264769 |
| POLE4 | 0.000239004 | 0.016887983 | 492.2649 | 11.94843 | 0.024684766 | 11.969742 | 11.921382 | 11.9541645 |
| POLR1C | 0.009996973 | 0.16508092 | 2108.773 | 9.900622 | 0.04392198 | 9.857753 | 9.898589 | 9.945526 |
| POLR1D | 0.005820632 | 0.12020297 | 1682.1271 | 10.227942 | 0.12656607 | 10.237425 | 10.349501 | 10.096902 |
| POLR2A | 0.003982504 | 0.0964194 | 1439.4614 | 10.445449 | 0.029667119 | 10.477795 | 10.439046 | 10.419506 |
| POLR2F | 0.001448781 | 0.05230385 | 962.6129 | 11.027573 | 0.07454883 | 11.062707 | 11.078064 | 10.94195 |
| POLR2G | 0.000200899 | 0.015119306 | 461.71884 | 12.031666 | 0.083287604 | 12.012693 | 11.959502 | 12.122804 |
| POLR2H | 0.000218165 | 0.015968354 | 476.05914 | 11.992511 | 0.04943718 | 12.048724 | 11.955796 | 11.973013 |
| POLR2I | 0.002161296 | 0.06600704 | 1125.9258 | 10.798714 | 0.09897456 | 10.685011 | 10.865556 | 10.845572 |
| POLR3C | 0.005134807 | 0.111411504 | 1597.2914 | 10.2985325 | 0.013649618 | 10.309708 | 10.30257 | 10.283319 |
| POLR3GL | 0.003317404 | 0.08634209 | 1335.979 | 10.550429 | 0.042580757 | 10.548863 | 10.593772 | 10.508654 |
| POM121C | 0.003182827 | 0.08442278 | 1313.7435 | 10.577346 | 0.05011401 | 10.5338 | 10.566113 | 10.632122 |
| PPA1 | 9.47714E-05 | 0.009158774 | 354.0713 | 12.400107 | 0.14574727 | 12.260701 | 12.55146 | 12.38816 |
| PPCS | 0.005032455 | 0.110224746 | 1584.3114 | 10.311664 | 0.06994054 | 10.241931 | 10.31125 | 10.38181 |
| PPIAL4A | 4.03528E-07 | 0.000186667 | 69.32217 | 13.883682 | 0.13015604 | 14.008287 | 13.894151 | 13.748608 |
| PPIC | 0.00171067 | 0.058072407 | 1027.5146 | 10.925807 | 0.03048111 | 10.905045 | 10.9608 | 10.911573 |
| PPM1D | 0.000391826 | 0.023001691 | 588.65955 | 11.687859 | 0.17938481 | 11.543165 | 11.888567 | 11.631843 |
| PPP1CC | 0.000444428 | 0.024829308 | 616.5443 | 11.620702 | 0.11415128 | 11.75159 | 11.541778 | 11.568737 |
| PPP1R11 | 0.002865712 | 0.07941134 | 1259.5188 | 10.636776 | 0.100320004 | 10.607218 | 10.748554 | 10.554556 |
| PPP1R14B | 0.001571857 | 0.055140547 | 994.2872 | 10.979379 | 0.08485115 | 11.045012 | 11.009562 | 10.883563 |
| PPP2CA | 0.000907563 | 0.039114285 | 804.2939 | 11.273414 | 0.13289362 | 11.2963295 | 11.130552 | 11.393359 |
| PPP2R1A | 0.00215458 | 0.06597617 | 1124.7373 | 10.802737 | 0.06916912 | 10.8808565 | 10.749276 | 10.778079 |
| PPP2R5E | 0.001129215 | 0.044927754 | 873.98065 | 11.1682005 | 0.0937212 | 11.1295185 | 11.100012 | 11.275071 |
| PPP4C | 0.000761227 | 0.034980133 | 752.2909 | 11.361066 | 0.10360384 | 11.35246 | 11.262034 | 11.468705 |
| PPP6C | 0.000824437 | 0.036670513 | 775.699 | 11.322528 | 0.16490792 | 11.143034 | 11.467331 | 11.357216 |
| PPT1 | 0.000737217 | 0.034516867 | 743.35614 | 11.377934 | 0.09063922 | 11.375345 | 11.288619 | 11.469842 |
| PQLC3 | 0.005167061 | 0.11169221 | 1601.5015 | 10.29657 | 0.11672158 | 10.172858 | 10.404745 | 10.312107 |
| PRC1 | 0.000826627 | 0.036720872 | 776.50903 | 11.320015 | 0.030390032 | 11.3547945 | 11.298586 | 11.306663 |
| PRDM4 | 0.006562979 | 0.12871453 | 1768.4807 | 10.154452 | 0.059196874 | 10.209535 | 10.091858 | 10.161963 |
| PRDX1 | 1.53917E-05 | 0.002752577 | 192.79482 | 13.134095 | 0.084658474 | 13.061555 | 13.113616 | 13.227115 |
| PRDX3 | 0.001830489 | 0.06036787 | 1055.6401 | 10.8898 | 0.097721696 | 10.777084 | 10.950741 | 10.941573 |
| PRDX4 | 0.000290021 | 0.019387282 | 527.1208 | 11.850517 | 0.120737806 | 11.89755 | 11.713341 | 11.94066 |
| PRDX5 | 0.000552805 | 0.028927602 | 668.2974 | 11.512029 | 0.053698912 | 11.510261 | 11.566589 | 11.459235 |
| PRDX6 | 0.000656627 | 0.03213117 | 712.0436 | 11.43431 | 0.09572259 | 11.428434 | 11.532835 | 11.3416605 |
| PRELID1 | 0.004692714 | 0.105926484 | 1539.289 | 10.348233 | 0.14203463 | 10.338666 | 10.494809 | 10.211224 |
| PRIC285 | 0.001169712 | 0.045855366 | 886.1208 | 11.145701 | 0.036040604 | 11.174214 | 11.105192 | 11.157696 |
| PRICKLE4 | 0.000276849 | 0.018796477 | 518.22986 | 11.869428 | 0.04042349 | 11.823894 | 11.901083 | 11.883309 |
| PRIM1 | 0.008898023 | 0.15358607 | 2008.4956 | 9.971603 | 0.08070498 | 9.8845 | 9.986466 | 10.043843 |
| PRKAR1A | 0.009545023 | 0.16059893 | 2067.8677 | 9.925872 | 0.023473306 | 9.907444 | 9.917871 | 9.952299 |
| PRKRA | 0.008609673 | 0.15108953 | 1980.2526 | 9.992183 | 0.10633592 | 10.014444 | 9.876479 | 10.085627 |
| PRKRIR | 0.001334265 | 0.049668454 | 932.47504 | 11.074417 | 0.115855195 | 10.983571 | 11.034796 | 11.2048855 |
| PRMT6 | 0.000392229 | 0.022947723 | 588.9024 | 11.686624 | 0.016734095 | 11.685396 | 11.670538 | 11.7039385 |
| PRPF3 | 0.008876924 | 0.15352742 | 2006.4435 | 9.972028 | 0.045249745 | 9.919797 | 9.999375 | 9.99691 |
| PRPF4 | 0.005222085 | 0.1124612 | 1608.358 | 10.286359 | 0.05744365 | 10.306728 | 10.330842 | 10.221506 |
| PRPF8 | 0.002373292 | 0.07043541 | 1168.4003 | 10.750678 | 0.05596966 | 10.793012 | 10.771802 | 10.687221 |
| PRPSAP1 | 0.00237162 | 0.07044606 | 1168.117 | 10.748158 | 0.034924697 | 10.788101 | 10.723375 | 10.732998 |
| PRR13 | 0.00505831 | 0.110511966 | 1587.5555 | 10.308267 | 0.04643202 | 10.318142 | 10.257691 | 10.348967 |
| PRR14 | 0.001345997 | 0.049944386 | 935.6941 | 11.064972 | 0.12578544 | 11.2048855 | 10.961248 | 11.028784 |
| PRR15L | 0.006451144 | 0.12789486 | 1755.9755 | 10.158623 | 0.041063823 | 10.151493 | 10.121591 | 10.202785 |
| PRRT3 | 0.006367239 | 0.12688397 | 1746.3074 | 10.170741 | 0.01536363 | 10.168802 | 10.186981 | 10.156438 |
| PRSS23 | 0.004041016 | 0.09688943 | 1447.9772 | 10.441084 | 0.098872185 | 10.451338 | 10.337484 | 10.53443 |
| PRSS8 | 0.001683115 | 0.05747441 | 1020.6374 | 10.944049 | 0.13424864 | 10.956812 | 11.071461 | 10.803875 |
| PSAP | 0.000111662 | 0.010275862 | 373.85132 | 12.316442 | 0.04956903 | 12.347284 | 12.259263 | 12.342776 |
| PSMA3 | 0.001428777 | 0.05190576 | 957.4685 | 11.033954 | 0.0658596 | 11.094416 | 10.963776 | 11.043668 |
| PSMA4 | 0.000123768 | 0.01103856 | 389.196 | 12.25072 | 0.04755329 | 12.286743 | 12.196818 | 12.268597 |
| PSMA5 | 7.22892E-05 | 0.007693252 | 323.24136 | 12.534565 | 0.08009784 | 12.521128 | 12.620532 | 12.462036 |
| PSMA6 | 0.000117974 | 0.010742783 | 382.1834 | 12.289975 | 0.08975556 | 12.2338085 | 12.242628 | 12.393492 |
| PSMB1 | 7.63821E-06 | 0.001698718 | 155.51775 | 13.350583 | 0.09020896 | 13.351594 | 13.440283 | 13.259873 |
| PSMB10 | 0.00269966 | 0.07677213 | 1229.9098 | 10.669575 | 0.026372556 | 10.653651 | 10.655056 | 10.700016 |
| PSMB2 | 0.002196288 | 0.066723295 | 1133.2136 | 10.79234 | 0.07805375 | 10.7085085 | 10.862919 | 10.805594 |
| PSMB3 | 0.000428604 | 0.02425775 | 608.0549 | 11.64251 | 0.09993533 | 11.695807 | 11.527225 | 11.7045 |
| PSMB4 | 0.000145529 | 0.012405406 | 411.77383 | 12.176643 | 0.058704827 | 12.198212 | 12.110206 | 12.221514 |
| PSMB5 | 0.00030452 | 0.019896422 | 537.4414 | 11.815175 | 0.06977865 | 11.814623 | 11.885229 | 11.745675 |
| PSMB6 | 2.36064E-05 | 0.00365625 | 221.5764 | 12.981869 | 0.11502222 | 12.920095 | 12.910931 | 13.114579 |
| PSMB7 | 0.000108953 | 0.01016129 | 371.05753 | 12.333915 | 0.105783165 | 12.394988 | 12.394988 | 12.211766 |
| PSMC1 | 8.81709E-05 | 0.008765043 | 345.39044 | 12.435472 | 0.10860883 | 12.43166 | 12.545936 | 12.328818 |
| PSMC2 | 0.000228339 | 0.016334021 | 484.05847 | 11.961613 | 0.056582835 | 11.94385 | 12.024946 | 11.916043 |
| PSMC3 | 0.00056468 | 0.029371815 | 673.2957 | 11.5032 | 0.106055856 | 11.501429 | 11.610129 | 11.39804 |
| PSMC5 | 2.10988E-05 | 0.00343662 | 213.29884 | 13.021423 | 0.10323477 | 13.132812 | 13.002498 | 12.928961 |
| PSMD14 | 0.00094538 | 0.040096577 | 816.3658 | 11.256974 | 0.11149769 | 11.262034 | 11.143034 | 11.365857 |
| PSMD2 | 0.003465787 | 0.08834828 | 1360.0972 | 10.527304 | 0.078501 | 10.601998 | 10.53443 | 10.445482 |
| PSMD4 | 0.006645645 | 0.12960315 | 1777.9961 | 10.144468 | 0.010206789 | 10.134912 | 10.15522 | 10.143274 |
| PSMD6 | 4.92881E-06 | 0.001266667 | 133.16365 | 13.494857 | 0.050143413 | 13.551417 | 13.455852 | 13.477301 |
| PSMD7 | 0.001214936 | 0.04657569 | 898.9303 | 11.120627 | 0.13994732 | 11.255315 | 10.975955 | 11.130612 |
| PSMD8 | 0.006168386 | 0.12493053 | 1723.4163 | 10.193116 | 0.07334544 | 10.271915 | 10.180598 | 10.126836 |
| PSME2 | 0.000230443 | 0.016416838 | 485.64368 | 11.969842 | 0.013745304 | 11.985687 | 11.962728 | 11.961114 |
| PSME4 | 0.0030343 | 0.08205144 | 1288.9014 | 10.604537 | 0.052322414 | 10.634382 | 10.544122 | 10.635107 |
| PSMG1 | 0.002313657 | 0.06949784 | 1156.6381 | 10.76313 | 0.10655757 | 10.721313 | 10.8842535 | 10.683824 |
| PSMG2 | 0.001980054 | 0.062908426 | 1088.126 | 10.849528 | 0.051813435 | 10.89985 | 10.796342 | 10.852393 |
| PTBP1 | 0.000100594 | 0.009640884 | 360.9917 | 12.374894 | 0.006108703 | 12.3757 | 12.38056 | 12.3684225 |
| PTCD1 | 0.006759353 | 0.13115716 | 1790.6234 | 10.134712 | 0.090103276 | 10.03199 | 10.171765 | 10.200381 |
| PTGES2 | 0.009934139 | 0.16435622 | 2102.942 | 9.90269 | 0.075984344 | 9.878191 | 9.987901 | 9.841976 |
| PTGES3 | 0.00091794 | 0.039365884 | 807.5463 | 11.276138 | 0.12057308 | 11.412458 | 11.183472 | 11.232487 |
| PTMA | 0.00070332 | 0.033426028 | 730.4292 | 11.4005785 | 0.10878449 | 11.277279 | 11.4414425 | 11.483013 |
| PTOV1 | 0.00256148 | 0.07405667 | 1204.3241 | 10.698379 | 0.0879982 | 10.746865 | 10.751469 | 10.596802 |
| PTPLAD1 | 0.005372514 | 0.11421201 | 1627.6292 | 10.273829 | 0.05998198 | 10.222399 | 10.259372 | 10.33972 |
| PTPLB | 0.000833343 | 0.036783714 | 778.72455 | 11.317773 | 0.109988876 | 11.332592 | 11.201127 | 11.419602 |
| PTPMT1 | 0.001065054 | 0.04342068 | 853.9778 | 11.202763 | 0.010623589 | 11.199503 | 11.194152 | 11.214635 |
| PTPN1 | 0.001332565 | 0.049765337 | 931.96814 | 11.078311 | 0.05340921 | 11.018843 | 11.093898 | 11.122193 |
| PTPN11 | 0.004600767 | 0.10522017 | 1526.7216 | 10.365284 | 0.094502136 | 10.432075 | 10.257156 | 10.406621 |
| PTPRF | 0.003132242 | 0.08352806 | 1305.1271 | 10.589181 | 0.043930415 | 10.574958 | 10.554125 | 10.638461 |
| PTPRK | 0.008226062 | 0.14726265 | 1943.1115 | 10.015721 | 0.1489218 | 9.875924 | 9.998901 | 10.172339 |
| PTTG1 | 0.001070329 | 0.043584507 | 855.5715 | 11.1935 | 0.049260817 | 11.165517 | 11.250379 | 11.164603 |
| PTTG1IP | 0.000747247 | 0.03465909 | 746.9512 | 11.370103 | 0.045868225 | 11.320046 | 11.410119 | 11.380145 |
| PTTG3P | 0.002619387 | 0.07510496 | 1215.5331 | 10.688439 | 0.1357726 | 10.743418 | 10.788101 | 10.5338 |
| PUF60 | 0.000356834 | 0.021795774 | 569.2182 | 11.728641 | 0.03590677 | 11.762584 | 11.732287 | 11.691049 |
| PUM1 | 0.00763939 | 0.14001109 | 1884.4966 | 10.064433 | 0.10203219 | 10.108118 | 10.137349 | 9.947831 |
| PURB | 0.006736814 | 0.13079295 | 1788.1376 | 10.14121 | 0.10148747 | 10.040972 | 10.243902 | 10.138757 |
| PUS7 | 0.00801493 | 0.14475273 | 1922.4484 | 10.036859 | 0.025523607 | 10.0300045 | 10.015463 | 10.06511 |
| PWP1 | 0.007645443 | 0.13997415 | 1885.1559 | 10.062124 | 0.09367965 | 10.159731 | 10.053701 | 9.97294 |
| QARS | 0.000527671 | 0.028164616 | 656.37897 | 11.541766 | 0.09328907 | 11.649217 | 11.481431 | 11.494652 |
| QRFPR | 0.006487231 | 0.12824388 | 1759.9473 | 10.159504 | 0.020072283 | 10.13988 | 10.1799965 | 10.158634 |
| RAB10 | 0.000792154 | 0.035692208 | 763.9734 | 11.341279 | 0.13443513 | 11.429563 | 11.186561 | 11.407716 |
| RAB11A | 0.000853519 | 0.03734174 | 785.6363 | 11.3096895 | 0.04685548 | 11.276272 | 11.289552 | 11.363249 |
| RAB11FIP3 | 0.007411368 | 0.13779743 | 1861.0168 | 10.082817 | 0.03193208 | 10.083327 | 10.1144905 | 10.050632 |
| RAB13 | 0.007480227 | 0.13841014 | 1868.0537 | 10.078962 | 0.030637436 | 10.043899 | 10.092428 | 10.100561 |
| RAB17 | 0.008940941 | 0.15409687 | 2012.5209 | 9.969019 | 0.08222068 | 10.058886 | 9.897567 | 9.950605 |
| RAB21 | 0.004377818 | 0.10186721 | 1495.9052 | 10.390887 | 0.034016266 | 10.369114 | 10.373464 | 10.430086 |
| RAB22A | 0.006568398 | 0.12867533 | 1769.1265 | 10.156116 | 0.07763574 | 10.238551 | 10.145401 | 10.084393 |
| RAB25 | 0.004600565 | 0.10528496 | 1526.6882 | 10.361066 | 0.075390585 | 10.292234 | 10.441638 | 10.349325 |
| RAB2B | 0.005578227 | 0.11714952 | 1653.2197 | 10.250867 | 0.10244213 | 10.329581 | 10.287978 | 10.135041 |
| RAB31 | 0.003448349 | 0.08803311 | 1357.341 | 10.527248 | 0.087399624 | 10.590886 | 10.427596 | 10.563263 |
| RAB3IP | 0.009193434 | 0.15658174 | 2036.0059 | 9.950032 | 0.06753173 | 9.878515 | 9.958874 | 10.012708 |
| RAB40C | 0.003063959 | 0.08253183 | 1293.9961 | 10.594892 | 0.031678002 | 10.619695 | 10.559207 | 10.605773 |
| RAB5A | 0.00983758 | 0.16353858 | 2094.3423 | 9.908395 | 0.16213124 | 9.924245 | 10.062018 | 9.73892 |
| RAB5B | 0.006032916 | 0.1232662 | 1707.3081 | 10.200769 | 0.010028996 | 10.191228 | 10.211224 | 10.199858 |
| RAB7A | 0.001297746 | 0.048885994 | 921.85315 | 11.090111 | 0.08963542 | 11.156823 | 11.125287 | 10.988223 |
| RAB8A | 0.002522626 | 0.07336128 | 1196.8984 | 10.709655 | 0.027236901 | 10.7094755 | 10.6825075 | 10.73698 |
| RAB9A | 0.008388021 | 0.14855233 | 1958.8428 | 10.0062685 | 0.07332738 | 9.923204 | 10.033584 | 10.062018 |
| RABEPK | 0.002655848 | 0.07577467 | 1221.8904 | 10.67931 | 0.08308162 | 10.67127 | 10.766118 | 10.600539 |
| RABGAP1 | 0.007319334 | 0.13704102 | 1851.0836 | 10.089589 | 0.025646487 | 10.108795 | 10.099508 | 10.060465 |
| RAD21 | 0.006115323 | 0.1241457 | 1716.9692 | 10.192541 | 0.043455493 | 10.160203 | 10.175483 | 10.241938 |
| RAD51C | 0.004262812 | 0.10013135 | 1479.6713 | 10.409036 | 0.09044028 | 10.336769 | 10.510458 | 10.379882 |
| RALA | 0.005745749 | 0.11908184 | 1673.3132 | 10.232791 | 0.15999572 | 10.054245 | 10.36317 | 10.280957 |
| RALB | 0.007087797 | 0.13466813 | 1826.4149 | 10.110686 | 0.13762978 | 10.059425 | 10.266589 | 10.006042 |
| RAN | 0.001258056 | 0.04770164 | 911.19934 | 11.110879 | 0.05295397 | 11.050953 | 11.151364 | 11.130322 |
| RANBP1 | 0.001077737 | 0.043732163 | 858.0828 | 11.192845 | 0.076259196 | 11.106026 | 11.248992 | 11.223518 |
| RANGAP1 | 0.003488644 | 0.08860542 | 1363.6288 | 10.518675 | 0.10568909 | 10.492126 | 10.635107 | 10.428791 |
| RAP2C | 0.007054303 | 0.13425232 | 1822.7253 | 10.11159 | 0.09180784 | 10.201633 | 10.018114 | 10.115024 |
| RARS | 0.001333401 | 0.04974301 | 932.1984 | 11.080887 | 0.033965103 | 11.061732 | 11.060827 | 11.120104 |
| RBBP7 | 0.002097827 | 0.06498393 | 1113.2601 | 10.818432 | 0.062418953 | 10.8421 | 10.747641 | 10.865556 |
| RBM14 | 0.002450539 | 0.07174599 | 1183.4313 | 10.726463 | 0.12935601 | 10.875758 | 10.647808 | 10.655821 |
| RBM22 | 0.001865193 | 0.060933147 | 1063.2582 | 10.884384 | 0.11608077 | 10.868151 | 11.007728 | 10.777275 |
| RBM23 | 0.006106647 | 0.12404215 | 1716.0013 | 10.197721 | 0.038463425 | 10.211628 | 10.227296 | 10.154238 |
| RBM25 | 0.004715715 | 0.10610052 | 1542.5391 | 10.347129 | 0.1728357 | 10.540575 | 10.292901 | 10.207909 |
| RBM47 | 0.006606531 | 0.1292035 | 1773.5062 | 10.147591 | 0.029503122 | 10.115079 | 10.17266 | 10.155033 |
| RBMX | 0.004152043 | 0.098665066 | 1464.0797 | 10.424395 | 0.022812478 | 10.411934 | 10.410526 | 10.450724 |
| RBX1 | 0.000939154 | 0.040028255 | 814.3905 | 11.262059 | 0.10082203 | 11.150343 | 11.346284 | 11.289552 |
| RCC2 | 0.000441402 | 0.0247 | 614.99756 | 11.625534 | 0.13139157 | 11.768599 | 11.510262 | 11.597744 |
| RDH11 | 0.008859861 | 0.15330873 | 2004.6881 | 9.971299 | 0.033412606 | 9.990079 | 9.932722 | 9.9910965 |
| RDX | 0.003387012 | 0.08717285 | 1347.3804 | 10.543031 | 0.08222902 | 10.47317 | 10.522272 | 10.63365 |
| REEP5 | 0.001967228 | 0.06267309 | 1085.4087 | 10.848609 | 0.10381348 | 10.728926 | 10.914301 | 10.902599 |
| REXO2 | 0.005442411 | 0.11513354 | 1636.2917 | 10.261994 | 0.0939313 | 10.1745205 | 10.361268 | 10.250195 |
| RFC4 | 0.004954546 | 0.109346695 | 1573.9937 | 10.321812 | 0.035669103 | 10.281321 | 10.3355255 | 10.348588 |
| RFWD3 | 0.00741575 | 0.13780503 | 1861.4448 | 10.078839 | 0.13074303 | 10.026343 | 9.982502 | 10.227671 |
| RHBDF1 | 0.00331824 | 0.0862991 | 1336.1467 | 10.5491495 | 0.07498614 | 10.526279 | 10.632907 | 10.48826 |
| RHEB | 0.001188073 | 0.046106264 | 891.1023 | 11.141331 | 0.06176753 | 11.071461 | 11.163865 | 11.188668 |
| RHOA | 0.00027512 | 0.018715687 | 517.20905 | 11.869443 | 0.12878829 | 11.868574 | 11.998665 | 11.741093 |
| RHOBTB3 | 0.000451461 | 0.025141252 | 620.27374 | 11.613059 | 0.06647728 | 11.546026 | 11.6789665 | 11.614182 |
| RHOC | 0.000441373 | 0.024738288 | 614.94336 | 11.625995 | 0.07620677 | 11.63694 | 11.544907 | 11.696136 |
| RHPN2 | 0.003925866 | 0.095581755 | 1431.4872 | 10.45715 | 0.05175091 | 10.42695 | 10.516907 | 10.427596 |
| RING1 | 0.001669251 | 0.057057142 | 1017.21484 | 10.938365 | 0.025287153 | 10.937626 | 10.964014 | 10.913456 |
| RIPK2 | 0.00542111 | 0.114822954 | 1633.6925 | 10.266113 | 0.061798137 | 10.308557 | 10.195213 | 10.29457 |
| RN7SL1 | 8.35879E-07 | 0.000329545 | 81.22692 | 13.8141985 | 0.0791142 | 13.904028 | 13.754895 | 13.783671 |
| RNASEK | 0.005361475 | 0.114117175 | 1626.3529 | 10.270202 | 0.037161548 | 10.307948 | 10.269003 | 10.233654 |
| RNASET2 | 0.000620251 | 0.031368803 | 697.4119 | 11.462746 | 0.08330687 | 11.404078 | 11.5581 | 11.426062 |
| RNF114 | 0.001357526 | 0.050211087 | 938.6761 | 11.063128 | 0.07429933 | 11.133988 | 10.985811 | 11.069586 |
| RNF181 | 0.000806076 | 0.03622539 | 768.8877 | 11.338928 | 0.15900776 | 11.220794 | 11.519719 | 11.276272 |
| RNGTT | 0.008717646 | 0.15236776 | 1990.7175 | 9.981435 | 0.2030865 | 10.06405 | 9.750061 | 10.130194 |
| RNU6-1 | 0.001331383 | 0.049828477 | 931.6763 | 11.070465 | 0.14969693 | 10.988223 | 10.9799185 | 11.243254 |
| RNU6-15 | 0.000764945 | 0.035011873 | 753.71014 | 11.361309 | 0.0642433 | 11.405386 | 11.3909445 | 11.287598 |
| ROBLD3 | 0.000368277 | 0.022337412 | 575.7015 | 11.716395 | 0.059361104 | 11.721001 | 11.77332 | 11.654866 |
| ROCK2 | 0.002127515 | 0.0655524 | 1119.2896 | 10.809685 | 0.044461243 | 10.80992 | 10.854027 | 10.765105 |
| ROD1 | 0.000770249 | 0.035023592 | 755.53674 | 11.35918 | 0.06944751 | 11.28849 | 11.361736 | 11.427315 |
| ROMO1 | 0.000268058 | 0.018343195 | 512.539 | 11.879921 | 0.3018763 | 11.536728 | 12.104369 | 11.998665 |
| RPA1 | 0.007433562 | 0.13784073 | 1863.2589 | 10.081552 | 0.07190758 | 10.066576 | 10.159768 | 10.0183115 |
| RPA2 | 0.002594944 | 0.074712865 | 1210.8698 | 10.692433 | 0.09846933 | 10.805594 | 10.626254 | 10.645452 |
| RPA3 | 0.00017663 | 0.013864254 | 440.33307 | 12.087257 | 0.10147232 | 12.02144 | 12.204117 | 12.036217 |
| RPAIN | 0.002912002 | 0.07980174 | 1267.8812 | 10.62128 | 0.026509568 | 10.622731 | 10.647033 | 10.594073 |
| RPF2 | 0.001761054 | 0.059088975 | 1039.3164 | 10.915013 | 0.1520569 | 10.81471 | 11.089968 | 10.840363 |
| RPL10A | 1.50458E-05 | 0.002704663 | 191.57327 | 13.148652 | 0.12563802 | 13.003809 | 13.213984 | 13.228164 |
| RPL11 | 0 | 0 | 31.40536 | 14.071007 | 0.120037585 | 14.187924 | 13.948075 | 14.077021 |
| RPL12 | 3.54528E-06 | 0.001042373 | 118.745445 | 13.581124 | 0.071040496 | 13.5420685 | 13.663124 | 13.53818 |
| RPL12P6 | 0.001570848 | 0.055160932 | 994.01447 | 10.983052 | 0.12679532 | 10.924298 | 10.896294 | 11.128569 |
| RPL13 | 0.006945524 | 0.13335252 | 1811.1617 | 10.121125 | 0.02541655 | 10.1216135 | 10.146295 | 10.0954685 |
| RPL13A | 6.75909E-05 | 0.007420886 | 316.00836 | 12.560842 | 0.07495087 | 12.573787 | 12.628476 | 12.480261 |
| RPL14 | 0.003160575 | 0.084089726 | 1309.7095 | 10.579223 | 0.077452675 | 10.640918 | 10.492302 | 10.604448 |
| RPL14L | 0.000103764 | 0.009809264 | 365.03732 | 12.360011 | 0.08235527 | 12.281255 | 12.445546 | 12.35323 |
| RPL15 | 0.00034395 | 0.021308929 | 561.5072 | 11.749317 | 0.102057956 | 11.843262 | 11.763962 | 11.640728 |
| RPL17 | 4.78181E-05 | 0.005862191 | 280.87366 | 12.701451 | 0.045613606 | 12.649052 | 12.732267 | 12.723035 |
| RPL18 | 0 | 0 | 12.01849 | 14.229716 | 0.12454459 | 14.304333 | 14.085939 | 14.298878 |
| RPL18A | 0 | 0 | 11.9721575 | 14.228906 | 0.09499352 | 14.328472 | 14.218984 | 14.139264 |
| RPL19 | 1.5997E-05 | 0.002817259 | 195.23427 | 13.113992 | 0.12973881 | 13.094524 | 13.252364 | 12.995087 |
| RPL21 | 1.66599E-05 | 0.00289 | 198.11105 | 13.111142 | 0.10378794 | 12.995087 | 13.143285 | 13.195058 |
| RPL22 | 0.001237765 | 0.047086623 | 905.55145 | 11.117467 | 0.051986437 | 11.060827 | 11.128569 | 11.163007 |
| RPL23 | 9.77979E-05 | 0.009425 | 357.86234 | 12.383708 | 0.053254694 | 12.441749 | 12.337094 | 12.372281 |
| RPL23A | 0.004943881 | 0.10918078 | 1572.5989 | 10.322419 | 0.17799583 | 10.288658 | 10.514877 | 10.163721 |
| RPL24 | 2.85352E-06 | 0.000891892 | 110.250946 | 13.631828 | 0.11543344 | 13.514325 | 13.636085 | 13.745074 |
| RPL26 | 2.36064E-05 | 0.00365625 | 221.64809 | 12.98589 | 0.007769613 | 12.9917555 | 12.977078 | 12.988837 |
| RPL26L1 | 0.00307007 | 0.08250426 | 1295.0028 | 10.597434 | 0.07004794 | 10.6432 | 10.516794 | 10.632307 |
| RPL27 | 6.57174E-06 | 0.0015 | 148.60915 | 13.384204 | 0.08446006 | 13.312153 | 13.47715 | 13.363307 |
| RPL27A | 2.68058E-06 | 0.000853211 | 108.24522 | 13.640935 | 0.07155564 | 13.619867 | 13.720659 | 13.582278 |
| RPL3 | 1.25382E-05 | 0.00244382 | 180.95932 | 13.199803 | 0.07801839 | 13.242086 | 13.109771 | 13.247553 |
| RPL30 | 0 | 0 | 14.479957 | 14.199436 | 0.15098837 | 14.153474 | 14.076771 | 14.368065 |
| RPL31 | 3.74705E-07 | 0.000178082 | 67.43945 | 13.87484 | 0.12398016 | 13.88736 | 13.745074 | 13.9920845 |
| RPL34 | 0.009388885 | 0.15874171 | 2053.9146 | 9.936782 | 0.08160353 | 9.868909 | 9.914113 | 10.027323 |
| RPL35 | 4.32351E-07 | 0.000189873 | 71.13786 | 13.863027 | 0.13281502 | 13.863877 | 13.995416 | 13.72979 |
| RPL35A | 8.35879E-07 | 0.000329545 | 80.983536 | 13.811023 | 0.016355712 | 13.810165 | 13.795114 | 13.827791 |
| RPL36 | 0.005254914 | 0.11267862 | 1612.5359 | 10.282802 | 0.08083993 | 10.236417 | 10.376146 | 10.23584 |
| RPL36AL | 2.27705E-05 | 0.003607306 | 219.20595 | 13.00333 | 0.085239306 | 13.031326 | 13.071051 | 12.907614 |
| RPL37A | 0.00295737 | 0.08040988 | 1275.9617 | 10.612594 | 0.016430076 | 10.614489 | 10.627994 | 10.595298 |
| RPL38 | 2.88234E-08 | 2.43902E-05 | 40.462955 | 14.035034 | 0.09010742 | 14.013335 | 14.134009 | 13.957757 |
| RPL39 | 1.29705E-06 | 0.000494506 | 90.84391 | 13.751991 | 0.04835519 | 13.79883 | 13.7022505 | 13.754895 |
| RPL39L | 0.008706779 | 0.15233132 | 1989.6547 | 9.982718 | 0.03531121 | 9.965038 | 9.95974 | 10.023377 |
| RPL4 | 3.80181E-05 | 0.005073077 | 259.2865 | 12.800378 | 0.022620192 | 12.777228 | 12.822429 | 12.8014765 |
| RPL41 | 0.0014559 | 0.05250624 | 964.80035 | 11.026847 | 0.13555662 | 10.876425 | 11.064567 | 11.139548 |
| RPL5 | 5.05851E-05 | 0.006136364 | 286.06436 | 12.676345 | 0.057915445 | 12.634005 | 12.652684 | 12.742344 |
| RPL6 | 6.21433E-05 | 0.007092105 | 305.96634 | 12.60039 | 0.03358865 | 12.609609 | 12.6284075 | 12.563155 |
| RPL7 | 0.000353231 | 0.021728724 | 566.91187 | 11.73777 | 0.0701732 | 11.733788 | 11.80985 | 11.669673 |
| RPL7A | 1.65735E-05 | 0.002889447 | 197.5033 | 13.115293 | 0.09537753 | 13.1631 | 13.005466 | 13.177312 |
| RPL7L1 | 0.000895947 | 0.038709838 | 799.9595 | 11.286395 | 0.011775699 | 11.284201 | 11.275869 | 11.299112 |
| RPL8 | 0.000304606 | 0.019864662 | 537.4794 | 11.814957 | 0.16664146 | 11.78162 | 11.995746 | 11.667502 |
| RPL9 | 0.000232432 | 0.016490798 | 487.4691 | 11.959495 | 0.05280028 | 11.903713 | 12.0086975 | 11.966071 |
| RPLP0 | 0.002257249 | 0.06833595 | 1145.9025 | 10.778293 | 0.023723725 | 10.787009 | 10.751443 | 10.796424 |
| RPLP1 | 3.64616E-05 | 0.004941406 | 255.95224 | 12.822726 | 0.023877023 | 12.829709 | 12.796136 | 12.842333 |
| RPLP2 | 3.17058E-07 | 0.000164179 | 59.19594 | 13.932464 | 0.09115905 | 14.033582 | 13.9072275 | 13.856581 |
| RPN1 | 0.000284084 | 0.019175097 | 523.21124 | 11.852101 | 0.10274067 | 11.748696 | 11.9541645 | 11.853444 |
| RPN2 | 0.003099844 | 0.083111286 | 1299.8073 | 10.588871 | 0.11079251 | 10.465931 | 10.680988 | 10.619695 |
| RPP21 | 0.000102986 | 0.009762296 | 363.87698 | 12.35899 | 0.05268994 | 12.419626 | 12.324357 | 12.332985 |
| RPP40 | 0.001484061 | 0.05313519 | 972.264 | 11.00729 | 0.10071425 | 10.971323 | 11.12105 | 10.929497 |
| RPRC1 | 0.006462645 | 0.1279766 | 1757.2938 | 10.163559 | 0.10628642 | 10.284521 | 10.121048 | 10.085108 |
| RPS10 | 2.88234E-08 | 2.43902E-05 | 44.9466 | 14.00368 | 0.10548321 | 13.955703 | 14.124624 | 13.930714 |
| RPS11 | 0 | 0 | 24.496597 | 14.136434 | 0.058763053 | 14.089321 | 14.1177025 | 14.20228 |
| RPS12 | 0 | 0 | 7.268482 | 14.286308 | 0.048187852 | 14.269613 | 14.340624 | 14.248689 |
| RPS13 | 3.94881E-06 | 0.001096 | 123.98131 | 13.55748 | 0.03247833 | 13.586366 | 13.522324 | 13.563751 |
| RPS14 | 7.20586E-07 | 0.000297619 | 76.79888 | 13.8364105 | 0.04829387 | 13.873062 | 13.781687 | 13.854482 |
| RPS15 | 0.001024961 | 0.04243437 | 840.9865 | 11.216643 | 0.0910545 | 11.321744 | 11.16158 | 11.166606 |
| RPS15A | 5.93763E-06 | 0.00138255 | 145.30014 | 13.420516 | 0.028061375 | 13.402529 | 13.406167 | 13.452849 |
| RPS16 | 3.45881E-07 | 0.000169014 | 66.36165 | 13.879947 | 0.14116728 | 13.827791 | 14.039771 | 13.772279 |
| RPS17 | 7.20586E-07 | 0.000297619 | 77.20472 | 13.813816 | 0.09613973 | 13.72888 | 13.794378 | 13.918189 |
| RPS18 | 3.54528E-06 | 0.001042373 | 118.26854 | 13.584809 | 0.08790377 | 13.615455 | 13.653289 | 13.485685 |
| RPS19 | 2.88234E-08 | 2.43902E-05 | 44.105804 | 14.007468 | 0.10740012 | 13.920511 | 13.97437 | 14.127521 |
| RPS2 | 5.47645E-07 | 0.000231707 | 74.764 | 13.817715 | 0.12236853 | 13.713431 | 13.787285 | 13.952426 |
| RPS20 | 5.76469E-08 | 0.00004 | 47.569057 | 13.997022 | 0.107310586 | 14.073897 | 14.042745 | 13.874422 |
| RPS21 | 0.000206866 | 0.015434409 | 466.50867 | 12.017179 | 0.10327149 | 12.136281 | 11.952528 | 11.962728 |
| RPS24 | 0.00022367 | 0.016268345 | 480.21887 | 11.9754715 | 0.08871622 | 11.887067 | 12.064496 | 11.9748535 |
| RPS25 | 2.88234E-08 | 2.43902E-05 | 37.20028 | 14.034886 | 0.16215895 | 13.850101 | 14.153474 | 14.101088 |
| RPS26L | 8.02733E-05 | 0.008338324 | 334.27356 | 12.486096 | 0.026707508 | 12.512563 | 12.459154 | 12.486574 |
| RPS26P11 | 0.009174497 | 0.15656665 | 2034.377 | 9.952922 | 0.053278904 | 9.983844 | 9.98352 | 9.8914 |
| RPS27 | 2.56529E-06 | 0.000824074 | 106.54002 | 13.6585455 | 0.034225788 | 13.670116 | 13.620033 | 13.685485 |
| RPS27A | 3.37234E-06 | 0.001026316 | 117.1005 | 13.591525 | 0.048668873 | 13.642432 | 13.586686 | 13.545456 |
| RPS28 | 0.000136133 | 0.0118075 | 402.05875 | 12.210434 | 0.09253379 | 12.2890215 | 12.108446 | 12.233832 |
| RPS29 | 2.27417E-05 | 0.003619266 | 218.86078 | 12.992112 | 0.044963345 | 13.033921 | 12.997867 | 12.944549 |
| RPS3 | 2.24823E-06 | 0.000795918 | 102.12339 | 13.673374 | 0.17403445 | 13.653289 | 13.856581 | 13.510255 |
| RPS3A | 1.52764E-06 | 0.000569892 | 95.35307 | 13.715103 | 0.11137722 | 13.607775 | 13.707403 | 13.83013 |
| RPS4X | 3.33199E-05 | 0.00477686 | 248.08868 | 12.841855 | 0.059511207 | 12.840137 | 12.902207 | 12.783222 |
| RPS5 | 7.83997E-06 | 0.001732484 | 156.43932 | 13.336011 | 0.06654825 | 13.294025 | 13.412741 | 13.301268 |
| RPS6 | 2.91117E-06 | 0.000901786 | 111.62438 | 13.627955 | 0.014264934 | 13.635286 | 13.637063 | 13.611515 |
| RPS6KB1 | 4.47916E-05 | 0.005650909 | 274.4588 | 12.734832 | 0.062097263 | 12.674491 | 12.731456 | 12.798548 |
| RPS8 | 7.20586E-07 | 0.000297619 | 77.59405 | 13.737831 | 0.32543197 | 13.436263 | 13.694453 | 14.082776 |
| RPS9 | 4.55122E-05 | 0.005679856 | 275.70615 | 12.72672 | 0.08943363 | 12.6959 | 12.827489 | 12.656774 |
| RPSA | 0.002548077 | 0.0738538 | 1201.7328 | 10.704276 | 0.039274838 | 10.725302 | 10.658964 | 10.728561 |
| RRAGA | 0.004147691 | 0.09869684 | 1463.4208 | 10.4244795 | 0.03577862 | 10.409853 | 10.465255 | 10.398333 |
| RRM1 | 0.001189716 | 0.046118435 | 891.52246 | 11.134514 | 0.15744855 | 11.016044 | 11.31318 | 11.07432 |
| RRP15 | 0.004245576 | 0.099929444 | 1477.2438 | 10.41147 | 0.033403177 | 10.38159 | 10.405289 | 10.447533 |
| RRP7B | 2.59411E-07 | 0.000157895 | 56.562363 | 13.937877 | 0.106811024 | 13.952426 | 13.824537 | 14.036668 |
| RSL1D1 | 0.00242944 | 0.07142966 | 1179.3527 | 10.730502 | 0.1460409 | 10.842996 | 10.783053 | 10.565458 |
| RSL24D1 | 0.00066781 | 0.032268804 | 716.4963 | 11.42263 | 0.05429908 | 11.431086 | 11.364598 | 11.472204 |
| RUSC1 | 0.003987202 | 0.09633148 | 1440.1858 | 10.447274 | 0.08415491 | 10.476541 | 10.512888 | 10.352393 |
| RXRA | 0.002185681 | 0.066459246 | 1130.9966 | 10.792565 | 0.060224537 | 10.77571 | 10.859422 | 10.742564 |
| S100A10 | 8.30115E-06 | 0.0018 | 158.94978 | 13.336417 | 0.086572595 | 13.436142 | 13.292554 | 13.280555 |
| S100A11 | 0.000904912 | 0.039048508 | 803.37244 | 11.283443 | 0.06403375 | 11.250868 | 11.357216 | 11.242248 |
| S100P | 0.002051911 | 0.06425 | 1103.4435 | 10.8266325 | 0.090051554 | 10.909029 | 10.840363 | 10.730503 |
| SAC3D1 | 0.00857595 | 0.15072644 | 1977.1049 | 9.99515 | 0.12223575 | 10.028999 | 9.859556 | 10.096893 |
| SAE1 | 5.84539E-05 | 0.006805369 | 300.44263 | 12.630074 | 0.08908644 | 12.5562315 | 12.729018 | 12.604972 |
| SAP30L | 0.003708509 | 0.092099495 | 1398.1603 | 10.487323 | 0.15549137 | 10.5838 | 10.307948 | 10.57022 |
| SAT1 | 0.005413213 | 0.11472572 | 1632.6974 | 10.26585 | 0.044897996 | 10.315745 | 10.22871 | 10.253093 |
| SCAP | 0.009210642 | 0.15656736 | 2037.6003 | 9.947821 | 0.0684805 | 9.961304 | 10.008557 | 9.873602 |
| SCARB2 | 0.001519225 | 0.0540041 | 981.2371 | 11.001872 | 0.054357886 | 11.028784 | 10.939308 | 11.037524 |
| SCD | 0.000142128 | 0.01214532 | 408.8717 | 12.190179 | 0.039780542 | 12.186669 | 12.152269 | 12.231598 |
| SCO1 | 0.006027786 | 0.123233944 | 1706.6969 | 10.208313 | 0.14071788 | 10.264684 | 10.312107 | 10.04815 |
| SCRN1 | 0.009008676 | 0.15472624 | 2018.8492 | 9.964805 | 0.06309647 | 10.02844 | 9.902262 | 9.963712 |
| SCYL1 | 0.005032282 | 0.11029059 | 1584.2772 | 10.308133 | 0.04211903 | 10.343333 | 10.319597 | 10.261469 |
| SCYL2 | 0.007316366 | 0.13705939 | 1850.842 | 10.088436 | 0.07475838 | 10.068253 | 10.025842 | 10.171214 |
| SDAD1 | 0.008118234 | 0.14585914 | 1932.6248 | 10.0275955 | 0.09373391 | 9.922734 | 10.103245 | 10.056808 |
| SDF2 | 0.004102409 | 0.09802273 | 1456.8826 | 10.428314 | 0.014690463 | 10.440322 | 10.411934 | 10.432687 |
| SDF2L1 | 0.003540612 | 0.089401744 | 1372.0182 | 10.515521 | 0.030164998 | 10.511426 | 10.487612 | 10.5475235 |
| SDHAF2 | 0.001498674 | 0.053603094 | 975.98926 | 11.00916 | 0.06902553 | 11.065523 | 10.932174 | 11.029784 |
| SDHB | 0.001590246 | 0.055393573 | 998.61 | 10.973027 | 0.07488162 | 10.976883 | 11.045908 | 10.896294 |
| SDHD | 0.007583415 | 0.13942714 | 1878.6241 | 10.068629 | 0.034453567 | 10.081778 | 10.029537 | 10.094572 |
| SEC11A | 8.41068E-05 | 0.008532164 | 340.31845 | 12.454787 | 0.13718097 | 12.385681 | 12.612778 | 12.365902 |
| SEC11C | 0.004854586 | 0.10789558 | 1561.041 | 10.33183 | 0.18374605 | 10.276138 | 10.536979 | 10.182372 |
| SEC23B | 0.004020465 | 0.09666389 | 1444.9801 | 10.441115 | 0.03194947 | 10.470136 | 10.406878 | 10.44633 |
| SEC61A1 | 0.000842739 | 0.037104063 | 781.9817 | 11.3110695 | 0.009857466 | 11.299687 | 11.316761 | 11.316761 |
| SEC61B | 7.0358E-05 | 0.007604362 | 320.31293 | 12.533498 | 0.21036169 | 12.609895 | 12.295613 | 12.694986 |
| SEC61G | 7.46815E-05 | 0.007851515 | 326.76172 | 12.512557 | 0.15026219 | 12.424328 | 12.686056 | 12.427286 |
| Selenoprotein 15 | 0.002871707 | 0.079450555 | 1260.7448 | 10.6309395 | 0.07306325 | 10.713406 | 10.6051235 | 10.574288 |
| SEMA3C | 0.008270161 | 0.14759517 | 1947.4258 | 10.013648 | 0.16267057 | 10.190192 | 9.869826 | 9.980924 |
| SEPHS2 | 0.001025048 | 0.042387366 | 841.05554 | 11.217122 | 0.044080365 | 11.166977 | 11.234637 | 11.249754 |
| Septin 9 | 0.00011146 | 0.010284575 | 373.6677 | 12.332024 | 0.2044217 | 12.363478 | 12.518894 | 12.113697 |
| SEPW1 | 0.003384908 | 0.08718337 | 1347.0526 | 10.539711 | 0.08979818 | 10.643343 | 10.490899 | 10.484891 |
| SEPX1 | 0.002510405 | 0.07312846 | 1194.5111 | 10.713183 | 0.053283025 | 10.774172 | 10.675663 | 10.689714 |
| SERF2 | 0.004659509 | 0.10586575 | 1534.7783 | 10.3579 | 0.06764454 | 10.3973 | 10.396606 | 10.279791 |
| SERPINA3 | 0.002422782 | 0.07141546 | 1178.0231 | 10.735249 | 0.064773984 | 10.664375 | 10.749989 | 10.791383 |
| SET | 0.0001977 | 0.014975983 | 458.9545 | 12.034307 | 0.015925137 | 12.039725 | 12.046817 | 12.01638 |
| SF3A3 | 0.003804116 | 0.09333805 | 1412.8574 | 10.471844 | 0.03973563 | 10.481444 | 10.428187 | 10.505899 |
| SF3B14 | 0.001253473 | 0.04757987 | 909.81964 | 11.114297 | 0.09204342 | 11.015882 | 11.198257 | 11.128755 |
| SF3B2 | 0.00562544 | 0.1175009 | 1658.9806 | 10.245873 | 0.07481341 | 10.283319 | 10.29457 | 10.159731 |
| SF3B3 | 0.00417663 | 0.0990458 | 1467.6437 | 10.418826 | 0.10674579 | 10.5155735 | 10.436595 | 10.304312 |
| SF3B4 | 0.000336917 | 0.021023382 | 557.7238 | 11.76118 | 0.14901192 | 11.90285 | 11.605777 | 11.77491 |
| SF3B5 | 0.002341529 | 0.06991136 | 1162.1681 | 10.756729 | 0.030248733 | 10.77488 | 10.773498 | 10.72181 |
| SFRS1 | 0.000121664 | 0.010992187 | 386.82898 | 12.272037 | 0.054221652 | 12.253972 | 12.332985 | 12.229154 |
| SFRS10 | 0.001418747 | 0.05164953 | 954.7955 | 11.036579 | 0.1335939 | 11.010458 | 10.917974 | 11.181304 |
| SFRS2 | 0.000290857 | 0.019368522 | 527.7927 | 11.84179 | 0.06131811 | 11.790283 | 11.909617 | 11.825471 |
| SFRS4 | 0.00636551 | 0.12692241 | 1746.1226 | 10.169246 | 0.06375451 | 10.185272 | 10.099007 | 10.223457 |
| SFRS5 | 0.000661671 | 0.032196354 | 714.16626 | 11.430043 | 0.05901756 | 11.38689 | 11.497297 | 11.405942 |
| SFRS6 | 0.000384822 | 0.022783276 | 584.73285 | 11.699441 | 0.14957403 | 11.63606 | 11.870271 | 11.591994 |
| SFRS9 | 0.00017712 | 0.013840091 | 440.66565 | 12.091542 | 0.06944939 | 12.092869 | 12.02144 | 12.160319 |
| SFT2D1 | 0.002659567 | 0.075818405 | 1222.6317 | 10.682162 | 0.06479437 | 10.611014 | 10.737781 | 10.697693 |
| SGSM2 | 0.00094195 | 0.04 | 815.274 | 11.256561 | 0.10103021 | 11.344073 | 11.145997 | 11.2796135 |
| SH3GLB2 | 0.000626333 | 0.03153846 | 699.96826 | 11.45906 | 0.13154285 | 11.601837 | 11.342789 | 11.432551 |
| SHCBP1 | 0.009062604 | 0.15519151 | 2023.8135 | 9.959652 | 0.041436058 | 9.927792 | 10.006495 | 9.944669 |
| SHFM1 | 0.001439038 | 0.05206048 | 960.29584 | 11.031932 | 0.07077718 | 11.043243 | 11.096373 | 10.956181 |
| SHISA5 | 0.005836802 | 0.1204652 | 1684.0328 | 10.224486 | 0.18624532 | 10.167042 | 10.432687 | 10.07373 |
| SHMT2 | 0.00278247 | 0.07803961 | 1244.5621 | 10.653478 | 0.048810456 | 10.706045 | 10.609588 | 10.644803 |
| SHPK | 0.003528823 | 0.08916897 | 1370.0988 | 10.516364 | 0.042128015 | 10.509977 | 10.477795 | 10.561321 |
| SIVA | 0.000813887 | 0.03643484 | 771.924 | 11.330358 | 0.027315982 | 11.321134 | 11.361093 | 11.308849 |
| SIVA1 | 0.000701274 | 0.033374485 | 729.62024 | 11.398873 | 0.04609813 | 11.452095 | 11.37307 | 11.371456 |
| SLC12A9 | 0.008962011 | 0.15423016 | 2014.5006 | 9.968053 | 0.09087078 | 10.037174 | 9.865125 | 10.001862 |
| SLC25A39 | 0.00039416 | 0.023021886 | 589.7573 | 11.684799 | 0.09745731 | 11.660432 | 11.792128 | 11.601837 |
| SLC25A5 | 1.8447E-06 | 0.000666667 | 98.30148 | 13.700336 | 0.032391626 | 13.697969 | 13.669193 | 13.733847 |
| SLC25A6 | 0.001021618 | 0.04239713 | 839.8944 | 11.223281 | 0.09156625 | 11.237891 | 11.306663 | 11.125287 |
| SLC27A3 | 0.005689716 | 0.11848679 | 1666.4028 | 10.236653 | 0.040781863 | 10.222119 | 10.282712 | 10.205131 |
| SLC2A8 | 0.00806419 | 0.14526428 | 1927.2605 | 10.031855 | 0.033424277 | 10.070417 | 10.013932 | 10.011213 |
| SLC30A9 | 0.009378769 | 0.15864797 | 2053.0369 | 9.941136 | 0.16281235 | 9.760593 | 10.076807 | 9.986008 |
| SLC35A1 | 0.003056494 | 0.08245879 | 1292.847 | 10.593301 | 0.025809094 | 10.622513 | 10.5838 | 10.573587 |
| SLC35B1 | 0.000861158 | 0.037628464 | 788.47815 | 11.302963 | 0.03463224 | 11.2631035 | 11.32009 | 11.325693 |
| SLC35B2 | 0.009071598 | 0.15526886 | 2024.6794 | 9.959304 | 0.029486772 | 9.97585 | 9.976803 | 9.925261 |
| SLC37A4 | 0.001539661 | 0.054340795 | 986.7445 | 10.990855 | 0.081573024 | 11.085023 | 10.94195 | 10.945589 |
| SLC38A2 | 0.001398859 | 0.051194094 | 949.90845 | 11.044258 | 0.057873923 | 11.109128 | 10.99792 | 11.025725 |
| SLC39A1 | 0.005858967 | 0.12070724 | 1686.5614 | 10.22168 | 0.054114725 | 10.27255 | 10.227671 | 10.164819 |
| SLC39A6 | 0.001137603 | 0.04500342 | 876.4563 | 11.162174 | 0.0713175 | 11.091746 | 11.234349 | 11.160428 |
| SLC44A1 | 0.002311898 | 0.06956548 | 1156.2495 | 10.763701 | 0.023373034 | 10.771481 | 10.782191 | 10.73743 |
| SLC44A2 | 0.002700467 | 0.07673219 | 1230.081 | 10.669266 | 0.06892085 | 10.742564 | 10.605773 | 10.659459 |
| SLC7A5 | 0.00349265 | 0.08857749 | 1364.2816 | 10.520004 | 0.14064772 | 10.518228 | 10.661531 | 10.380253 |
| SLC9A1 | 0.006593849 | 0.1290282 | 1771.9464 | 10.151134 | 0.1551922 | 10.330232 | 10.056347 | 10.066821 |
| SLC9A3R1 | 0.000316856 | 0.02020772 | 545.3575 | 11.799609 | 0.114697985 | 11.851962 | 11.87879 | 11.6680765 |
| SMARCA4 | 0.00140886 | 0.051397476 | 952.342 | 11.041766 | 0.048082303 | 11.096771 | 11.020799 | 11.007728 |
| SMARCD2 | 0.006927797 | 0.13315956 | 1809.1268 | 10.121388 | 0.079000756 | 10.038889 | 10.128926 | 10.19635 |
| SMS | 0.002058742 | 0.06434775 | 1104.7859 | 10.829277 | 0.053252272 | 10.877017 | 10.771844 | 10.838969 |
| SMUG1 | 0.005707961 | 0.11865309 | 1668.7677 | 10.235465 | 0.029388323 | 10.202155 | 10.257734 | 10.246505 |
| SNF8 | 0.000947282 | 0.040128205 | 816.9039 | 11.258201 | 0.1336459 | 11.306663 | 11.360854 | 11.107084 |
| SNHG1 | 0.005160691 | 0.1116937 | 1600.693 | 10.298221 | 0.22397743 | 10.274262 | 10.533214 | 10.087185 |
| SNHG5 | 0.000109587 | 0.010193029 | 371.77682 | 12.326497 | 0.070476174 | 12.301632 | 12.271824 | 12.406035 |
| SNHG6 | 0.001170462 | 0.045832958 | 886.2912 | 11.145381 | 0.050751317 | 11.194152 | 11.092857 | 11.149131 |
| SNHG9 | 0.005245633 | 0.11268854 | 1611.36 | 10.28991 | 0.15823749 | 10.392907 | 10.107711 | 10.369114 |
| SNORA12 | 0.008830633 | 0.15303198 | 2001.8481 | 9.975616 | 0.09005358 | 10.05107 | 9.875924 | 9.999854 |
| SNORD13 | 0.006353231 | 0.12682337 | 1744.7074 | 10.175595 | 0.058686737 | 10.223997 | 10.192468 | 10.11032 |
| SNRPA1 | 0.00015164 | 0.012646635 | 417.64648 | 12.157687 | 0.01716648 | 12.154094 | 12.142603 | 12.176367 |
| SNRPB | 0.000404306 | 0.023378333 | 595.2519 | 11.674179 | 0.026935378 | 11.702969 | 11.649592 | 11.669973 |
| SNRPB2 | 0.001400242 | 0.051190726 | 950.27893 | 11.044884 | 0.08778884 | 10.986244 | 11.145814 | 11.002596 |
| SNRPD2 | 0.004412838 | 0.1023389 | 1500.6724 | 10.38818 | 0.046467476 | 10.431226 | 10.338917 | 10.394397 |
| SNRPF | 9.36761E-05 | 0.009180791 | 352.2933 | 12.412563 | 0.034413688 | 12.437401 | 12.427008 | 12.3732815 |
| SNRPG | 1.23653E-05 | 0.002423729 | 180.36665 | 13.190248 | 0.0795024 | 13.12537 | 13.16644 | 13.2789345 |
| SNX2 | 0.007516142 | 0.13877861 | 1871.7726 | 10.073895 | 0.1272764 | 9.966744 | 10.2145815 | 10.0403595 |
| SNX27 | 0.001128754 | 0.044960964 | 873.81946 | 11.16354 | 0.13455273 | 11.251786 | 11.008675 | 11.23016 |
| SOD1 | 1.59394E-05 | 0.002821429 | 195.09618 | 13.114604 | 0.13883232 | 13.100678 | 13.259873 | 12.983258 |
| SOX4 | 0.001903788 | 0.061613806 | 1071.6119 | 10.8726225 | 0.0973703 | 10.844779 | 10.792206 | 10.980881 |
| SPC24 | 0.002567591 | 0.07398671 | 1205.6199 | 10.699554 | 0.14078015 | 10.810585 | 10.746865 | 10.541213 |
| SPCS1 | 0.001154234 | 0.04545403 | 881.4217 | 11.152476 | 0.13271618 | 11.00574 | 11.26412 | 11.187567 |
| SPINT2 | 8.07921E-05 | 0.008367164 | 335.18936 | 12.487418 | 0.047298707 | 12.494312 | 12.53089 | 12.43705 |
| SPIRE1 | 0.008457889 | 0.14925636 | 1965.7744 | 10.002851 | 0.1164978 | 9.881673 | 10.114023 | 10.012855 |
| SPNS1 | 0.008019571 | 0.14468591 | 1922.844 | 10.032067 | 0.17596439 | 10.229272 | 9.891082 | 9.97585 |
| SPPL2A | 0.001715599 | 0.058182795 | 1028.7339 | 10.927928 | 0.041376572 | 10.891755 | 10.918984 | 10.973045 |
| SPSB3 | 0.008472099 | 0.14935519 | 1967.0848 | 9.999803 | 0.11344413 | 10.110853 | 9.884108 | 10.004447 |
| SQLE | 0.006566899 | 0.12871864 | 1768.9498 | 10.153492 | 0.054473944 | 10.120537 | 10.216369 | 10.12357 |
| SQSTM1 | 0.000108347 | 0.010132075 | 370.21783 | 12.338089 | 0.053861544 | 12.282698 | 12.390278 | 12.341291 |
| SRF | 0.008394419 | 0.14851402 | 1959.5347 | 10.010066 | 0.06073306 | 10.008557 | 10.071539 | 9.950101 |
| SRP14 | 0.000922263 | 0.039502468 | 809.0499 | 11.2717085 | 0.041310348 | 11.25271 | 11.319099 | 11.243314 |
| SRP14P1 | 0.000527613 | 0.028204931 | 656.34326 | 11.529517 | 0.055244222 | 11.466108 | 11.567258 | 11.555184 |
| SRP19 | 0.004096328 | 0.09794486 | 1455.9517 | 10.430238 | 0.048866954 | 10.449376 | 10.374699 | 10.4666395 |
| SRP9 | 0.003281864 | 0.08567419 | 1330.0083 | 10.558642 | 0.027370641 | 10.558209 | 10.531491 | 10.586227 |
| SRPK1 | 0.008253127 | 0.14744285 | 1945.8073 | 10.016757 | 0.073939994 | 9.934574 | 10.037808 | 10.0778885 |
| SRPRB | 0.006855854 | 0.13236338 | 1801.2666 | 10.127355 | 0.042087387 | 10.089824 | 10.172858 | 10.119383 |
| SRRM1 | 0.001525768 | 0.053960245 | 983.0401 | 10.990833 | 0.06784813 | 10.933008 | 10.9739685 | 11.065523 |
| SRRM2 | 0.004879374 | 0.10810026 | 1564.2673 | 10.328355 | 0.09757892 | 10.441022 | 10.27316 | 10.270884 |
| SS18L2 | 0.000637747 | 0.03160857 | 704.42 | 11.445121 | 0.14731589 | 11.292739 | 11.455838 | 11.586785 |
| SSBP1 | 0.000635557 | 0.031726617 | 703.5078 | 11.449437 | 0.054189343 | 11.402953 | 11.436405 | 11.508955 |
| SSR1 | 0.003743904 | 0.0925809 | 1403.3597 | 10.484561 | 0.048651036 | 10.442165 | 10.473839 | 10.537679 |
| SSR4 | 0.000124488 | 0.011046036 | 390.00366 | 12.260737 | 0.09067214 | 12.317703 | 12.3083315 | 12.156178 |
| SSU72 | 0.001114977 | 0.04482387 | 869.4271 | 11.17439 | 0.04095926 | 11.169169 | 11.217709 | 11.136291 |
| STARD10 | 0.000660662 | 0.032192416 | 713.6569 | 11.429713 | 0.090419516 | 11.531432 | 11.358465 | 11.399244 |
| STAT1 | 0.001514066 | 0.05393121 | 979.98364 | 10.9974165 | 0.009495248 | 10.990284 | 11.008194 | 10.993771 |
| STC2 | 0.002160604 | 0.066044055 | 1125.8463 | 10.799012 | 0.13929917 | 10.926099 | 10.650082 | 10.820857 |
| STIP1 | 0.00477365 | 0.10684968 | 1550.3937 | 10.338042 | 0.094199724 | 10.299022 | 10.445482 | 10.269623 |
| STK3 | 0.007010377 | 0.13378328 | 1818.1489 | 10.11652 | 0.029662969 | 10.08284 | 10.138757 | 10.127964 |
| STK4 | 0.007074653 | 0.13449205 | 1825.0105 | 10.112132 | 0.08610892 | 10.2056675 | 10.094572 | 10.036157 |
| STMN3 | 0.00187211 | 0.06104417 | 1064.7622 | 10.879205 | 0.069739096 | 10.827543 | 10.958532 | 10.851541 |
| STOML2 | 0.001382083 | 0.050956428 | 945.6237 | 11.058235 | 0.016614012 | 11.077129 | 11.05167 | 11.045908 |
| STRA13 | 0.000371707 | 0.0224669 | 577.66846 | 11.709854 | 0.05496945 | 11.77332 | 11.677277 | 11.6789665 |
| STRAP | 0.001177466 | 0.04600338 | 888.3178 | 11.144868 | 0.009351316 | 11.140002 | 11.138953 | 11.155649 |
| STRN3 | 0.009711881 | 0.16261776 | 2083.0361 | 9.914987 | 0.09121148 | 9.932152 | 9.996397 | 9.816413 |
| SUCLG1 | 0.00103969 | 0.04273815 | 845.65576 | 11.205025 | 0.19349372 | 11.023831 | 11.408832 | 11.182411 |
| SUCLG2 | 0.009511241 | 0.16026372 | 2064.8408 | 9.92763 | 0.10532392 | 9.976803 | 9.806713 | 9.999375 |
| SULF2 | 0.003255779 | 0.08524981 | 1325.6807 | 10.560763 | 0.039907254 | 10.558898 | 10.60157 | 10.521821 |
| SUMF2 | 0.009755318 | 0.16295186 | 2086.9866 | 9.913063 | 0.058542814 | 9.903062 | 9.975963 | 9.860166 |
| SUMO2 | 0.000670318 | 0.032344922 | 717.4424 | 11.425862 | 0.038857184 | 11.38299 | 11.435841 | 11.458758 |
| SUMO3 | 6.27198E-05 | 0.007087948 | 307.3548 | 12.595051 | 0.12386172 | 12.475793 | 12.723053 | 12.586308 |
| SUPT16H | 0.001781518 | 0.05914641 | 1044.1299 | 10.910874 | 0.11827189 | 10.853221 | 10.832486 | 11.046918 |
| SURF4 | 0.009926299 | 0.16438329 | 2102.2983 | 9.902531 | 0.057131544 | 9.873602 | 9.968341 | 9.86565 |
| SURF6 | 0.008954056 | 0.15416972 | 2013.7393 | 9.965255 | 0.08067925 | 9.992218 | 10.028999 | 9.874547 |
| SUZ12 | 0.002920419 | 0.07971755 | 1269.3467 | 10.626427 | 0.09742411 | 10.521652 | 10.714285 | 10.643343 |
| SYNCRIP | 0.000634 | 0.031694524 | 702.86694 | 11.4526 | 0.078235 | 11.387373 | 11.539341 | 11.431086 |
| TACO1 | 0.00158206 | 0.055330645 | 996.5183 | 10.974037 | 0.054170225 | 11.000861 | 10.911689 | 11.009562 |
| TACSTD1 | 0.000326079 | 0.020606557 | 551.1968 | 11.773488 | 0.039720867 | 11.817687 | 11.740778 | 11.761999 |
| TACSTD2 | 6.26333E-05 | 0.007101307 | 307.0716 | 12.591896 | 0.19662125 | 12.61791 | 12.383563 | 12.774216 |
| TAF4 | 0.007224102 | 0.1361396 | 1841.0549 | 10.096012 | 0.029216753 | 10.095109 | 10.067258 | 10.12567 |
| TARS | 0.008896956 | 0.1536441 | 2008.3945 | 9.970433 | 0.15063362 | 10.033129 | 9.798577 | 10.079593 |
| TATDN1 | 0.005315818 | 0.113563426 | 1620.359 | 10.276624 | 0.09000053 | 10.190929 | 10.370387 | 10.268555 |
| TAX1BP1 | 0.003174094 | 0.08425555 | 1312.1179 | 10.576461 | 0.028607104 | 10.609231 | 10.556477 | 10.563675 |
| TAX1BP3 | 0.002096386 | 0.06505546 | 1112.9705 | 10.816602 | 0.11131406 | 10.8927965 | 10.868151 | 10.688857 |
| TBC1D7 | 0.009007062 | 0.15477514 | 2018.7104 | 9.962276 | 0.12515071 | 9.995792 | 9.823781 | 10.067258 |
| TBC1D9 | 0.0023971 | 0.070959896 | 1173.221 | 10.738194 | 0.10015027 | 10.650082 | 10.717388 | 10.847114 |
| TBCA | 2.85064E-05 | 0.004262931 | 235.89388 | 12.920265 | 0.08746732 | 12.967685 | 12.973782 | 12.819327 |
| TBCB | 0.002014469 | 0.063421056 | 1095.4755 | 10.839317 | 0.15688618 | 10.736122 | 11.019858 | 10.761972 |
| TBL1X | 0.007498875 | 0.13868123 | 1869.9797 | 10.074683 | 0.11392246 | 9.947601 | 10.167652 | 10.108795 |
| TBPL1 | 0.007245979 | 0.13625583 | 1843.5154 | 10.095038 | 0.08686812 | 9.994758 | 10.147164 | 10.143192 |
| TBX2 | 0.008079063 | 0.14538123 | 1928.8063 | 10.030919 | 0.08770175 | 9.987475 | 10.131863 | 9.973419 |
| TCEAL3 | 0.002017525 | 0.063402176 | 1096.1617 | 10.836291 | 0.032835416 | 10.807611 | 10.829155 | 10.872108 |
| TCEAL4 | 0.007421975 | 0.13777314 | 1862.075 | 10.082774 | 0.075737074 | 10.064734 | 10.165903 | 10.017687 |
| TCEB1 | 0.001634029 | 0.056241073 | 1009.03345 | 10.949745 | 0.041499283 | 10.945426 | 10.993236 | 10.910575 |
| TCEB2 | 0.003424108 | 0.08780192 | 1353.2842 | 10.531964 | 0.08721989 | 10.631015 | 10.498219 | 10.466659 |
| TCF25 | 0.000768029 | 0.034968503 | 754.90967 | 11.356425 | 0.11647535 | 11.480059 | 11.340463 | 11.2487545 |
| TCFL5 | 0.007969274 | 0.14415328 | 1917.9125 | 10.038754 | 0.035945736 | 10.067258 | 10.050632 | 9.998373 |
| TCTEX1D2 | 0.004541823 | 0.104492046 | 1518.6914 | 10.372069 | 0.025586413 | 10.373034 | 10.346014 | 10.39716 |
| TDG | 0.003205425 | 0.08476296 | 1317.3492 | 10.574943 | 0.124149434 | 10.602531 | 10.439319 | 10.682976 |
| TEAD2 | 0.003067216 | 0.08249147 | 1294.5729 | 10.592877 | 0.108286 | 10.491546 | 10.7069845 | 10.580104 |
| TECR | 0.000909754 | 0.039111525 | 804.96576 | 11.275907 | 0.17276876 | 11.12105 | 11.4622555 | 11.244417 |
| TEX2 | 0.006981264 | 0.13366887 | 1815.0425 | 10.113687 | 0.06959624 | 10.108221 | 10.046984 | 10.185854 |
| TFAP2C | 0.003999423 | 0.09649235 | 1441.979 | 10.447207 | 0.057190355 | 10.417798 | 10.410706 | 10.513118 |
| TFDP1 | 0.004701879 | 0.105926625 | 1540.5195 | 10.350411 | 0.028074596 | 10.349923 | 10.378728 | 10.322585 |
| TFF1 | 2.34334E-05 | 0.003662162 | 221.07137 | 12.981354 | 0.08005664 | 13.041263 | 13.012367 | 12.8904295 |
| TFF3 | 0.000822592 | 0.03672973 | 774.9265 | 11.328334 | 0.12057139 | 11.283244 | 11.464952 | 11.236807 |
| TFRC | 0.000380037 | 0.022693632 | 582.0644 | 11.712049 | 0.123330876 | 11.6789665 | 11.848547 | 11.608633 |
| TGOLN2 | 0.007245402 | 0.13631888 | 1843.4655 | 10.095031 | 0.025213433 | 10.083839 | 10.07735 | 10.123902 |
| TH1L | 0.000637747 | 0.03160857 | 704.46466 | 11.445454 | 0.038367238 | 11.426458 | 11.489613 | 11.420289 |
| THAP11 | 0.001942296 | 0.062164206 | 1080.1621 | 10.858455 | 0.09143285 | 10.91693 | 10.905344 | 10.753089 |
| THEM2 | 0.009287485 | 0.15748778 | 2044.6046 | 9.945872 | 0.092919454 | 9.838854 | 10.006042 | 9.992721 |
| THOC7 | 4.81351E-06 | 0.001255639 | 131.93936 | 13.505096 | 0.11070721 | 13.6109 | 13.390063 | 13.514325 |
| TIGA1 | 0.001507696 | 0.053814813 | 978.32715 | 11.006417 | 0.16397762 | 10.818293 | 11.081892 | 11.119067 |
| TIMELESS | 0.004682424 | 0.10590091 | 1537.8926 | 10.351814 | 0.14279449 | 10.516282 | 10.279735 | 10.259427 |
| TIMM23 | 0.005388338 | 0.11447826 | 1629.664 | 10.270579 | 0.038904004 | 10.227488 | 10.303118 | 10.281134 |
| TIMP1 | 0.000602611 | 0.03079087 | 689.9976 | 11.476525 | 0.11831459 | 11.414931 | 11.61293 | 11.401715 |
| TINP1 | 0.000520119 | 0.027890263 | 652.88904 | 11.549777 | 0.09661066 | 11.555184 | 11.643569 | 11.450575 |
| TK1 | 0.006993313 | 0.1338257 | 1816.31 | 10.118885 | 0.09886051 | 10.0801 | 10.231257 | 10.045299 |
| TKT | 0.000596645 | 0.030530974 | 687.6462 | 11.474434 | 0.04703826 | 11.424836 | 11.480059 | 11.518407 |
| TM7SF2 | 0.005607368 | 0.11726462 | 1656.8497 | 10.243771 | 0.033407316 | 10.252544 | 10.271915 | 10.206852 |
| TM9SF2 | 0.003950481 | 0.09591182 | 1434.9354 | 10.453043 | 0.09572297 | 10.379676 | 10.561321 | 10.418131 |
| TMBIM4 | 0.000517842 | 0.027854264 | 651.7537 | 11.545513 | 0.07593104 | 11.515611 | 11.631843 | 11.489085 |
| TMBIM6 | 0.000160892 | 0.0130726 | 425.86765 | 12.137691 | 0.03048287 | 12.144529 | 12.164173 | 12.104369 |
| TMCO1 | 0.000828299 | 0.03670115 | 777.10516 | 11.318988 | 0.037462555 | 11.362165 | 11.295112 | 11.299687 |
| TMED1 | 0.005259353 | 0.11263457 | 1613.0745 | 10.282531 | 0.062365193 | 10.348126 | 10.27547 | 10.223997 |
| TMED10P | 8.58938E-05 | 0.008637682 | 342.13266 | 12.4474945 | 0.089114435 | 12.381301 | 12.412363 | 12.5488205 |
| TMED2 | 0.001180492 | 0.04601798 | 889.1319 | 11.1434965 | 0.07717885 | 11.2188 | 11.064569 | 11.147119 |
| TMED3 | 9.18891E-05 | 0.009056818 | 349.9986 | 12.418561 | 0.08579352 | 12.459974 | 12.475793 | 12.319918 |
| TMED9 | 0.001026143 | 0.042382143 | 841.3797 | 11.217952 | 0.07757133 | 11.291693 | 11.225113 | 11.137047 |
| TMEM106C | 0.001204646 | 0.04638624 | 895.9365 | 11.1345625 | 0.073822945 | 11.092857 | 11.09103 | 11.219799 |
| TMEM111 | 0.000458898 | 0.025392344 | 623.9206 | 11.6065855 | 0.07728093 | 11.560602 | 11.563346 | 11.695807 |
| TMEM123 | 0.00654753 | 0.12848416 | 1766.6539 | 10.159841 | 0.22298977 | 10.006506 | 10.4156475 | 10.05737 |
| TMEM126A | 0.006311581 | 0.12635545 | 1739.9783 | 10.174709 | 0.07998293 | 10.085627 | 10.240357 | 10.198143 |
| TMEM126B | 0.001809967 | 0.05974786 | 1050.816 | 10.895356 | 0.029408962 | 10.894002 | 10.866648 | 10.925419 |
| TMEM131 | 0.005273189 | 0.11286119 | 1614.8734 | 10.280609 | 0.057619885 | 10.338054 | 10.280957 | 10.2228155 |
| TMEM141 | 0.005120568 | 0.111241706 | 1595.4252 | 10.300709 | 0.024778046 | 10.297056 | 10.277961 | 10.327111 |
| TMEM147 | 0.002617052 | 0.07510008 | 1215.002 | 10.689248 | 0.04511197 | 10.669669 | 10.740843 | 10.657235 |
| TMEM14A | 0.00558575 | 0.11716566 | 1654.2092 | 10.247264 | 0.053584896 | 10.185854 | 10.271418 | 10.284521 |
| TMEM14B | 0.000246613 | 0.017180722 | 498.36908 | 11.912457 | 0.15023555 | 11.8032465 | 12.083791 | 11.850334 |
| TMEM14C | 0.000417363 | 0.02381579 | 602.4102 | 11.654937 | 0.03720744 | 11.630537 | 11.636512 | 11.697762 |
| TMEM14D | 0.000704012 | 0.033367485 | 730.8079 | 11.399953 | 0.1342273 | 11.359631 | 11.54972 | 11.290509 |
| TMEM160 | 0.005587681 | 0.117135346 | 1654.4897 | 10.249831 | 0.10287439 | 10.368465 | 10.195754 | 10.185272 |
| TMEM183B | 0.000607857 | 0.030967694 | 692.4558 | 11.473808 | 0.12248844 | 11.388632 | 11.614182 | 11.41861 |
| TMEM203 | 0.005103044 | 0.111069635 | 1593.291 | 10.300812 | 0.16307697 | 10.1378355 | 10.30061 | 10.463989 |
| TMEM205 | 0.000729406 | 0.034243573 | 740.29156 | 11.3825655 | 0.07022683 | 11.378807 | 11.454597 | 11.314295 |
| TMEM216 | 0.006866577 | 0.13234945 | 1802.451 | 10.123549 | 0.15442584 | 9.945989 | 10.198143 | 10.226519 |
| TMEM30B | 0.00589981 | 0.121404506 | 1691.5284 | 10.215833 | 0.13599879 | 10.121048 | 10.154793 | 10.371657 |
| TMEM4 | 0.005038422 | 0.1102858 | 1585.0885 | 10.307906 | 0.098205715 | 10.317793 | 10.205131 | 10.400794 |
| TMEM43 | 0.007838704 | 0.14238533 | 1904.7961 | 10.045258 | 0.0767836 | 10.111923 | 9.961304 | 10.062546 |
| TMEM49 | 9.30132E-05 | 0.009141643 | 351.40164 | 12.413074 | 0.16189209 | 12.569252 | 12.423951 | 12.2460165 |
| TMEM5 | 0.006503171 | 0.12812096 | 1761.7769 | 10.161044 | 0.06651812 | 10.164289 | 10.22588 | 10.092962 |
| TMEM59 | 0.000441287 | 0.024773464 | 614.86975 | 11.62597 | 0.07742108 | 11.642035 | 11.694099 | 11.541778 |
| TMEM60 | 0.003123566 | 0.08336077 | 1303.8191 | 10.582246 | 0.027536554 | 10.557186 | 10.611725 | 10.577827 |
| TMEM66 | 0.000542659 | 0.028656011 | 664.0776 | 11.520226 | 0.08233374 | 11.58375 | 11.427207 | 11.54972 |
| TMEM83 | 0.006672422 | 0.12997922 | 1780.9879 | 10.146001 | 0.08834957 | 10.187391 | 10.206058 | 10.044555 |
| TMEM85 | 0.002450049 | 0.07179223 | 1183.2606 | 10.731792 | 0.13514934 | 10.575783 | 10.806457 | 10.813137 |
| TMEM87A | 0.001477057 | 0.052993797 | 970.4151 | 11.013745 | 0.06472081 | 10.968862 | 11.087936 | 10.984442 |
| TMEM97 | 0.006220096 | 0.12553811 | 1729.3074 | 10.186947 | 0.009914312 | 10.196954 | 10.186758 | 10.177128 |
| TMSB10 | 2.68058E-06 | 0.000853211 | 106.80025 | 13.655635 | 0.046378132 | 13.645062 | 13.615455 | 13.706386 |
| TMSB4X | 0.002315617 | 0.069436476 | 1157.0253 | 10.762367 | 0.07466988 | 10.677694 | 10.81879 | 10.790618 |
| TMSL3 | 0.000133568 | 0.011672544 | 399.63232 | 12.227725 | 0.1601708 | 12.102364 | 12.408168 | 12.172641 |
| TNFRSF12A | 0.004325734 | 0.1008582 | 1488.4187 | 10.4002905 | 0.11844468 | 10.452691 | 10.264684 | 10.483497 |
| TNFRSF21 | 0.005946273 | 0.122071005 | 1697.4337 | 10.2126 | 0.07431679 | 10.167652 | 10.29838 | 10.171765 |
| TNPO2 | 0.003684182 | 0.09189001 | 1394.1865 | 10.494145 | 0.064118356 | 10.544806 | 10.5155735 | 10.422057 |
| TOB1 | 0.001519802 | 0.053969294 | 981.3629 | 11.000779 | 0.111620516 | 10.876636 | 11.0328455 | 11.092857 |
| TOMM20 | 0.002724218 | 0.07702853 | 1234.3159 | 10.666098 | 0.044629667 | 10.711855 | 10.622688 | 10.66375 |
| TOMM22 | 0.006576094 | 0.12875338 | 1769.9316 | 10.155215 | 0.08798703 | 10.256047 | 10.09401 | 10.115588 |
| TOMM40 | 0.000400559 | 0.02323913 | 593.1768 | 11.670484 | 0.11142028 | 11.750175 | 11.543165 | 11.718109 |
| TOMM5 | 0.00228933 | 0.069066085 | 1151.9995 | 10.762593 | 0.17375945 | 10.655056 | 10.669669 | 10.963057 |
| TOMM6 | 0.000387387 | 0.022818336 | 585.9751 | 11.692636 | 0.063847356 | 11.710464 | 11.745675 | 11.62177 |
| TOMM7 | 3.72687E-05 | 0.004992278 | 257.63245 | 12.814364 | 0.016366486 | 12.83051 | 12.797786 | 12.814797 |
| TOMM70A | 0.004887214 | 0.10820485 | 1565.3116 | 10.330955 | 0.034214865 | 10.370338 | 10.308557 | 10.313968 |
| TOP2A | 0.001919583 | 0.06189405 | 1075.1766 | 10.865479 | 0.065397866 | 10.791383 | 10.8899145 | 10.915142 |
| TOP2B | 0.002055946 | 0.06431831 | 1104.305 | 10.826823 | 0.08868294 | 10.737781 | 10.915142 | 10.827543 |
| TP53I13 | 0.002045858 | 0.06411834 | 1102.2223 | 10.833333 | 0.023271801 | 10.859422 | 10.82587 | 10.81471 |
| TPD52L1 | 0.009146308 | 0.15631625 | 2031.9008 | 9.9518 | 0.018402409 | 9.970055 | 9.933253 | 9.952092 |
| TPD52L2 | 0.005517352 | 0.11636414 | 1645.7789 | 10.253295 | 0.030309932 | 10.286382 | 10.246632 | 10.226871 |
| TPI1 | 0.000120972 | 0.010958225 | 385.83786 | 12.265556 | 0.08267655 | 12.334259 | 12.1737995 | 12.2886095 |
| TPM4 | 0.009117283 | 0.15589699 | 2029.1436 | 9.953685 | 0.107723214 | 10.063471 | 9.949434 | 9.84815 |
| TPT1 | 1.67176E-06 | 0.000617021 | 97 | 13.709373 | 0.007598201 | 13.704502 | 13.705491 | 13.718129 |
| TRAM1 | 0.000453652 | 0.025222756 | 621.4249 | 11.606842 | 0.100972846 | 11.498708 | 11.6986685 | 11.623149 |
| TRAPPC2L | 0.000689226 | 0.03293664 | 725.0283 | 11.40183 | 0.099396996 | 11.49177 | 11.41861 | 11.295112 |
| TRAPPC3 | 0.008810543 | 0.15291296 | 1999.8573 | 9.975487 | 0.07004284 | 10.056347 | 9.9365225 | 9.933589 |
| TRAPPC4 | 0.002498472 | 0.07284202 | 1192.5558 | 10.716709 | 0.023909755 | 10.700739 | 10.705191 | 10.744198 |
| TRIAP1 | 0.004502652 | 0.10386635 | 1513.5665 | 10.373048 | 0.081230015 | 10.292901 | 10.370924 | 10.455319 |
| TRIB3 | 0.009193751 | 0.15651031 | 2036.0435 | 9.953304 | 0.085778676 | 10.042729 | 9.871709 | 9.945475 |
| TRIM8 | 0.007144752 | 0.13537957 | 1832.4357 | 10.105233 | 0.03235714 | 10.092428 | 10.142034 | 10.081239 |
| TRIP6 | 0.005351848 | 0.114052214 | 1625.1304 | 10.271754 | 0.054034796 | 10.269003 | 10.327111 | 10.219147 |
| TRMT112 | 6.48239E-05 | 0.007231511 | 311.0106 | 12.587102 | 0.07103623 | 12.566641 | 12.528543 | 12.666124 |
| TRMT12 | 0.005172912 | 0.11174907 | 1602.2133 | 10.295909 | 0.090349786 | 10.327111 | 10.194093 | 10.366521 |
| TSEN34 | 0.000663458 | 0.032238096 | 714.78094 | 11.426442 | 0.040833943 | 11.440481 | 11.380441 | 11.458405 |
| TSG101 | 0.004589497 | 0.10517041 | 1525.1584 | 10.364749 | 0.07214243 | 10.285086 | 10.425673 | 10.383488 |
| TSPAN13 | 0.000714936 | 0.03374694 | 734.98224 | 11.391126 | 0.095346026 | 11.299644 | 11.383817 | 11.489916 |
| TSPYL1 | 0.007515767 | 0.13884558 | 1871.7358 | 10.070218 | 0.09753724 | 10.144297 | 9.959709 | 10.106648 |
| TSR2 | 0.004671096 | 0.105782636 | 1536.4 | 10.355213 | 0.16438906 | 10.447533 | 10.452691 | 10.165417 |
| TST | 0.000172249 | 0.013643836 | 436.3844 | 12.101845 | 0.055235945 | 12.160319 | 12.0505495 | 12.094666 |
| TTC4 | 0.00954583 | 0.16053466 | 2067.9336 | 9.929471 | 0.08263444 | 9.867544 | 10.023301 | 9.897567 |
| TTF2 | 0.004450337 | 0.103002004 | 1506.1132 | 10.380828 | 0.14519702 | 10.342939 | 10.541213 | 10.258331 |
| TUBA1A | 3.67787E-05 | 0.004945736 | 256.28903 | 12.814601 | 0.028019672 | 12.782789 | 12.825391 | 12.83562 |
| TUBA1B | 3.17058E-07 | 0.000164179 | 63.60326 | 13.892667 | 0.12608653 | 13.946452 | 13.748608 | 13.982943 |
| TUBA1C | 1.1587E-05 | 0.002323699 | 176.3327 | 13.233747 | 0.015426899 | 13.215954 | 13.241899 | 13.243387 |
| TUBB | 0.000585202 | 0.030123146 | 682.26373 | 11.485324 | 0.07220681 | 11.509368 | 11.542439 | 11.404161 |
| TUBB2C | 0.000537903 | 0.028491603 | 661.7168 | 11.5267515 | 0.07770895 | 11.45205 | 11.607154 | 11.521048 |
| TUBB3 | 0.00187923 | 0.06116135 | 1066.3704 | 10.875846 | 0.057657503 | 10.897068 | 10.810585 | 10.919885 |
| TUBD1 | 0.003063181 | 0.08257498 | 1293.8298 | 10.600389 | 0.13783005 | 10.448742 | 10.634382 | 10.71804 |
| TUFM | 0.002637084 | 0.075425394 | 1218.8282 | 10.68488 | 0.032768346 | 10.704517 | 10.647053 | 10.7030735 |
| TUFT1 | 0.004400559 | 0.10219076 | 1498.9738 | 10.391813 | 0.058995754 | 10.416271 | 10.434647 | 10.324522 |
| TUG1 | 0.000313455 | 0.020101663 | 543.42084 | 11.8030815 | 0.15917973 | 11.839849 | 11.94066 | 11.628736 |
| TXLNA | 0.004214562 | 0.0996728 | 1472.9769 | 10.414889 | 0.12047025 | 10.53842 | 10.408514 | 10.297732 |
| TXN | 5.13057E-06 | 0.001280576 | 137.14453 | 13.454346 | 0.097328044 | 13.490345 | 13.528546 | 13.344147 |
| TXNDC12 | 0.009103447 | 0.15573718 | 2027.7992 | 9.955819 | 0.038929142 | 9.989535 | 9.964709 | 9.913215 |
| TXNDC17 | 9.43967E-05 | 0.00917367 | 353.57858 | 12.404388 | 0.13480856 | 12.264769 | 12.414593 | 12.533806 |
| TXNIP | 0.001412665 | 0.05148214 | 953.44214 | 11.04243 | 0.13005055 | 11.033761 | 11.176598 | 10.91693 |
| TYMS | 0.003968352 | 0.09621104 | 1437.3984 | 10.449247 | 0.095788546 | 10.502487 | 10.338666 | 10.50659 |
| UBA1 | 0.007231769 | 0.1362101 | 1841.9426 | 10.099831 | 0.051341478 | 10.043371 | 10.143719 | 10.112403 |
| UBA52 | 0.001401309 | 0.051175788 | 950.5592 | 11.047175 | 0.031029612 | 11.051482 | 11.075827 | 11.014217 |
| UBAC1 | 0.005257278 | 0.11265966 | 1612.8036 | 10.281373 | 0.17476518 | 10.139346 | 10.476541 | 10.228234 |
| UBB | 2.24823E-06 | 0.000795918 | 101.65254 | 13.681584 | 0.019572573 | 13.685513 | 13.660346 | 13.698895 |
| UBC | 0.000379086 | 0.022754325 | 581.694 | 11.702209 | 0.0679173 | 11.729241 | 11.624938 | 11.752448 |
| UBE2E1 | 0.008019139 | 0.14475338 | 1922.8041 | 10.036334 | 0.054464847 | 10.0578785 | 10.076732 | 9.974393 |
| UBE2F | 0.008344498 | 0.14808388 | 1954.5432 | 10.009697 | 0.08400514 | 9.951664 | 9.971402 | 10.106027 |
| UBE2M | 0.00100882 | 0.041966427 | 836.2842 | 11.225842 | 0.101127595 | 11.33814 | 11.197426 | 11.141965 |
| UBE2N | 0.007985761 | 0.14430104 | 1919.5796 | 10.036164 | 0.062085688 | 9.974864 | 10.034622 | 10.099007 |
| UBE2Q2 | 0.003333545 | 0.08637341 | 1338.805 | 10.5457735 | 0.106706016 | 10.627742 | 10.584459 | 10.425121 |
| UBE2T | 0.001942296 | 0.062164206 | 1080.1593 | 10.860031 | 0.031123767 | 10.870688 | 10.824978 | 10.884425 |
| UBE4A | 0.00937358 | 0.15863757 | 2052.55 | 9.937832 | 0.017718114 | 9.957693 | 9.92365 | 9.932152 |
| UBL3 | 0.007972992 | 0.14414538 | 1918.2544 | 10.034628 | 0.11653427 | 10.132892 | 10.06511 | 9.905882 |
| UBP1 | 0.003204935 | 0.084814645 | 1317.2322 | 10.573186 | 0.08363806 | 10.603638 | 10.478588 | 10.637331 |
| UBQLN4 | 0.009582176 | 0.16098984 | 2071.2676 | 9.92553 | 0.034604438 | 9.938022 | 9.8864155 | 9.952156 |
| UBXN1 | 0.003228887 | 0.085059226 | 1321.3403 | 10.5655155 | 0.0810592 | 10.566113 | 10.646275 | 10.484159 |
| UBXN4 | 0.001886868 | 0.061294943 | 1068.0774 | 10.874148 | 0.08346239 | 10.780636 | 10.9410925 | 10.900716 |
| UCKL1 | 0.006239696 | 0.12549566 | 1731.5658 | 10.183188 | 0.02237394 | 10.15916 | 10.203423 | 10.186981 |
| UFC1 | 0.006187266 | 0.12509382 | 1725.6393 | 10.187228 | 0.10078459 | 10.087754 | 10.184657 | 10.289274 |
| UGDH | 0.000161382 | 0.013081776 | 426.3976 | 12.131213 | 0.10595248 | 12.030637 | 12.121172 | 12.241827 |
| UIMC1 | 0.00831406 | 0.14784624 | 1951.7388 | 10.012288 | 0.059256274 | 10.0501375 | 9.943999 | 10.042729 |
| UNC50 | 0.005111086 | 0.11117492 | 1594.2806 | 10.301309 | 0.090117075 | 10.239754 | 10.259427 | 10.404745 |
| UNC84B | 0.008493342 | 0.14965363 | 1969.1277 | 10.002265 | 0.041594576 | 10.020074 | 10.03199 | 9.954731 |
| UQCRC1 | 0.008750995 | 0.1524897 | 1993.9067 | 9.981983 | 0.10093179 | 9.868944 | 10.063075 | 10.013932 |
| UQCRC2 | 0.008288753 | 0.1474718 | 1949.1833 | 10.011712 | 0.12401015 | 10.106027 | 10.057868 | 9.8712435 |
| UQCRFS1 | 0.000122759 | 0.011005168 | 387.86884 | 12.265899 | 0.048772298 | 12.235666 | 12.322164 | 12.239865 |
| UQCRH | 0.001009512 | 0.04194491 | 836.5054 | 11.223788 | 0.09754816 | 11.221444 | 11.127434 | 11.322488 |
| UQCRHL | 3.00052E-05 | 0.00437395 | 239.76505 | 12.895386 | 0.089023896 | 12.945323 | 12.792603 | 12.948229 |
| UQCRQ | 1.82741E-05 | 0.00307767 | 203.90172 | 13.0747595 | 0.048308313 | 13.019023 | 13.104576 | 13.100678 |
| URM1 | 0.002548452 | 0.07380301 | 1201.8179 | 10.706146 | 0.057985064 | 10.649393 | 10.765289 | 10.703756 |
| UROD | 0.007930795 | 0.14360699 | 1914.1499 | 10.045749 | 0.042524196 | 10.007072 | 10.091287 | 10.038889 |
| UROS | 0.006507321 | 0.12812996 | 1762.2739 | 10.1546545 | 0.14024659 | 10.234205 | 9.992721 | 10.23704 |
| USP3 | 0.006415346 | 0.12747651 | 1751.9332 | 10.167862 | 0.072566256 | 10.232475 | 10.089353 | 10.181759 |
| VAMP7 | 0.005142503 | 0.1114391 | 1598.3466 | 10.297343 | 0.09760725 | 10.351803 | 10.35557 | 10.184657 |
| VAMP8 | 0.000341414 | 0.021227598 | 560.23737 | 11.756203 | 0.18785185 | 11.973013 | 11.65356 | 11.642035 |
| VBP1 | 0.001721912 | 0.058339845 | 1030.2102 | 10.924823 | 0.06708742 | 10.878513 | 11.001758 | 10.894197 |
| VCP | 0.004225774 | 0.099734016 | 1474.4313 | 10.413605 | 0.03335289 | 10.405924 | 10.450128 | 10.384762 |
| VDAC1 | 0.000353779 | 0.021723894 | 567.26746 | 11.743173 | 0.1993412 | 11.63908 | 11.973013 | 11.617427 |
| VDAC3 | 0.000559347 | 0.029225904 | 671.15845 | 11.505044 | 0.10468001 | 11.442639 | 11.446596 | 11.625896 |
| VEZF1 | 0.003509512 | 0.08887518 | 1367.004 | 10.516391 | 0.117507786 | 10.460567 | 10.651404 | 10.4372 |
| VIL2 | 0.001303741 | 0.049005415 | 923.54663 | 11.084981 | 0.19625783 | 11.080908 | 11.283244 | 10.890792 |
| VKORC1 | 0.00411319 | 0.09821267 | 1458.4038 | 10.427252 | 0.030120557 | 10.39605 | 10.456161 | 10.429544 |
| VPS26A | 0.002871592 | 0.07951077 | 1260.7299 | 10.637572 | 0.12147294 | 10.497641 | 10.715902 | 10.699175 |
| VPS29 | 0.000533003 | 0.028275229 | 659.2003 | 11.533778 | 0.085665196 | 11.631843 | 11.47352 | 11.495971 |
| VPS35 | 0.001848735 | 0.06068117 | 1059.805 | 10.885628 | 0.03296183 | 10.895381 | 10.912613 | 10.84889 |
| VPS37C | 0.001586903 | 0.055332664 | 997.7031 | 10.975045 | 0.1482739 | 10.829961 | 10.968862 | 11.126315 |
| VTI1B | 0.007825532 | 0.14229508 | 1903.488 | 10.049766 | 0.086987816 | 10.013932 | 9.986419 | 10.148947 |
| WASL | 0.004658241 | 0.10597574 | 1534.6177 | 10.356175 | 0.017211972 | 10.336823 | 10.369773 | 10.36193 |
| WBP2 | 0.000636565 | 0.031685796 | 703.92035 | 11.442283 | 0.08770208 | 11.34513 | 11.466108 | 11.515611 |
| WBSCR22 | 0.006313455 | 0.12632006 | 1740.1836 | 10.17217 | 0.063862935 | 10.098496 | 10.206231 | 10.211781 |
| WDR18 | 0.006150256 | 0.12470894 | 1721.2738 | 10.189605 | 0.07957399 | 10.179462 | 10.115588 | 10.273764 |
| WDR54 | 0.002860466 | 0.079329334 | 1258.6626 | 10.631015 | 0.045281295 | 10.638819 | 10.671886 | 10.582338 |
| WDR6 | 0.003246123 | 0.085189864 | 1324.0355 | 10.564415 | 0.032076295 | 10.53443 | 10.560578 | 10.598237 |
| WDR61 | 0.001044532 | 0.04288639 | 847.0934 | 11.21103 | 0.0751156 | 11.136291 | 11.210284 | 11.286516 |
| WDR68 | 0.005856344 | 0.120724894 | 1686.2224 | 10.220828 | 0.06406817 | 10.28572 | 10.219147 | 10.157617 |
| WDR75 | 0.003786448 | 0.09310205 | 1409.9233 | 10.480502 | 0.061254773 | 10.4168625 | 10.485588 | 10.539055 |
| WISP2 | 0.00984346 | 0.16355795 | 2094.8235 | 9.906119 | 0.088640876 | 9.930774 | 9.807761 | 9.979823 |
| WRB | 0.009051479 | 0.15523085 | 2022.8247 | 9.957054 | 0.024962083 | 9.965076 | 9.929068 | 9.977019 |
| XBP1 | 1.60258E-05 | 0.002808081 | 195.41223 | 13.112912 | 0.114661984 | 13.034109 | 13.244455 | 13.060175 |
| XPNPEP3 | 0.004464259 | 0.10318654 | 1508.187 | 10.379776 | 0.13682991 | 10.226123 | 10.424738 | 10.488468 |
| XPO1 | 0.002358189 | 0.070167236 | 1165.5826 | 10.751609 | 0.024556695 | 10.725054 | 10.756275 | 10.773498 |
| XRCC6 | 0.005712198 | 0.11867006 | 1669.2073 | 10.232724 | 0.04340253 | 10.282586 | 10.2121725 | 10.203416 |
| XYLT2 | 0.00727094 | 0.13650325 | 1846.043 | 10.097442 | 0.067382336 | 10.029537 | 10.164289 | 10.098496 |
| YARS | 0.005820257 | 0.120266825 | 1682.0654 | 10.227456 | 0.116036475 | 10.24457 | 10.333985 | 10.103813 |
| YBX1 | 0.000185046 | 0.014298441 | 448.65604 | 12.070241 | 0.015001526 | 12.05412 | 12.072812 | 12.083791 |
| YES1 | 0.00979371 | 0.16343579 | 2090.4495 | 9.910809 | 0.08272529 | 9.952156 | 9.815561 | 9.964709 |
| YIF1A | 0.003340924 | 0.08630677 | 1339.9025 | 10.548482 | 0.06477315 | 10.608872 | 10.480073 | 10.556502 |
| YIPF3 | 0.006105119 | 0.124156505 | 1715.7886 | 10.198823 | 0.12866624 | 10.304935 | 10.235824 | 10.055711 |
| YTHDF1 | 0.002781634 | 0.07814251 | 1244.4054 | 10.651283 | 0.059755974 | 10.61469 | 10.720241 | 10.61892 |
| YWHAB | 0.005004467 | 0.109958835 | 1580.6599 | 10.314233 | 0.002707598 | 10.311178 | 10.316336 | 10.3151865 |
| YWHAG | 0.007100594 | 0.13469 | 1827.7603 | 10.10357 | 0.11000849 | 10.15235 | 9.977607 | 10.180755 |
| YWHAH | 0.000167896 | 0.013390805 | 432.5316 | 12.1120615 | 0.044576347 | 12.122804 | 12.063096 | 12.150286 |
| YWHAQ | 1.84182E-05 | 0.003086957 | 204.65466 | 13.063275 | 0.06716942 | 12.998754 | 13.132812 | 13.058264 |
| YWHAZ | 0.006862512 | 0.13241825 | 1802.0332 | 10.12934 | 0.08151526 | 10.149419 | 10.198939 | 10.039661 |
| YY1 | 0.000889405 | 0.038523097 | 797.8988 | 11.289176 | 0.022289198 | 11.302855 | 11.301216 | 11.263455 |
| ZBTB33 | 0.002855537 | 0.079256 | 1257.7098 | 10.63775 | 0.08189277 | 10.561321 | 10.724187 | 10.627742 |
| ZBTB42 | 0.009323888 | 0.15795068 | 2047.9308 | 9.941987 | 0.07071899 | 10.019569 | 9.925261 | 9.88113 |
| ZC3H15 | 0.009062144 | 0.15526025 | 2023.7745 | 9.959378 | 0.05570774 | 9.900133 | 9.967301 | 10.0107 |
| ZDHHC7 | 0.003605522 | 0.09051375 | 1382.3464 | 10.5042715 | 0.061814267 | 10.532587 | 10.433372 | 10.546856 |
| ZDHHC8 | 0.006331441 | 0.12653399 | 1742.3102 | 10.174994 | 0.08067134 | 10.265897 | 10.111923 | 10.147164 |
| ZFAND2A | 0.005056148 | 0.11053434 | 1587.3014 | 10.309956 | 0.004509999 | 10.307352 | 10.307352 | 10.315164 |
| ZFAND5 | 0.001501239 | 0.053639546 | 976.60645 | 11.005351 | 0.14113854 | 10.981694 | 10.877536 | 11.156823 |
| ZFP36L1 | 0.006633078 | 0.12943082 | 1776.4672 | 10.142532 | 0.15191117 | 10.128926 | 9.997882 | 10.300789 |
| ZFYVE20 | 0.005401481 | 0.114617124 | 1631.2382 | 10.264141 | 0.14506592 | 10.1614 | 10.430086 | 10.200937 |
| ZFYVE21 | 0.002668963 | 0.07596144 | 1224.4215 | 10.674281 | 0.13828318 | 10.651404 | 10.8225765 | 10.548863 |
| ZMAT2 | 0.00669698 | 0.13023823 | 1783.6594 | 10.144088 | 0.15883617 | 10.229942 | 10.241521 | 9.960802 |
| ZMAT3 | 0.002930363 | 0.07986332 | 1271.0482 | 10.624101 | 0.058919255 | 10.589617 | 10.590553 | 10.692133 |
| ZMIZ1 | 0.007027008 | 0.13387974 | 1819.9407 | 10.113942 | 0.06048319 | 10.111424 | 10.175645 | 10.054757 |
| ZMPSTE24 | 0.001622355 | 0.056117646 | 1006.3094 | 10.960134 | 0.010132587 | 10.969664 | 10.949491 | 10.961248 |
| ZNF207 | 0.005005304 | 0.1099076 | 1580.7511 | 10.311873 | 0.080045 | 10.295379 | 10.39888 | 10.24136 |
| ZNF217 | 0.000832968 | 0.03681401 | 778.6614 | 11.321422 | 0.15441974 | 11.1534195 | 11.3536825 | 11.457162 |
| ZNF364 | 0.005944457 | 0.12210598 | 1697.2053 | 10.215096 | 0.13796438 | 10.206231 | 10.357279 | 10.081778 |
| ZNF428 | 0.000339079 | 0.021120287 | 558.9689 | 11.752792 | 0.009257122 | 11.74271 | 11.754756 | 11.760909 |
| ZNF430 | 0.008463855 | 0.14928572 | 1966.3353 | 9.998828 | 0.15584698 | 10.151562 | 9.840044 | 10.004879 |
| ZNF486 | 0.000374186 | 0.022538194 | 578.9861 | 11.711768 | 0.13645059 | 11.599071 | 11.863475 | 11.672757 |
| ZNF511 | 0.004394103 | 0.10210918 | 1498.0709 | 10.391262 | 0.11849554 | 10.346757 | 10.525567 | 10.301463 |
| ZNF593 | 0.004242434 | 0.09992328 | 1476.7642 | 10.414188 | 0.23100717 | 10.380896 | 10.201633 | 10.660035 |
| ZNF622 | 0.003752436 | 0.09272578 | 1404.737 | 10.482196 | 0.09414107 | 10.546206 | 10.374102 | 10.526279 |
| ZNF674 | 4.03528E-07 | 0.000186667 | 69.23243 | 13.88638 | 0.05272365 | 13.834939 | 13.940299 | 13.883904 |
| ZNF738 | 0.002846342 | 0.07919086 | 1255.9113 | 10.643909 | 0.07905852 | 10.667472 | 10.555749 | 10.7085085 |
| ZNHIT1 | 0.001696691 | 0.057710785 | 1024.048 | 10.9354925 | 0.0602679 | 10.977846 | 10.866494 | 10.962135 |
