# Supplementary material for: Cellular Mechanisms Triggered by the Cotreatment of Resveratrol and Doxorubicin in Breast Cancer: A Translational In Vitro–In Silico Model
Source: Oxid Med Cell Longev. 2020 Nov 1;2020:5432651. doi: 10.1155/2020/5432651 (PMC7654215; doi:10.1155/2020/5432651)
Supplement: Supplementary 7 — Supplementary Table 2 CentiScape analysis of the intersection CP-PPI network. (A) Degree and betweenness centrality scores of each node. (B) Hub and bottleneck nodes. [file 5432651.f7.docx]

**Supplementary table 2.** CentiScape Analysis. (A) Degree and betweeness centrality scores of each node from Intersection CP-PPI network . (B) Hub and bottlenecks nodes.

**A**

| ID | **CentiScape Betweeness** | **CentiScape Degree** |
| --- | --- | --- |
| ACP5 | 0 | 3 |
| ACTG1 | 115.8619321 | 25 |
| AGR2 | 7.664385374 | 9 |
| ANXA2 | 111.9205367 | 23 |
| ANXA5 | 497.4321423 | 52 |
| APEX1 | 39.16279096 | 16 |
| APOB | 54.504922 | 13 |
| AREG | 33.18912693 | 13 |
| ARG1 | 92.99097838 | 18 |
| AURKA | 197.5673902 | 28 |
| BMI1 | 149.6651434 | 32 |
| BMP2 | 400.6342761 | 16 |
| BNIP3 | 12.19875614 | 9 |
| C10orf54 | 0 | 2 |
| CALR | 39.86947591 | 16 |
| CARM1 | 94.97273514 | 21 |
| CCL22 | 2.694570245 | 12 |
| CCNA2 | 177.4189899 | 30 |
| CCND1 | 1152.997347 | 70 |
| CCND3 | 16.13443559 | 15 |
| CD19 | 217.1666919 | 25 |
| CD24 | 69.60733092 | 19 |
| CD33 | 36.18110195 | 15 |
| CD63 | 12.6951042 | 12 |
| CDC20 | 21.37038299 | 14 |
| CDH1 | 1665.744706 | 66 |
| CDH5 | 19.62233952 | 14 |
| CDK4 | 303.8592736 | 39 |
| CDKN1B | 37.07168053 | 23 |
| CDKN3 | 85.97612027 | 21 |
| CEBPB | 245.5712128 | 16 |
| CFL1 | 47.68160416 | 17 |
| CLDN3 | 15.29334249 | 12 |
| CLDN7 | 0.153846154 | 9 |
| CSF3 | 105.8418527 | 24 |
| CXCL9 | 23.39908599 | 16 |
| CYP17A1 | 0 | 2 |
| CYP1B1 | 19.32775729 | 9 |
| DLL4 | 12.86128066 | 8 |
| DNMT1 | 220.8956065 | 35 |
| EBF1 | 0.533333333 | 3 |
| EIF3A | 8.553388278 | 6 |
| EIF3H | 0.4 | 4 |
| EPCAM | 192.7155407 | 23 |
| EPO | 30.69026843 | 14 |
| ESR1 | 1794.250246 | 67 |
| FADD | 17.38899802 | 14 |
| FASN | 63.72943817 | 13 |
| FLT1 | 144.63218 | 23 |
| FOXA1 | 298.5511272 | 25 |
| FOXC1 | 31.23849231 | 11 |
| FSCN1 | 0 | 4 |
| GAPDH | 6219.377231 | 112 |
| GATA3 | 484.5721611 | 35 |
| GDI2 | 3.134829985 | 5 |
| GGCT | 0 | 1 |
| GLI2 | 25.27299994 | 12 |
| GZMB | 69.53526523 | 16 |
| H3F3A | 55.47429451 | 22 |
| H3F3B | 20.13268 | 17 |
| HDAC1 | 478.9968222 | 45 |
| HEATR6 | 0 | 1 |
| HIST1H4C | 25.89163291 | 24 |
| HIST2H2AA | 14.00068831 | 18 |
| HIST2H2AA3 | 14.00068831 | 18 |
| HIST2H2AC | 92.52237369 | 25 |
| HIST2H2BE | 244.6629207 | 32 |
| HNF4G | 0 | 2 |
| HPRT1 | 23.01453301 | 12 |
| HSP90AA1 | 899.141619 | 56 |
| HSPB1 | 75.75441999 | 18 |
| HSPG2 | 3.508823529 | 6 |
| IDH2 | 36.39773289 | 11 |
| IDO1 | 318.0474976 | 19 |
| IGFBP5 | 5.682221111 | 7 |
| IL18 | 41.33440755 | 18 |
| IL1A | 34.58098493 | 12 |
| IRS1 | 84.27980193 | 23 |
| KDM1A | 161.5970348 | 28 |
| KDM5B | 24.60385469 | 16 |
| KRT19 | 71.85318829 | 19 |
| KRT5 | 19.51415337 | 14 |
| KRT8 | 78.76202197 | 21 |
| LDHA | 221.1817902 | 17 |
| LGALS3 | 51.0391971 | 18 |
| LIN28A | 304.1818182 | 8 |
| LOXL2 | 0 | 2 |
| LYVE1 | 8.058808345 | 7 |
| MAP2K1 | 311.472058 | 37 |
| MAP3K1 | 159.4994846 | 12 |
| MAPK3 | 696.9443768 | 57 |
| MCAM | 42.62430789 | 15 |
| MGMT | 73.55452768 | 21 |
| MMP8 | 2.840836649 | 6 |
| MRC1 | 39.31048156 | 15 |
| MRPS30 | 0.774399314 | 2 |
| MSH6 | 12.86667989 | 11 |
| MTDH | 1.157936508 | 3 |
| NFE2L2 | 12.18378623 | 12 |
| NFKBIA | 73.92583581 | 22 |
| NME1 | 9.887625241 | 12 |
| NME1-NME2 | 23.21239201 | 10 |
| NQO1 | 10.92134515 | 12 |
| NRAS | 88.2455833 | 22 |
| OSR1 | 0 | 1 |
| PARP1 | 243.5500661 | 34 |
| PDCD1LG2 | 54.94858864 | 11 |
| PDGFRB | 100.9294815 | 26 |
| PHGDH | 9.45203524 | 7 |
| POU5F1B | 0 | 1 |
| PPM1D | 6.765857832 | 5 |
| PSMD4 | 37.48945427 | 13 |
| PTPN1 | 30.70732583 | 14 |
| PTPN11 | 259.6431584 | 27 |
| PTTG1 | 2.978370836 | 9 |
| RAD51C | 16.60027607 | 11 |
| RHOA | 221.8554933 | 31 |
| RHOC | 42.60118027 | 13 |
| ROS1 | 0.555555556 | 5 |
| RPS3A | 87.25745493 | 11 |
| RPS6 | 213.5777748 | 15 |
| RPS6KB1 | 411.6660202 | 31 |
| SDHB | 0 | 3 |
| SLC39A6 | 0.902136752 | 6 |
| SMARCA4 | 377.0777492 | 34 |
| SNAI1 | 478.6696864 | 37 |
| SOX4 | 1.557243381 | 7 |
| SQSTM1 | 40.60474377 | 18 |
| SRSF1 | 11.69560747 | 8 |
| STAT1 | 474.0392499 | 39 |
| SUZ12 | 17.09373301 | 17 |
| TBL1X | 0 | 4 |
| TFAP2C | 7.876004144 | 7 |
| TFF1 | 39.84420274 | 14 |
| TFRC | 65.46635411 | 17 |
| TIMP1 | 193.6894915 | 24 |
| TLR1 | 39.48359879 | 19 |
| TLR2 | 236.5195706 | 33 |
| TLR7 | 81.8556399 | 23 |
| TOP2A | 89.85700481 | 25 |
| TSG101 | 7.122633127 | 9 |
| TYMS | 95.64279416 | 17 |
| UIMC1 | 18.88812198 | 11 |
| VCAN | 33.52608946 | 9 |
| VWF | 129.2843575 | 23 |
| XBP1 | 51.84567972 | 15 |
| XRCC6 | 86.63154509 | 23 |
| YBX1 | 41.85592589 | 14 |
| YWHAZ | 37.19131125 | 18 |
| ZEB1 | 174.8820284 | 29 |
| ZMIZ1 | 0.898025346 | 2 |
| ZNF217 | 5.010113082 | 10 |
| doxorubicin | 252.2991472 | 31 |
| resveratrol | 330.698698 | 33 |
| **Mean** | **168** | **18.54545455** |

**B**

| **ID** | **CentiScape Betweeness** | **CentiScape Degree** |
| --- | --- | --- |
| ANXA5 | 497.4321423 | 52 |
| AURKA | 197.5673902 | 28 |
| CCNA2 | 177.4189899 | 30 |
| CCND1 | 1152.997347 | 70 |
| CD19 | 217.1666919 | 25 |
| CDH1 | 1665.744706 | 66 |
| CDK4 | 303.8592736 | 39 |
| DNMT1 | 220.8956065 | 35 |
| EPCAM | 192.7155407 | 23 |
| ESR1 | 1794.250246 | 67 |
| FOXA1 | 298.5511272 | 25 |
| GAPDH | 6219.377231 | 112 |
| GATA3 | 484.5721611 | 35 |
| HDAC1 | 478.9968222 | 45 |
| HIST2H2BE | 244.6629207 | 32 |
| HSP90AA1 | 899.141619 | 56 |
| IDO1 | 318.0474976 | 19 |
| MAP2K1 | 311.472058 | 37 |
| MAPK3 | 696.9443768 | 57 |
| PARP1 | 243.5500661 | 34 |
| PTPN11 | 259.6431584 | 27 |
| RHOA | 221.8554933 | 31 |
| RPS6KB1 | 411.6660202 | 31 |
| SMARCA4 | 377.0777492 | 34 |
| SNAI1 | 478.6696864 | 37 |
| STAT1 | 474.0392499 | 39 |
| TIMP1 | 193.6894915 | 24 |
| TLR2 | 236.5195706 | 33 |
| ZEB1 | 174.8820284 | 29 |
| doxorubicin | 252.2991472 | 31 |
| resveratrol | 330.698698 | 33 |
