# Supplementary material for: Cellular Mechanisms Triggered by the Cotreatment of Resveratrol and Doxorubicin in Breast Cancer: A Translational In Vitro–In Silico Model
Source: Oxid Med Cell Longev. 2020 Nov 1;2020:5432651. doi: 10.1155/2020/5432651 (PMC7654215; doi:10.1155/2020/5432651)
Supplement: Supplementary 8 — Supplementary Table 3 Experimental evidences assessing the modulation of putative genes by Rsv in breast cancer cells. We search for studies assessing the effect of Rsv in the 7 putative genes found through the in silico strategy. [file 5432651.f8.docx]

| **Gene/Protein** | **Cell type** | **Rsv treatment**  **(Concentration/Time)** | **Finding (method)** | **Ref.** |
| --- | --- | --- | --- | --- |
| CCND1 /  Cyclin D1 | MCF-7 cells  MCF7 cells  MDA-MB-231 and MCF-7 cells  MDA-MB-231 cells | 10 µM for 24h.  70 μM for 48h  100 µM for 24h;  100 µM for 48h.  50 μM for 24 h. | Down (WB).  Down (WB).  Down (WB);  Down (WB).  Down (RT-PCR). | [116]  [80]  [81]  [50] |
|  |  |  |  |  |
| CDH1 /  e-cadherin | SGC7901/DOX cells  MDA-MB-231 cells | 50 mg/l for 48h.  72 and 144 µM for 48h. | Up (WB).  Up (WB). | [89]  [90] |
|  |  |  |  |  |
| ESR1 /  ERα | HCC1806 cells  T-47D cells  MCF7cells | 15 μM for 72h.  80 μM for 24h.  0.5, 1 and 5 μM for 72h. | Up (WB)  Down (Real-time-PCR)  Down (WB) | [117]  [67]  [118] |
|  |  |  |  |  |
| MAPK3 /  ERK1 | MCF7 and MDA-MB-231 cells.  NALM-6 cells.  MCF-7.cells  MCF-7 cells | 10 μM for 4h  50 μM for 24h  10 μM for 30 min  1, 10 and 100 μM for 4h | Up (WB)  Down (WB)  Down (WB)  Up (WB) | [119]  [120]  [121]  [122] |
|  |  |  |  |  |
| PTPN11 /  SHP2 | 786-O cells  Fluo-3-AM-loaded platelets  Hela, SiHa and C33A cells | 10, 30 and 50 μM for 6h  10 μM for 5 min  100 μM for 48h | Up (WB)  Down (WB)  No change (WB and RT-PCR) | [123]  [124]  [125] |
|  |  |  |  |  |
| RPS6KB1  S6k | Cardiomyocytes  LY1 and LY18 cells  HEK293 cells  DU145 cells | 10 μM for 18h.  25 or 50 μM for 4h  12.5 – 100 μM for 30 min  50 µmol/ for 24h | Down (WB).  Down (WB)  Down (WB)  Down (WB) | [126]  [127]  [128]  [129] |
|  |  |  |  |  |
| HSP90AA1 /  HSP90A | Jejunal mucosa of black-boned chickens  MCF7 cells | 200, 400, 600 mg per kg/ 1-15 days.  10 μM for 72h | Down (WB and RT-PCR).  Down (WB). | [114]  [115] |

**Supplementary Table 3 – Experimental evidences assessing the modulation of putative genes by Rsv in breast cancer cells.** We search for studies assessing the effect of Rsv in the 7 putative genes found through the in silico strategy.

Abbreviations: WB, Western Blot;

**WB - Western blot analysis**
