# Supplementary material for: Cellular Mechanisms Triggered by the Cotreatment of Resveratrol and Doxorubicin in Breast Cancer: A Translational In Vitro–In Silico Model
Source: Oxid Med Cell Longev. 2020 Nov 1;2020:5432651. doi: 10.1155/2020/5432651 (PMC7654215; doi:10.1155/2020/5432651)
Supplement: Supplementary 2 — Supplementary Figure 2 Kinetics of acridine orange staining after Doxo treatment and cell growth after early autophagy inhibition. (A) Cells were treated with Doxo 100 nM for 24 h, followed by cell replating in a Drug-Free Medium. The percentage of AO-positive cells and the intensity of red fluorescence in AO-positive cells were determined 5, 10, and 15 days after treatment. (B) Autophagy was inhibited with 2 mM of 3-MA for 1 h before treatments. Cumulative Population Doubling was performed after 15 days. ∗p < 0.05, ∗∗p < 0.01, and ∗∗∗p < 0.001 in relation to control; #p < 0.05, ##p < 0.01, and ###p < 0.001 in relation to Doxo. [file 5432651.f2.pdf]

A

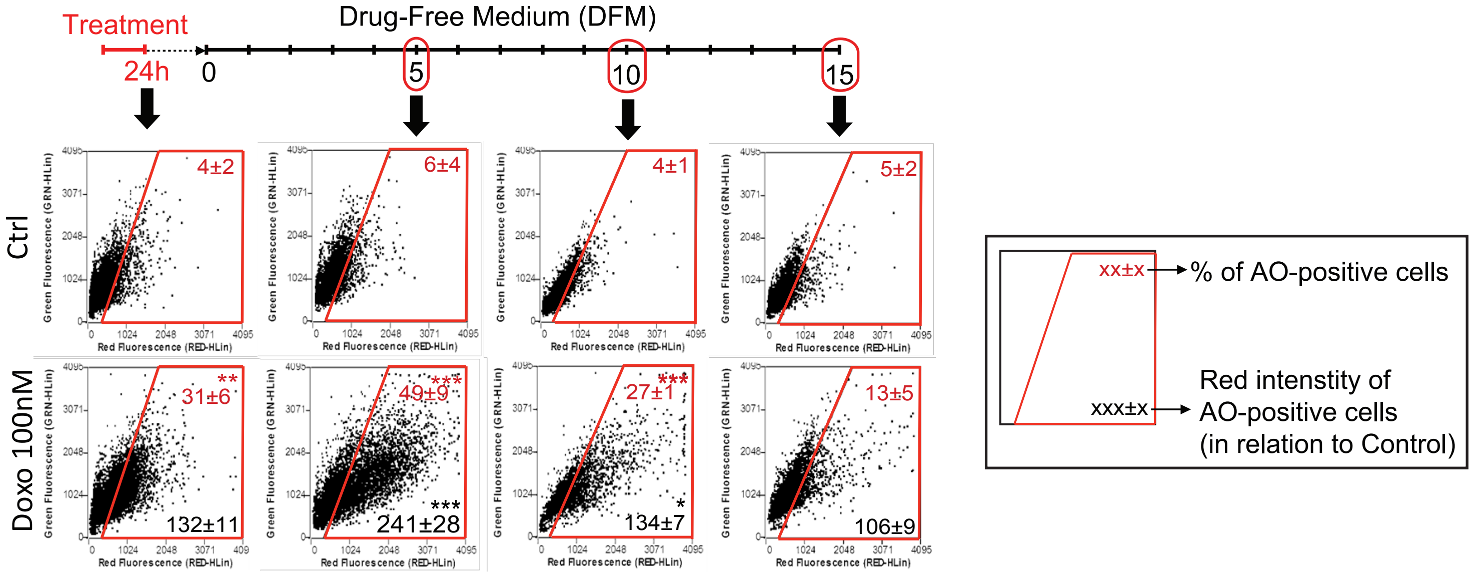

Treatment
24h
Drug-Free Medium (DFM)
0
5
10
15
Ctrl
Doxo 100nM
Green Fluorescence (GRN-HLin)
Red Fluorescence (RED-HLin)
4±2
6±4
4±1
5±2
** 31±6
*** 49±9
*** 27±1
13±5
132±11
*** 241±28
* 134±7
106±9
xx±x → % of AO-positive cells
xxx±x → Red intensity of AO-positive cells (in relation to Control)

B

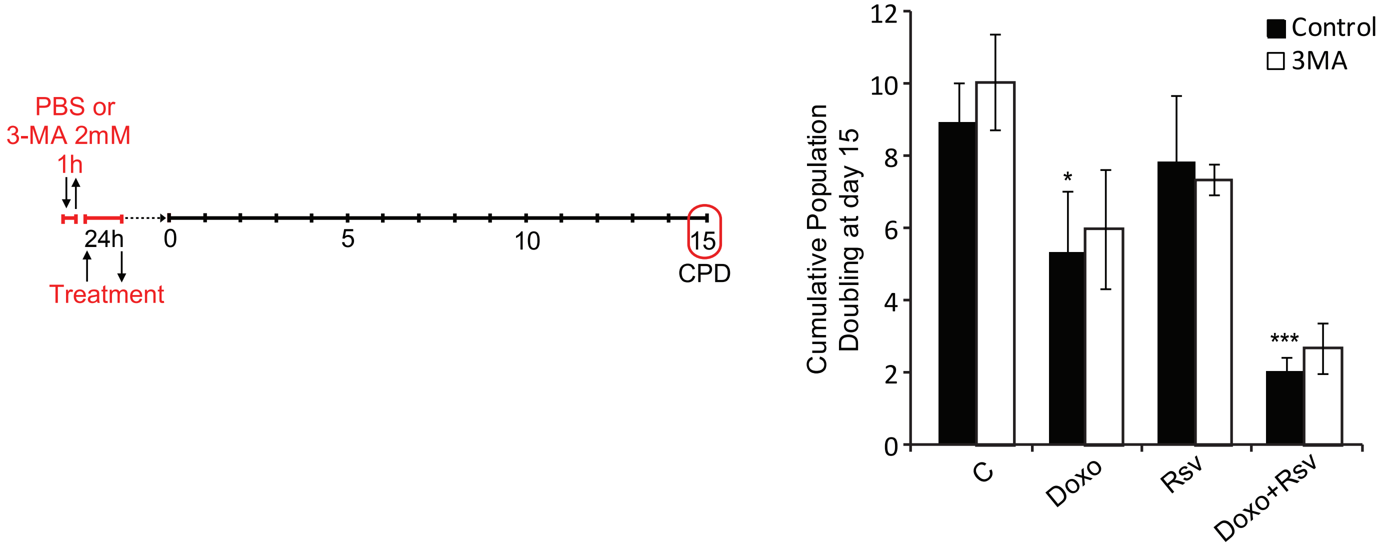

PBS or
3-MA 2mM
1h
24h
Treatment
0
5
10
15
CPD
Cumulative Population Doubling at day 15
Control
3MA
C
Doxo
Rsv
Doxo+Rsv
*
***
